# Crystallographic, Electronic Structure, and Computational Studies of PHOX–Ni Aryne Complexes: Origins of Regioselectivity in Metal-Bound Aryne Synthesis and Difunctionalization

**DOI:** 10.1021/jacsau.5c01159

**Published:** 2025-11-01

**Authors:** Alexander Umanzor, Nicholas A. Garcia, Kevin P. Quirion, Alex Lovstedt, Peng Liu, Courtney C. Roberts

**Affiliations:** † Department of Chemistry, 5635 University of Minnesota, Minneapolis, Minnesota 55455, United States; ‡ Department of Chemistry, 6614 University of Pittsburgh, Pittsburgh, Pennsylvania 15260, United States

**Keywords:** nickel, PHOX ligands, arynes, regioselectivity, selectivity models

## Abstract

Late transition metal aryne complexes are stable, isolable counterparts to free aryne intermediates. However, their utility has largely been limited since the Aryne Distortion Model (ADM) cannot be applied to substituted aryne complex reactivity, leading to nonselective reactions. Our group recently reported the first regioselective synthesis and difunctionalization of a CyPHOX–Ni *o*-methoxybenzyne complex. However, to increase the utility of these complexes in synthesis, their electronic structure, reactivity, and the impact of aryne substituents on selectivity must be understood. Herein, we report the first comprehensive experimental electronic structure study of aryne complexes, which has been carried out via UV/vis spectroscopy and cyclic voltammetry (CV) with an array of *o*-substituted arynes. CyPHOX–Ni aryne complexes exhibit a metal-to-ligand charge transfer (MLCT), and this transition as well as their oxidation potentials trend with Hammett parameters for the aryne substituents. To gain further insight into the origins of regioselectivity in CyPHOX–Ni aryne complex formation and difunctionalization, a combination of single-crystal X-ray crystallographic and density functional theory (DFT) structural studies were carried out. Our findings lead us to propose a Metal Aryne Reactivity/Selectivity (MAR/S) Model, which shows that CyPHOX–Ni aryne binding selectivity is governed by a combination of sterics and aryne distortion, whereas selectivity in functionalizations is directed by the phosphine *trans* influence.

## Graphical abstract

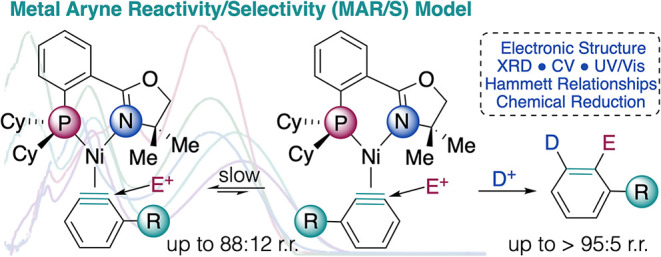

## Introduction

Arynes are reactive intermediates consisting of an aromatic ring with a triple bond that have been used in the synthesis of over 75 natural products.^1,2^ However, one limitation of unsymmetrically substituted free arynes is their substrate-controlled regioselectivity in reactions.^3^ The utility of *o-*substituted free aryne intermediates in synthesis has been expanded by the Aryne Distortion Model, developed by Garg and Houk (Figure 1a).^4–8^ In this pioneering model, it was shown that inductively electron-withdrawing substituents in *o-*substituted benzyne intermediates distort the internal aryne bond angles such that the *meta*-position is distorted toward linearity. This site has a larger internal angle and is the preferred site of nucleophilic functionalization. Greater internal angle differences are directly correlated with greater regioselectivities, allowing for more selective difunctionalizations (Figure 1a).

**Figure 1:**
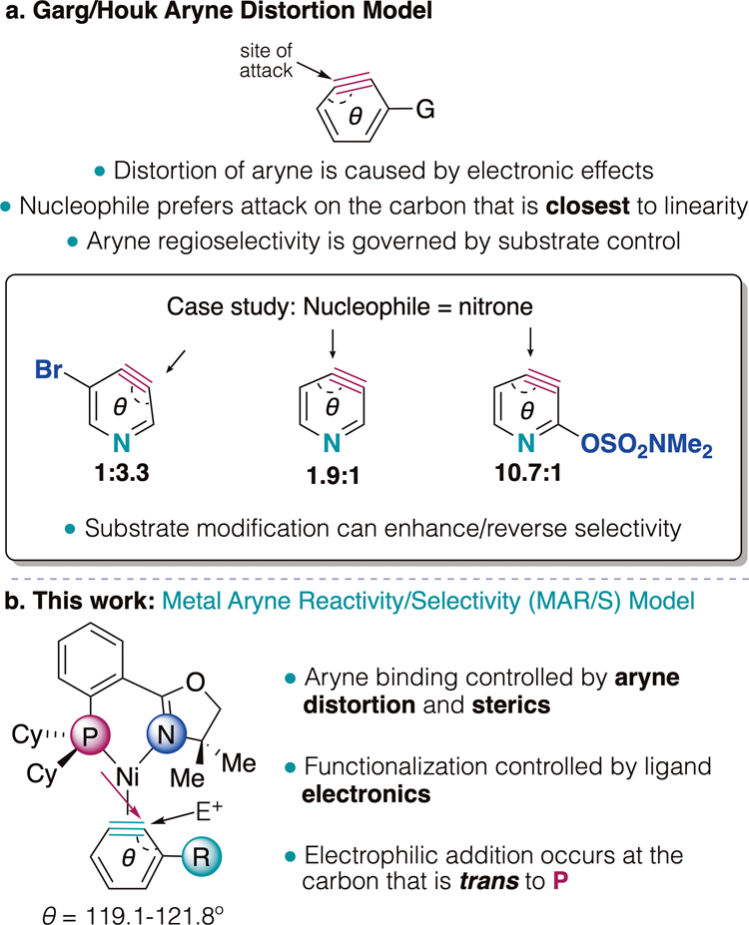
(a) Aryne Distortion Model for free arynes. (b) This work: Spectroscopic, crystallographic, and computational structural studies lead us to propose the Metal Aryne Reactivity/Selectivity (MAR/S) Model.

Late transition metal aryne complexes are isolable counterparts to free arynes which have been shown to exhibit umpolung reactivity as nucleophiles.^9,10^ They are invoked as intermediates in catalytic reactions and are capable of a wide array of reactivity.^11^ This makes them potentially useful synthons for rapidly accessing a variety of arene substitution patterns. However, unsymmetrically substituted aryne complexes often generate equimolar mixtures of regioisomeric products.^12–16^ For example, Hosoya and co-workers reported a difunctionalization of a (Et_3_P)_2_Ni *o*-methoxybenzyne complex, which gave a product ratio of 51:49.^17^ This is in stark contrast to the corresponding free aryne, which exclusively generates one regioisomer.^6^ Therefore, for aryne complexes to gain utility in synthesis, a platform must be developed to establish and predict their selectivity in reactions. The fact that they can be isolated, characterized, and reacted in a controlled stoichiometric manner allows for ease of study.^18–20^ Once this model is understood, selectivity can be further improved and implemented in Ni catalysis.

Thus, we sought to establish a system by which late transition metal aryne complexes could be synthesized and functionalized selectively. Our group recently reported the regioselective synthesis and difunctionalization of a CyPHOX–Ni *o*-methoxybenzyne, which showed that the concept of ligand control could be applied to influence *o-*substituted aryne complex selectivity.^21^ We now wanted to probe the electronic structure of an isostructural series of CyPHOX–Ni aryne complexes with varying aryne substituents to better understand the impact of substituent effects in combination with ligand effects on selectivity and reactivity. This was accomplished by studying these complexes via UV/vis spectroscopy, cyclic voltammetry, natural population analysis (NPA) calculations, and their correlations to linear free energy relationships. In this report, we propose the Metal Aryne Reactivity/Selectivity (MAR/S) Model, which has been developed using a combination of spectroscopy, crystallography, density functional theory (DFT), and reactivity studies (Figure 1b).

## Results and Discussion

### Synthesis and Crystallographic Studies of CyPHOX–Ni Aryne Complexes

Similarly to our previous studies, the synthesis of the aryne complexes **4-R** was accomplished via a three-step route involving (i) oxidative addition of the aryne precursor, (ii) CyPHOX ligand exchange, and (iii) transmetalation using NaO^*t*^Bu as an activator to form the aryne. All novel PPh_3_ σ-aryls **2-R** were isolable and characterized crystallographically via single-crystal X-ray diffraction (XRD). CyPHOX σ-aryls **3-CN, 3-Me, 3-Cl** and **3-F** were found to be isolable after steps (i) and (ii) and were characterized via XRD. As observed with the *o*-methoxy oxidative addition complex **3-OMe**, the unsubstituted σ-aryl (**3-H**) was not isolable and thus was subjected to an *in situ* ligand exchange followed by transmetalation to form **4-H**.^21^

With this scope of electronically varied arynes, the aryne complexes were observed in the solution state by ^31^P­{^1^H} NMR spectroscopy in regioisomeric ratios ranging from 67:33 to 88:12 (Figure 2b). In order to explain the relationship between our aryne regioisomers, ^31^P­{^1^H}–^31^P­{^1^H} NOESY experiments were employed. As was found with **4-OMe**, experiments show that the substituted arynes **4-R** all interconvert on the 2.5 s mixing time scale. This is indicative of slow interconversion between regioisomers. We hypothesized that a structural feature could correlate with the observed binding selectivities. Using the **4-R** solid-state structures (Figure 2c), the most significant feature exhibited between the crystal structures was the variable C1–C2 aryne bond lengths. It should be noted that the substituent will impact the electronics of both termini of the aryne through both resonance and inductive effects through the conjugated system. The arynes surveyed cover a wide range of C1–C2 bond lengths. **4-Me** (1.333(16) Å) is comparable to other late transition metal benzyne complexes reported in the literature (dcpe–Ni 1.332(6) Å, (Cy_3_P)_2_–Pd 1.324­(8) Å).^9,19^ However, the aryne bond length of **4-F** is significantly longer, at 1.365(9) Å. This approaches the double bond length of benzene (1.396 Å).^22^ The minor regioisomers were not observed universally across the substituted complexes **4-R** in the solid state; they manifested as rotational disorder for **4-Me**, **4-OMe**, and **4-Cl**, and were not observed for **4-CN** and **4-F**, the CyPHOX–Ni aryne complexes with the longest C1–C2 bond lengths. The Dewar-Chatt-Duncanson model has long been used to describe the bonding in metal π complexes.^23,24^ Crystallographic bond length comparisons can provide insight into the degree of π backdonation to the aryne π* orbitals. Our series of CyPHOX–Ni aryne complexes **4-R** exhibit a wide range of aryne C1–C2 bond lengths (1.329(6) to 1.365(9) Å). Since **4-CN** and **4-F** are thus closer to the metallacycloarene end of the spectrum, this increased degree of π backdonation to the aryne may induce a higher degree of rigidity that hinders aryne rotation. Examining other features of the crystal structures, the Ni–C2 distance is longer than the Ni–C1 distance across the series. This confirms that the *trans* influence of the P donor is stronger than that of the N donor. Consequently, C2 will likely be the more reactive site. Despite these observations, we were unable to find any trend that correlated with the aryne complex regioisomeric ratios. Analysis of the internal aryne angles (θ_C1_ and θ_C2_) also did not correlate with the regioisomeric ratios observed in the solution state despite this angle difference (Δ_θ_) being a computationally derived predictor in the Garg/Houk Aryne Distortion Model. This is likely due to crystal packing effects that distort bond lengths and angles.^25,26^

**Figure 2:**
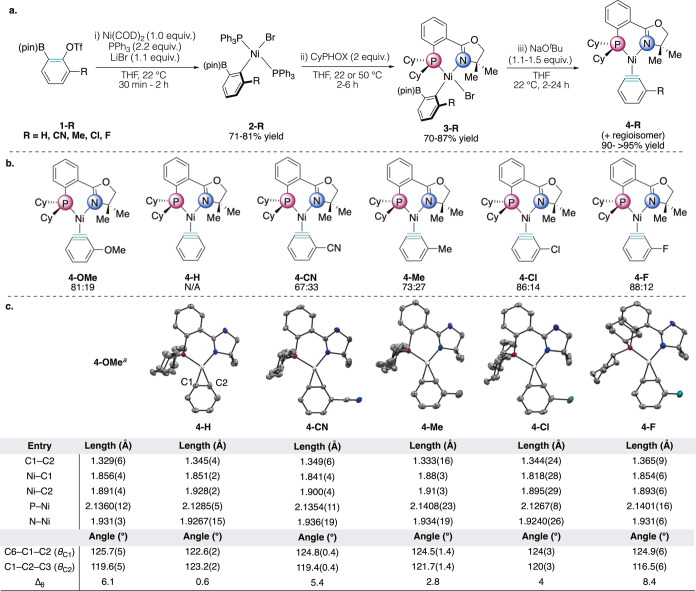
(a) Representative 3-step synthetic route toward CyPHOX–Ni aryne complexes. (b) Scope of CyPHOX–Ni aryne complexes in this study. (c) Single crystal X-ray crystal structures and select structural features of CyPHOX–Ni aryne complexes in this study. Note: For crystal structures with *Z*′ > 1 and/or disorder, the reported bond lengths and angles are the average of those in the asymmetric unit. ^a^ref 21.

### Electronic Structure Studies

Recently, our group reported that 5-membered Ni *N*-heteroaryne complexes can react as both nucleophiles and electrophiles.^27^ Therefore, it is important to understand the electronic structure of complexes **4-R** in order to inform reactivity studies. Because electronic structure studies in the solid state can be limited due to crystal packing effects, as well as the fact that only a single regioisomer was observed in the solid state for multiple complexes in our scope (R = CN, F), these factors posed limitations on the ability to find trends between structural and electronic features. Thus, we turned to solution-state studies, which can provide added insight into complex systems. In our previous studies with a CyPHOX–Ni *o-*methoxybenzyne **(4-OMe)**, a proposed weak metal-to-ligand charge transfer (MLCT) band was observed in the UV/vis spectrum. This MLCT band was also observed in all of our complexes **4-R**. We then proceeded to study the aryne substituent effects on the MLCT (Figure 3a). It was found that the MLCT transition across CyPHOX–Ni arynes lie in the cyan-green region of the visible light spectrum (466 nm < λ_max_ < 509 nm). It was found that the λ_max_ for the MLCT is strongly correlated to the σ_m_ Hammett parameter (Figure 3b, *R* ^2^ = 0.99). Thus, the amount of electron density at the *meta*-position (C1) has the greatest effect on the energy required for this transition. Regarding the intensity of the transition, **4-CN** (5950 M^–1^·cm^–1^ **)** exhibited the strongest MLCT, while **4-OMe** had the weakest extinction coefficient (ε = 457 M^–1^·cm^–1^). Interestingly, the extinction coefficient for the MLCT was found to be correlated with the σ_p+_ Hammett parameter (Figure 3c, *R* ^2^ = 0.99). From this trend, it appears that substituents that withdraw electron density through both resonance and inductive effects from the *ortho*-position (C2) correlate to a more intense charge transfer. Given the degree of correlation with Hammett parameters for the respective aryne substituents, we hypothesize that the MLCT involves the transfer of an electron from the Ni center to a π* orbital on the aryne. The σ_p+_ parameter accounts for stabilization of a cation at the benzylic position.^28^ Since this proposed electron transfer would leave the “benzylic” Ni center cationic and form an intermittent Ni^I^ species, this parameter most accurately describes the substituent effects on this transition. Additionally, due to the absence of d–d transitions across aryne complexes **4-R**, these spectral features preliminarily suggest a Ni(0) oxidation state. However, further studies (*vide infra*) were needed in order to support this hypothesis.

**Figure 3:**
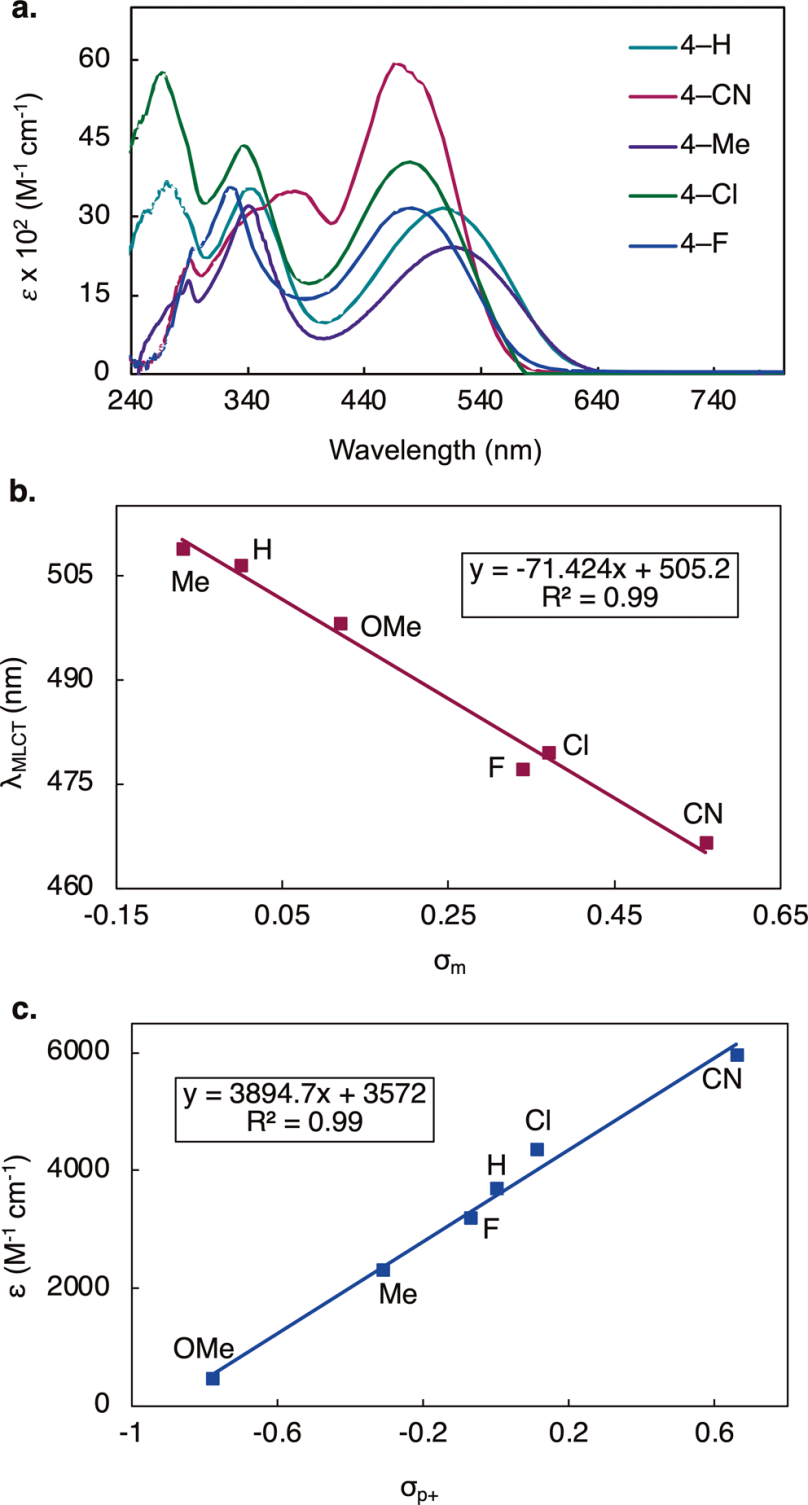
(a) UV/vis spectra of CyPHOX–Ni arynes **4-R** in this study. Spectra were collected on 0.3 mM samples under Ar in THF at 298 K. (b) Trend between the maximum absorbance wavelength λ_max_ for MLCT and the σ_m_ Hammett parameter for the respective aryne substituent in **4-R**. (c) Trend between the extinction coefficient ε for MLCT and the σ_p+_ Hammett parameter for the respective aryne substituent in complexes **4-R**.

Intrigued by the photophysical properties of **4-R**, we then moved on to study their redox properties. Like the previously studied **4-OMe**, the voltammograms of all the arynes exhibited a single, irreversible oxidation event (*E*_pa_) and a single, quasi-reversible reduction (*E*_pc_) in Figure 4a. The difference in oxidation potentials across the full **4-R** series was 470 mV and the difference in the reduction potentials was 160 mV. These differences in redox potentials highlight that the identity of the R group imposes significant effects on the electrochemical properties of complexes **4-R**. A summary of all oxidation and reduction potentials for **4-R** is found in Figure 4b. In contrast to the MLCT features, the oxidation and reduction potentials of these complexes were found to have disparate correlations to Hammett parameters. The σ_m_ parameter shows good correlation with the oxidation potential, suggesting that inductively electron-withdrawing groups lead to harsher oxidation potentials (Figure 4c, *R* ^2^ = 0.82). Conversely, the reduction potential exhibits a poor trend with both σ_p_ (SI Figure S124, *R* ^2^ = 0.43) and σ_p+_ (SI Figure S125, *R* ^2^ = 0.19).

**Figure 4:**
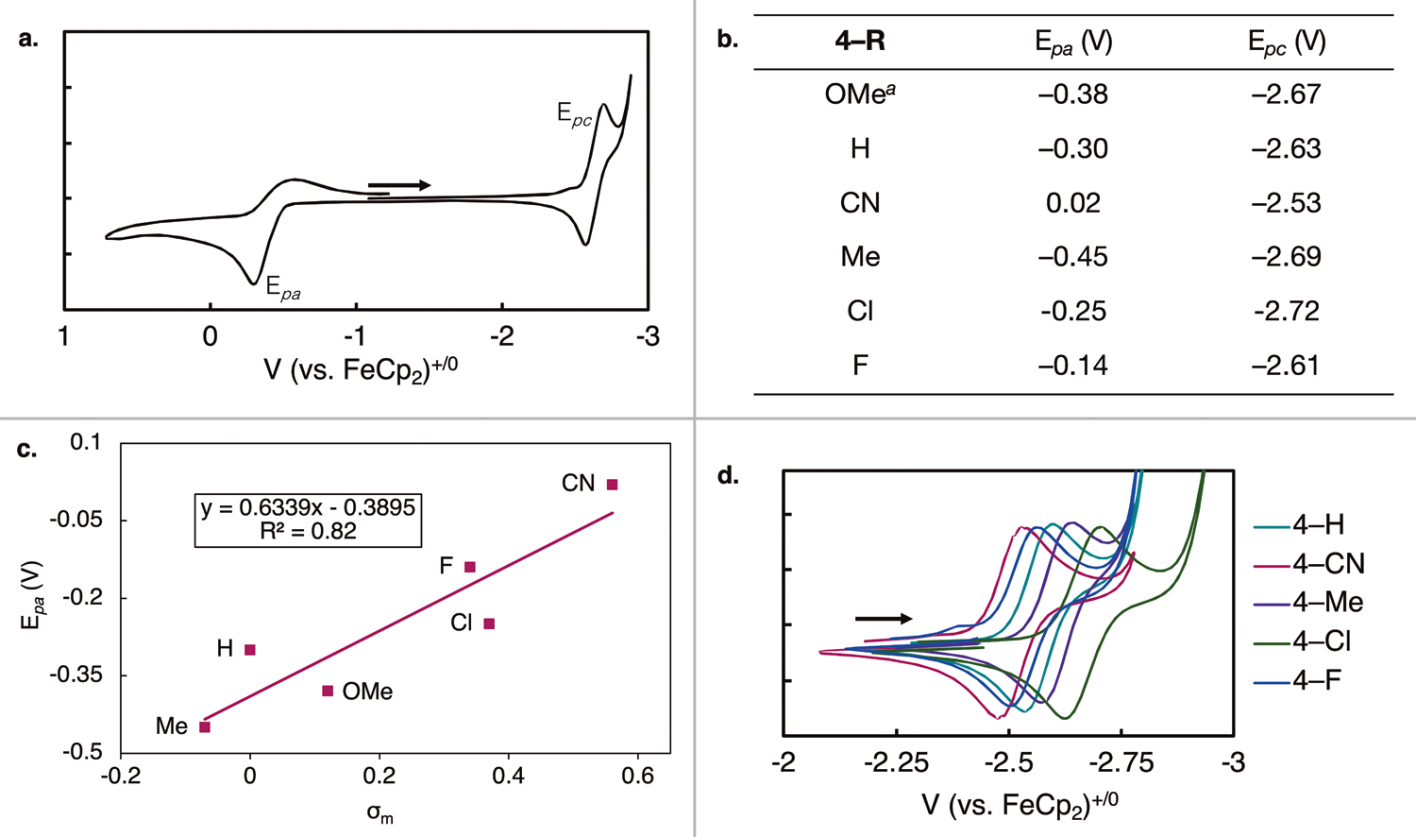
(a) Representative voltammogram displaying full electrochemical window events at 100 mV/s for **4-R** (**4-H** shown). CVs collected in 0.1 M [^n^Pr_4_N]­[BAr_4_^F^] electrolyte solutions in THF with 3 mM analyte under Ar. (b) Tabulated redox potentials for aryne complexes **4-R**. ^a^ref 21. (c) Hammett parameter trend between oxidation potential *E*_pa_ and σ_m_. (d) Overlay of reduction event *E*_pc._ for compounds **4-R** at 100 mV/s.

Considering the distorted trigonal planar geometry, diamagnetic character, and MLCT exhibited by complexes **4-R**, they are hypothesized to exist in the Ni^0^ formal oxidation state. Therefore, the presence of a reduction in the voltammograms warranted further investigation as it is likely not metal-based. When **4-Me** was compared to the CV of free CyPHOX ligand and **3-Me**, the latter only exhibited oxidation events related to the ligand and 2 reduction events presumed to correspond to Ni^I/II^ and Ni^0/I^ redox events (SI Figure S122).^29^ Thus, this reduction is unique to the aryne complexes **4-R**.

Therefore, we sought to chemically reduce our CyPHOX–Ni benzyne complex (**4-H**) to gain further insight into the identity of this reduced species, and whether the reduction is aryne- or CyPHOX-based. The unsubstituted complex **4-H** was chosen as a model system. Single-electron reduction of **4-H** with KC_8_ at −78 °C in THF with 18-crown-6 as an encapsulant cleanly produced complex **5-H**. A single new resonance in the ^31^P­{^1^H} NMR spectrum was observed at 69.0 ppm, which is downfield of the neutral species (38.3 ppm) (SI Figure S64). The aromatic region of the ^1^H NMR did not exhibit appreciable changes in the chemical shifts of the resonances. However, broadening of the resonances was observed and the chemical shift of the methylene protons on the oxazoline ring shifted significantly from 3.62 to 2.94 ppm. Crystals suitable for X-ray crystallographic analysis were grown from a saturated THF solution with vapor diffusion of pentane. The crystal structure of this complex showed a monoanionic CyPHOX–Ni benzyne complex (**5-H**) with K­(18-crown-6)­(THF)_2_ as the counterion (Figure 5a). The most dramatic structural change is seen in the imine C=N bond length (1.284(3) Å to 1.331(2) Å), suggesting partial reduction of this bond (Figure 5b). As expected, the radical character appears to be delocalized throughout the conjugated π system of the CyPHOX backbone, manifesting in alternating bond lengths of the aryl ring (SI Figure S96). The aryne C≡C bond only slightly lengthened (1.345(4) Å vs 1.349(2) Å), and there is an unexpected but substantial bond elongation of the C2–C3 bond (1.354(3) Å to 1.387(2) Å). The DFT-calculated spin densities show that the radical character is indeed delocalized throughout the CyPHOX ligand (Figure 5c). This manifests in the UV/vis spectrum of **5-H**, which shows that it is capable of similar transitions to the parent benzyne, but the former exhibits a more intense charge transfer at λ_max_ = 257 nm (ε = 10,140 M^–1^·cm^–1^). This represents, to our knowledge, the first reduced metal aryne complex. Considering that the MLCT is still present after reduction of the benzyne complex (SI Figure S105), this supports our hypothesis that this charge transfer involves transfer of an electron from the Ni center to the aryne. Interestingly, EDA-NOCV computational analyses carried out by Mondal and co-workers suggest an alternative bonding scenario in d^10^ metal aryne complexes to closed-shell σ-donation/π-backdonation described by the Dewar-Chatt-Duncanson model. Their studies show that a mix of σ-donation and an e^**‑**^ sharing π-bond between positively charged ligand–metal and negatively charged aryne fragments is another plausible bonding scenario that is at times energetically favored over purely dative bonding.^30^ Their studies suggest that enhancing the ability of the ligand–metal fragment to undergo electron transfer to the aryne fragment may be beneficial toward reactivity and stability. Thus, we postulate that this visible light-enabled MLCT observed for aryne complexes **4-R** may be helpful in stabilizing these complexes.

**Figure 5:**
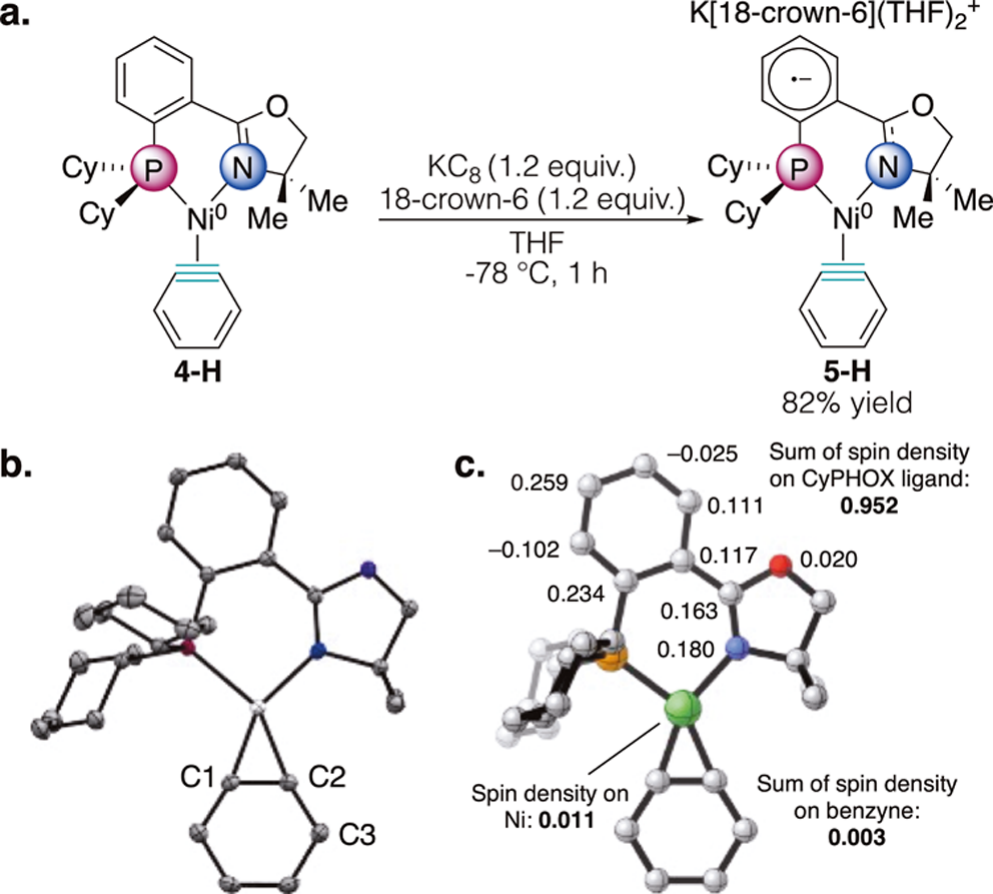
(a) Chemical reduction of **4-H** with KC_8_. (b) X-ray crystal structure of reduced CyPHOX–Ni benzyne complex **5-H**. K^+^[18-crown-6]­(THF)_2_ counterion omitted for clarity. (c) Calculated spin density of **5-H** shows that the radical character is localized on CyPHOX.

To further elucidate the electronic structure of complex **5–H**, X–Band EPR studies were conducted in fluid THF to elucidate the nature of the radical at 298 K. An anisotropic signal was observed with *g*_*x,y,z*_ values of 1.9954, 2.065, and 2.067, respectively (SI Figure S126). Resolved significant hyperfine coupling to the imine nitrogen (*A*_iso_ = (^14^N = 1, *n* = 1) = 159.7 MHz) and phosphine (*A*_iso_ = (^31^P = 1, *n* = 1) = 167.8 MHz) was observed. Additional hyperfine coupling was observed to the four distinct, inequivalent protons on the ligand backbone (*A*_iso_ = (^1^H, *n* = 1) = 34.9, 52.6, 54.2, and 70.6 MHz). No superhyperfine coupling was observed with the most abundant ^58^Ni nucleus (*I* = 3/2, 68.08%). These findings are consistent with a radical delocalized across the CyPHOX ligand backbone as observed in the computed spin–density plot (*vide supra*). Subjecting **4–H** to the same conditions resulted in no observable signal at 298 K, further supporting the closed–shell electronic structure of the proposed Ni(0) center.

### Origins of Binding Selectivity in CyPHOX–Ni Benzyne Complexes

Given that the solid-state structures did not provide significant insight into the observed regioisomeric ratios of complexes **4-R**, DFT calculations were employed to study the structural features of the metal aryne complexes and their impact on regioisomeric ratios. Whereas the solid-state studies were limited due to crystal packing effects, computational studies allow for modeling of species in the solution state, which may better represent the system. Thus, to elucidate the origins of CyPHOX ligand-induced selectivity as well as aryne substituent effects, the geometries of CyPHOX–Ni benzyne and substituted aryne regioisomers **4-R** were optimized at the B3LYP-D3/def2-SVP level of theory. The optimized geometries for the major (**4-F-a**) and minor (**4-F-b**) regioisomers of aryne complex **4-F** are shown in Figure 6a. Single point energies were calculated with ωB97X-D/def2-TZVP/SMD­(THF). Gibbs free energy differences (SI Figure S132) and quasi-harmonic corrected enthalpy differences (Δ*H*_qh_, Figure 6b) between regioisomers calculated at this level of theory were found to correlate well with the experimentally observed regioisomeric ratios, which suggests that this is a valid level of theory for modeling these complexes.^31,32^

**Figure 6:**
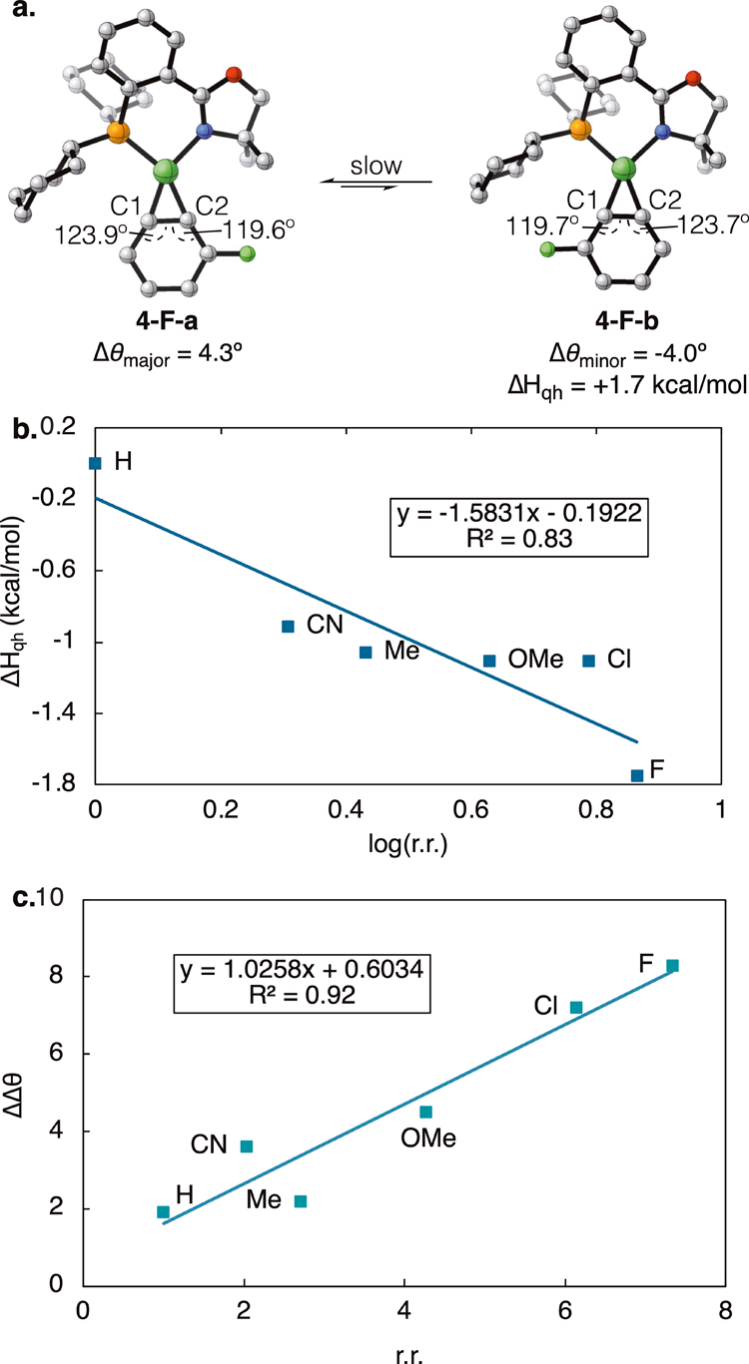
(a) Optimized geometries of regioisomers of aryne **4-F** and their internal aryne angle and energy differences. (b) Correlation between experimentally observed regioisomeric ratios of aryne complexes and calculated quasi-harmonic corrected enthalpy difference between regioisomers (Δ*H*_qh_). (c) Correlation between calculated ΔΔθ (internal aryne angle differences) vs experimentally observed regioisomeric ratios for CyPHOX–Ni aryne complexes.

In order to decouple the effects of the CyPHOX ligand and the aryne substituent on binding selectivity, benzyne complex **4-H** was used as a benchmark. It was found to exhibit an appreciable CyPHOX ligand-induced distortion present in the internal aryne angles (θ_C1_ and θ_C2_) leading to an angle difference Δθ = θ_C1_ – θ_C2_ = 1.9°, where θ_C2_ is the smaller internal angle (that which is *trans* to phosphine). The major regioisomers of the substituted aryne complexes followed this same trend but exhibited varying degrees of additional aryne distortion–while major regioisomer **4-F-a** features the greatest angle difference of Δθ_major_ = 4.3°, **4-Me-a** only has a 1.8° difference (SI Figure S127). Interestingly, the opposite is true for the calculated geometries of all minor aryne regioisomers **4-R-b** in this study. That is, placing the substituent on the phosphine side of the CyPHOX ligand results in the internal aryne angle θ_C1_ becoming the smaller internal angle (Figure 6a). In order to include both regioisomers in our model, we determined the difference in angle distortion ΔΔθ between each pair of substituted aryne regioisomers, where ΔΔθ = Δθ_major_ – Δθ_minor_. Gratifyingly, similar to the Aryne Distortion Model, this ΔΔθ parameter was found to have a strong correlation with the experimentally observed regioisomeric ratios (*R* ^2^ = 0.92, Figure 6c). It appears that for the major regioisomers, the ligand and aryne distortion are “matched,” to yield the lowest energy conformation. On the other hand, in the case of the minor regioisomer, the ligand and aryne distortion are “mismatched,” and the aryne substituent erodes the CyPHOX ligand-induced distortion to make θ_C1_ the smaller internal angle (SI Figure S127). Furthermore, the greater the minor regioisomer geometry (**4-R-b**) deviates from the major (**4-R-a**), the higher the energy penalty, leading to higher regioisomeric ratios. Our model suggests that the CyPHOX ligand-induced aryne distortion is a governing effect that can be tuned to further improve selectivities and is necessary for Ni aryne complexes to be synthesized and functionalized selectively.

In studying the electronic structure of CyPHOX–Ni aryne complexes, HOMO/LUMO and natural population analysis (NPA) charge analysis was performed in order to gain further insight into reactivity. These calculations show that the HOMO across all substituted aryne complexes **4-R** is aryne-based (SI Figure S130). The LUMO is imine-based (SI Figure S132). An appreciable NPA charge difference (Δ*q*_NPA_ = |*q*_C2_ – *q*_C1_|) was observed for all aryne complexes across the aryne carbons. As a charge-controlled model had once been postulated for free arynes,^33,34^ we evaluated this possibility against our distortion model. The Δq_NPA_ values for the major regioisomers **4-R-a** also showed a strong trend with our observed selectivities (SI Figure S131). Thus, distortion-interaction analysis was needed in order to determine whether binding selectivity in this CyPHOX–Ni aryne system is governed by distortion or atomic charge control.

In order to determine the phenomenon that controls binding selectivity, distortion-interaction analysis was carried out on the aryne complexes **4-R**.^35,36^ The interaction energy differences (ΔΔ*E*_interaction_) between regioisomers are much smaller than the distortion energy differences between regioisomers (ΔΔ*E*_distortion_) (SI Figure S128). These results indicate that the regioselectivity is mainly controlled by distortion energies. Further decomposition of the distortion energies indicates that both the CyPHOX–Ni and aryne fragments are slightly more distorted in the minor regioisomers (SI Figure S129). This supports that the steric repulsions between the bulkier phosphine arm of the C1-symmetric ligand and aryne substituent are among the factors that contribute to regioinduction (SI Figure S127).

### Benchmarking the Translation of Selectivity from Aryne Binding to Difunctionalizations

Our group previously reported that upon difunctionalization of **4-OMe**, the arene product ratio (r.r. 88:12) was slightly enhanced compared to the observed aryne regioisomeric ratio (r.r. 81:19). In order to apply our knowledge of selectivity to reactivity, we hypothesized that the product ratios of difunctionalizations would continue to be in agreement with the ratios of the aryne complexes across the series **4-R**. Therefore, we subjected the substituted arynes **4-R** to methyl triflate (MeOTf) followed by deuterated trifluoroacetic acid (TFA-*d*), to give the corresponding difunctionalized products (Figure 7a). **4-Me** gave the corresponding products in 79:21 r.r., once again showing good retention in selectivity from aryne to arene product. Excitingly, the difunctionalizations of **4-Cl** and **4-F** were found to produce a single regioisomer. **4-CN** was found to not be compatible with this sequence, as MeOTf is known to methylate nitriles and methyl iodide was not a strong enough methylating source, which agrees with the CV data which shows that **4-CN** exhibits the harshest oxidation potential.^37^ These difunctionalizations proceeded with low yields, primarily due to the rather inert nature of the methylated intermediates.

**Figure 7:**
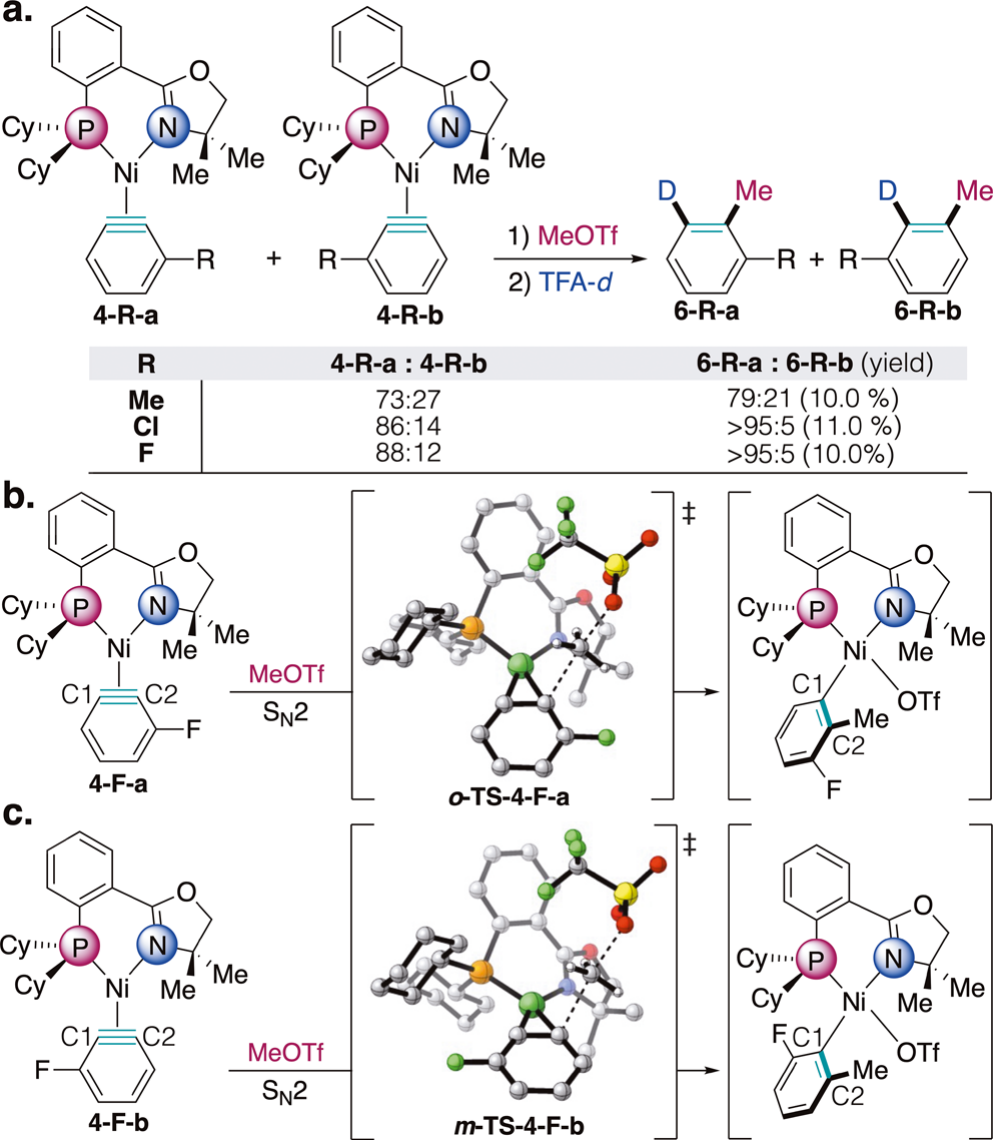
(a) Difunctionalization of CyPHOX–Ni aryne complexes **4-Me**, **4-Cl**, and **4-F** via methylation/deuteration shows retention in selectivity from aryne complexes to arene products. Yields calculated via ^2^H NMR using CD_2_Cl_2_ as an internal standard. Calculated methylation transition states originating from (b) the major regioisomer **4-F-a**. and (c) the minor regioisomer **4-F-b**. Transition states were calculated at the ωB97X-D/def2-TZVP/SMD­(THF)//B3LYP-D3/def2-SVP level of theory.

Having validated that our unsymmetrical ligand-controlled approach could be leveraged to not only synthesize aryne complexes regioselectively but maintain selectivity in difunctionalizations, we wanted to further investigate the relative contributions of phosphine *trans* influence- and aryne-induced distortion on selectivity in these difunctionalizations. Thus, transition state calculations were carried out at the ωB97X-D/def2-TZVP/SMD­(THF)//B3LYP-D3/def2-SVP level of theory for the methylations of both regioisomers of aryne complexes **4-Me** and **4-F** at each terminus of the aryne (SI Figures S134–137). The methylation with the major regioisomer **4-F-a** prefers the *ortho-*methylation at the C2 position, *trans* to the phosphine (***o*** **-TS-4-F-a**). This process proceeds via a direct S_N_2-type reaction between the aryl carbon and the MeOTf electrophile to furnish a methylated σ-aryl intermediate, which is consistent with previous computational studies on methylation of (dcpe)­Ni–benzynes by Hosoya.^38^ By contrast, *meta-*methylation of **4-F-a** at the C1 position, *trans* to oxazoline, is less favorable—the same direct S_N_2-type transition state could not be located, whereas the alternative MeOTf oxidative addition process has a 4.6 kcal/mol higher barrier in energy (SI Figure S136). Similar calculations with the minor regioisomer **4-F-b** show that the *meta-*methylation of C2, which is now positioned *trans* to the phosphine (Figure 7c), has a 4.2 kcal/mol lower barrier than the *ortho-*methylation *trans* to oxazoline (SI Figure S137). Our computational studies suggest the phosphine *trans* influence governs the regioselectivity in aryne complex difunctionalizations, with the preferred methylation occurring trans to the phosphine ligand in the reactions with both regiosiomers of the aryne.

Given the isomerization of the aryne complexes **4-R** observed via ^31^P­{^1^H}–^31^P­{^1^H} NOESY as well as the rotational disorder between several regioisomer pairs observed *in crystallo*, the transition state for this isomerization process was calculated for **4-F**, and was found to have a 31.6 kcal/mol energy barrier (SI Figure S138). This high kinetic barrier leading to slow isomerization as well as the modest yields in these model difunctionalizations are likely contributors for the selectivities in the difunctionalizations being in good agreement with the aryne ratios, but not identical.

### Development of the Metal Aryne Reactivity/Selectivity (MAR/S) Model

With the combined experimental, spectroscopic, and computational insights gathered from this study, we propose the Metal Aryne Reactivity/Selectivity (MAR/S) Model. For unsymmetrically substituted CyPHOX–Ni benzynes, the binding selectivity is controlled by an interplay of ligand and aryne substituent-induced distortion. These two effects are matched in the major regioisomer to minimize ring strain, compounding on C2 to result in both a longer Ni–C2 bond and a smaller internal angle θ_C2_, respectively (Figure 8a). This orientation also minimizes the steric interactions between the aryne substituent and the ligand. Ultimately, it is selective for *ortho-*methylation at the C2 position, leading to formation of major product **6-R-a**, as supported by our transition state analysis for the methylations (*vide supra*). In the minor regioisomer, the ligand and aryne substituent-induced distortion are mismatched, acting on C2 to produce a longer Ni–C2 bond and a smaller internal angle θ_C1_, respectively (Figure 8b). This binding orientation is generally higher in energy due to a combination of higher ring strain as well as steric interactions between the aryne substituent and the bulky cyclohexyl groups of the phosphine donor. However, it demonstrates that the *trans* influence is the predominant factor in maintaining selectivity from aryne complex to arene product, as it is selective for *meta-*methylation to produce the minor product **6-R-b**.

**Figure 8:**
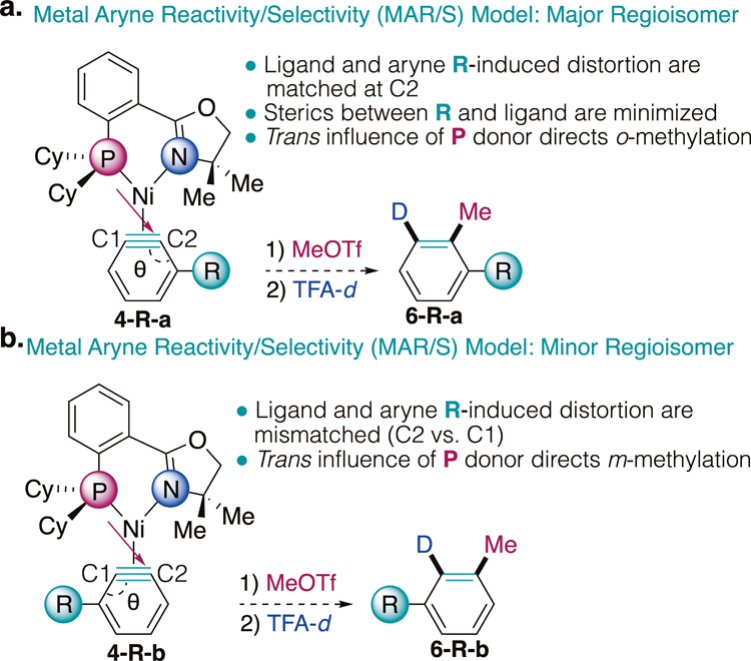
MAR/S Model for the (a) major and (b) minor regioisomers of CyPHOX–Ni benzyne complexes.

## Conclusion

In summary, we have carried out the first comprehensive experimental electronic structure study across a series of CyPHOX–Ni aryne complexes. While X-ray crystallographic studies showed important trends regarding π-backdonation and the *trans* influence of the phosphine, no structural feature was found to correlate to the aryne complex regioisomeric ratios. CyPHOX–Ni aryne complexes exhibit intense MLCT absorbances, the properties for which are well-described by Hammett parameters (ε by σ_p+_ and λ_max_ by σ_m_, respectively). In terms of their redox properties, they are capable of an irreversible one-electron oxidation and a quasi-reversible, one-electron, ligand-centered reduction event. Trends in oxidation potentials are well-described by Hammett parameters (σ_m_ for *E*_pa_). We have probed the reduction experimentally, and an anionic benzyne complex has been synthesized, characterized, and studied computationally. This finding leads us to propose a Ni^0^ oxidation state for CyPHOX–Ni benzyne complexes.

In addition to the electronic structure studies, DFT and NMR spectroscopic studies have allowed us to develop the MAR/S Model. Selectivity in aryne formation is a result of a combination of sterics and CyPHOX ligand and aryne substituent-induced aryne distortion. Similarly to the Aryne Distortion Model, calculated geometries show that greater ΔΔθ values correlate with higher CyPHOX–Ni aryne complex regioisomeric ratios. Furthermore, due to the slow interconversion of the substituted arynes and the phosphine *trans* influence, difunctionalizations proceed with excellent retention of selectivity from aryne to arene products. However, unlike the Aryne Distortion Model, the relative internal angles do not dictate the site selectivity in functionalizations. Transition state analysis performed on the methylations at each aryne terminus for arynes **4-Me** and **4-F** support that electrophilic addition consistently occurs at the position *trans* to phosphine for both aryne regioisomers. This governing phenomenon essentially leads to regiospecific difunctionalizations with respect to the aryne complex regiochemistry. Interestingly, the ligand-induced aryne distortion contribution was found to be very consistent across the unsymmetrically substituted aryne complexes surveyed, suggesting that ligand-induced distortion can be tuned via ligand design to further improve selectivities. CyPHOX proved to be a helpful first-generation unsymmetrical ancillary ligand for stabilizing Ni aryne complexes for these electronic structure studies and uncovering the origins of selectivity in their synthesis and difunctionalization. Future work will focus on ligand modifications to expand the reactivity of Ni aryne complexes based on this conceptual framework, improving selectivities, and applications to catalytic reactivity.

## Supplementary Material



## Notes

Experimental details and characterization of all new compounds (NMR, UV–vis, CV, etc.) (PDF)

## Abbreviations

Cy: cyclohexyl
PHOX: phosphinooxazoline
COD: cyclooctadiene
