# Supplementary material for: Crystallographic, Electronic Structure, and Computational Studies of PHOX–Ni Aryne Complexes: Origins of Regioselectivity in Metal-Bound Aryne Synthesis and Difunctionalization
Source: JACS Au. 2025 Nov 1;5(11):5656–64. doi: 10.1021/jacsau.5c01159 (PMC12648280; doi:10.1021/jacsau.5c01159)
Supplement: Supplementary file 1 [file au5c01159_si_001.pdf]

Supporting Information

Crystallographic, Electronic Structure, and Computational Studies of PHOX-Ni Aryne Complexes: Origins of Regioselectivity in Metal-Bound Aryne Synthesis & Difunctionalization

Alexander Umanzor[a], Nicholas A. Garcia[a], Kevin P. Quirion[b], Alex Lovstedt[a], Peng Liu[b], and Courtney C. Roberts[a]**

[a] Department of Chemistry, University of Minnesota – Twin Cities, Minneapolis, 207 Pleasant St SE, Minneapolis MN 55455, United States

[b] Department of Chemistry, University of Pittsburgh, Pittsburgh, PA 15260, United States

***Corresponding Author**
Email: ccrob@umn.edu
Email: pengliu@pitt.edu

Table of Contents

General Experimental Details…………………………………..……………………..S3-S4
Synthesis and Characterization of Substrates 1-R………………………………..S4-S5
Synthesis and Characterization of PPh_3 Sigma Aryl Complexes 2-R……..…..S6-S7
Synthesis and Characterization of PHOX Sigma Aryl Complexes 3-R…..........S8-S9
Synthesis and Characterization of PHOX Aryne Complexes 4-R……………..S10-S12
Synthesis and Characterization of Reduced PHOX Benzyne 5-H…………………S13
Synthesis of 6-R-ab/Difunctionalization of Complexes 4-R…………………....S14-S15
NMR Spectra of Compounds…………………………..……………..…………..S16-S79
NMR Spectra and GC/MS Data for Difunctionalizations………………………...S80-S90
Crystallographic Data for 2-R…………….…………….……………….….…......S91-S98
Crystallographic Data for 3-R…………….…………….………………….…..S99-S107
Crystallographic Data for 4-R…………….…………….…………………...…..S108-S123
Crystallographic Data for 5-H…………….…………….………………………S124-S126
UV/Vis Spectra……………………………………………..……………..…….…S127-131
Cyclic Voltammetry…………………..………………..…………………..………..S131-144
EPR Spectroscopy…………………..………………..………………..…….........S145
Computational Data……………………………………………………………….S146-154
Cartesian Coordinates……..………………………………………….………… S154-S223
References…………………………………………..……………..……….S224-S226

General Experimental Details

Unless specified, all chemical transformations were performed in a glovebox or using standard Schlenk line techniques under N_2. (3-Cyano-2-hydroxyphenyl)boronic acid was ordered from Oakwood and used as received. 2-Chloro-6-(4,4,5,5-tetramethyl-1,3,2-dioxaborolan-2-yl)phenol and (3-Fluoro-2-hydroxyphenyl)boronic acid were ordered from Ambeed and used as received. 4,4-Dimethyl-2-phenyl-2-oxazoline was ordered from Sigma-Aldrich and Santa Cruz Biotechnology, Inc. and used as received. The following were purchased from Sigma Aldrich and used as received: Pinacol, N,N-diisopropylethylamine, *sec*-butyllithium (1.4 M solution in cyclohexane), triphenylphosphine, *o*-xylene, *m*-xylene, 3-chlorotoluene. Lithium bromide was purchased from Sigma Aldrich and dried under vacuum at 110 °C for 72 h before use. 18-crown-6 was purchased from Sigma Aldrich and recrystallized from acetonitrile, then dried under vaccum at 22 °C for 24 h. The following were purchased from Tokyo Chemical Industry (TCI) and used as received: trifluoromethanesulfonic anhydride, chlorodicyclohexylphosphine, 2-chlorotoluene, 2-fluorotoluene, 3-fluorotoluene. $Ni(COD)_2$ was used as is from Strem Chemicals. Sodium *tert*-butoxide was purchased from Oakwood Chemical and used as received. Methyl trifluoromethanesulfonate was purchased from Sigma-Aldrich and stored over molecular sieves. Pentane, diethyl ether, benzene, toluene tetrahydrofuran, acetonitrile and dichloromethane were obtained from J.T Baker and dried on a Pure Process Technology solvent purification system and deoxygenated by sparging with Ar gas for at least 1 hour. Substrates **1-H**[1]**, 1-Me**[2] and **CyPHOX**[3] were prepared according to literature procedures. $CDCl_3$, CD_2Cl_2, CD_3CN, C_6D_6, and C_7D_8, were ordered from Cambridge Isotope Libraries, Inc. and THF-d_8 was purchased from Sigma Aldrich, all of which were stored over 4Å molecular sieves. Trifluoroacetic acid-*d* was also purchased from Sigma Aldrich and used as received.

^1H, ^2H, ^{11}B, $^{13}C\{^1H\}$, $^{19}F\{^1H\}$ and $^{31}P\{^1H\}$ NMR spectra were recorded on Bruker AVANCE 400 MHz spectrometers at 298 K. Chemical shifts are referenced to residual protiosolvents: 7.26 ppm (C*H*Cl3), 7.16 ($C_6\mathit{H}_6$), 5.32 ($C\mathit{H}Cl_2$) and 3.58 ($OC_4D_7\mathit{H}$) for ^1H NMR; 128.06 (C_6D_6) and 67.21 ($O\mathit{C}_4D_8$) for ^{13}C. ^2H NMR shifts are referenced to CD_2Cl_2. $^{31}P\{^1H\}$ NMR shifts are referenced to a 85% H_3PO_4 internal standard. MS Data was collected on a Bruker BioTOF II ESI/TOF-MS, a Sciex X500R UPLC/QTOF-MS, and an Agilent 7200 GC/QTOF-MS.

X-ray data were collected using a Bruker Photon III CPAD diffractometer for data collection at 125(2) K using Mo Kα radiation (normal parabolic mirrors). The data intensity was corrected for absorption and decay (SADABS). Final cell constants were obtained from least-squares fits of all measured reflections and the structure was solved and refined using SHELXL-2014/7.39. All non-hydrogen atoms were refined with anisotropic displacement parameters. All hydrogen atoms were geometrically placed. Details regarding refined data and cell parameters are available in Table S1-S14.

UV/Vis spectra were recorded in THF at 298 K at 0.3 M and 1 mM concentrations. Cyclic voltammograms were collected at 298 K with a CH Instruments 600 electrochemical analyzer with a one–cell setup, comprising of a 3 mm glassy carbon working electrode (polished using an alumina slurry)[4], a platinum wire counter electrode, and Ag wire pseudo–reference electrode. Analytes (3 mM) were dissolved in 0.1 M $[^nPr4N][BAr^F4]$ (BAr^F4 = tetrakis(3,5-bis(trifluoromethyl)phenyl)borate)) electrolyte solutions in THF, and internally referenced to the $FeCp_2/FeCp_2^+$ redox couple. Cyclic voltammograms are plotted in the polarographic convention. Voltammograms were initiated at the measured open circuit potential and scanned reductively. Peak currents are normalized with respect to scan rates by using the peak ratio between the observed current and the square root of the scan rate in V/s.

EPR spectroscopy was performed on an Elexsys E500 continuous wave spectrometer operating at an X–band of 9 GHz in the EPR Facility at the Biophysical Technology Center at UMN. Simulations of EPR spectra were executed in MATLAB 9.11 using EasySpin 6.0.0.

Synthesis and Characterization of Substrates 1-R

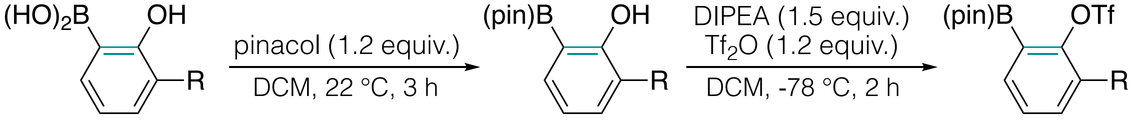

1

Scheme S1. Synthetic route towards borylaryl triflate precursors from *o*-hydroxyboronic acids.[2] **1-H**[1] and **1-Me**[2] were synthesized according to literature procedures and the chemical shifts of the ^{1}H NMR spectra were consistent with those reported in the literature.

General Procedure A: To an oven-dried 100 mL round bottom flask charged with a stir bar was added boronic acid (6.4 mmol) and pinacol (908 mg, 7.68 mmol, 1.2 equiv.), which were dissolved in 25.6 mL dry dichloromethane (250 mM). The reaction was allowed to stir under N_2 at 22 °C for 3 hours. To the reaction was added 5 mL of water and stirred for 5 mins. The layers were then separated, and the aqueous layer was extracted 3 times with 8 mL DCM. The organic fractions were combined, dried over sodium sulfate, and transferred to an oven-dried round bottom flask before being concentrated under reduced pressure.

Upon isolation, the crude boronic ester was redissolved in 25.6 mL dry dichloromethane (250 mM) and the flask was charged with a stir bar and placed under N_2. To the flask was then added diisopropylethylamine (1.67 mL, 9.6 mmol, 1.5 equiv.) before cooling the flask to - 78 C for 15 mins. Trifluoromethanesulfonic anhydride (1.29 mL, 7.68 mmol, 1.2 equiv.) was then added dropwise to the reaction, which was allowed to stir for 2 h before quenching by the addition of 5 mL water. The layers were then separated, and the aqueous layer was then extracted 3 times with 8 mL DCM. The

organics were combined, washed with a saturated solution of sodium chloride, and then dried over sodium sulfate before being concentrated under reduced pressure. The crude oily solids were then dissolved in hexanes, passed through a silica plug, and reconcentrated to remove the ammonium triflate salt from the product. The boryl triflate product was then purified via flash column chromatography and azeotroped with benzene before use.

1-CN: Prepared using General Procedure A. Crude **1-CN** was purified via flash column chromatography using a gradient of hexanes to 10:1 hexanes:ethyl acetate to give 1.25 g of an orange crystalline solid (52% yield.)
^{1}H NMR (400 MHz, CDCl3, 298 K) δ 8.09 (dd, *J* = 7.5, 1.9 Hz, 1H), 7.81 (dd, *J* = 7.7, 1.9 Hz, 1H), 7.50 (t, *J* = 7.6 Hz, 1H), 1.38 (s, 12H).
^{11}B NMR (128 MHz, CDCl3, 298 K) δ 29.8.
^{13}C{^{1}H} NMR (101 MHz, $CDCl_3$, 298 K) δ 154.1, 141.4, 136.8, 128.1, 118.9 (q, *J* = 320.9 Hz) 114.0, 107.1, 85.5, 24.9. The signal for the carbon attached to the boron atom was not observed.
^{19}F{^{1}H} NMR (376 MHz, $CDCl_3$, 298 K) δ -72.9.
HRMS (ESI-MS) m/z= 400.06220 [$C_{14}H_{15}BF_3NO_5SNa$]$^+$ (Calculated 400.06081 for [M+Na]$^+$)

1-Cl: Prepared using General Procedure A from the corresponding 2-hydroxy pinacolboronic ester on 3.93 mmol scale. Crude 1-Cl was purified via flash column chromatography using hexanes to give 1.52 g of a colorless oil (81% yield.)
^{1}H NMR (400 MHz, $CDCl_3$, 298 K) δ 7.73 (dd, *J* = 7.4, 1.8 Hz, 1H), 7.56 (dd, *J* = 7.9, 1.8 Hz, 1H), 7.31 (t, *J* = 7.7 Hz, 1H), 1.37 (s, 12H).
^{13}C NMR (101 MHz, $CDCl_3$, 298 K) δ 149.7, 135.1, 133.9, 128.6, 126.7, δ 118.8 (q, *J* = 320.8 Hz), 85.1, 25.0. The signal for the carbon attached to the boron atom was not observed.
^{19}F NMR (376 MHz, $CDCl_3$, 298 K) δ -73.5.
^{11}B NMR (128 MHz, CDCl3, 298 K) δ 30.1.
HRMS (GC-MS/EI) m/z 386.0574 [$C_{13}H_{15}BClF_3O_5S$] (Calculated 386.0374 for [M])

1-F: Prepared using General Procedure A. Crude **1-F** was purified via flash column chromatography using a gradient of hexanes to 10:1 hexanes:ethyl acetate to give 1.2 g of a colorless oil (50% yield.)
^{1}H NMR (400 MHz, $CDCl_3$, 298 K) δ 7.61 (ddd, *J* = 6.8, 2.2, 1.2 Hz, 1H), 7.39 – 7.26 (m, 2H), 1.37 (s, 12H).
^{11}B NMR (128 MHz, $CDCl_3$, 298 K) δ 29.6.
^{13}C{^{1}H} NMR (101 MHz, $CDCl_3$, 298 K) δ 153.4 (d, *J* = 253.4 Hz), 141.2 (d, *J* = 12.1 Hz), 131.8 (d, *J* = 4.0 Hz), 129.1 (d, *J* = 6.8 Hz), 120.2 (d, *J* = 19.0 Hz), 118.9 (q, *J* = 320.7 Hz), 85.1, 24.9. The signal for the carbon attached to the boron atom was not observed.

$^{19}F\{^1H\}$ NMR (376 MHz, $CDCl_3$, 298 K) δ -73.8 (d, J = 12.4 Hz), -127.9 (ddd, J = 13.2, 9.3, 5.0 Hz).
HRMS (ESI-MS) m/z= 393.0561 $[C_{13}H_{15}BF_4O_5SNa]^+$ (Calculated 393.0574 for $[M+Na]^+$)

Synthesis and Characterization of PPh_3 σ-Aryl Complexes 2-R

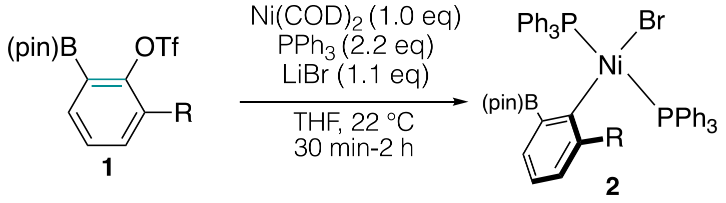

Scheme S2. Oxidative addition of **1** to furnish σ-aryl complex **2**.

General Procedure B: To a 20 mL scintillation vial charged with a stir bar was added boryl triflate aryne precursor **1-R** (0.550 mmol) along with LiBr (52.5 mg, 0.605 mmol, 1.1 equiv.), PPh_3 (317 mg, 1.21 mmol, 2.2 equiv.), and $Ni(COD)_2$ (151 mg, 0.550 mmol, 1.0 equiv.). THF (2.20 mL, 250 mM) was added, and the dark red mixture was allowed to stir at room temperature (see below for reaction times). The reaction was then concentrated under reduced pressure until less than approx. 0.5 mL solvent remained and the reaction appeared viscous. The remaining THF was azeotroped with pentane (3 x 2 mL) to encourage the product to powder out. The crude residue was then washed with 2 mL pentane followed by 8 mL diethyl ether and filtered over a celite plug to isolate the red impurities from the orange-brown solid. The solid was then recovered by dissolving in 10 mL THF and concentrating under reduced pressure, once again until the solution appeared viscous, and the remaining THF was azeotroped with pentane (2 x 2 mL) to powder out the σ-aryl complex. Single crystals were grown from a saturated toluene solution layered with pentane stored at -35 °C.

Note: Similarly to a previous report on related $(Cy_3P)_2Ni$ σ-aryl complexes, no clear resonances were observed in the ^{11}B NMR spectra of complexes **2-R**.[5]

2-H: *Stirred for 45 mins at rt. Obtained as a yellow solid (386 mg, 81% yield.) The chemical shifts of the ^1H spectrum were consistent with those reported in the literature.[6]

2-CN: Reaction was stirred for 2 h. Obtained as a yellow solid (366 mg, 75% yield.)
^1H NMR (400 MHz, C_6D_6, 298 K) δ 9.00-7.58 (br, 12H), 7.33 (dd, J = 7.4, 1.7 Hz, 1H), 7.27-6.60 (br, 18H) 6.32 (dd, J = 7.6, 1.7 Hz, 1H), 6.05 (t, J = 7.5 Hz, 1H), 1.08 (s, 12H).
$^{13}C\{^1H\}$ NMR (101 MHz, C_6D_6, 298 K) δ 177.1 (t, J = 32.7 Hz), 138.5 (d, J = 2.4 Hz), 137.0-134.6 (br), 134.4 (d, J = 2.6 Hz), 132.6 (t, J = 21.8 Hz), 130.4-129.0 (br), 129.3, 125.7, 123.1, 122.2 (t, J = 4.0 Hz), 121.0 (d, J = 2.3 Hz), 84.1, 24.7.
$^{31}P\{^1H\}$ NMR (162 MHz, C_6D_6, 298 K) δ 19.30.
HRMS (ESI-MS) m/z= 548.1461 $[C_{31}H_{30}BNNiO_2P]^+$ (Calculated 548.1459 for $[M-Br-PPh_3]^+$)

2-Me: Reaction was stirred for 1.5 h. Obtained as a yellow-brown solid (344 mg, 71% yield.)
^{1}H NMR (400 MHz, C_6D_6, 298 K) δ 8.70-7.29 (br, 12H), 7.26 (dd, *J* = 7.3, 1.6 Hz, 1H), 7.09-6.86 (br, 18H), 6.50 (tt, *J* = 7.2, 1.3 Hz, 1H), 6.25 (dd, *J* = 7.4, 1.7 Hz, 1H), 3.07 (s, 3H), 1.17 (s, 12H).
^{13}C{^{1}H} NMR (126 MHz, C_6D_6, 298 K) δ 163.5 (t, *J* = 32.4 Hz), 142.9, 137.0, 136.0-135.4 (br), 135.3 (d, *J* = 5.4 Hz), 133.4 (t, *J* = 20.4 Hz), 132.8 (t, *J* = 21.4 Hz), 130.0-129.3 (br), 129.7, 129.1 (t, *J* = 2.9 Hz), 121.7 (t, *J* = 2.3 Hz), 83.4, 25.3.
^{31}P{^{1}H} NMR (162 MHz, C_6D_6, 298 K) δ 17.3.
HRMS (ESI-MS) m/z= 537.1670 $[C_{31}H_{33}BNiO_2P]^+$ (Calculated 537.1669 for $[M\text{-}Br\text{-}PPh_3]^+$)

2-Cl: Reaction was stirred for 30 mins. Obtained as a yellow solid (382 mg, 77% yield.)
^{1}H NMR (400 MHz, C_6D_6, 298 K) δ 9.51-7.61 (br, 12H), 7.18 (dd, *J* = 7.2, 1.5 Hz, 1H), 7.14-6.41 (br, 18H), 6.31 (dd, *J* = 7.8, 1.5 Hz, 1H), 6.24 – 6.15 (m, 1H), 1.15 (s, 12H).
^{13}C{^{1}H} NMR (101 MHz, C_6D_6, 298 K) δ 165.1 (t, *J* = 33.2 Hz), 141.1, 137.9-133.9 (br), 135.4, 133.3, 133.1, 130.3-129.1 (br), 129.0 (t, *J* = 2.8 Hz), 123.0, 83.8, 25.0.
^{31}P{^{1}H} NMR (162 MHz, C_6D_6, 298 K) δ 18.7.
HRMS (ESI-MS) m/z= 557.11176 $[C_{30}H_{30}BClNiO_2P]^+$ (Calculated 557.1118 for $[M\text{-}Br\text{-}PPh_3]^+$)

2-F: Reaction was stirred for 2 h. Obtained as a yellow solid (320 mg, 72% yield.)
^{1}H NMR (400 MHz, C_6D_6, 298 K) δ 8.26-7.76 (br, 12H), 7.24 (d, *J* = 7.2 Hz, 1H), 7.13-6.89 (br, 18H), 6.28 (td, *J* = 7.5, 5.9 Hz, 1H), 5.85 (td, *J* = 7.8, 1.4 Hz, 1H), 1.24 (s, 12H).
^{13}C{^{1}H} NMR (101 MHz, C_6D_6, 298 K) δ 136.0-135.2 (br), 133.4, 133.2, 133.0, 129.7-129.3 (br), 123.6-123.3 (m), 115.1, 114.8, 83.9, 25.0.
^{19}F{^{1}H} NMR (376 MHz, C_6D_6, 298 K) δ -83.86.
^{31}P{^{1}H} NMR (162 MHz, C_6D_6, 298 K) δ 20.47 (d, *J* = 5.9 Hz).
HRMS (ESI-MS) m/z= 541.1414 $[C_{30}H_{30}BFNiO_2P]^+$ (Calculated 541.1412 for $[M\text{-}Br\text{-}PPh_3]^+$)

Synthesis and Characterization of CyPHOX–Ni σ-Aryl Complexes 3-R

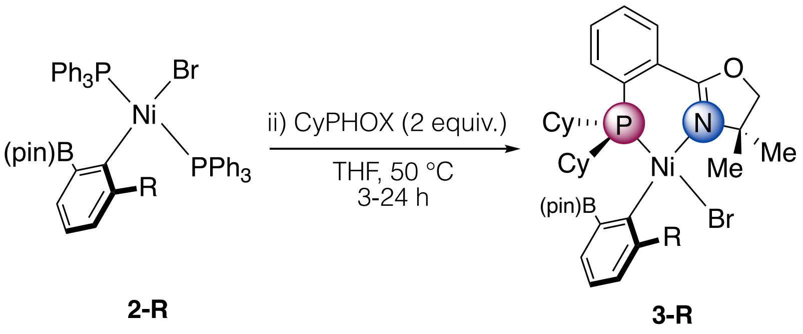

Scheme S3. Ligand exchange of PPh_3 σ-aryl complex **2-R** to furnish CyPHOX–Ni σ-aryl complex **3-R**.

General Procedure: To a 20 mL scintillation vial charged with a stir bar was added PPh_3 σ-aryl complex **2-R** (0.15 mmol), CyPHOX (111 mg, 0.3 mmol, 2 equiv.), and THF (1.0 mL, 0.15 mM). The suspension was heated on a hot plate to 50 °C with stirring. Afterwards, 2 mL pentane was added to the reaction to encourage precipitation of the product, and it was concentrated under reduced pressure. The crude residue was further azeotroped 2x with 1 mL pentane to remove residual THF. The crude residue was then washed with 5 mL pentane and filtered through a glass pipet plugged with celite and glass wool. The residue was then washed with a mixture of pentane and diethyl ether, leaving behind a solid. The remaining solids were recovered by redissolving in THF and once again azeotroping with pentane (3 x 1 mL) to give the desired CyPHOX–Ni σ-aryl complex **3-R**.

Note: Similarly to a previous report on related $(Cy_3P)_2Ni$ σ-aryl complexes, no clear resonances were observed in the ^{11}B NMR spectra of complexes **3-R**.[5]

***3-H** was not isolable and thus a combined ligand exchange and *in-situ* transmetalation was used to access **4-H** directly (See Synthesis and Characterization of **4-H** *vide infra*).

3-CN: Reaction was heated for 6 h, and the crude residue was washed with 1:1 pentane:diethyl ether. Isolated as a yellow solid (77.5 mg, 70% yield).
^{1}H NMR (400 MHz, C_6D_6, 298 K) δ 7.71 (dd, J = 7.2, 1.7 Hz, 1H), 7.67 – 7.62 (m, 1H), 7.24 – 7.16 (m, 1H), 7.08 (dd, J = 7.7, 1.6 Hz, 1H), 7.04 – 6.92 (m, 2H), 6.47 (t, J = 7.4 Hz, 1H), 3.56 (d, J = 8.1 Hz, 1H), 3.39 (d, J = 8.2 Hz, 1H), 2.86-0.58 (m, 22H) 2.23 (s, 3H), 1.82 (s, 3H), 1.26 (s, 6H), 0.98 (s, 6H).

$^{13}C\{^1H\}$ NMR (101 MHz, C_6D_6, 298 K) δ 163.7, 134.7, 132.5, 130.9, 130.3, 130.2, 129.5, 123.5, 122.5, 121.5, 83.9, 82.0, 72.8, 38.5, 30.6, 29.3, 29.2, 28.3, 28.1, 27.9, 27.8, 27.7, 26.6, 26.3, 26.2, 25.6, 25.5, 24.8. *Note: Aromatic signals missing due to overlap with C_6D_6 resonance
$^{31}P\{^1H\}$ NMR (162 MHz, C_6D_6, 298 K) δ 14.2.
HRMS (ESI-MS) m/z 657.2914 $[C_{36}H_{49}BN_2NiO_3P]^+$ (Calculated 657.2927 for $[M\text{-}Br]^+$)

3-Me: Reaction was heated for 3 h, and the crude residue was washed with 8:1 pentane:diethyl ether. Isolated as an orange-brown solid (92.7 mg, 85% yield).
^1H NMR (400 MHz, THF-*d8*, 298 K) δ 7.96 – 7.86 (m, 1H), 7.56 (ddd, *J* = 5.0, 3.1, 0.9 Hz, 2H), 7.50 – 7.40 (m, 1H), 7.02 (dd, *J* = 7.2, 1.7 Hz, 1H), 6.57 (dd, *J* = 7.3, 1.7 Hz, 1H), 6.45 (td, *J* = 7.2, 1.2 Hz, 1H), 4.23 (d, *J* = 8.2 Hz, 1H), 3.83 (d, *J* = 8.2 Hz, 1H), 3.07 (s, 3H), 2.19-0.56 (22 H), 2.05 (s, 3H), 1.78 (s, 3H), 1.19 (s, 6H), 0.93 (s, 6H)
$^{13}C\{^1H\}$ NMR (101 MHz, THF-*d8*, 298 K) δ 164.4 (d, *J* = 4.4 Hz), 143.5, 133.5, 132.4, 132.3, 131.8, 131.7 (d, *J* = 4.2 Hz), 131.0 (d, *J* = 6.4 Hz), 130.5, 130.3, 129.9 (d, *J* = 1.8 Hz), 128.2 (d, *J* = 2.9 Hz), 121.0 (d, *J* = 2.1 Hz), 83.5, 82.5, 72.9, 29.3, 28.9, 28.7, 28.5, 28.5, 28.3, 28.2, 27.9, 27.8, 26.9, 26.7, 26.6, 26.2.
$^{31}P\{^1H\}$ NMR (162 MHz, THF-*d8*, 298 K) δ 14.9.
HRMS (ESI-MS) m/z = 646.3115 $[C_{36}H_{52}BNNiO_3P]^+$ (Calculated 646.3131 for $[M\text{-}Br]^+$)

3-Cl: Reaction was heated for 24 h, and the crude residue was washed with 8:1 pentane:diethyl ether. Isolated as an orange solid (97.6 mg, 87% yield).
^1H NMR (400 MHz, C_6D_6, 298 K) 7.70 (dd, *J* = 7.0, 1.5 Hz, 1H), 7.68 – 7.62 (m, 1H), 7.24 (d, *J* = 4.5 Hz, 1H), 7.10 – 7.03 (m, 1H), 7.05 – 6.93 (m, 2H), 6.65 (t, *J* = 7.4 Hz, 1H), 3.59 (m, 1H) 3.38 (d, *J* = 8.1 Hz, 1H), 2.63-0.63 (m, 22 H), 2.15 (s, 3H), 1.88 (s, 3H), 1.29 (s, 6H), 1.01 (s, 6H).
$^{13}C\{^1H\}$ NMR (101 MHz, C_6D_6, 298 K) δ 163.6, 154.0, 148.7, 142.6, 137.7, 135.7, 132.2, 130.9, 130.2, 130.1, 129.3, 123.3, 111.4, 83.6, 82.0, 72.7, 30.3, 29.8, 29.2, 28.4, 28.1, 28.0, 27.9, 26.7, 26.4, 26.2, 25.8, 24.9.
$^{31}P\{^1H\}$ NMR (162 MHz, C_6D_6, 298 K) δ 14.1.
HRMS (ESI-MS) m/z 666.2571 $[C_{36}H_{49}BClNNiO_3P]^+$ (Calculated 666.2585 for $[M\text{-}Br]^+$)

3-F: Reaction was heated for 18 h, and the crude residue was washed with 4:1 pentane:diethyl ether. Isolated as a yellow solid (96 mg, 71% yield).
^1H NMR (400 MHz, THF–*d8*, 298 K) δ 7.93 – 7.84 (m, 1H), 7.63 – 7.54 (m, 2H), 7.50 (dd, *J* = 5.9, 3.2 Hz, 1H), 7.03 (dd, *J* = 7.0, 1.4 Hz, 1H), 6.59 (ddd, *J* = 8.0, 7.0, 5.4 Hz, 1H), 6.40 (ddd, *J* = 8.1, 7.1, 1.4 Hz, 1H), 4.30 (d, *J* = 8.2 Hz, 1H), 3.88 (d, *J* = 8.3 Hz, 1H), 2.25-0.44 (m, 22H), 2.07 (s, 3H), 1.77 (s, 3H), 1.19 (s, 6H), 0.94 (s, 6H).
$^{13}C\{^1H\}$ NMR (101 MHz, THF–*d8*, 298 K) δ 163.7, 131.3, 131.2, 129.9, 129.7, 129.3, 128.8, 128.1, 127.9, 121.9, 121.8 (d, *J* = 6.4 Hz), 113.6, 113.3, 82.9, 82.2, 72.1, 40.6, 31.7, 31.7, 28.4, 28.2, 27.7, 27.6, 27.2, 26.8, 26.1, 25.9.
$^{19}F\{^1H\}$ NMR (376 MHz, THF–*d8*, 298 K) δ -89.2.
$^{31}P\{^1H\}$ NMR (162 MHz, THF–*d8*, 298 K) δ 18.7.
HRMS (ESI-MS) m/z 650.2857 $[C_{35}H_{49}BFNNiO_3P]^+$ (Calculated 650.2881 for $[M\text{-}Br]^+$)

Synthesis and Characterization of CyPHOX–Ni Aryne Complexes 4-R

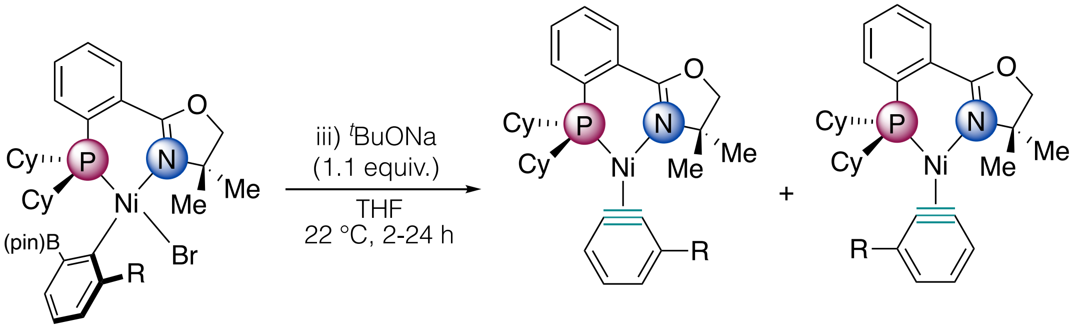

Scheme S4: Transmetalation of CyPHOX σ-aryl complex **3-R** to furnish CyPHOX–Ni aryne complexes **4-R**.

General Procedure: To a 20 mL scintillation vial charged with a stir bar was added CyPHOX σ-aryl complex **3-R** (0.1 mmol), sodium *tert*-butoxide (10.6 mg, 0.11 mmol, 1.1 equiv.), and THF (1.0 mL, 100 mM). The reaction was stirred at room temperature for a variable amount of time (see below), during which the color changed from orange-brown to fuchsia-red. Afterwards, 2 mL pentane was added to promote precipitation of the product, and the reaction was concentrated under reduced pressure. The crude residue was then azeotroped with 2 x 1 mL pentane to remove residual THF to give an oily red-purple solid. The product was then filtered through a glass pipet plugged with celite and glass wool using pentane and isolated as detailed for each compound (see below).

4-H: Prepared on 0.167 mmol scale *via* an *in-situ* ligand exchange-transmetalation sequence, run and worked up in the same manner as previously reported for the synthesis of **4-OMe**, except the ligand exchange step was run at 22 °C for 2 h.[7] Isolated as a dark fuchsia-red solid (76.9 mg, 91% yield.)
^{1}H NMR (400 MHz, THF–d_8, 298 K) δ 8.22 (ddd, *J* = 7.9, 3.4, 1.5 Hz, 1H), 7.97 – 7.89 (m, 1H), 7.68 – 7.47 (m, 2H), 7.32 (d, *J* = 5.7 Hz, 1H), 7.21 (d, *J* = 6.0 Hz, 1H), 6.79 – 6.62 (m, 2H), 4.31 (s, 2H), 2.40 – 2.25 (m, 2H), 2.18 (d, *J* = 13.1 Hz, 2H), 1.88-1.04 (18H), 1.79 (s, 6H).
^{13}C{^{1}H} NMR (101 MHz, THF–d_8, 298 K) δ 162.6 (d, *J* = 5.7 Hz), 144.4, 143.7, 137.3 (d, *J* = 8.5 Hz), 133.4 (d, *J* = 14.5 Hz), 132.9, 131.8 (d, *J* = 6.7 Hz), 131.6 – 131.1 (m), 130.4, 126.8, 125.9 (d, *J* = 6.6 Hz), 125.3 (d, *J* = 6.7 Hz), 121.4 (d, *J* = 7.6 Hz), 78.9, 72.2 (d, *J* = 3.8 Hz), 36.5, 36.3, 29.5, 29.4, 29.2, 29.1, 28.2, 27.8, 27.7, 27.6, 26.8.
^{31}P{^{1}H} NMR (162 MHz, THF–d_8, 298 K) δ 38.3.

HRMS (ESI-MS) m/z 506.2110 [$C_{29}H_{38}NNiOP$] (Calculated 506.2116 for $[M+H]^+$)

4-CN: Prepared *via* the General Procedure above. The reaction was stirred for 18 h before work up. The solid was washed with 5 mL pentane and was then redissolved and passed through the celite plug using THF. The orange solution was concentrated under reduced pressure, and azeotroped 3 x 1 mL pentane to give an orange solid (51.5 mg, >95 % yield).

^{1}H NMR (400 MHz, THF-d_8, 298 K) δ 8.29 – 8.23 (m, 1H), 7.97 (t, *J* = 6.9 Hz, 1H), 7.79-7.50 (m, 2H), 7.30 (d, *J* = 6.2 Hz, 1H), 7.02 – 6.91 (m, 1H), 6.74 (q, *J* = 6.8 Hz, 1H), 4.34 (s, 2H), 2.70-0.58 (m, 22H), 1.86 (s, 6H). Minor regioisomer: 8.05 (t, *J* = 6.9 Hz, 1H), 7.37 (s, 1H), 4.34 (s, 2H), 2.70-0.58 (m, 28H).

^{13}C{^{1}H} NMR (101 MHz, THF–d_8, 298 K) δ 163.4 (d, *J* = 6.0 Hz), 146.6, 145.8, 139.4, 139.3, 133.8, 133.1, 133.0, 132.2, 132.1, 131.8 (d, *J* = 3.7 Hz), 130.9, 128.9 (d, *J* = 4.9 Hz), 128.8 (d, *J* = 5.6 Hz), 126.7, 126.7, 124.9 (d, *J* = 6.7 Hz), 123.4, 122.8, 122.5, 105.3, 105.2, 79.4, 78.8, 72.2, 71.8, 71.7, 37.0, 36.8, 34.9, 29.69, 29.63, 29.28, 28.48, 27.69, 27.66, 27.62, 27.56, 27.52, 26.76.

^{31}P NMR (162 MHz, THF-d_8, 298 K) δ 38.5. Minor Regioisomer: 37.6. **r.r. 67:33.**

HRMS (ESI-MS) m/z= 531.2052 [$C_{30}H_{37}N_2NiOP$] (Calculated 531.2070 for $[M+H]^+$)

4-Me: Prepared *via* the General Procedure above. The reaction was stirred for 2 h before work up. The solid was washed with 5 mL pentane and was then redissolved and passed through the celite plug using Et_2O. The fuchsia solution was concentrated under reduced pressure, and azeotroped 3 x 1 mL pentane to give a bright red solid (52 mg, 92% yield.)

^{1}H NMR (400 MHz, THF–d_8, 298 K) δ 8.32-8.18 (m, 1H), 7.99-7.88 (m, 1H) 7.70-7.44 (m, 2H), 7.03 (d, *J* = 6.4 Hz, 1H), 6.80 – 6.28 (m, 2H), 4.30 (s, 2H), 2.57-0.83 (m, 22H), 2.48 (s, 3H), 1.89 (s, 6H). Minor Regioisomer: 8.07-7.99 (m, 1H), 7.17 (d, *J* = 4.6 Hz, 1H), 4.27 (s, 2H), 2.57-0.83 (m, 28H), 2.41 (s, 3H).

^{13}C{^{1}H} NMR (101 MHz, THF–d_8, 298 K) δ 162.4 (d, *J* = 5.8 Hz), 144.3, 143.6, 141.8, 141.0, 137.4 (d, *J* = 8.7 Hz), 135.2 (d, *J* = 7.3 Hz), 133.6 (d, *J* = 2.0 Hz), 133.5, 133.4 – 133.2 (m), 133.0 (d, *J* = 2.2 Hz), 132.2, 132.1, 131.8 (dd, *J* = 10.2, 6.8 Hz), 131.3 (d, *J* = 3.2 Hz), 131.2 (d, *J* = 2.9 Hz), 131.0 (d, *J* = 8.7 Hz), 130.5 (d, *J* = 1.7 Hz), 130.4 (d, *J* = 1.6 Hz), 128.3 (d, *J* = 2.1 Hz), 127.4 (d, *J* = 2.6 Hz), 126.9 (d, *J* = 6.0 Hz), 125.8 (d, *J* = 6.9 Hz), 123.2 (d, *J* = 7.2 Hz), 119.1 (d, *J* = 7.4 Hz), 79.1, 78.8, 72.6 (d, *J* = 4.0 Hz), 71.6 (d, *J* = 3.8 Hz), 38.3, 38.1, 37.4, 37.2, 34.9, 30.7, 30.6, 29.6, 29.5, 29.5, 29.3, 29.3, 29.3, 28.7, 28.3, 28.0, 27.9, 27.8, 27.8, 27.7, 27.7, 26.9, 26.9, 23.2, 23.2, 22.9.

^{31}P{^{1}H} NMR (162 MHz, THF–d_8, 298 K) δ 38.5. Minor Regioisomer: 37.9. **r.r. 73:27.**

HRMS (ESI-MS) m/z 520.2277 [$C_{30}H_{40}NNiOP$] (Calculated 520.2273 for $[M+H]^+$)

4-Cl: Prepared *via* the General Procedure above. The reaction was stirred for 24 h before work up. The solid was completely dissolved in pentane, passed through the celite plug, and the purple solution was concentrated under reduced pressure to give a bright red solid (48.7 mg, 90% yield).

^{1}**H NMR** (400 MHz, THF–d_8, 298 K) δ 8.26 (ddd, *J* = 8.0, 3.5, 1.5 Hz, 1H), 7.98 (ddd, *J* = 7.5, 5.8, 1.5 Hz, 1H), 7.81 – 7.45 (m, 2H), 7.03 (dq, *J* = 4.3, 2.4 Hz, 1H), 6.71 – 6.59 (m, 2H), 4.31 (s, 2H), 2.69-1.03 (m, 22H), 1.86 (s, 6H).
Minor Regioisomer: 8.04 (t, *J* = 6.9 Hz, 1H), 7.16 (dd, *J* = 5.7, 3.0 Hz, 1H), 4.10 (s, 2H), 2.69-1.03 (m, 28H)
^{13}**C{^{1}H} NMR** (101 MHz, THF–d_8, 298 K) δ 162.9 (d, *J* = 6.0 Hz), 143.9, 143.8, 138.0, 137.3, 133.7, 133.2, 133.1 (d, *J* = 1.8 Hz), 133.0, 133.0, 132.0, 132.0 (d, *J* = 6.8 Hz), 131.6 (d, *J* = 3.5 Hz), 131.1, 131.0, 130.8 (d, *J* = 1.8 Hz), 129.4, 129.4, 127.5, 127.3, 125.6, 125.6, 123.3, 123.3, 118.8 (d, *J* = 7.3 Hz), 79.4, 79.1, 78.8, 72.2, 71.8 (d, *J* = 3.1 Hz), 37.8, 37.6, 37.4, 37.1, 30.6, 30.6, 29.7, 29.7, 29.5, 29.3, 28.6, 28.1, 27.9, 27.8, 27.7, 27.7, 27.6, 27.6, 27.0, 26.8.
^{31}**P NMR** (162 MHz, THF–d_8, 298 K) δ 39.4. Minor Regioisomer: 36.6. **r.r. 86:14.**
HRMS (ESI-MS) m/z 540.1740 [$C_{29}H_{37}ClNNiOP$] (Calculated 540.1727 for $[M+H]^+$)

4-F: Prepared *via* the General Procedure above. The reaction was stirred for 6 h before work up. The solid was washed with 5 mL pentane and was then redissolved and passed through the celite plug using diethyl ether. The fuchsia solution was concentrated under reduced pressure, and azeotroped 3 x 1 mL pentane to give a bright red solid (47.5 mg, 91% yield).
^{1}**H NMR** (400 MHz, THF–d_8, 298 K) δ 8.24 (ddd, *J* = 8.0, 3.5, 1.5 Hz, 1H), 7.96 (ddd, *J* = 7.5, 5.8, 1.5 Hz, 1H), 7.68 – 7.51 (m, 2H), 6.98 (td, *J* = 6.6, 2.1 Hz, 1H), 6.75 (td, *J* = 6.5, 3.5 Hz, 1H), 6.32 (ddd, *J* = 8.4, 6.9, 3.9 Hz, 1H), 4.31 (s, 2H), 2.50-1.03 (m, 22H), 1.84 (s, 6H). Minor Regioisomer: δ 8.20 (d, *J* = 1.2 Hz, 1H), 7.14 (ddd, *J* = 7.3, 6.0, 3.1 Hz, 1H), 4.31 (s, 2H), 2.50-1.03 (m, 28H).
^{13}**C{^{1}H} NMR** (101 MHz, THF–d_8, 298 K) δ 163.0 (d, *J* = 5.8 Hz), 162.1 (d, *J* = 5.0 Hz), 159.6 (d, *J* = 4.9 Hz), 146.97-146.71 (m), 133.2, 133.1, 133.1, 133.0 (d, *J* = 1.7 Hz), 132.0 (d, *J* = 6.8 Hz), 131.6 (d, *J* = 3.5 Hz), 131.1, 130.9, 130.7 (d, *J* = 1.6 Hz), 129.6 (d, *J* = 2.4 Hz), 127.6 (d, *J* = 8.0 Hz), 122.8, 122.3, 122.1, 121.5, 121.0 (dd, *J* = 5.3, 1.9 Hz), 116.6 (d, *J* = 7.9 Hz), 113.0, 112.7, 111.3 (d, *J* = 3.6 Hz), 111.0 (d, *J* = 3.5 Hz), 79.2, 78.9, 72.2 (d, *J* = 3.6 Hz), 71.8 (d, *J* = 3.0 Hz), 37.0, 36.7, 36.5, 36.3, 29.7, 29.6, 29.3, 28.3, 28.0, 27.8, 27.7, 27.7, 27.6, 27.5, 26.8, 26.7.
^{19}**F{^{1}H} NMR** (376 MHz, THF–d_8, 298 K) δ -107.1 (d, *J* = 4.3 Hz).
Minor Regioisomer: δ -111.6 (d, *J* = 2.3 Hz).
^{31}**P{^{1}H} NMR** (162 MHz, THF–d_8, 298 K) δ 40.4 (d, *J* = 4.8 Hz).
Minor Regioisomer: δ 36.8. **r.r. 88:12.**
HRMS (ESI-MS) m/z 524.2028 [$C_{29}H_{37}FNNiOP$] (Calculated 524.2022 for $[M+H]^+$)

Synthesis and Characterization of Reduced Benzyne Complex 5-H

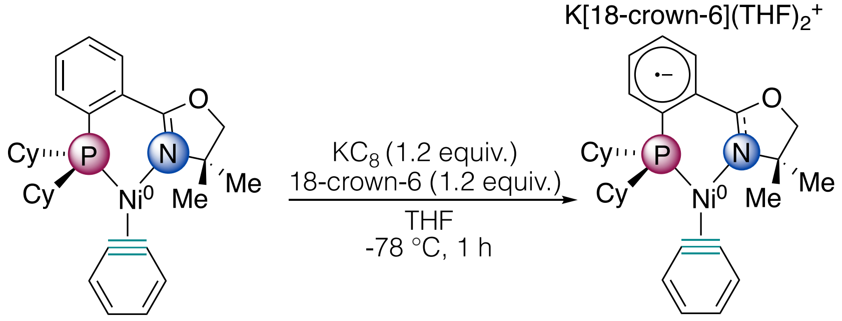

Scheme S5: Reduction of CyPHOX–Ni aryne complex **4-H** to anionic benzyne complex **5-H**.

Procedure: To a 20 mL scintillation vial charged with a glass-coated stir bar was added benzyne complex **4-H** (19.0 mg, 0.0375 mmol) and 3 mL THF (12.5 mM). The fuchsia solution was cooled to -78 °C and KC_8 (6.1 mg, 0.045 mmol, 1.2 equiv.) was added as a solid. The reaction mixture was allowed to stir and warm to 22 °C over the course of 1 h. A color change from fuchsia to a deep merlot was observed. The reaction mixture was filtered over glass filter paper to filter out the graphite and added into a 1 mL THF solution of 11.9 mg 18-crown-6 (1.2 equiv.). The volatiles were removed under reduced pressure and the resulting residue was triturated with pentane. The residue was washed with pentane and dried under vacuum to isolate the reduced benzyne complex **5-H** (24.9 mg, 82 % yield, average of two runs.) **Note:** Extended periods of time in solution resulted in decomposition, preventing the acquisition of a $^{13}C\{^1H\}$ spectrum.
^{1}H NMR (400 MHz, THF–*d8*, 298 K) δ 7.84 (d, *J* = 7.4 Hz, 1H), 7.66 (d, *J* = 6.8 Hz, 1H), 7.30 (d, *J* = 5.4 Hz, 1H), 7.04 (dt, *J* = 22.7, 6.7 Hz, 2H), 6.75 (t, *J* = 7.1 Hz, 2H), 6.60 (t, *J* = 6.8 Hz, 1H), 3.36-3.39 (br, 24H), 2.91 (d, *J* = 5.1 Hz, 2H), 2.06-0.94 (28H).
$^{31}P\{^1H\}$ NMR (162 MHz, THF–*d8*, 298 K) δ 69.1.

Synthesis of 6-R-ab/Difunctionalizations of Complexes 4-R

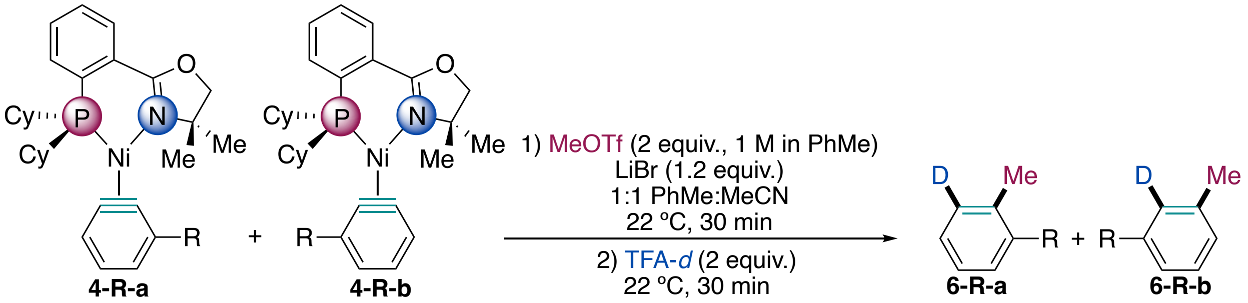

Scheme S6: Methylation/deuteration of CyPHOX σ-aryl complexes **4-R** to furnish regioisomeric arene products **6-R-ab**.

General Procedure: To a 20 mL scintillation vial charged with a stir bar was added CyPHOX–Ni aryne complex **4-R** (0.1 mmol), lithium bromide (10.4 mg, 0.12 mmol, 1.2 equiv.), toluene (0.3 mL), and acetonitrile (0.5 mL). Methyl triflate (1 M solution in toluene, 0.2 mL, 2 equiv.) was added with stirring. The reaction was stirred at room temperature for 30 mins, during which the color changed from red/fuchsia to orange. Afterwards, TFA-*d* (16.7 μL) was added with stirring. The reaction was allowed to stir for 30 mins during which it turned a lighter orange-yellow color. Afterwards, the reaction was opened to air, and filtered through a silica plug. The crude residue was then azeotroped with 2 x 1 mL pentane to remove residual THF to give an oily red-purple solid. The product was then filtered through a glass pipet plugged with silica and glass wool with an additional 0.4 mL 1:1 PhMe:MeCN. The filtrate was added to an NMR tube to which was also added 5 μL CD_2Cl_2 as an internal standard. **Note:** A PhMe:MeCN solvent combination was used due to the incompatibility of complexes **4-R** with chlorinated solvents, their limited solubility in MeCN, the polymerization of THF in the presence of MeOTf, and the use of a solution of MeOTf in PhMe.)

6-Me-ab: Synthesized *via* the General Procedure above. Product resonances overlapped in ^{2}H NMR spectrum but gave a total internal yield of 10.0% (1.07 mg, average of 2 runs). Both regioisomers were detected *via* GC/MS to give a 79:21 r.r.
^{2}H NMR (61 MHz, 1:1 PhMe:MeCN, 298 K) δ 7.52, 7.39.
MS (GC-MS/EI) m/z 107.0830 [C_8H_9D] (Calculated 107.0800 for [M])

6-Cl-ab: Synthesized *via* the General Procedure above. Only 1 regioisomer detected *via* ^{2}H NMR and GC/MS. Internal yield calculated by ^{2}H NMR to be 11.0% (1.40 mg **6-Cl-a**, average of 2 runs).
^{2}H NMR (61 MHz, 1:1 PhMe:MeCN, 298 K) δ 7.45.
HRMS (GC-MS/EI) m/z 112.0070 [C_6H_3DCl] (Calculated 112.0100 for [$M-CH_3$]$^+$)

6-F-a: Synthesized *via* the General Procedure above. Only 1 regioisomer detected *via* ^{2}H and $^{19}F\{^{1}H\}$ NMR. Internal yield calculated by ^{2}H NMR to be 10.0% (1.11 mg **6-F-a**, average of 2 runs).

Due to its volatility, the product coeluted with toluene solvent on GC/MS.

^{2}H NMR (61 MHz, 1:1 PhMe:MeCN, 298 K) δ 7.54 (d, J = 8.6 Hz).

^{19}F{^{1}H} NMR (376 MHz, 1:1 PhMe:MeCN, 298 K) δ -119.32 (d, J = 8.5 Hz).

MS (GC/MS) m/z = 110.0495 [C_7H_5DF] (calculated 111.0500 for $[M-H]^+$)

NMR Spectra of Compounds

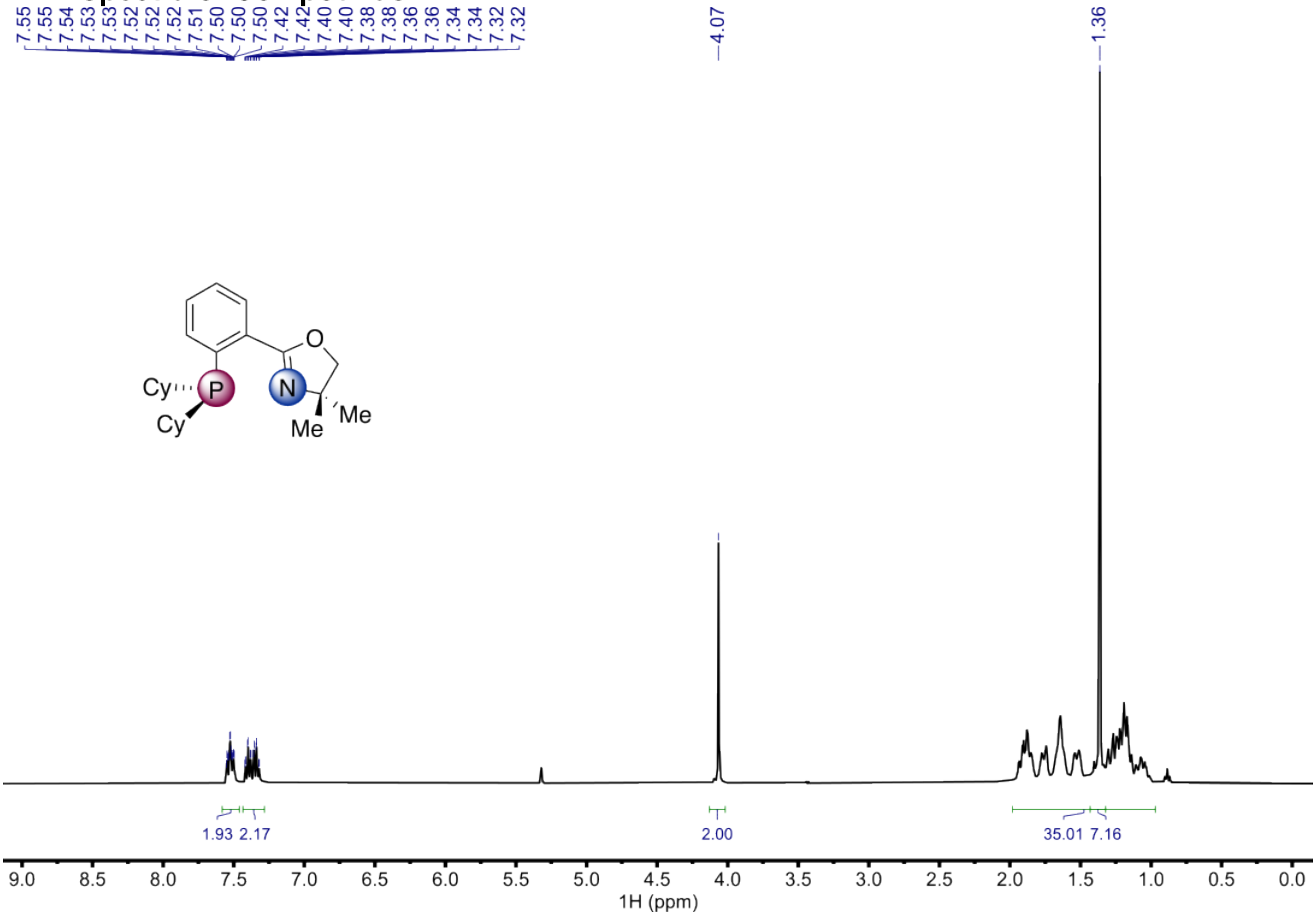

Figure S1. ^{1}H NMR spectrum (400 MHz, CD_2Cl_2, 298 K) of **CyPHOX.**

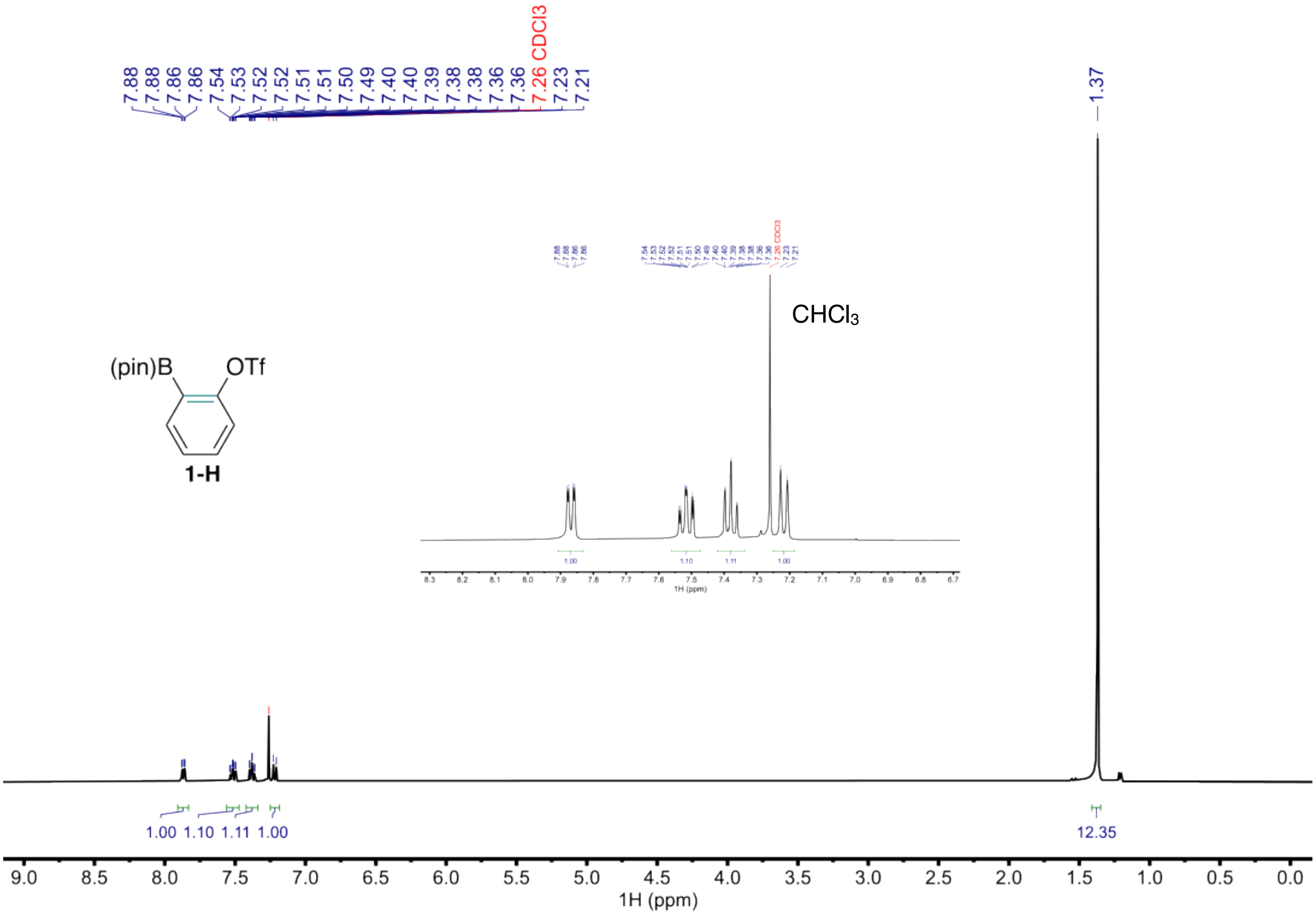

Figure S2. ^{1}H NMR spectrum (400 MHz, $CDCl_3$, 298 K) of **1-H.**

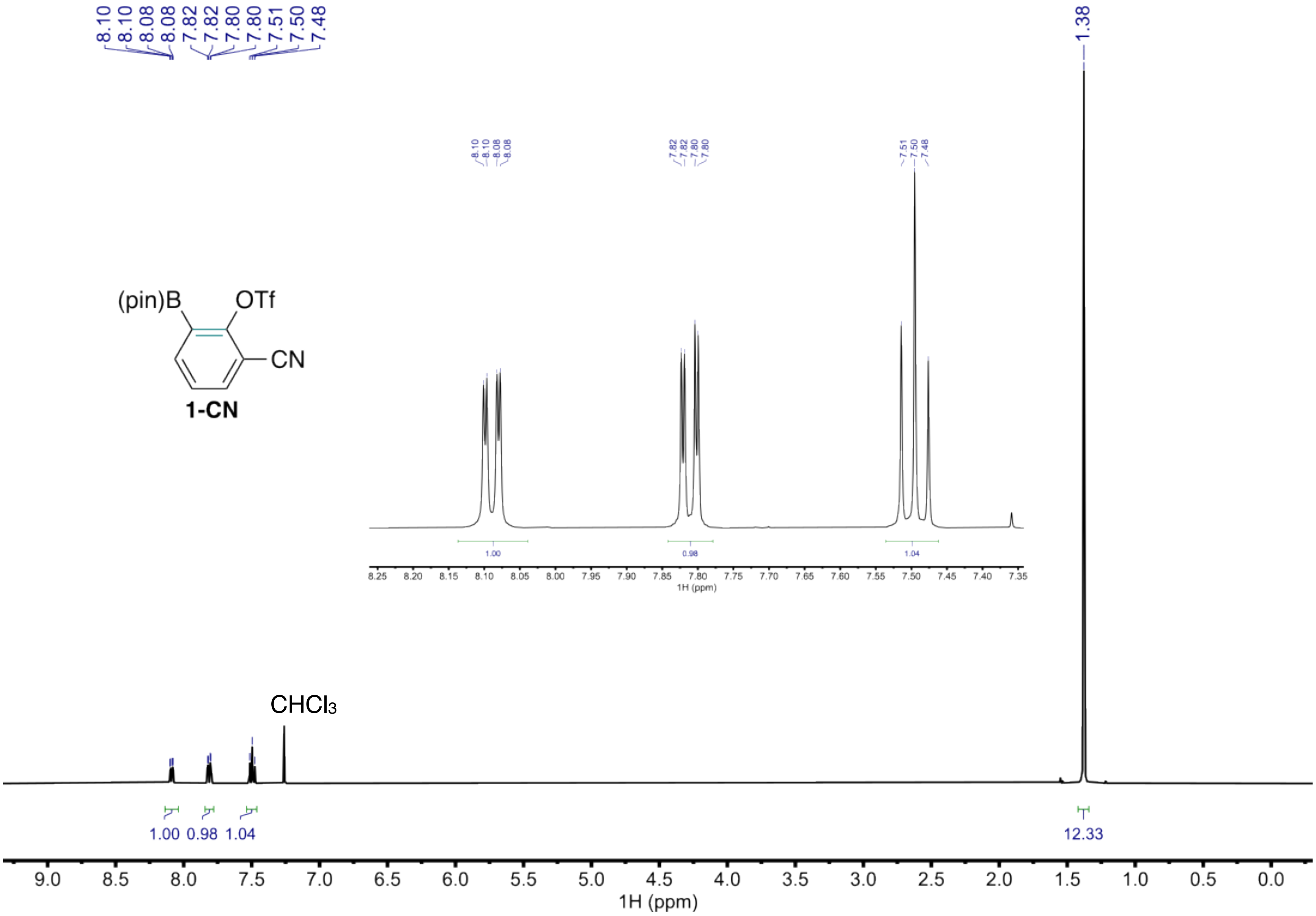

Figure S3. ^{1}H NMR spectrum (400 MHz, $CDCl_3$, 298 K) of **1-CN**.

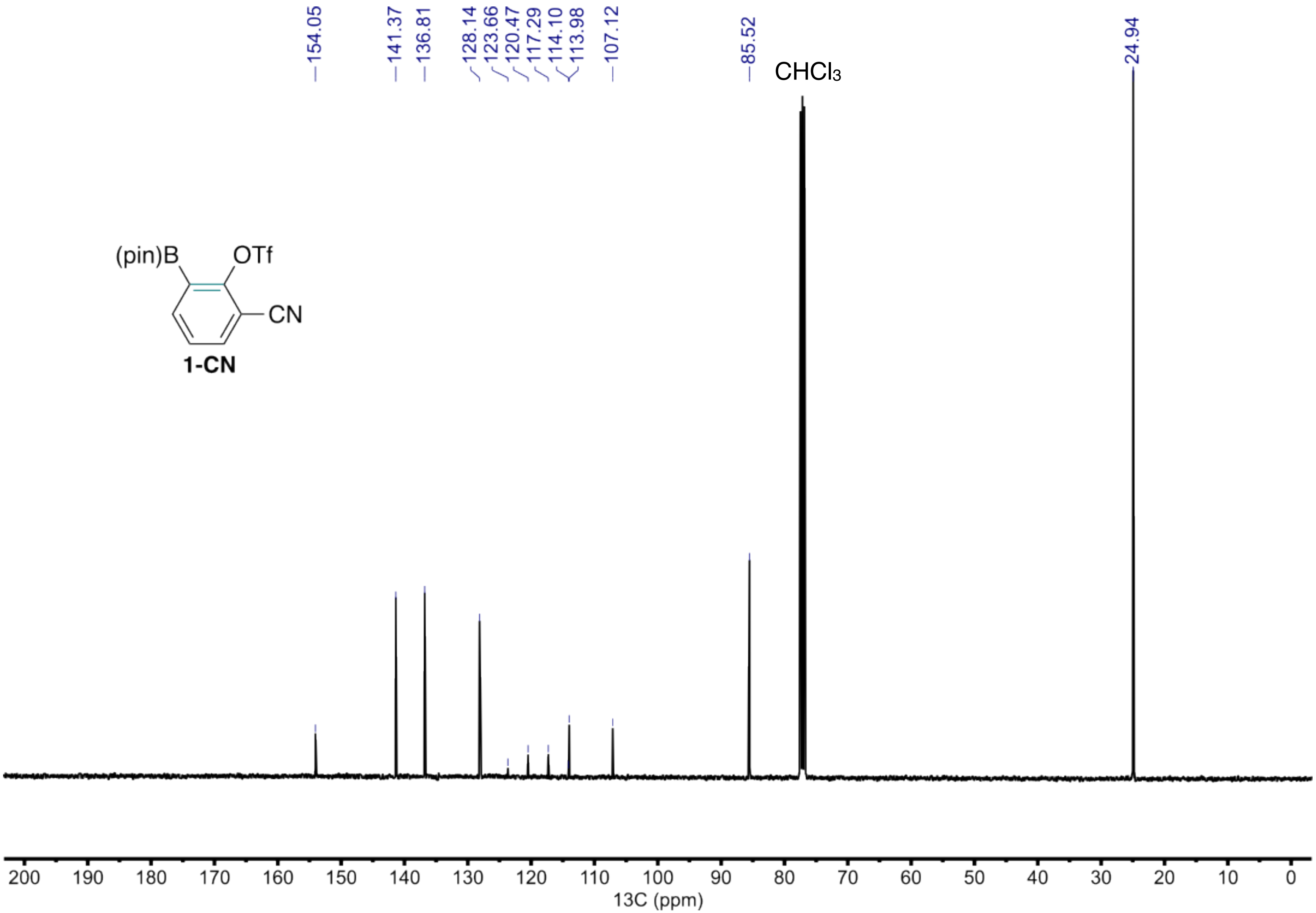

Figure S4. $^{13}C\{^1H\}$ NMR spectrum (101 MHz, $CDCl_3$, 298 K) of **1-CN**.

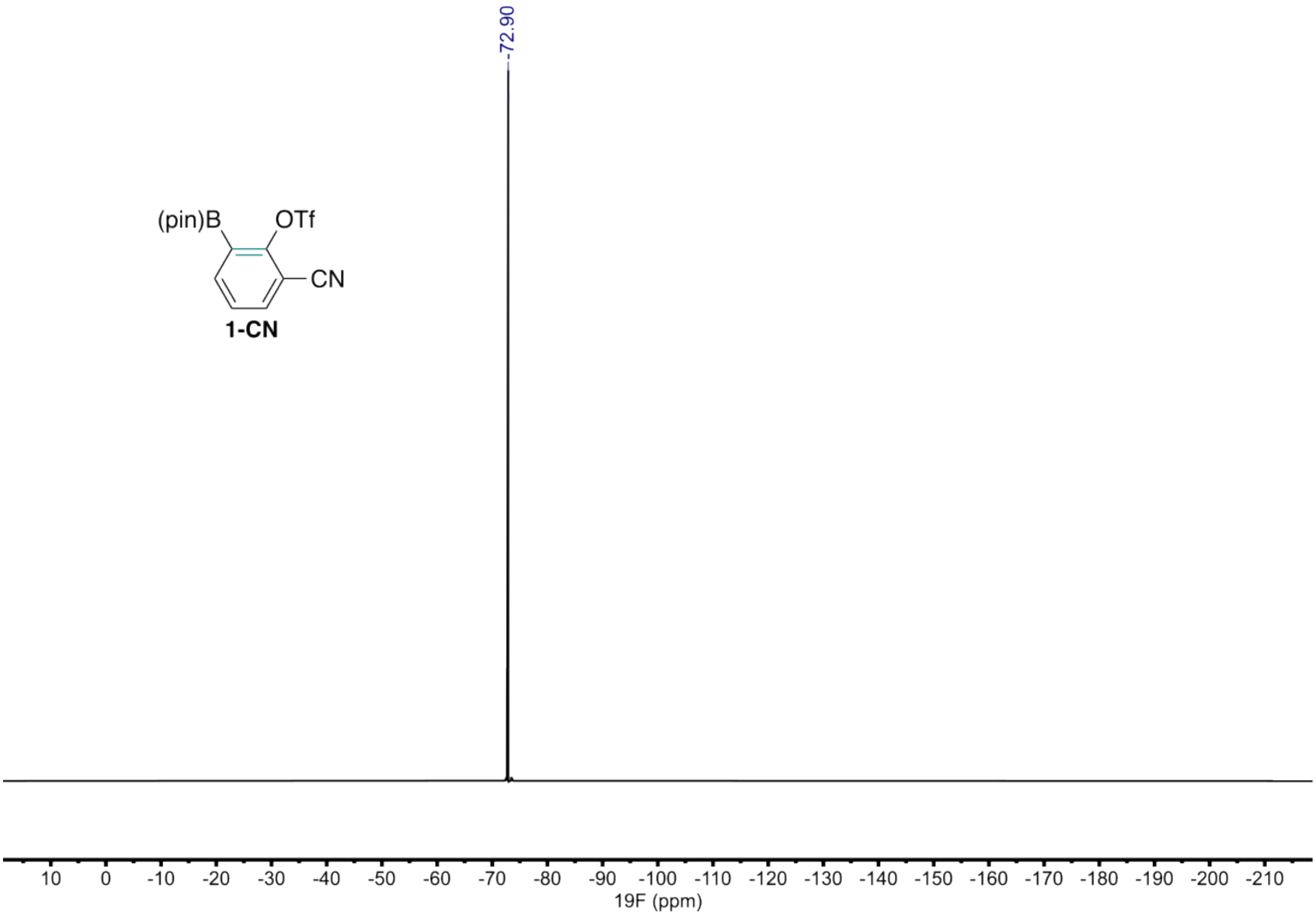

Figure S5. $^{19}F\{^{1}H\}$ NMR spectrum (376 MHz, $CDCl_3$, 298 K) of **1-CN**.

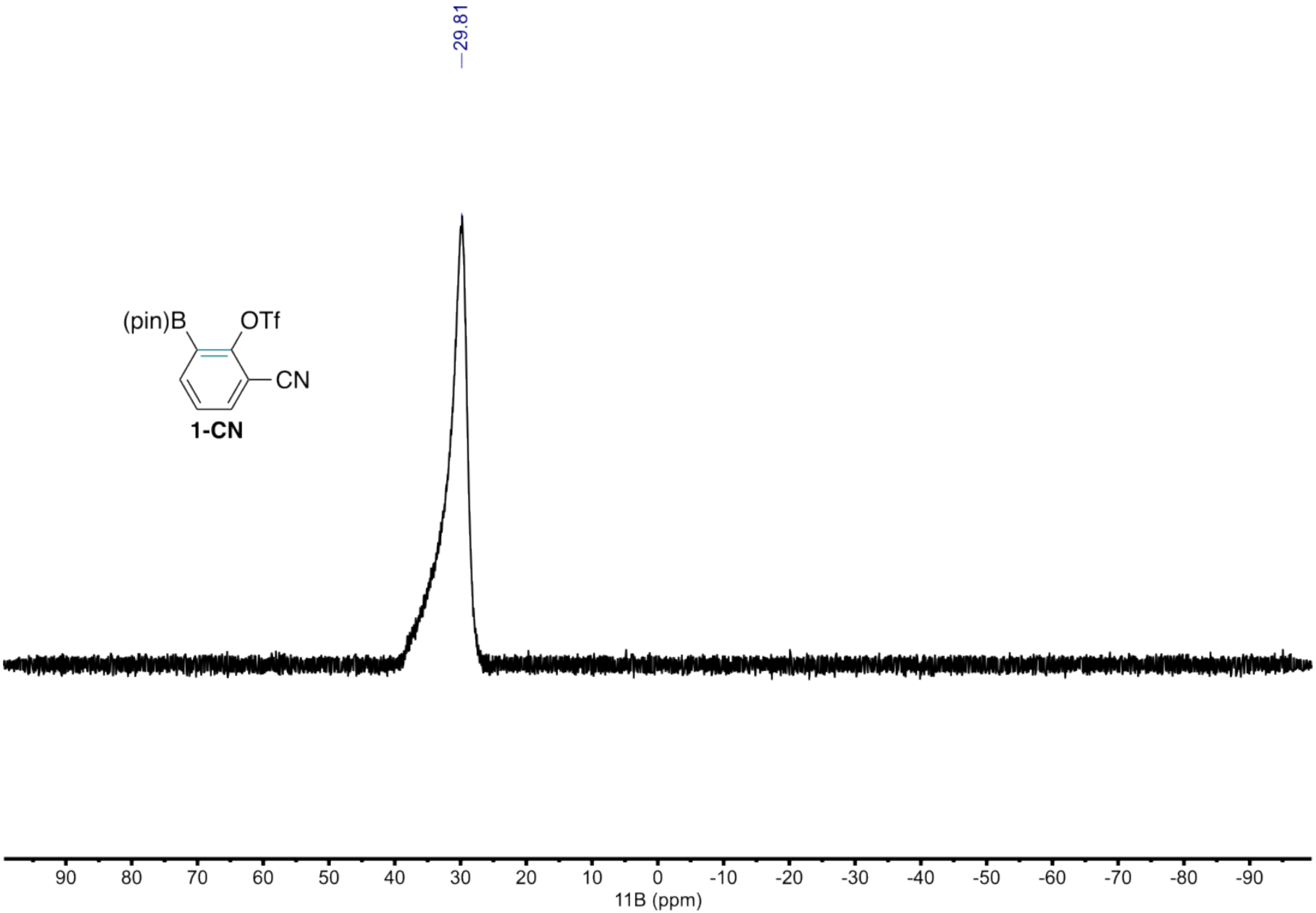

Figure S6. ^{11}B NMR spectrum (128 MHz, $CDCl_3$, 298 K) of **1-CN**. Whittaker Smoother baseline correction performed.

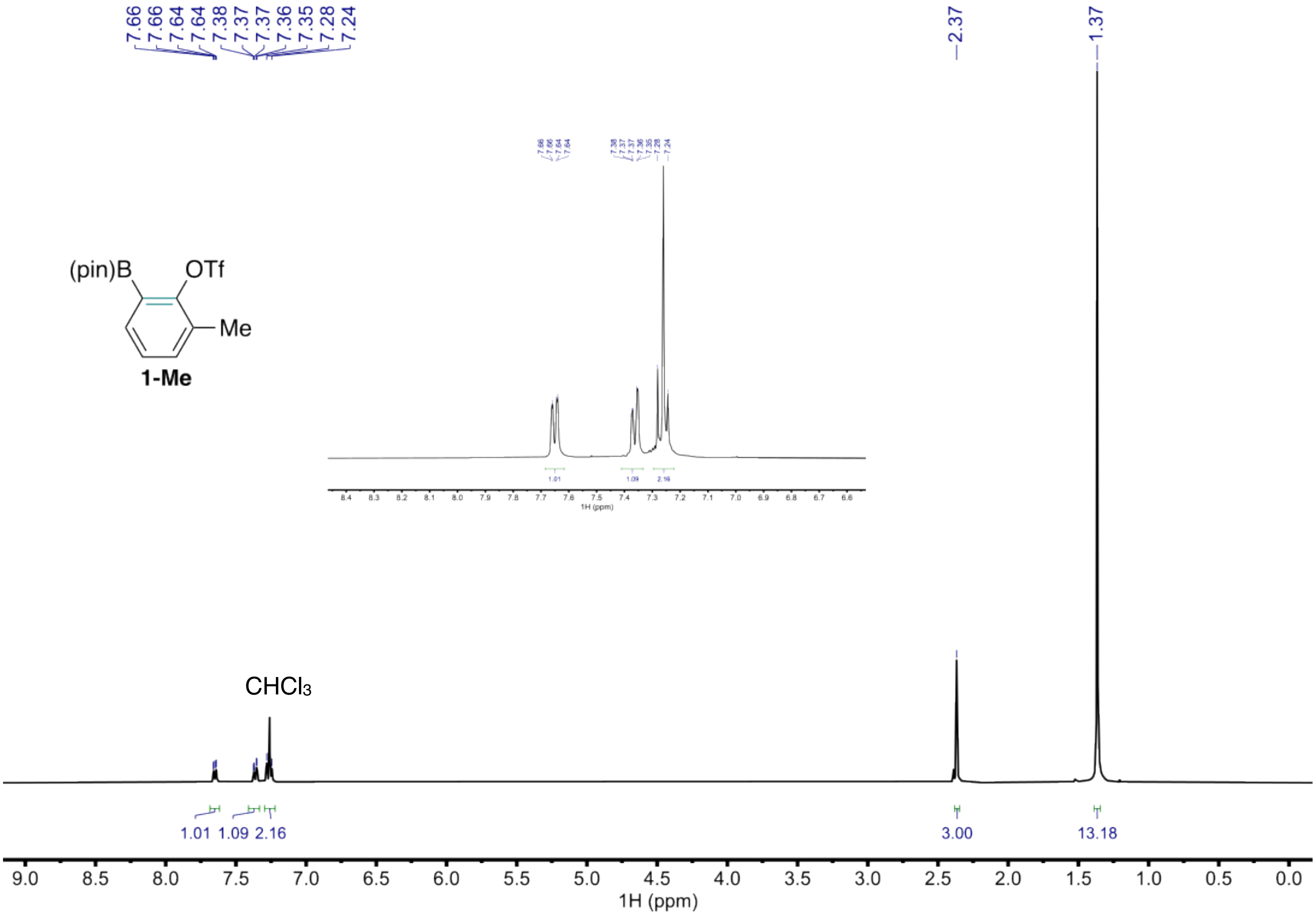

Figure S7. ^{1}H NMR spectrum (400 MHz, $CDCl_3$, 298 K) of **1-Me**.

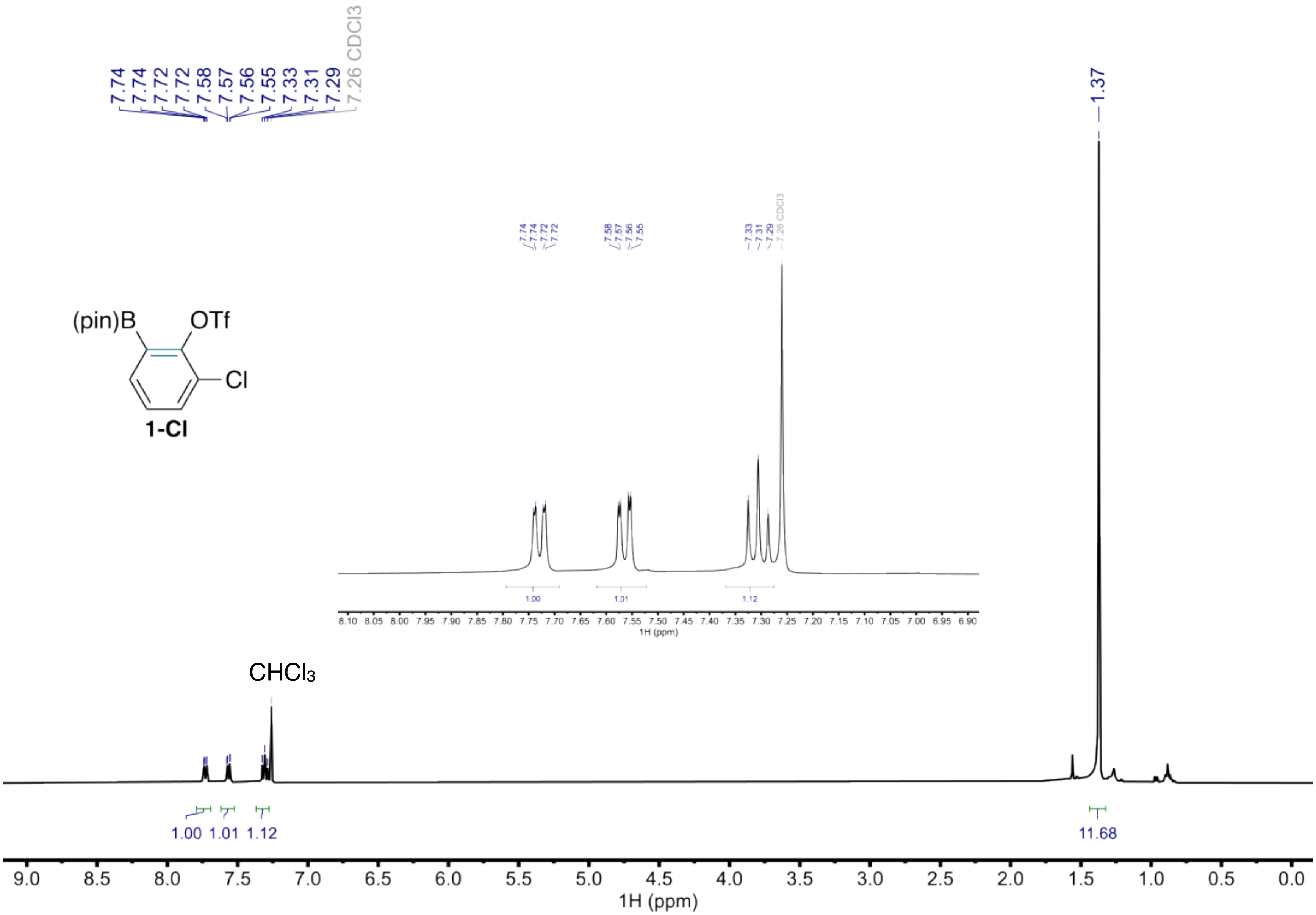

Figure S8. ^{1}H NMR spectrum (400 MHz, $CDCl_3$, 298 K) of **1-Cl**.

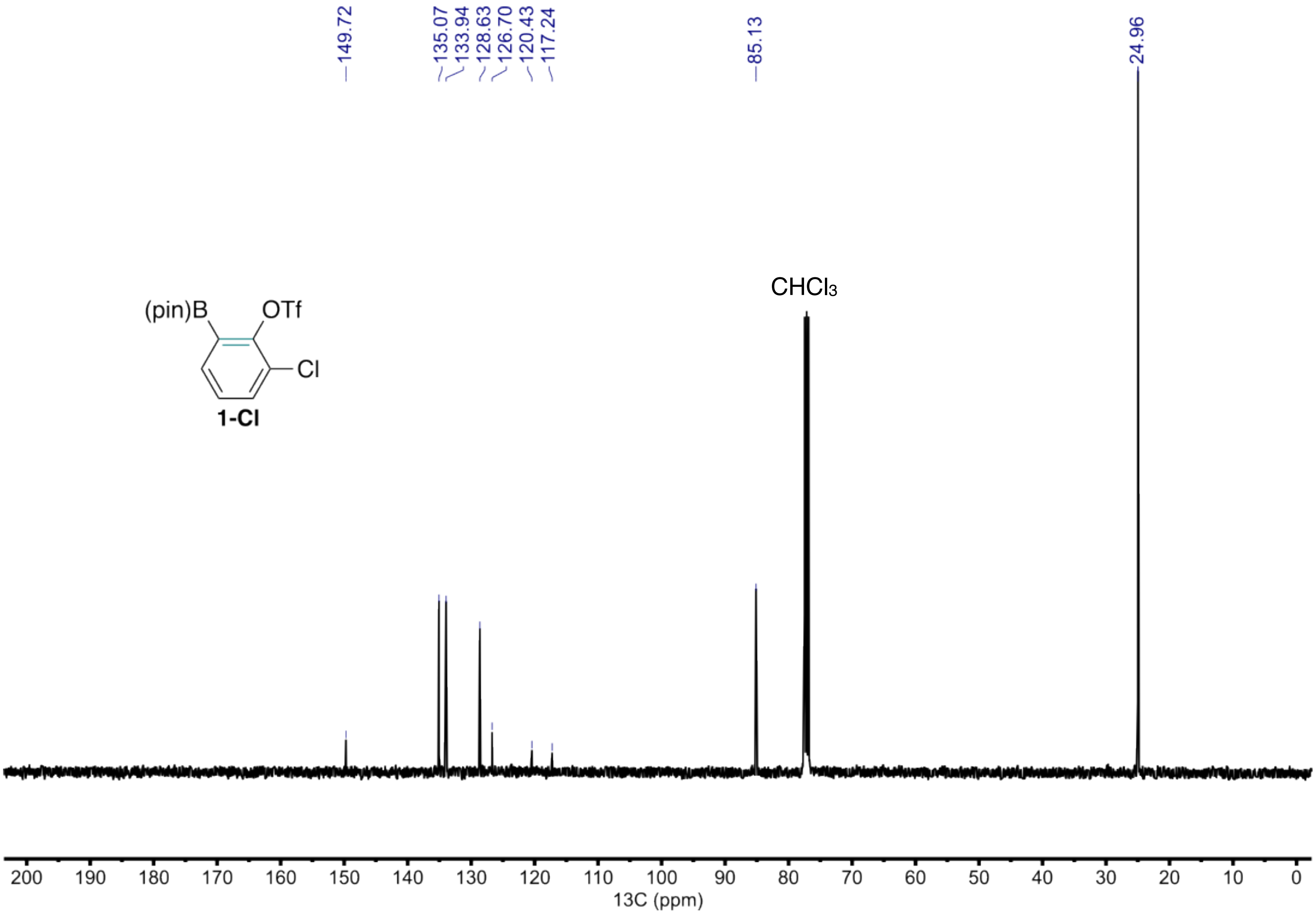

Figure S9. $^{13}C\{^1H\}$ NMR spectrum (101 MHz, $CDCl_3$, 298 K) of **1-Cl**.

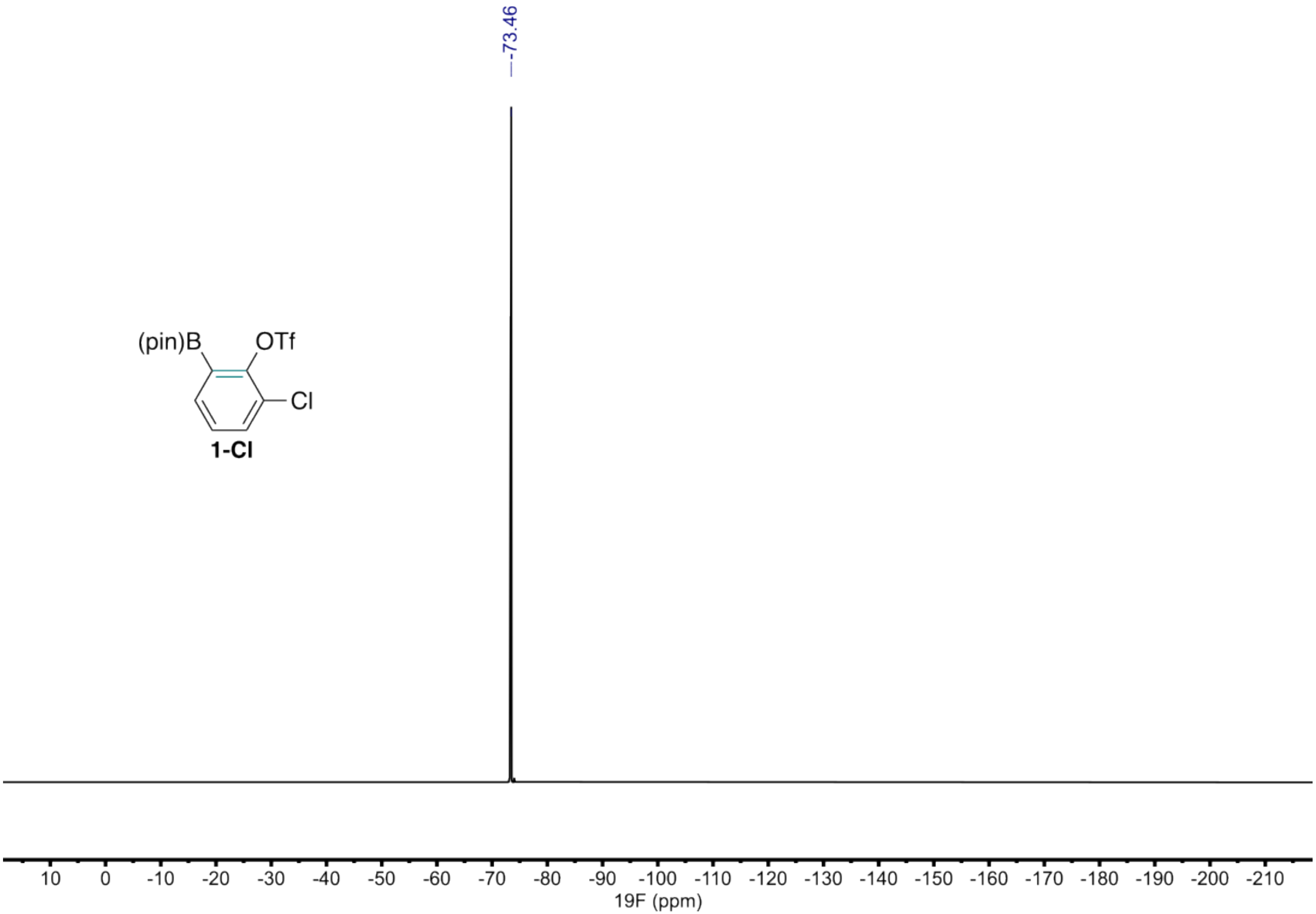

Figure S10. $^{19}F\{^{1}H\}$ NMR spectrum (376 MHz, $CDCl_3$, 298 K) of **1-Cl**.

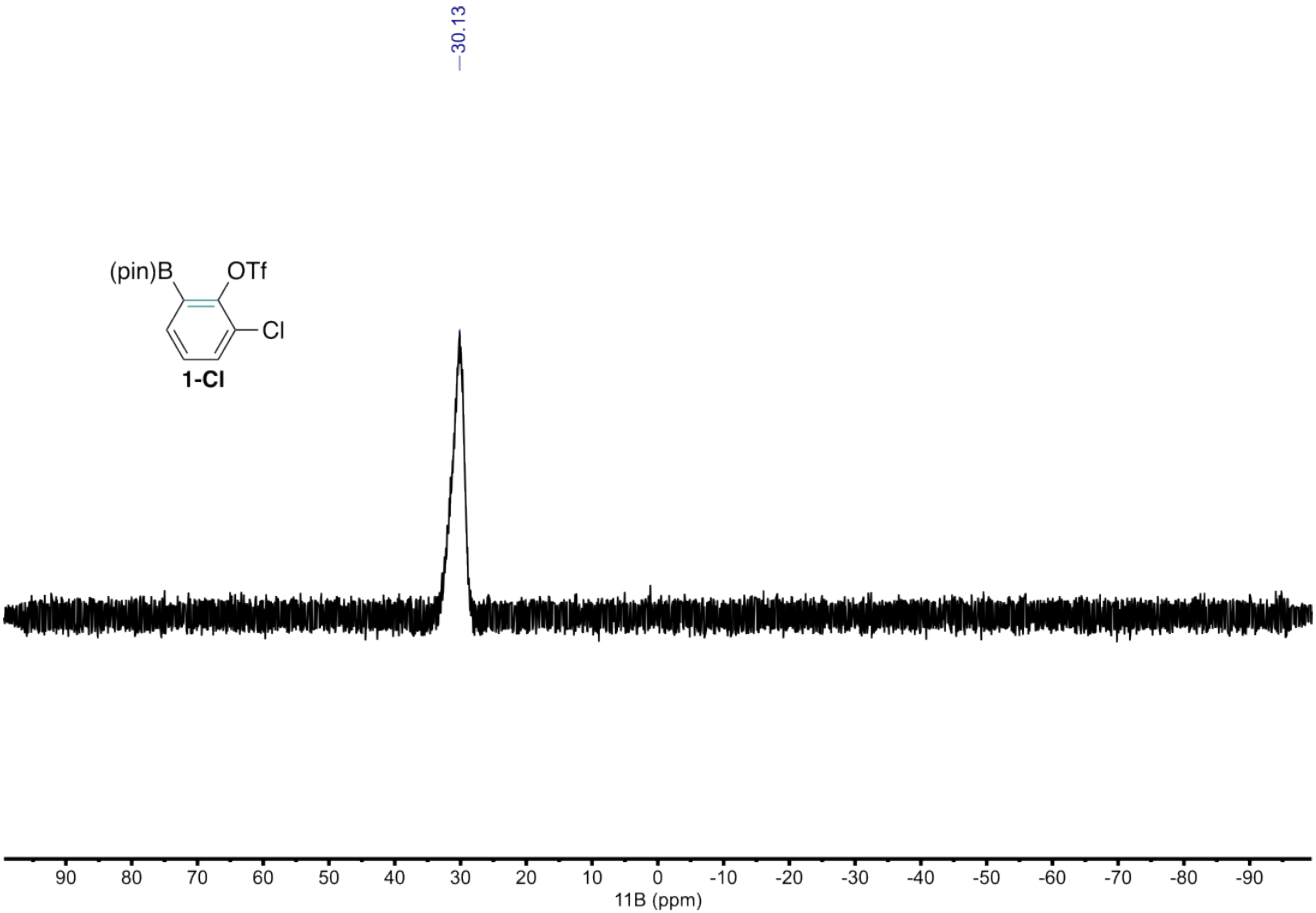

Figure S11. ^{11}B NMR spectrum (128 MHz, $CDCl_3$, 298 K) of **1-Cl**. Whittaker Smoother baseline correction performed.

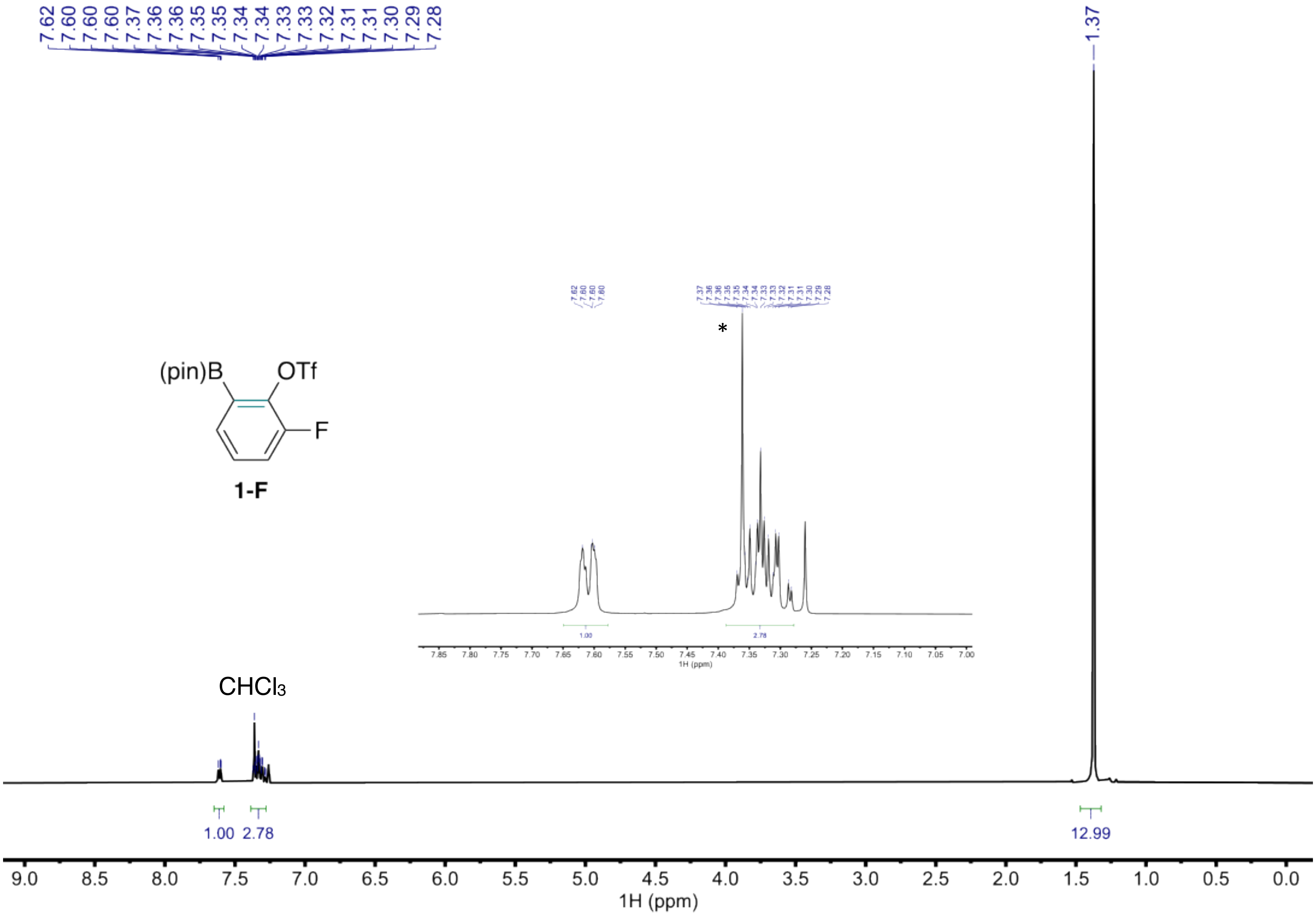

Figure S12. ^{1}H NMR spectrum (400 MHz, $CDCl_3$, 298 K) of **1-F.** *Residual benzene from azeotroping with benzene.

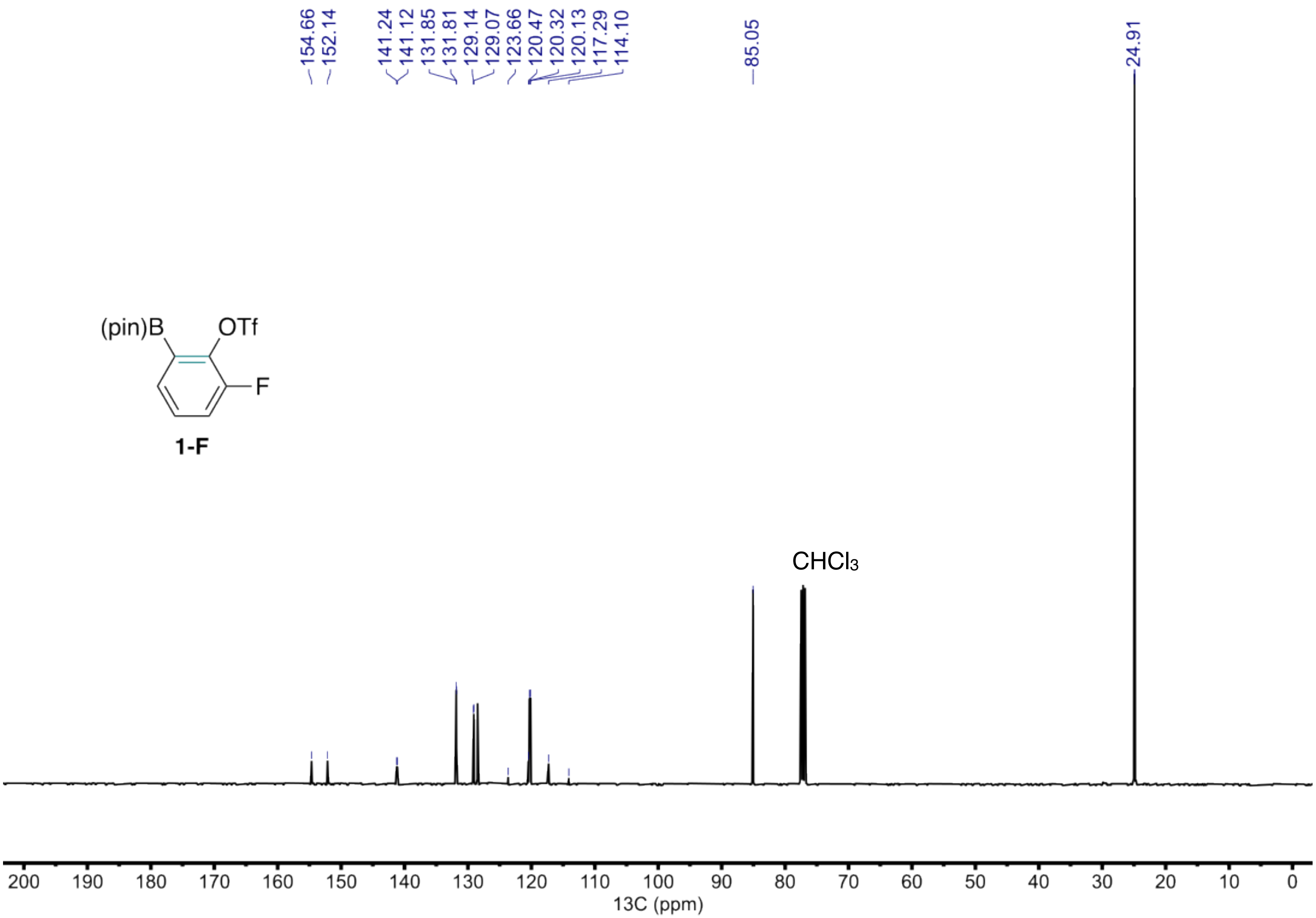

Figure S13. $^{13}C\{^{1}H\}$ NMR spectrum (101 MHz, $CDCl_3$, 298 K) of **1-F**.

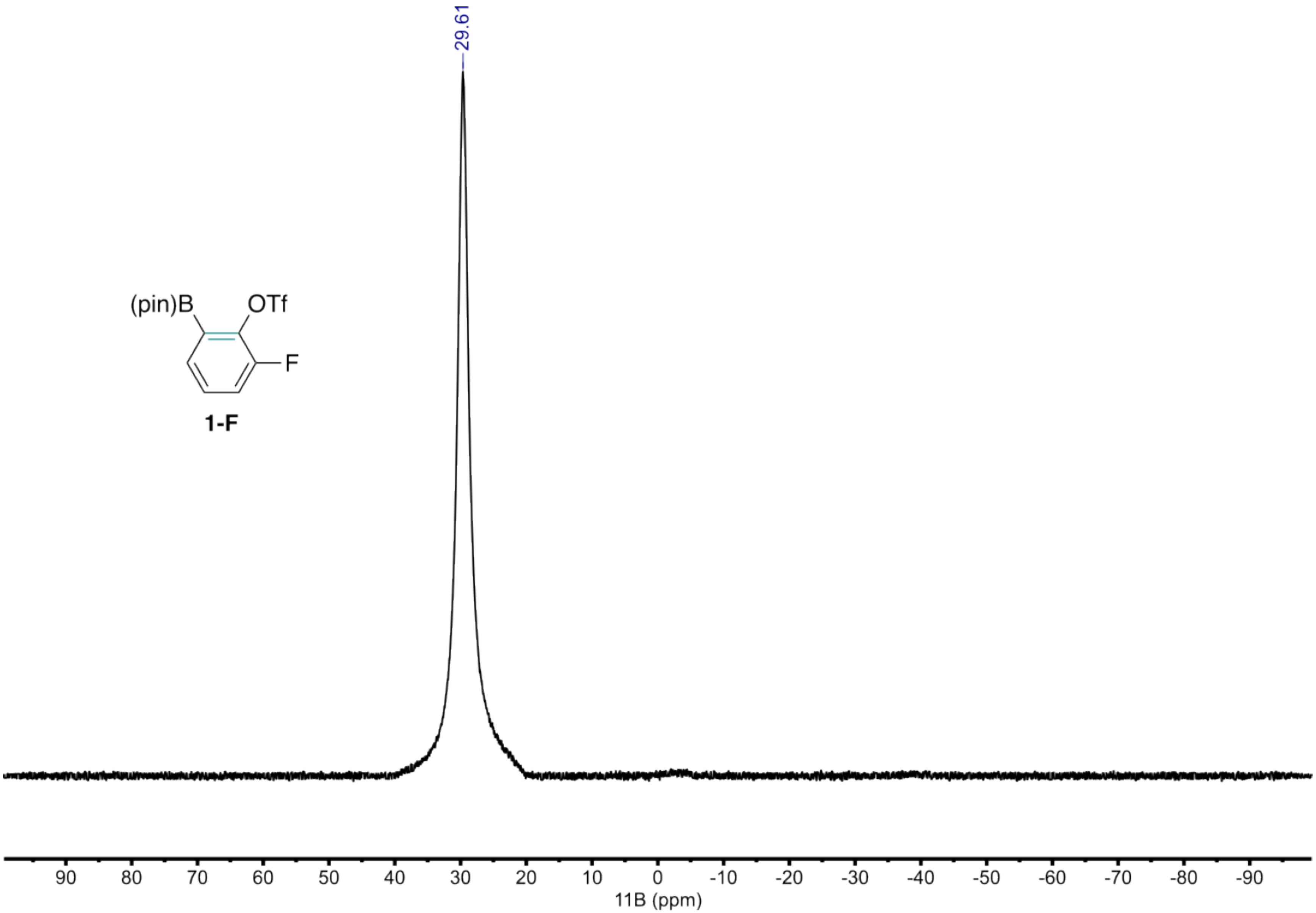

Figure S14. ^{11}B NMR spectrum (128 MHz, $CDCl_3$, 298 K) of **1-F.** Whittaker Smoother baseline correction performed.

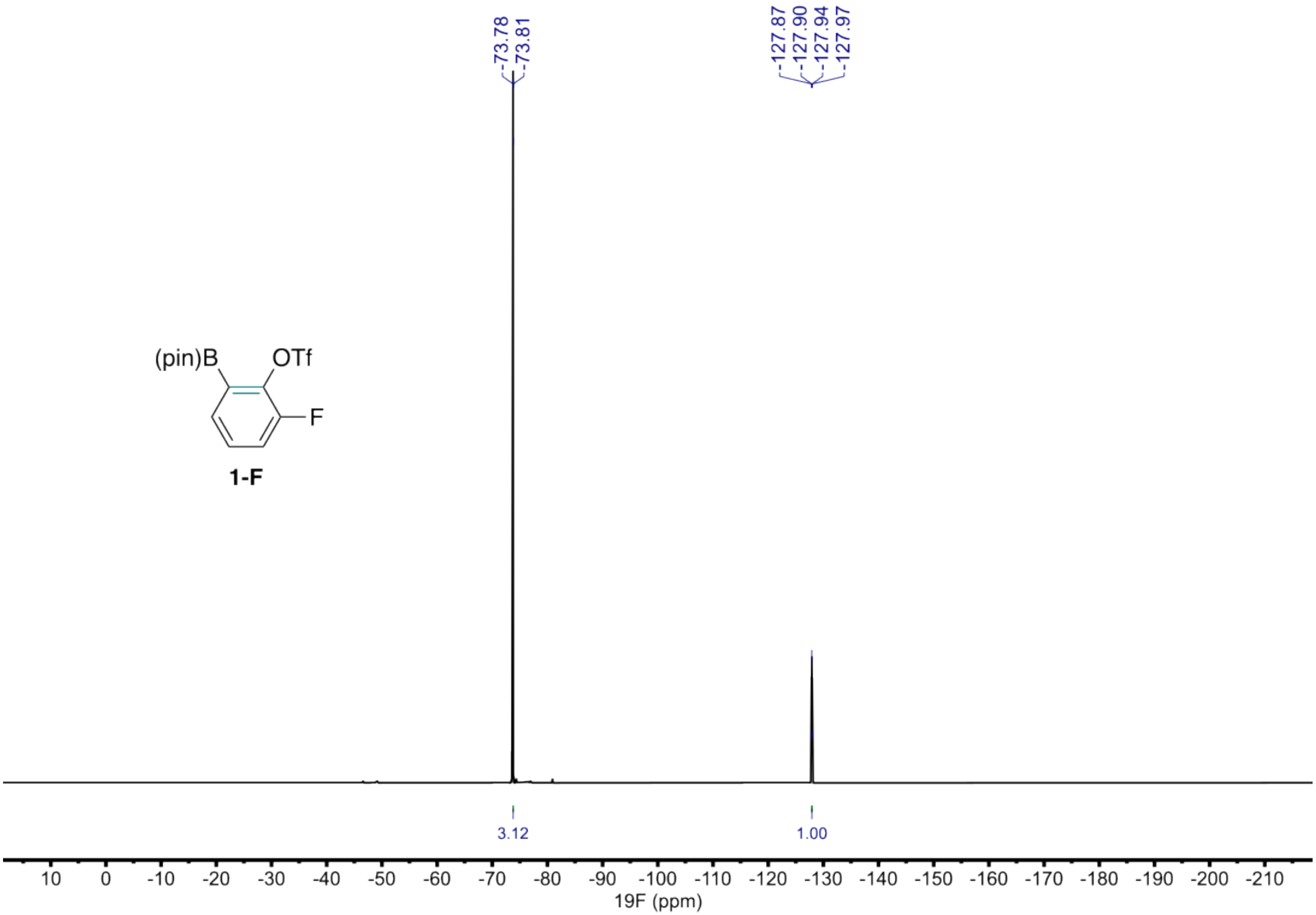

Figure S15. $^{19}F\{^{1}H\}$ NMR spectrum (128 MHz, $CDCl_3$, 298 K) of **1-F.**

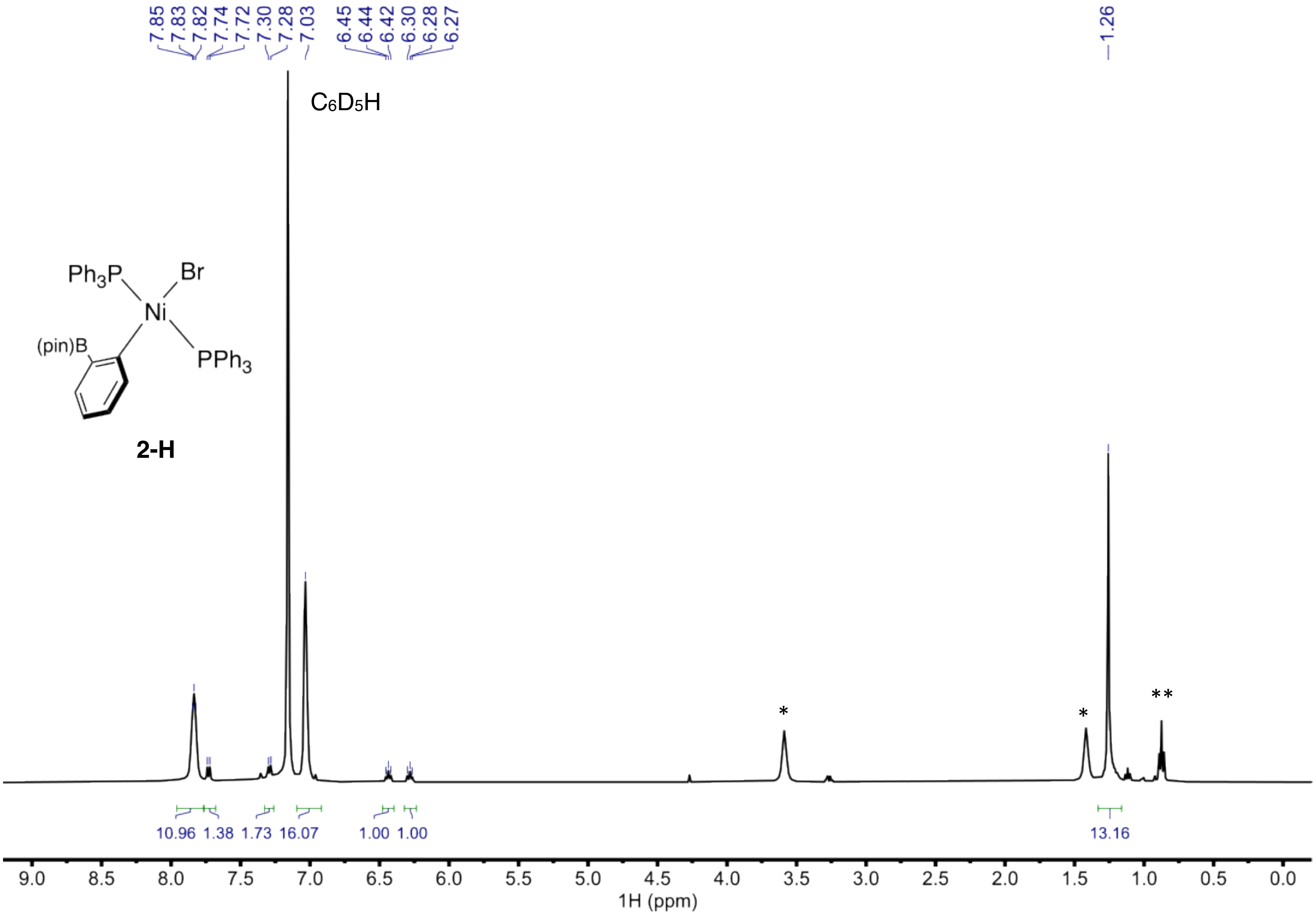

Figure S16. ^{1}H NMR spectrum (400 MHz, C_6D_6, 298 K) of **2-H**.[6] *residual THF **residual pentane

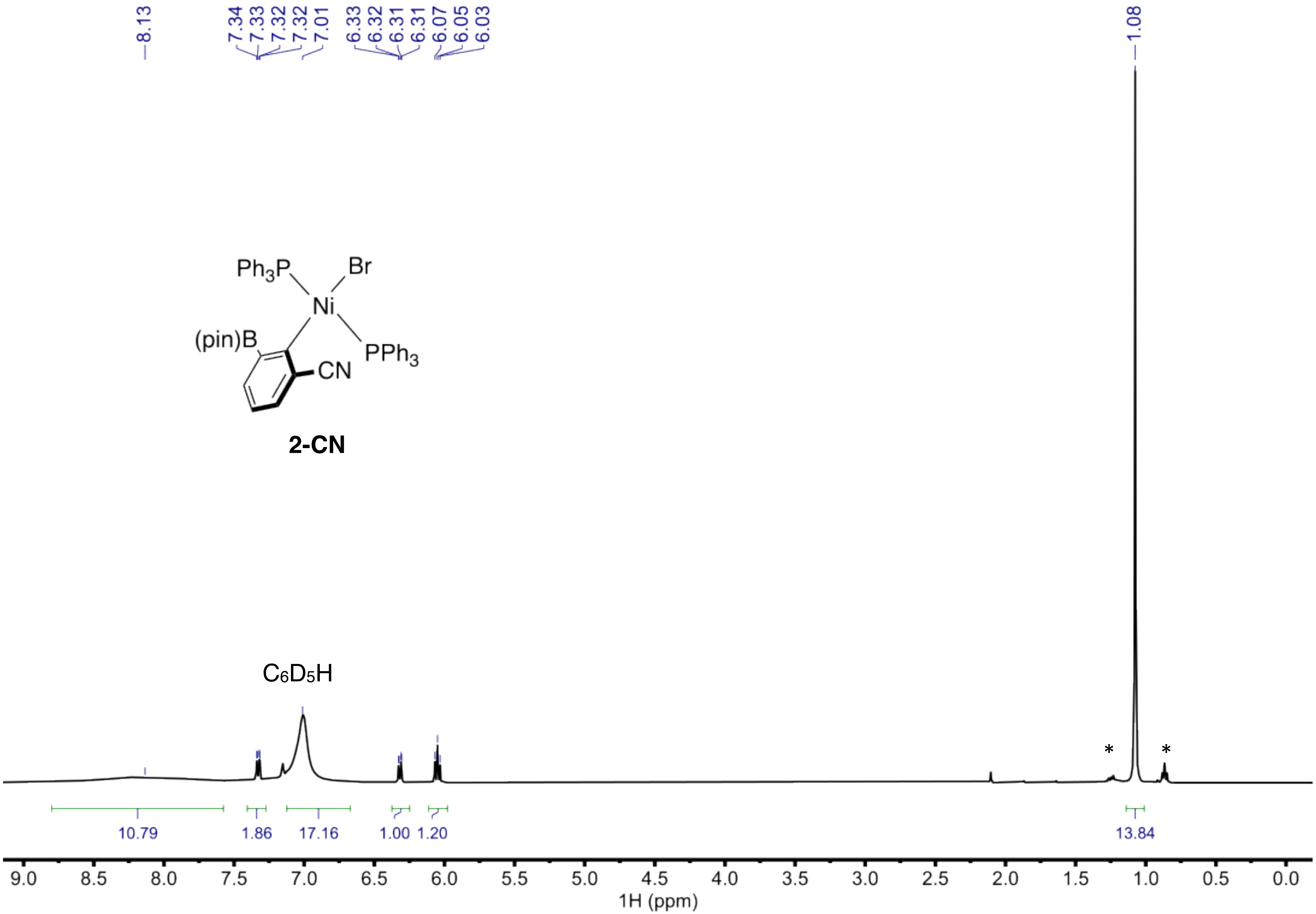

Figure S17. ^{1}H NMR spectrum (400 MHz, C_6D_6, 298 K) of **2-CN**. *residual pentane

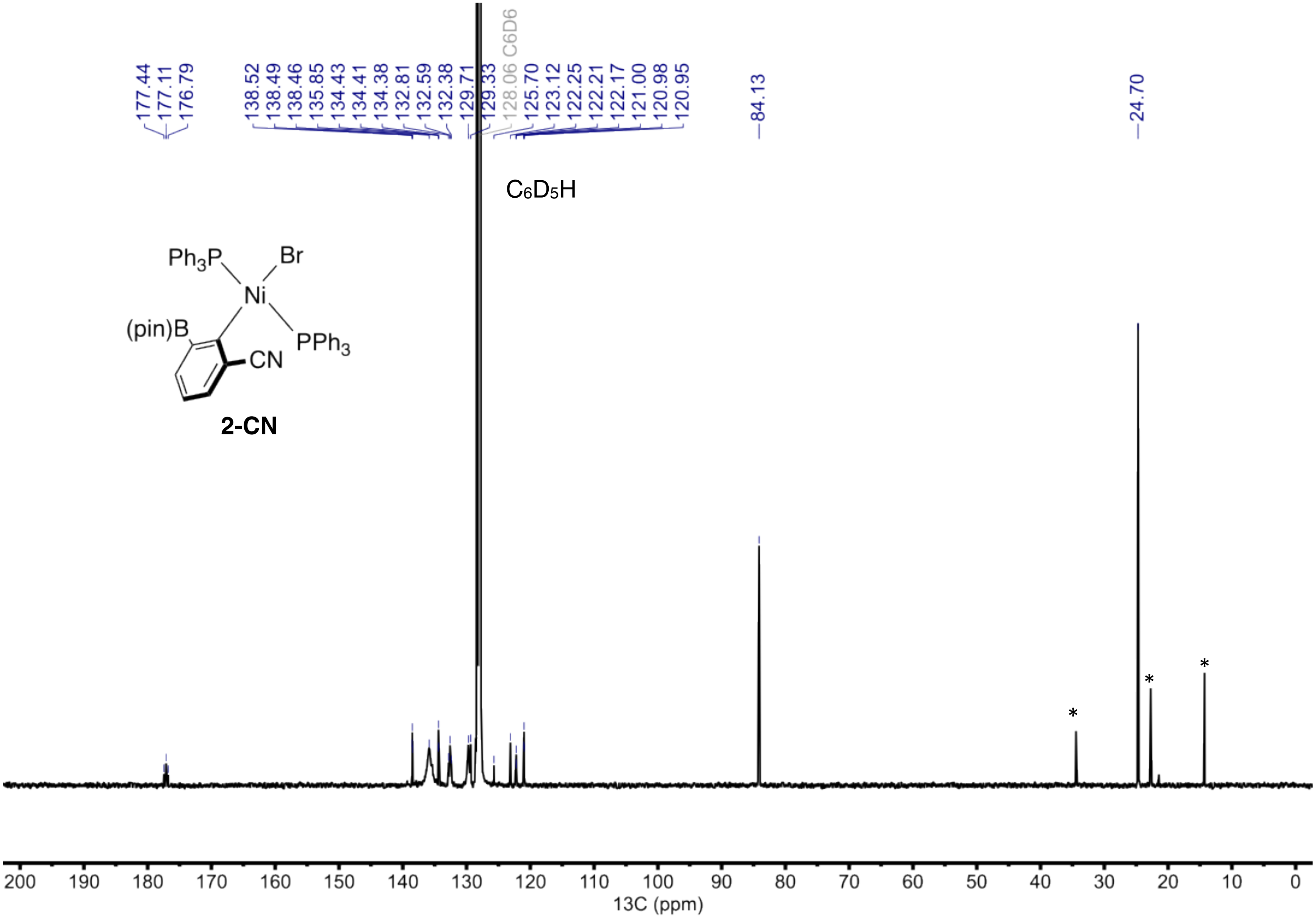

Figure S18. $^{13}C\{^1H\}$ NMR spectrum (101 MHz, C_6D_6, 298 K) of **2-CN**. *residual pentane

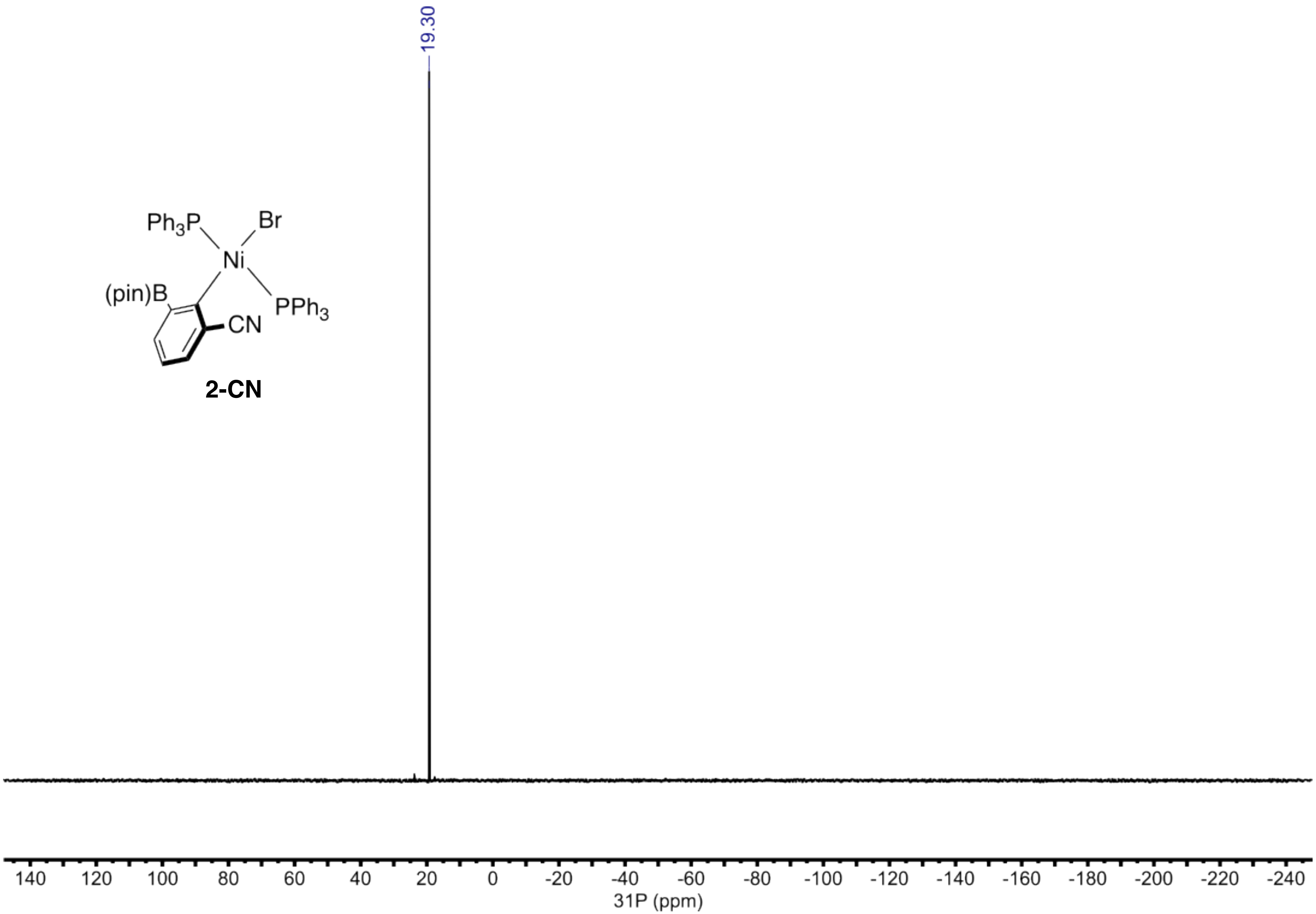

Figure S19. $^{31}P\{^1H\}$ NMR spectrum (162 MHz, C_6D_6, 298 K) of **2-CN**.

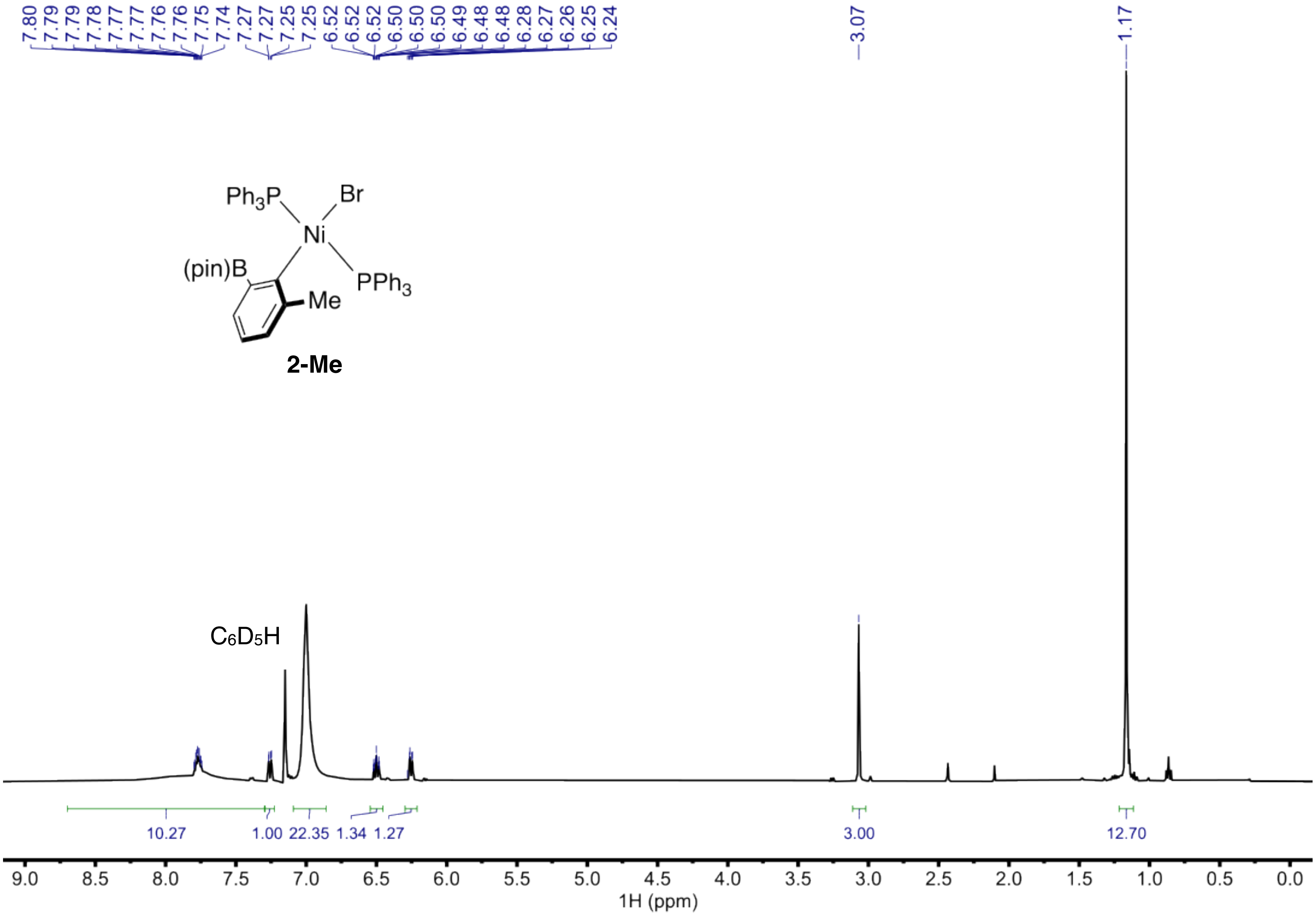

Figure S20. ^{1}H NMR spectrum (400 MHz, C_6D_6, 298 K) of **2-Me**.

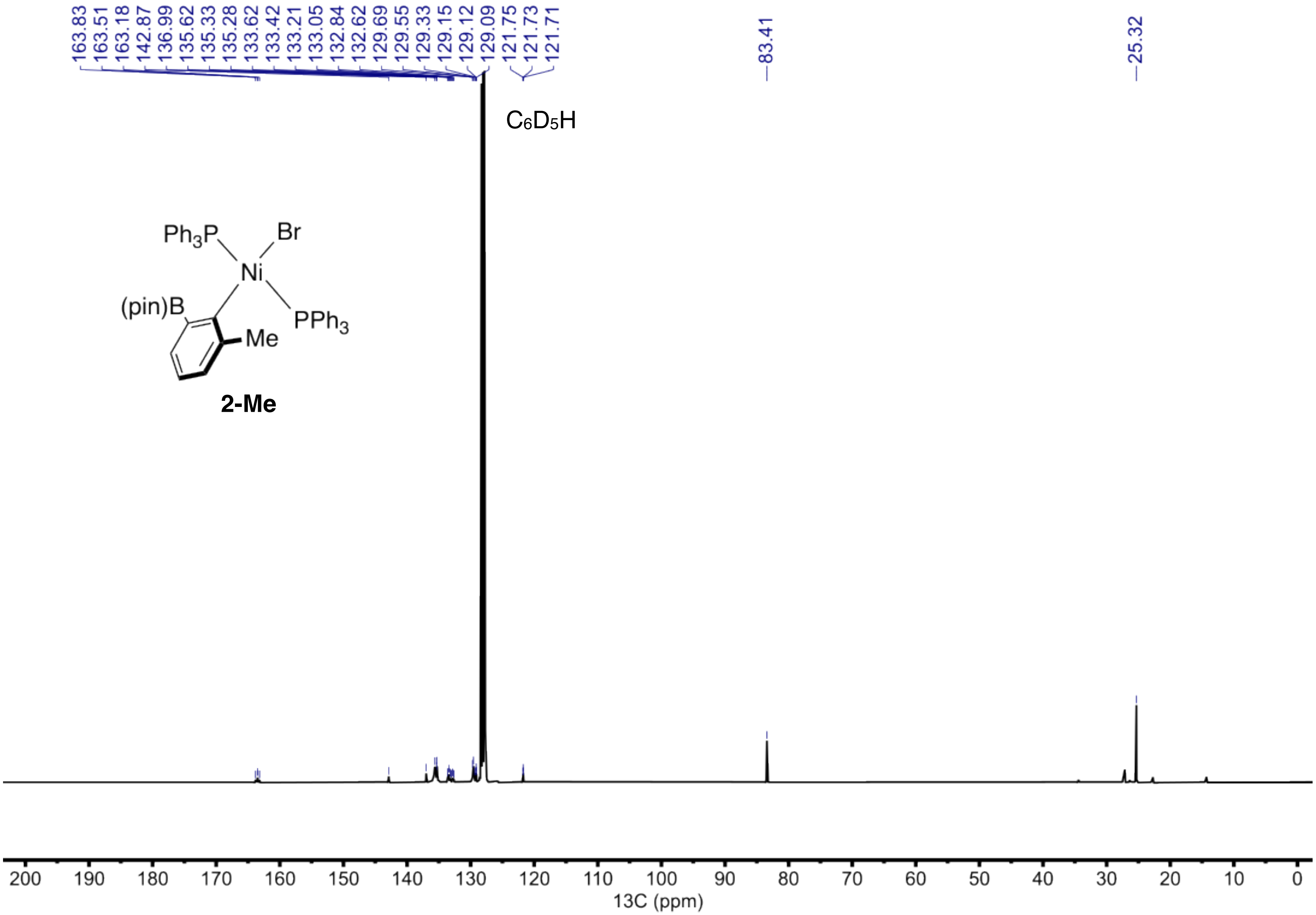

Figure S21. $^{13}C\{^{1}H\}$ NMR spectrum (101 MHz, C_6D_6, 298 K) of **2-Me**.

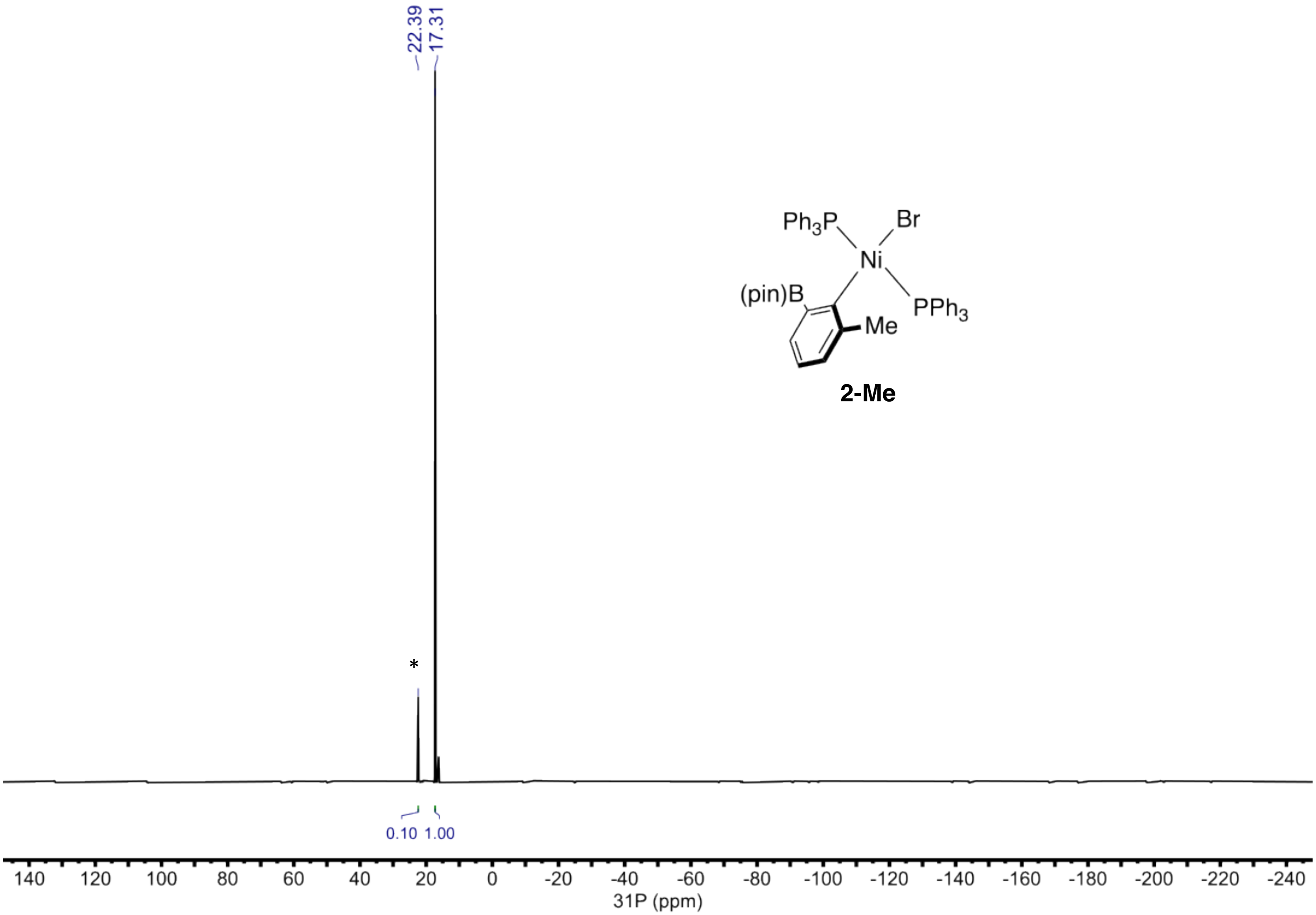

Figure S22. $^{31}P\{^{1}H\}$ NMR spectrum (162 MHz, C_6D_6, 298 K) of **2-Me**. *unknown impurity

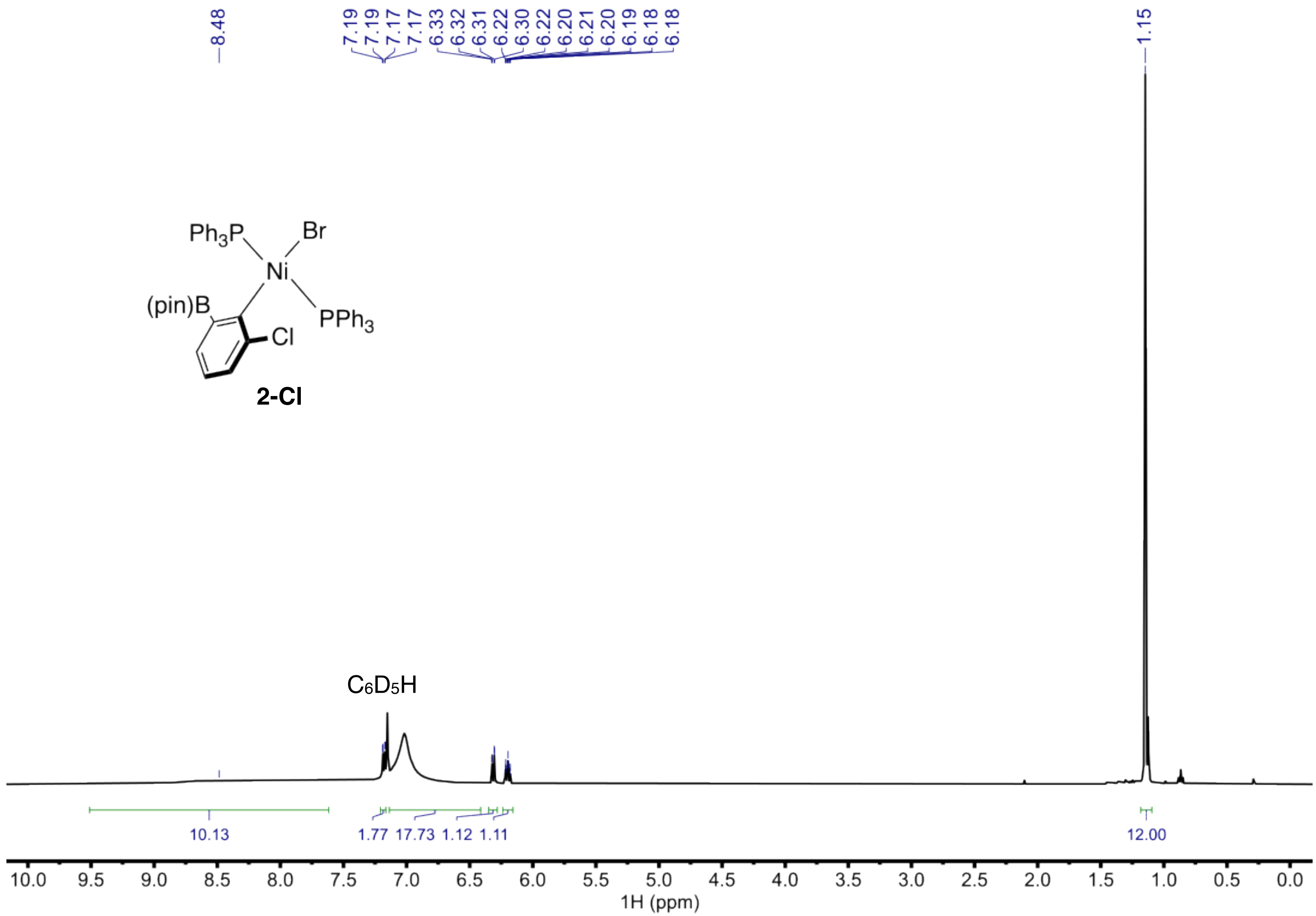

Figure S23. ^{1}H NMR spectrum (400 MHz, C_6D_6, 298 K) of **2-Cl**.

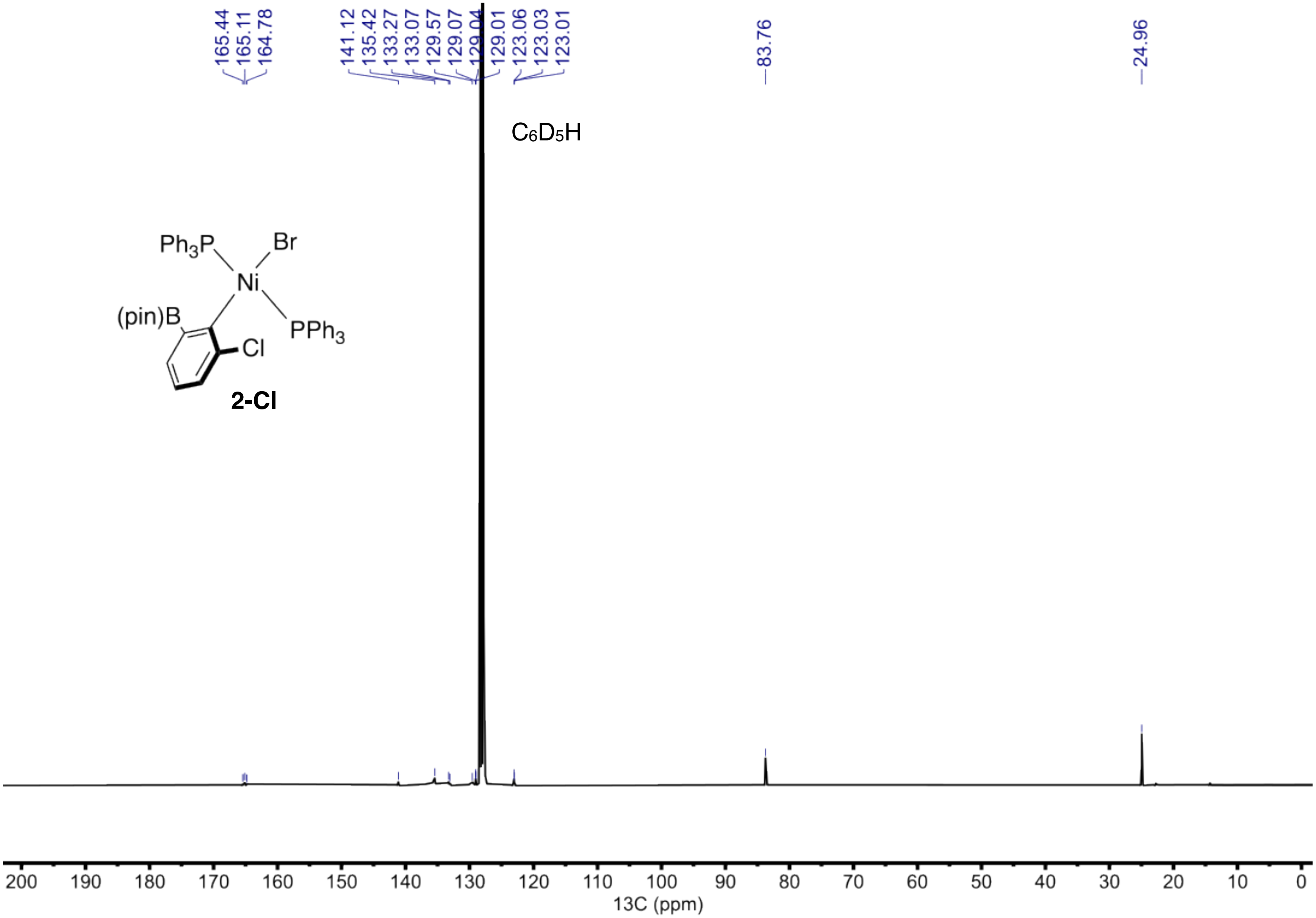

Figure S24. $^{13}C\{^1H\}$ NMR spectrum (101 MHz, C_6D_6, 298 K) of **2-Cl**.

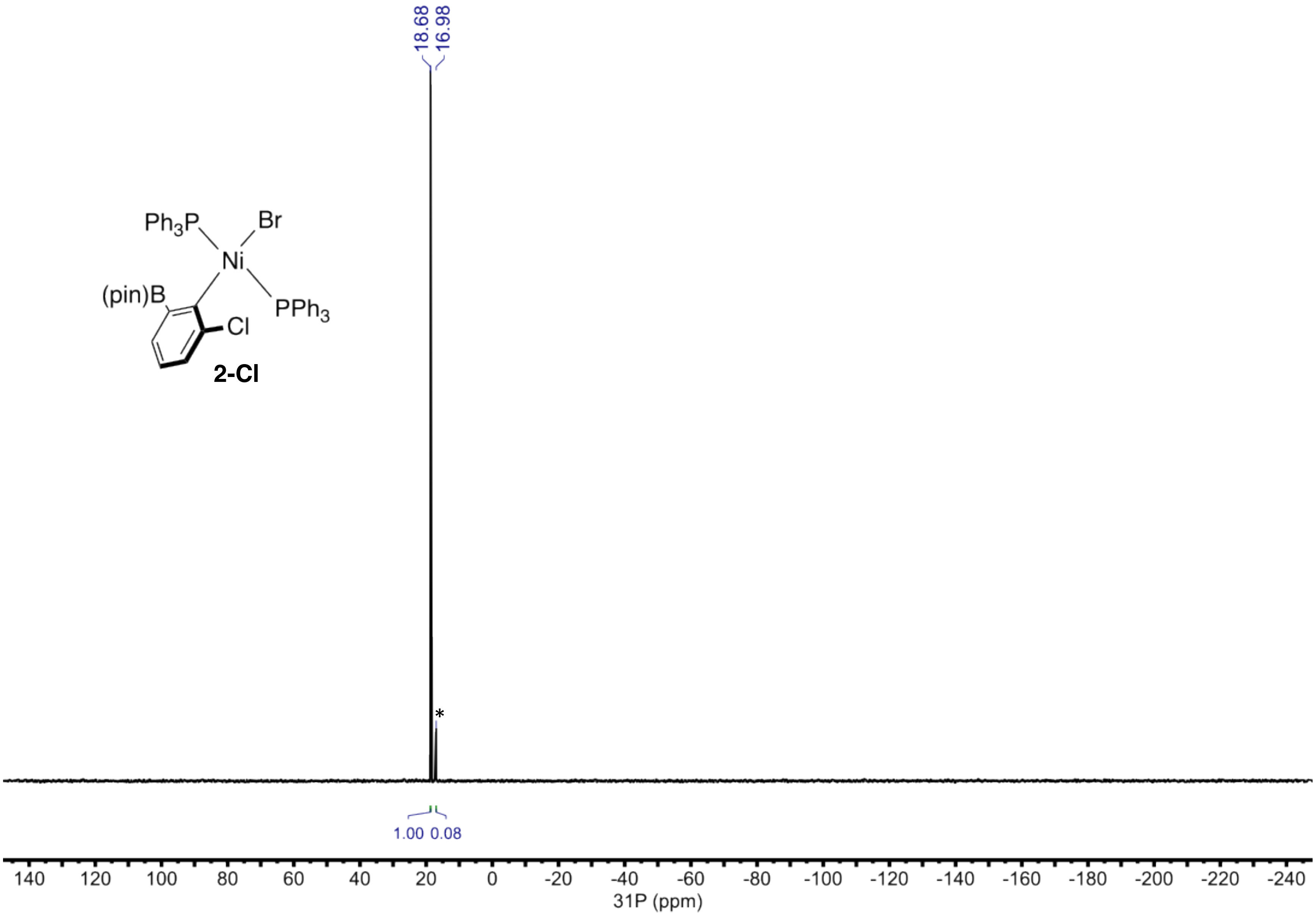

Figure S25. $^{31}P\{^{1}H\}$ NMR spectrum (162 MHz, C_6D_6, 298 K) of **2-Cl**. *unknown impurity

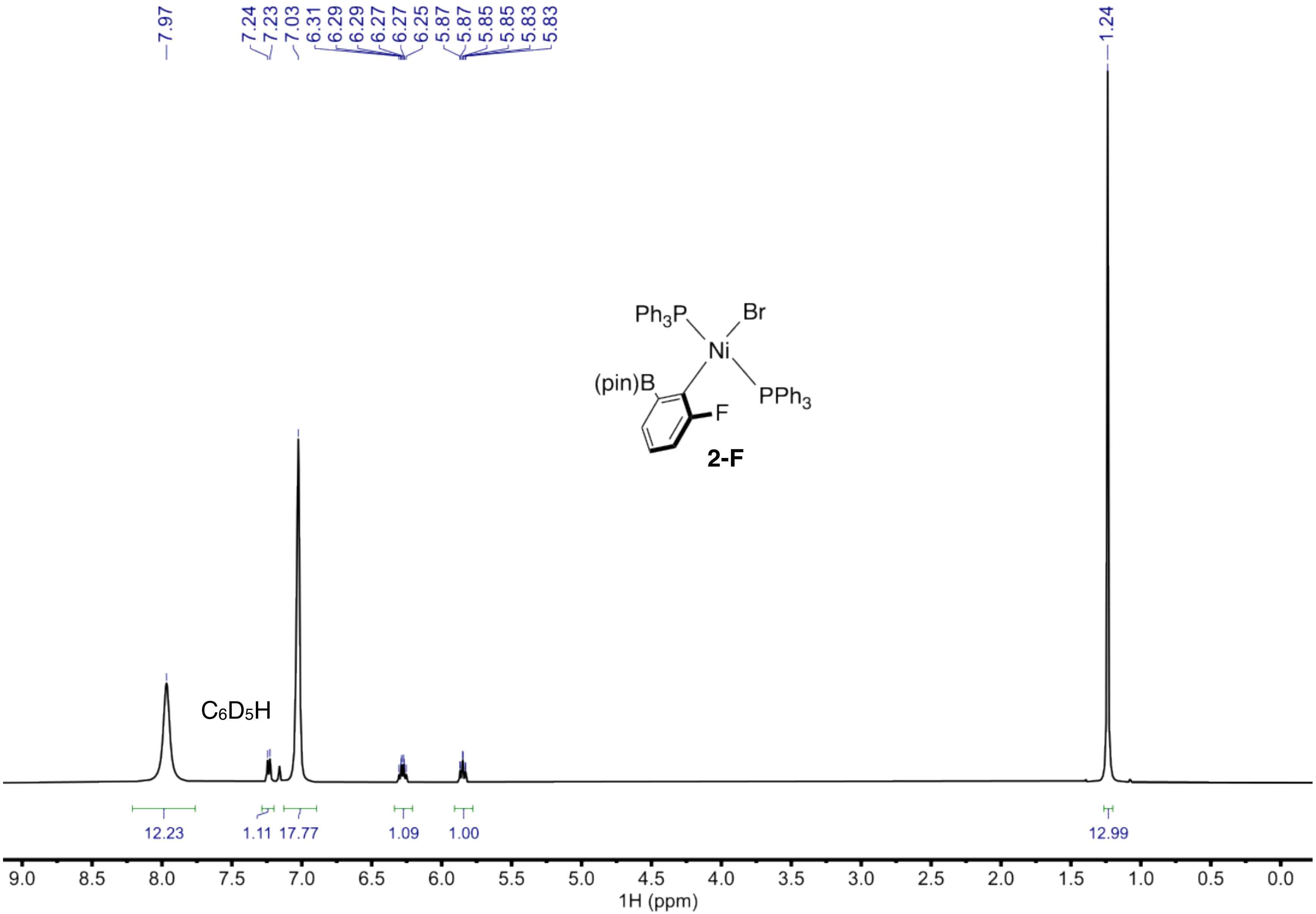

Figure S26. ^{1}H NMR spectrum (400 MHz, C_6D_6, 298 K) of **2-F**.

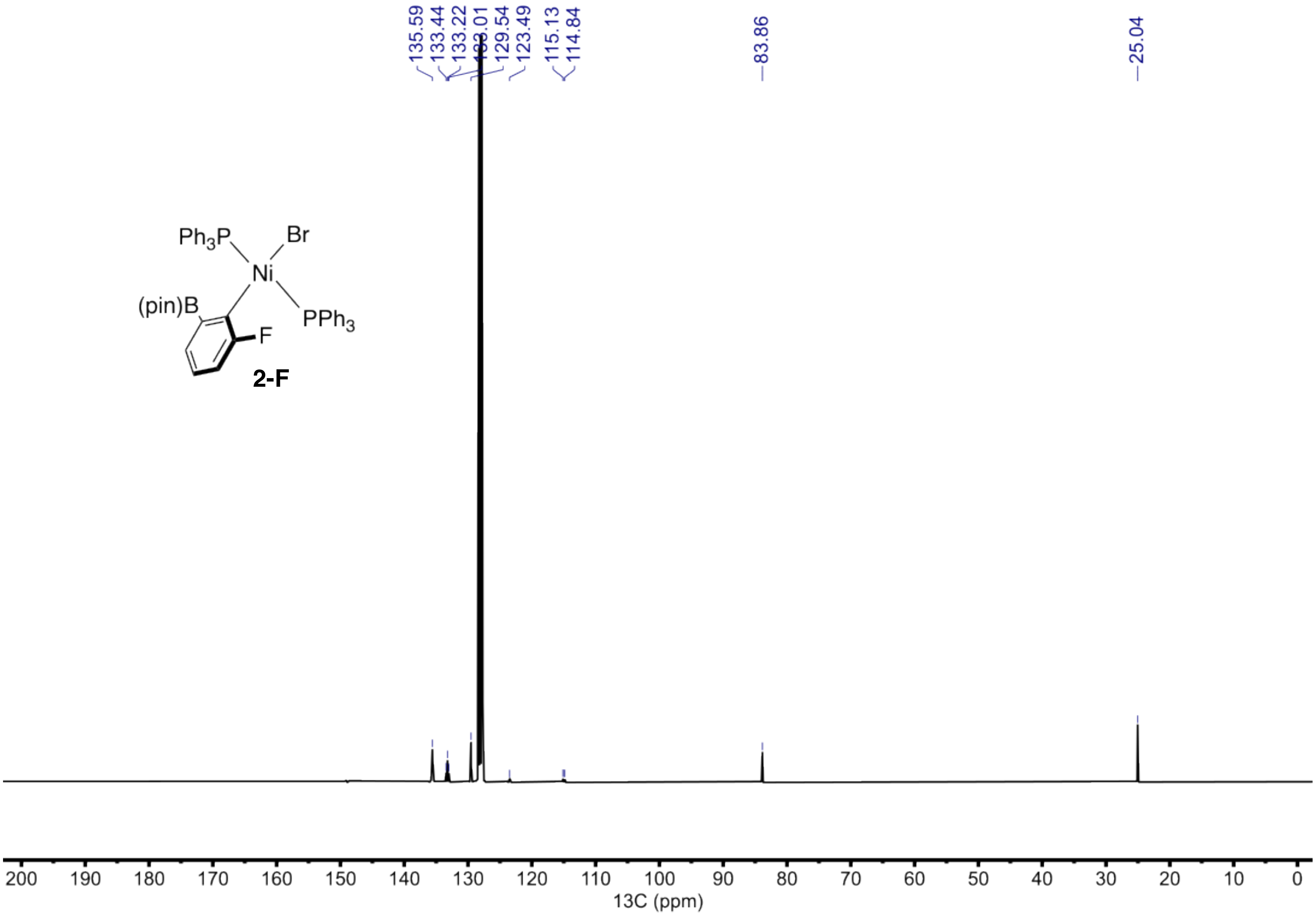

Figure S27. $^{13}C\{^1H\}$ NMR spectrum (101 MHz, C_6D_6, 298 K) of **2-F**.

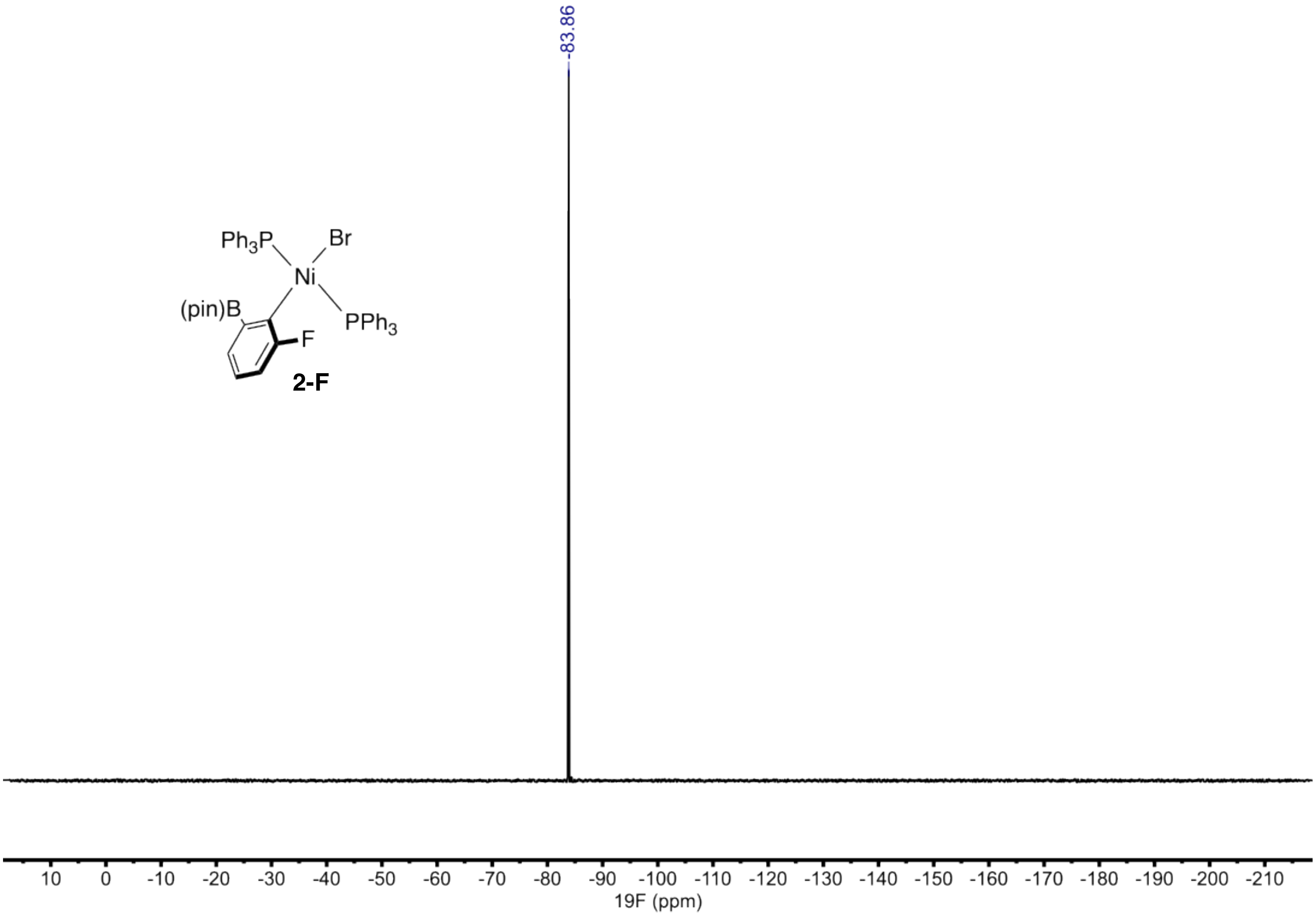

Figure S28. $^{19}F\{^1H\}$ NMR spectrum (128 MHz, C_6D_6, 298 K) of **2-F**. Whittaker Smoother baseline correction performed.

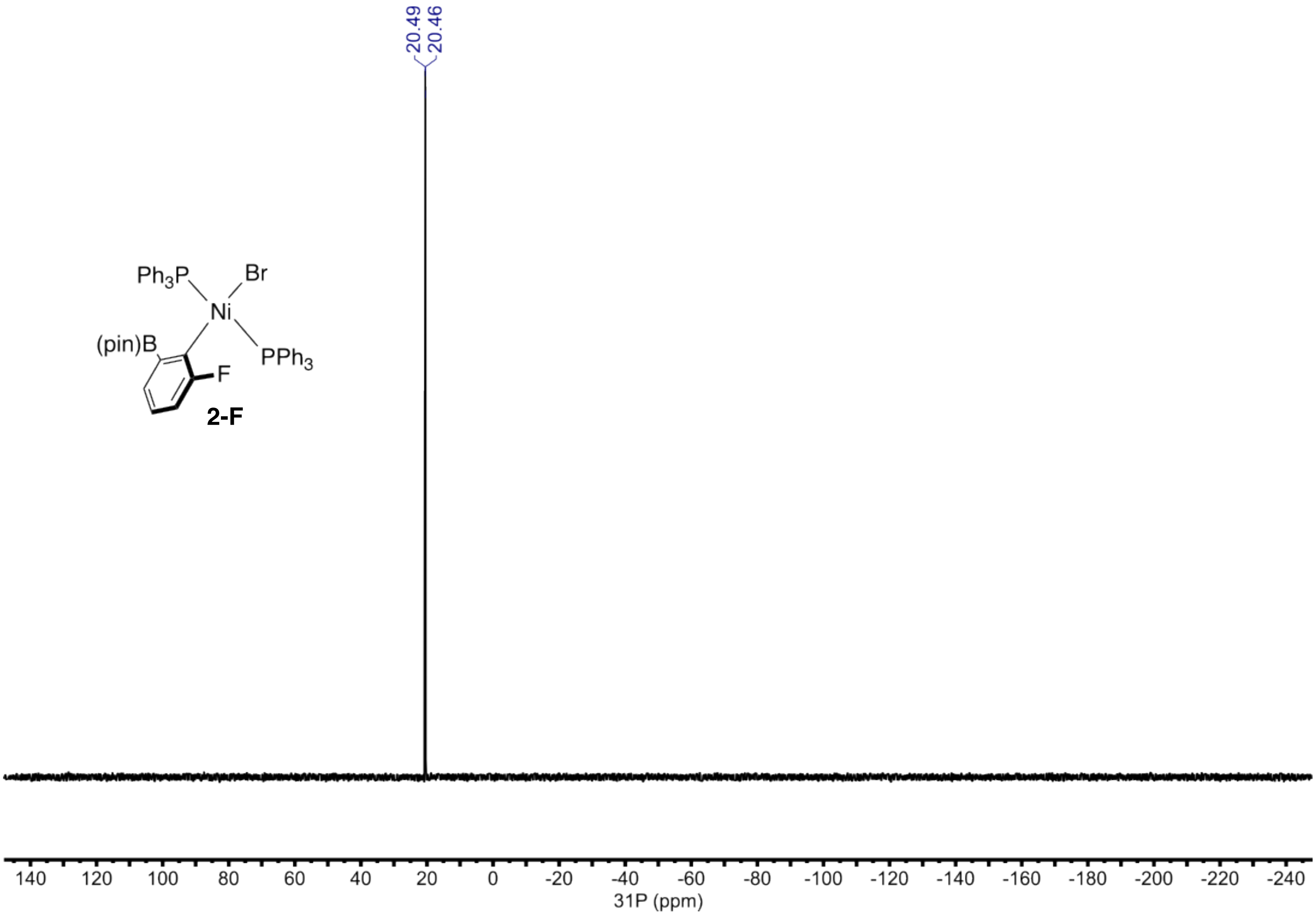

Figure S29. $^{31}P\{^{1}H\}$ NMR spectrum (162 MHz, C_6D_6, 298 K) of **2-F**.

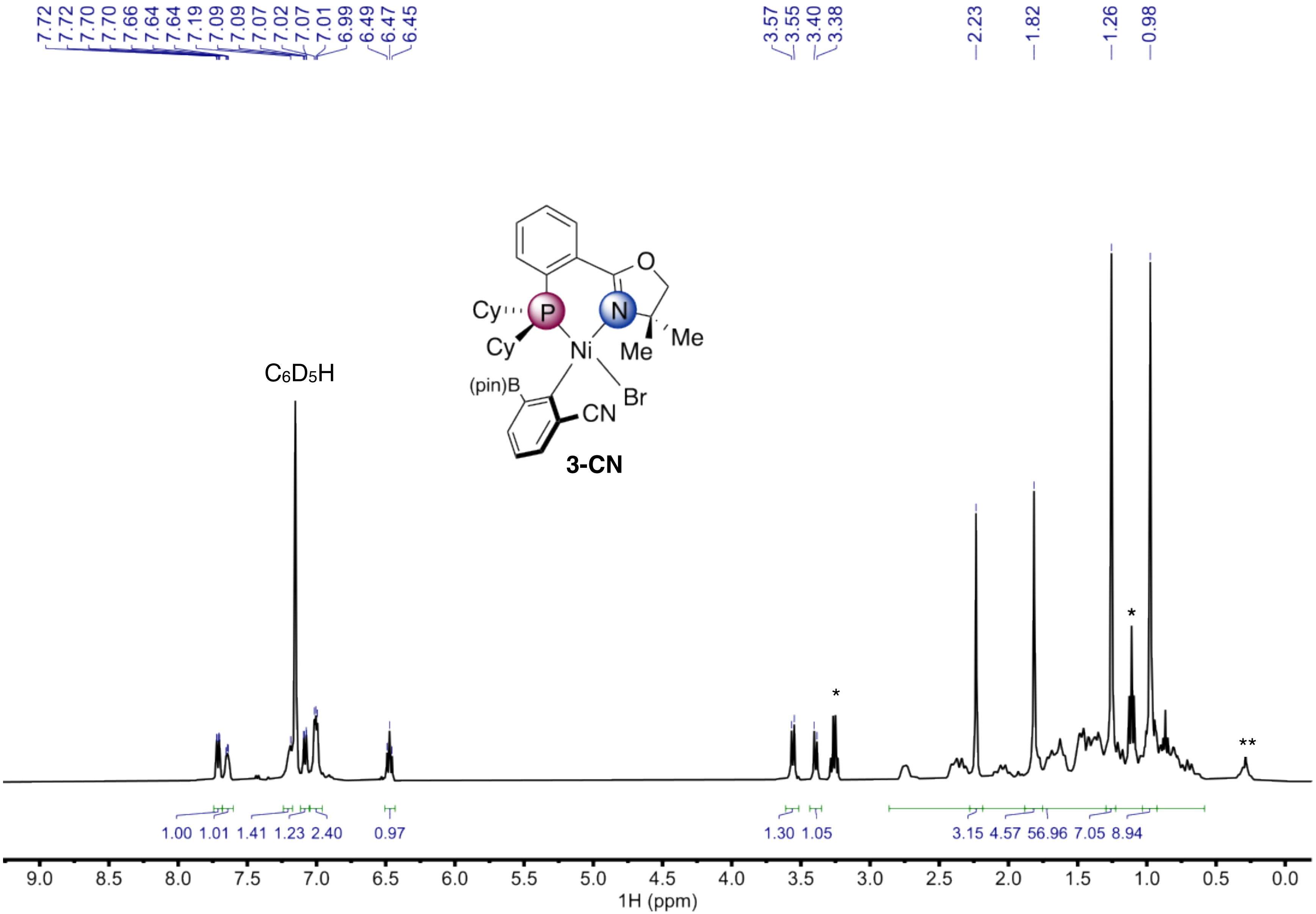

Figure S30. ^{1}H NMR spectrum (400 MHz, C_6D_6, 298 K) of **3-CN**. *residual diethyl ether **silicone grease

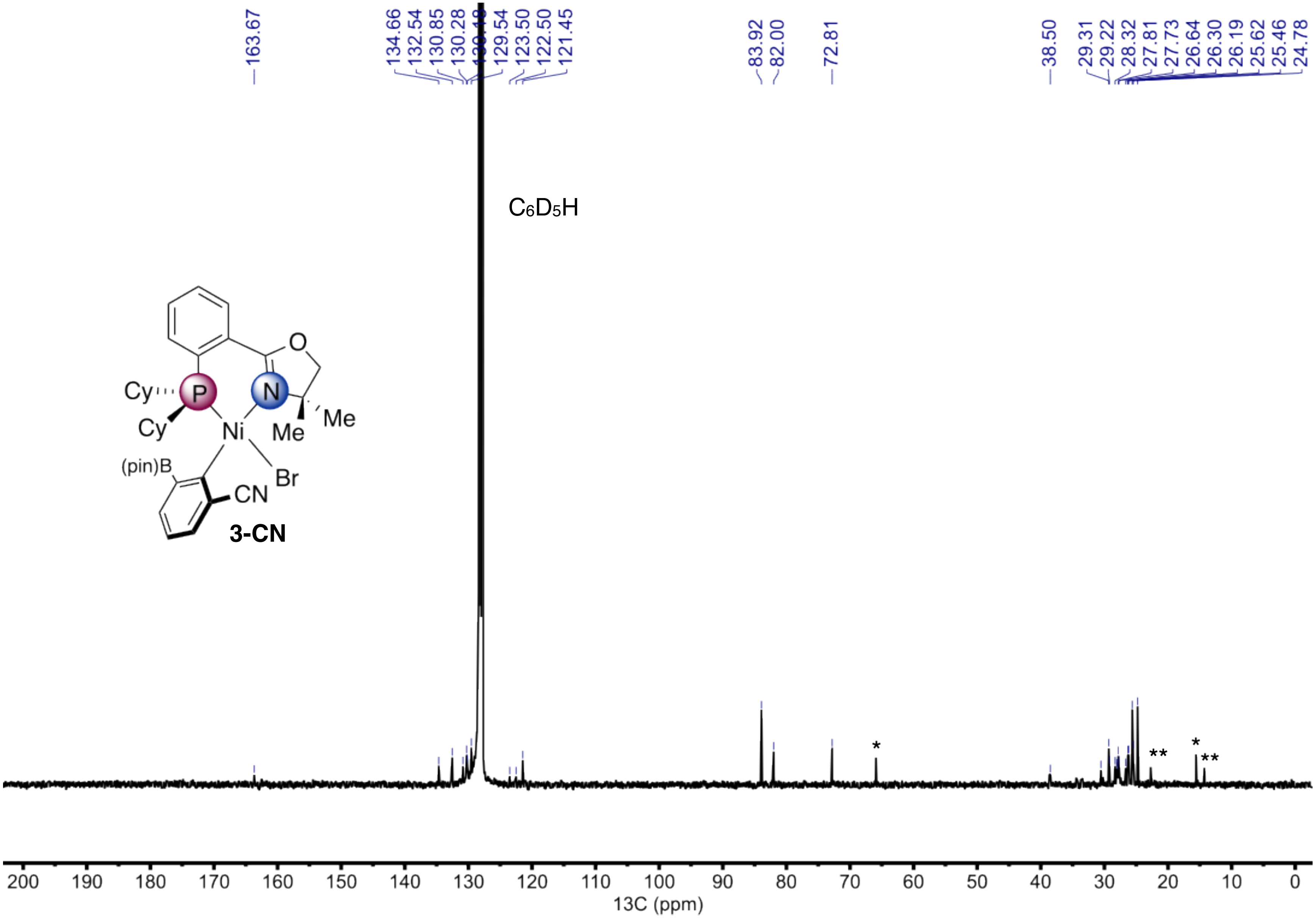

Figure S31. $^{13}C\{^{1}H\}$ NMR spectrum (101 MHz, C_6D_6, 298 K) of **3-CN**. *residual diethyl ether **pentane

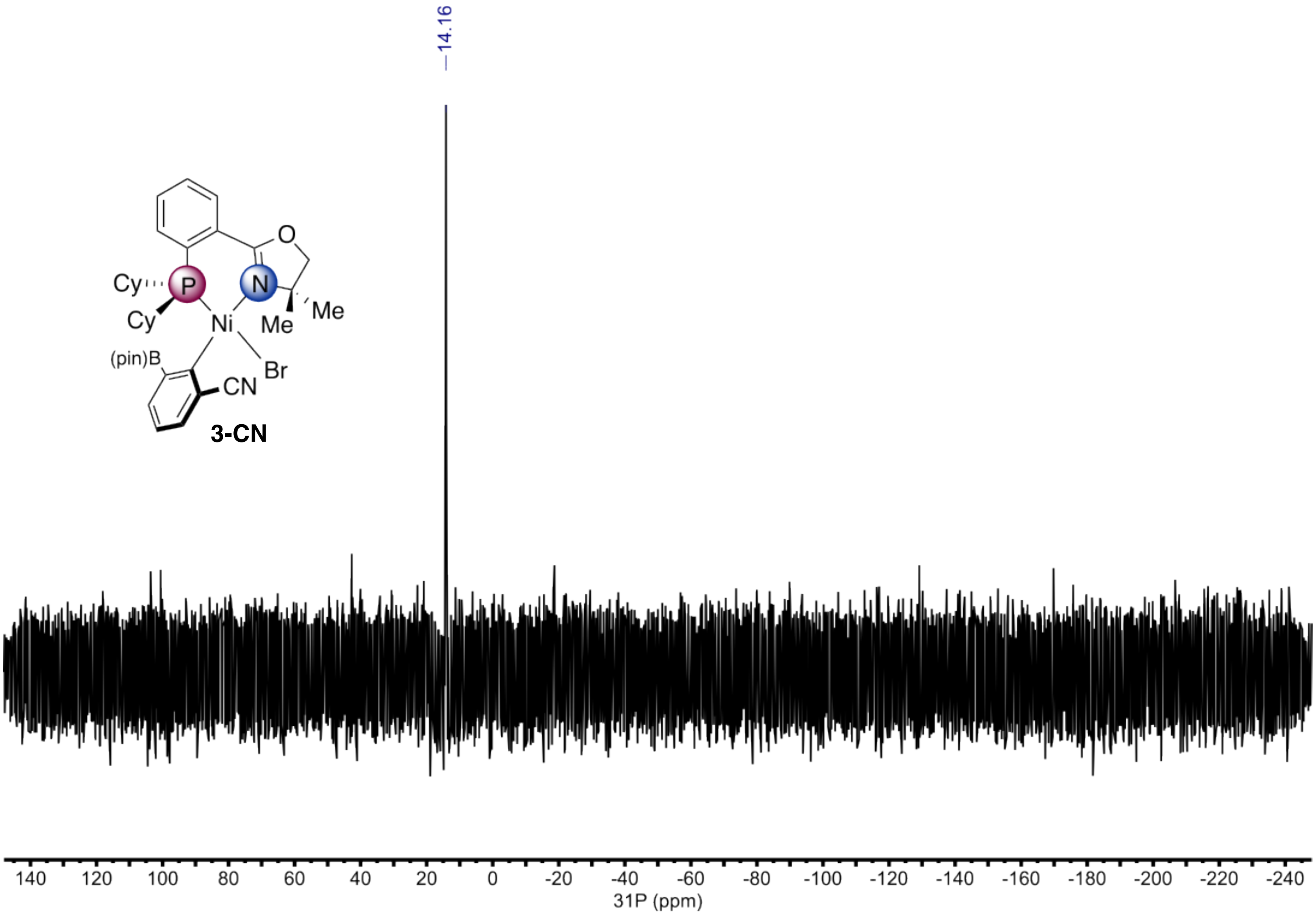

Figure S32. $^{31}P\{^{1}H\}$ NMR spectrum (162 MHz, C_6D_6, 298 K) of **3-CN**.

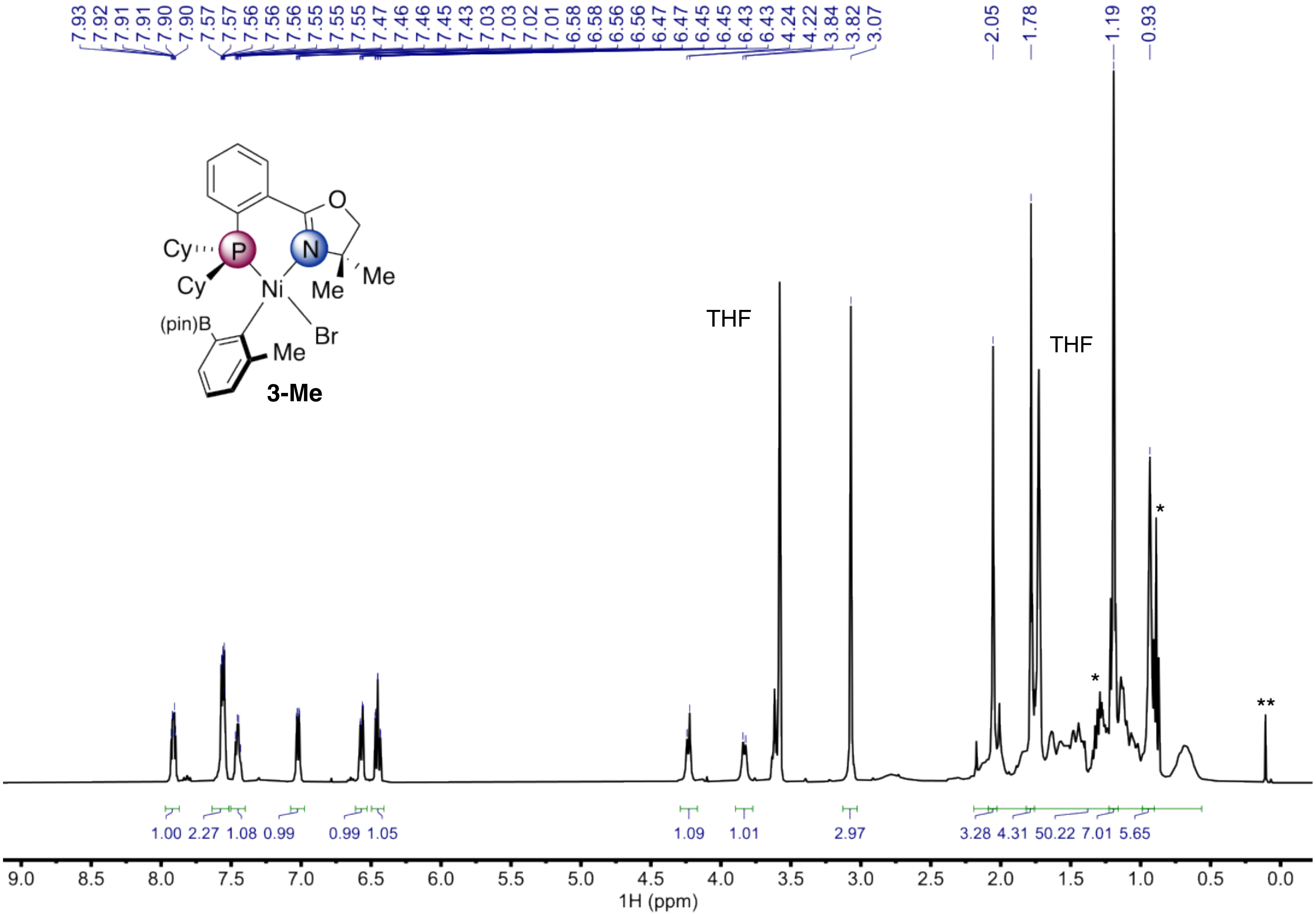

Figure S33. ^{1}H NMR spectrum (400 MHz, THF-d_8, 298 K) of **3-Me**. *residual pentane **silicone grease

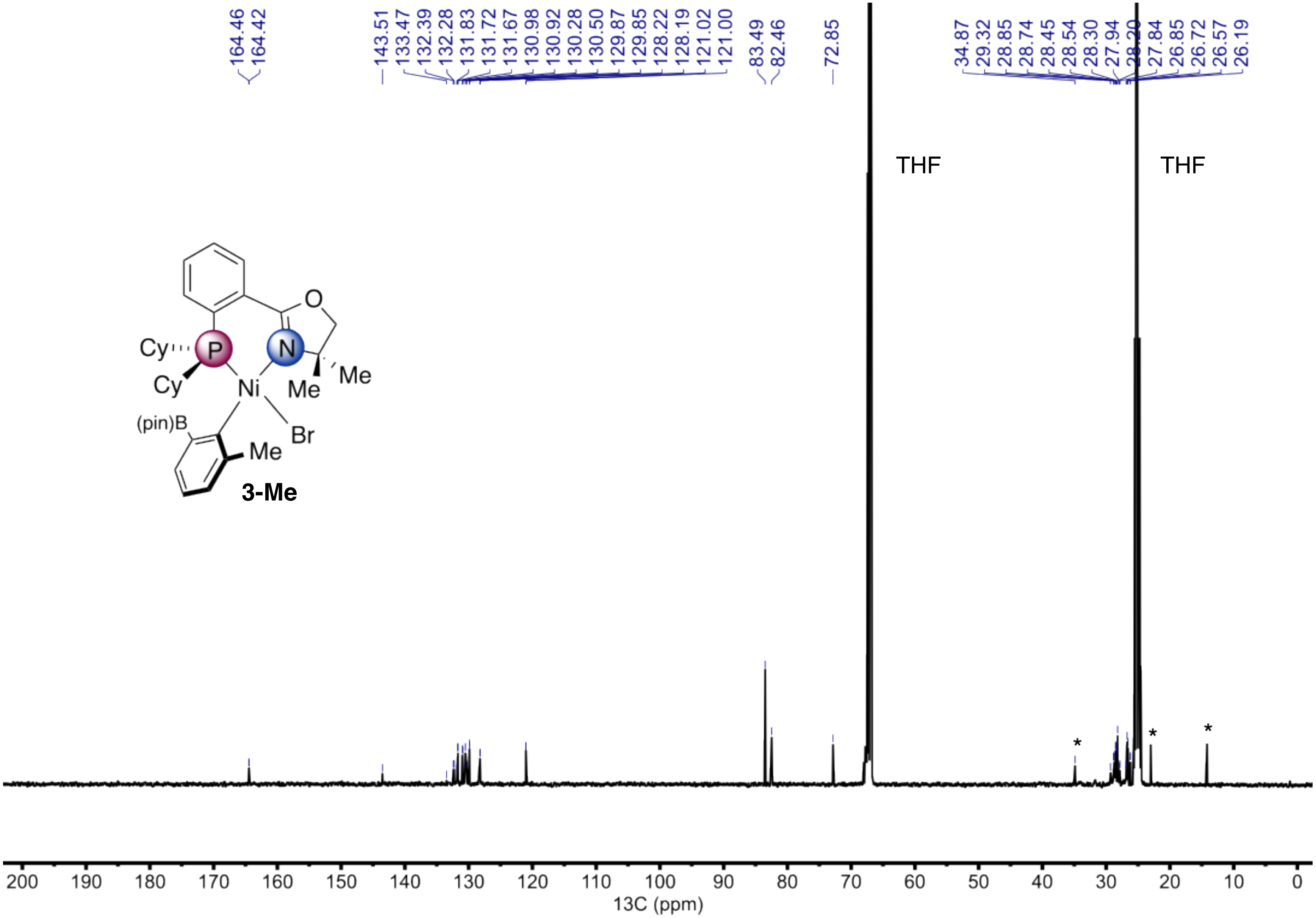

Figure S34. $^{13}C\{^{1}H\}$ NMR spectrum (101 MHz, THF-d_8, 298 K) of **3-Me**. *residual pentane

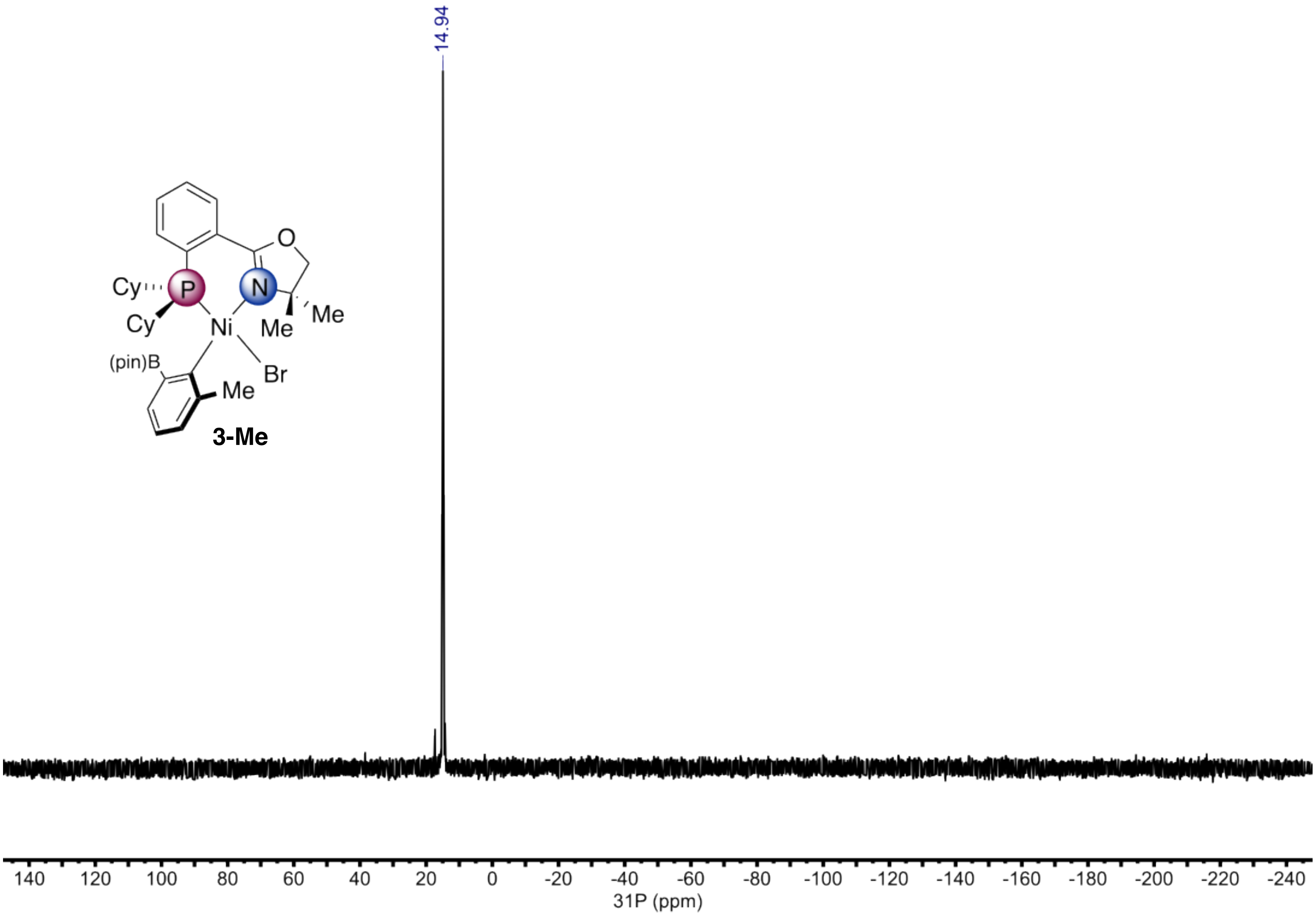

Figure S35. $^{31}P\{^{1}H\}$ NMR spectrum (162 MHz, THF-d_8, 298 K) of **3-Me**.

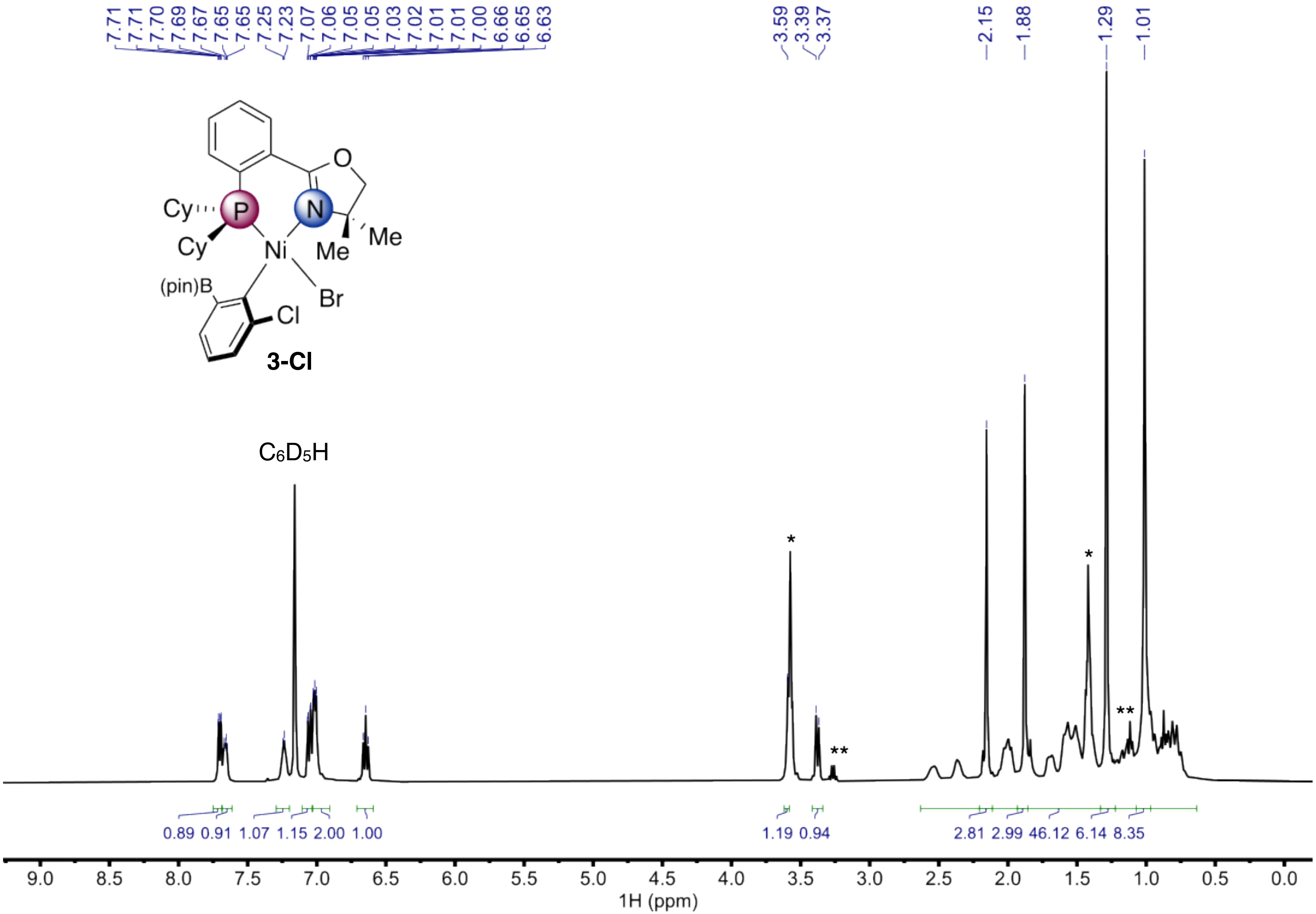

Figure S36. ^{1}H NMR spectrum (101 MHz, C_6D_6, 298 K) of **3-Cl**. *residual THF **diethyl ether

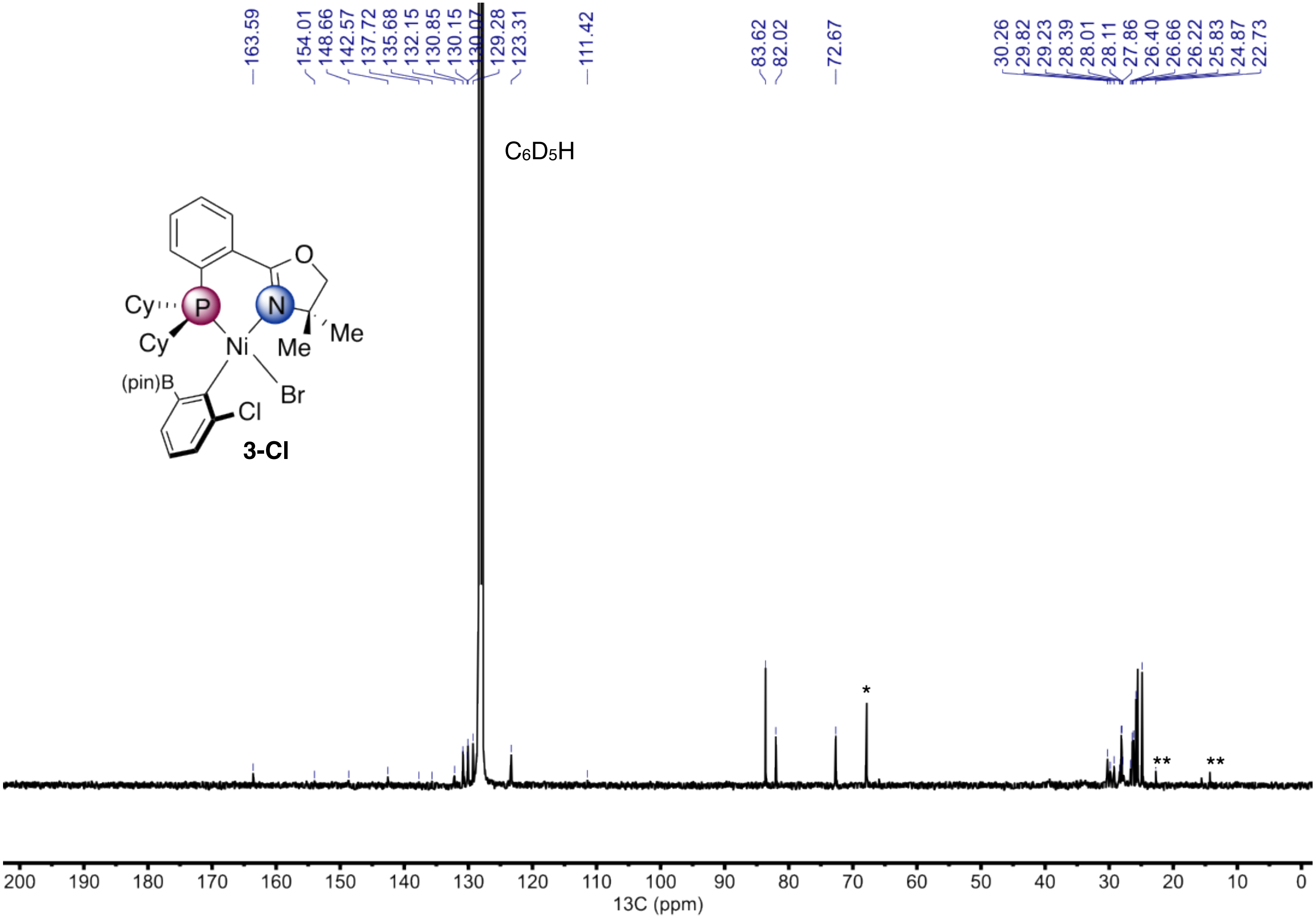

Figure S37. $^{13}C\{^{1}H\}$ NMR spectrum (400 MHz, C_6D_6, 298 K) of **3-Cl**. *residual THF **pentane

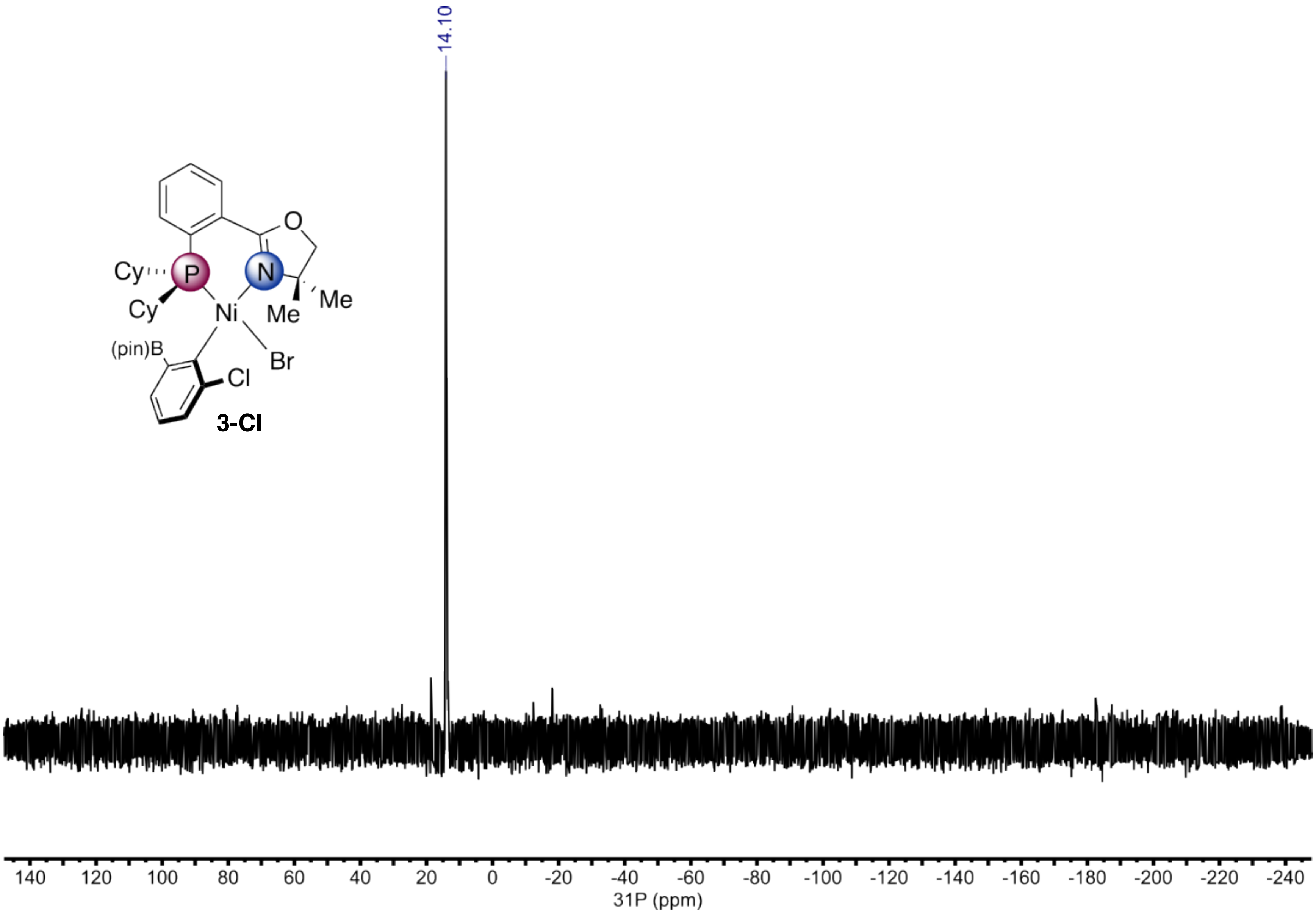

Figure S38. $^{31}P\{^{1}H\}$ NMR spectrum (162 MHz, C_6D_6, 298 K) of **3-Cl**.

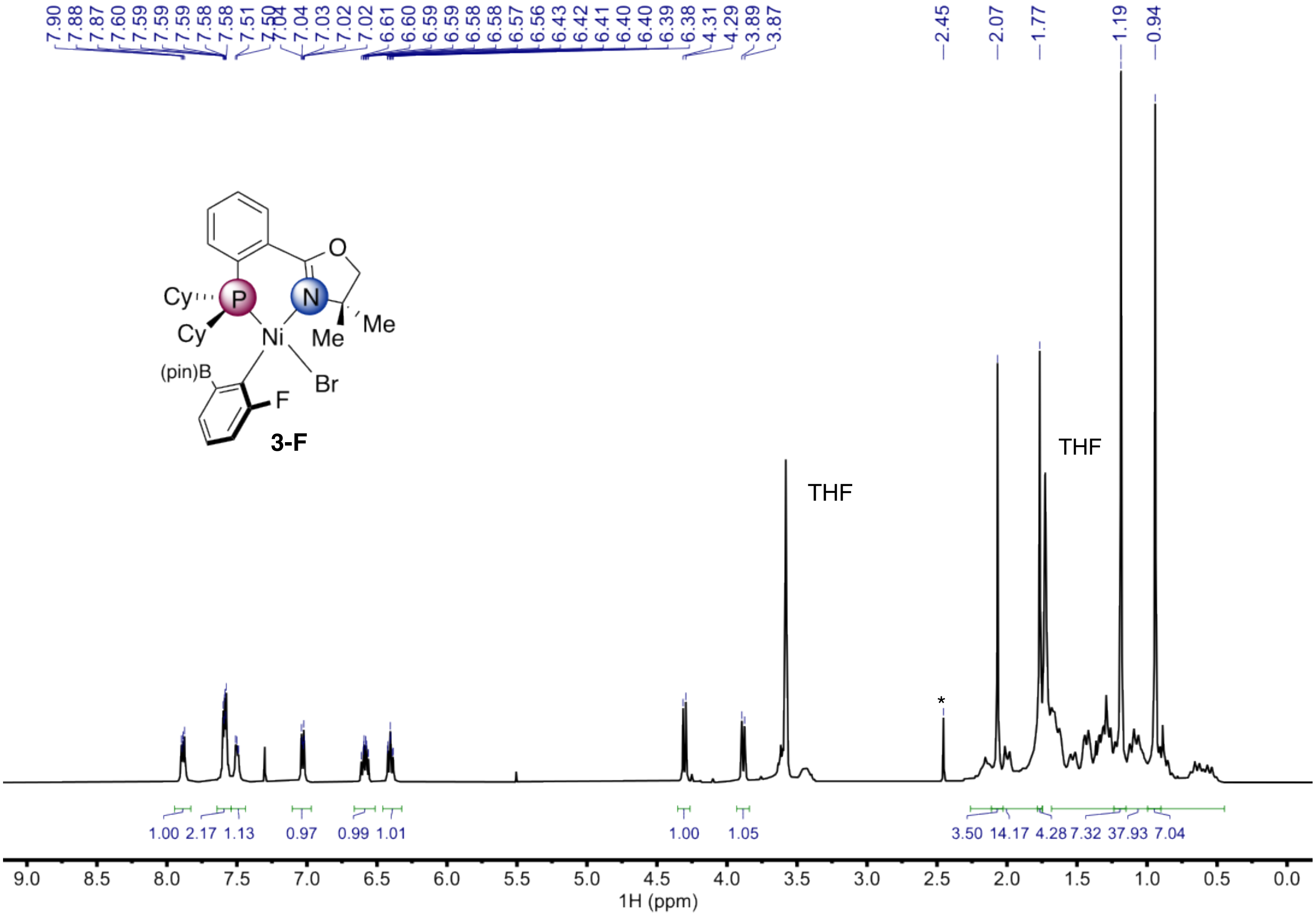

Figure S39. ^1H NMR spectrum (101 MHz, THF–d_8, 298 K) of **3-F**. *residual water from THF-d_8

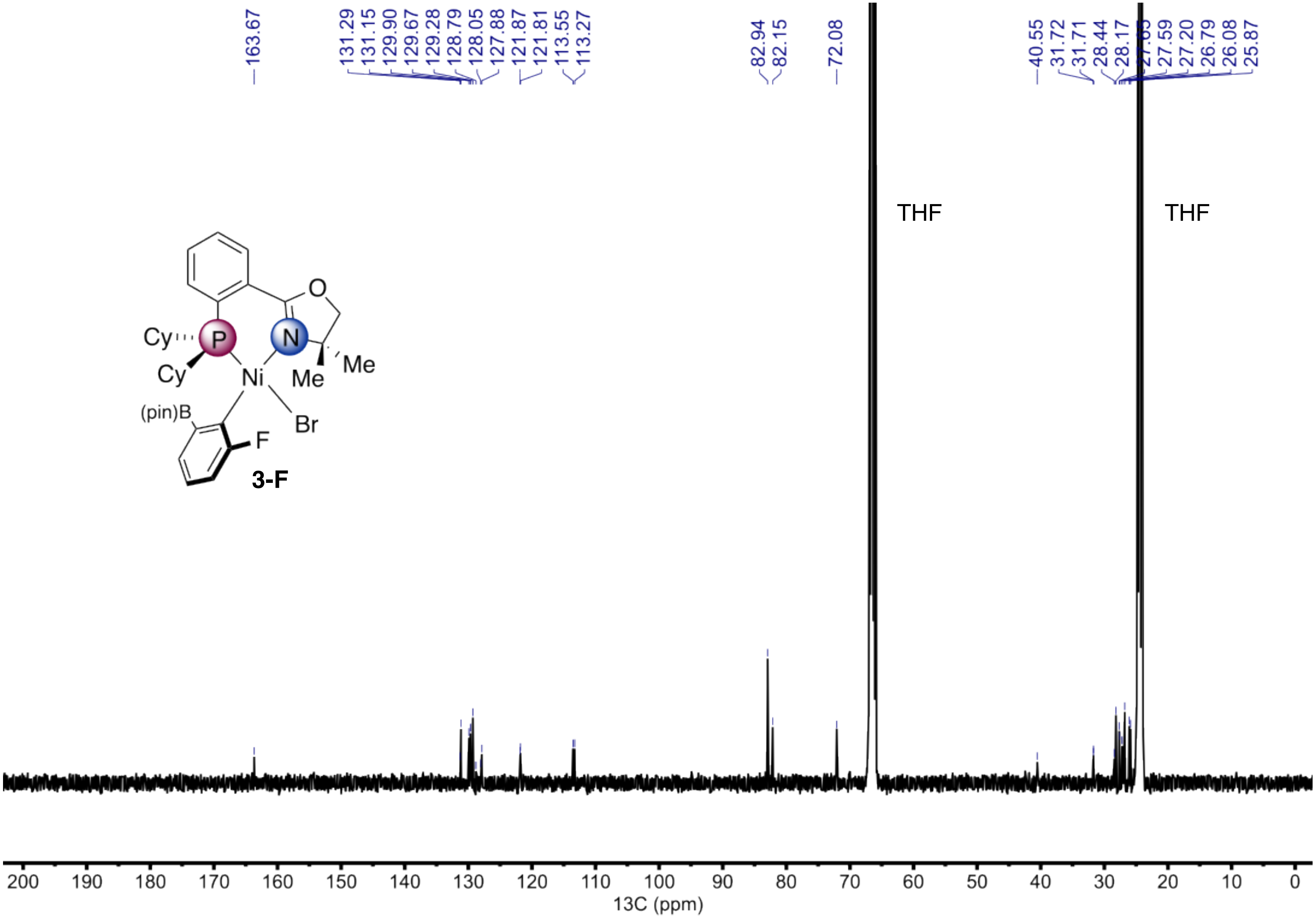

Figure S40. $^{13}C\{^{1}H\}$ NMR spectrum (101 MHz, THF–d_8, 298 K) of **3-F**.

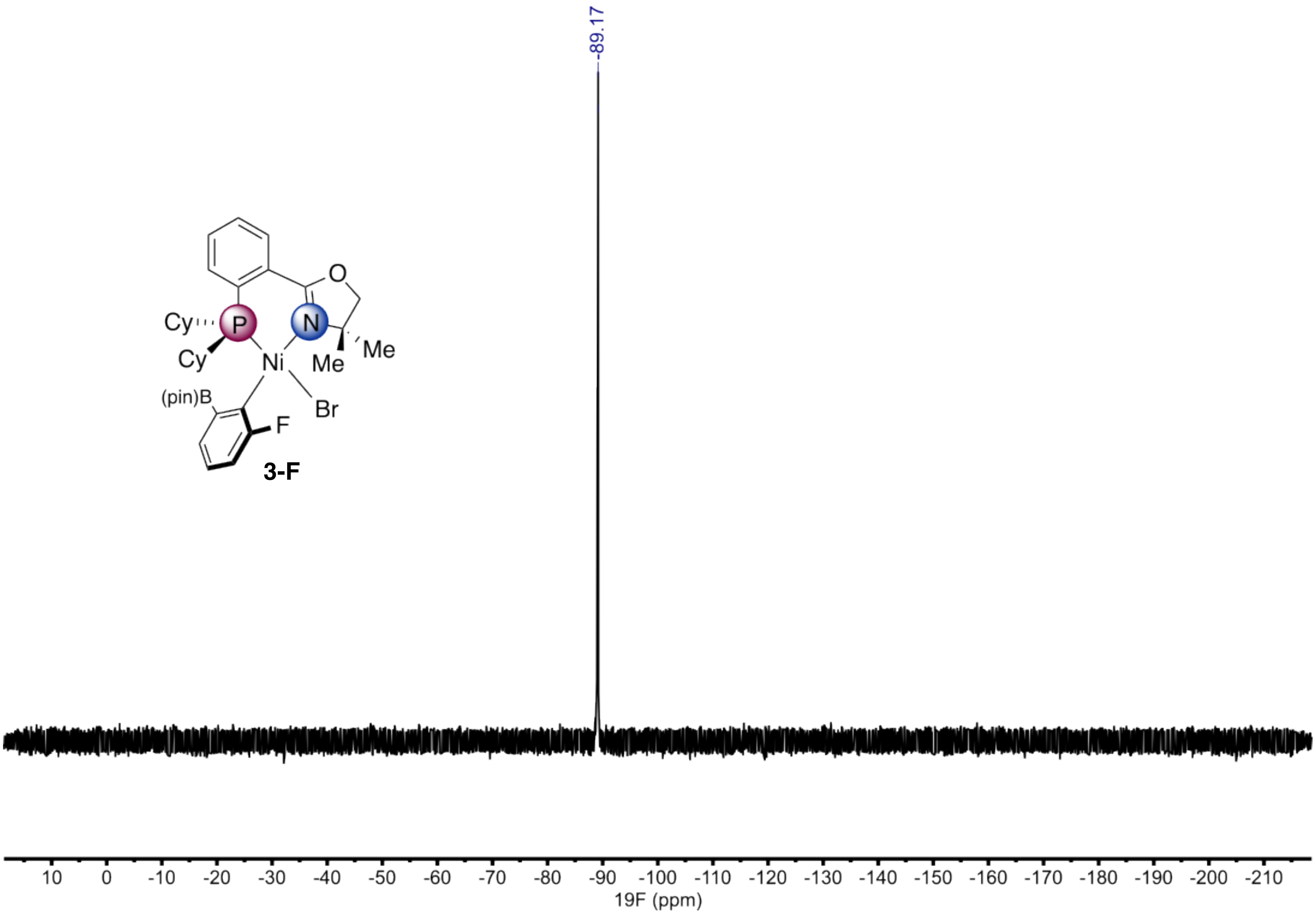

Figure S41. $^{19}F\{^{1}H\}$ NMR spectrum (376 MHz, THF–d_8, 298 K) of **3-F**.

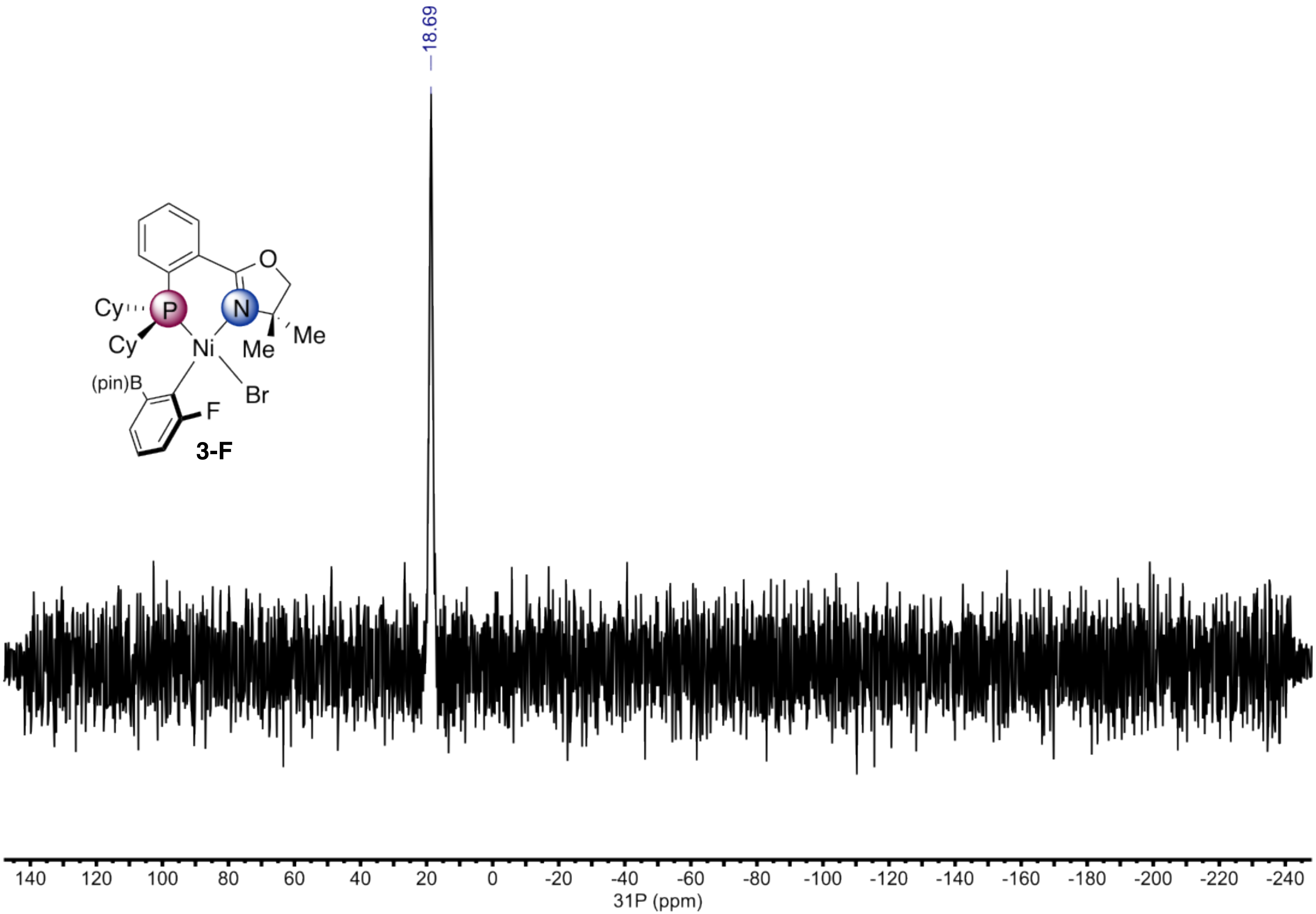

Figure S42. $^{31}P\{^1H\}$ NMR spectrum (162 MHz, THF–d_8, 298 K) of **3-F**.

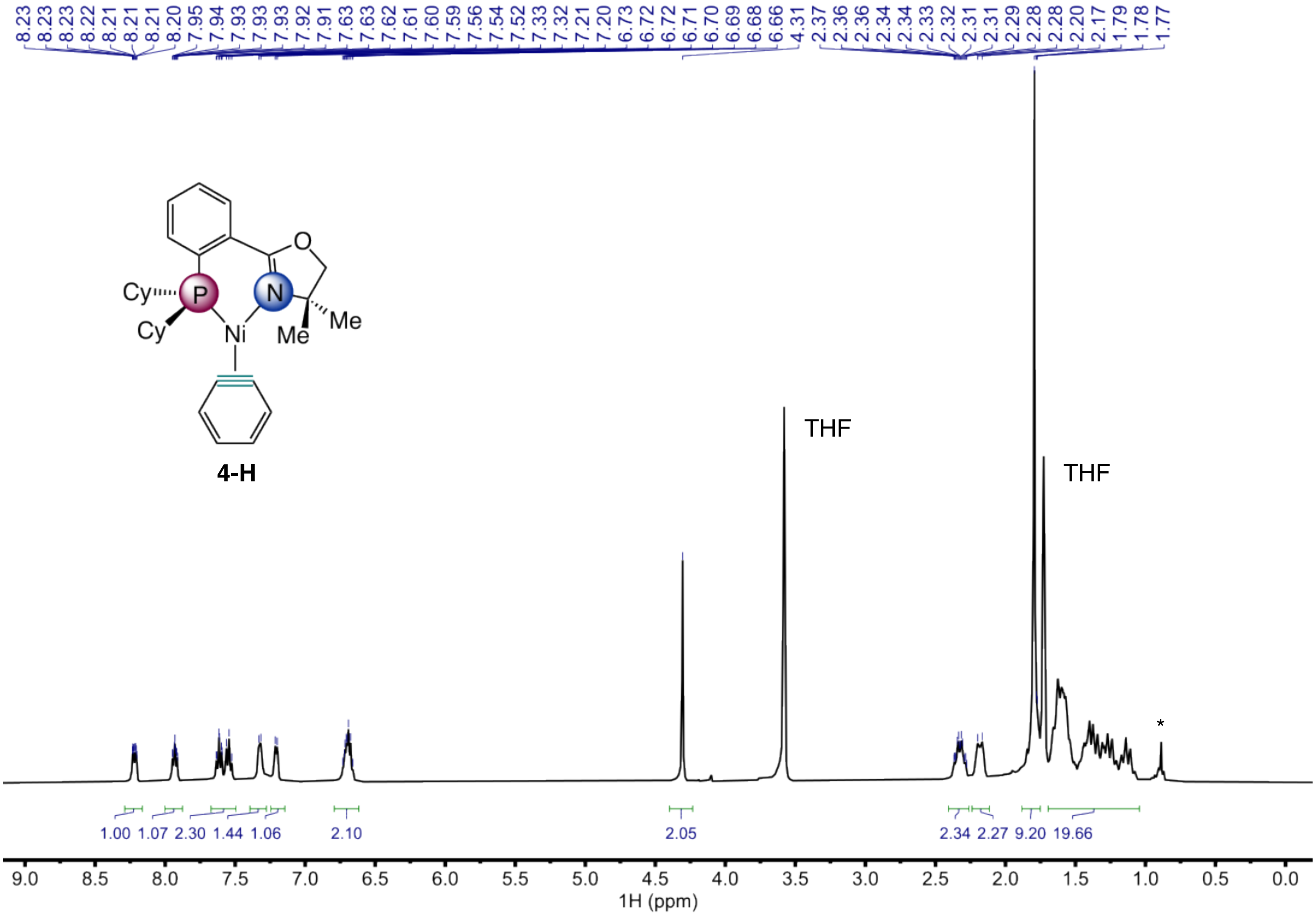

Figure S43. ^{1}H NMR spectrum (400 MHz, THF–d_8, 298 K) spectrum of **4-H**. *residual pentane

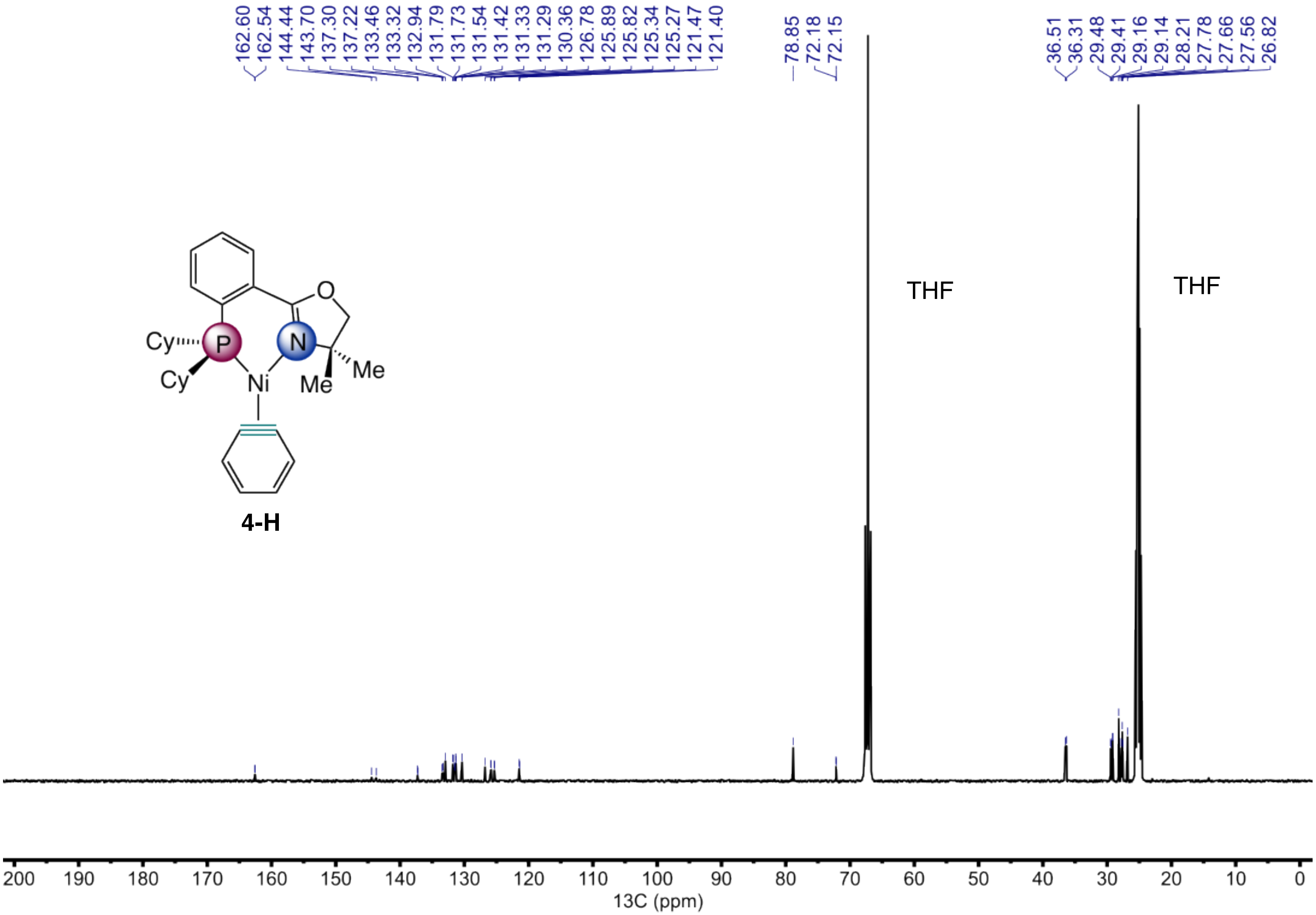

Figure S44. $^{13}C\{^{1}H\}$ NMR spectrum (101 MHz, THF–d_8, 298 K) spectrum of **4-H**.

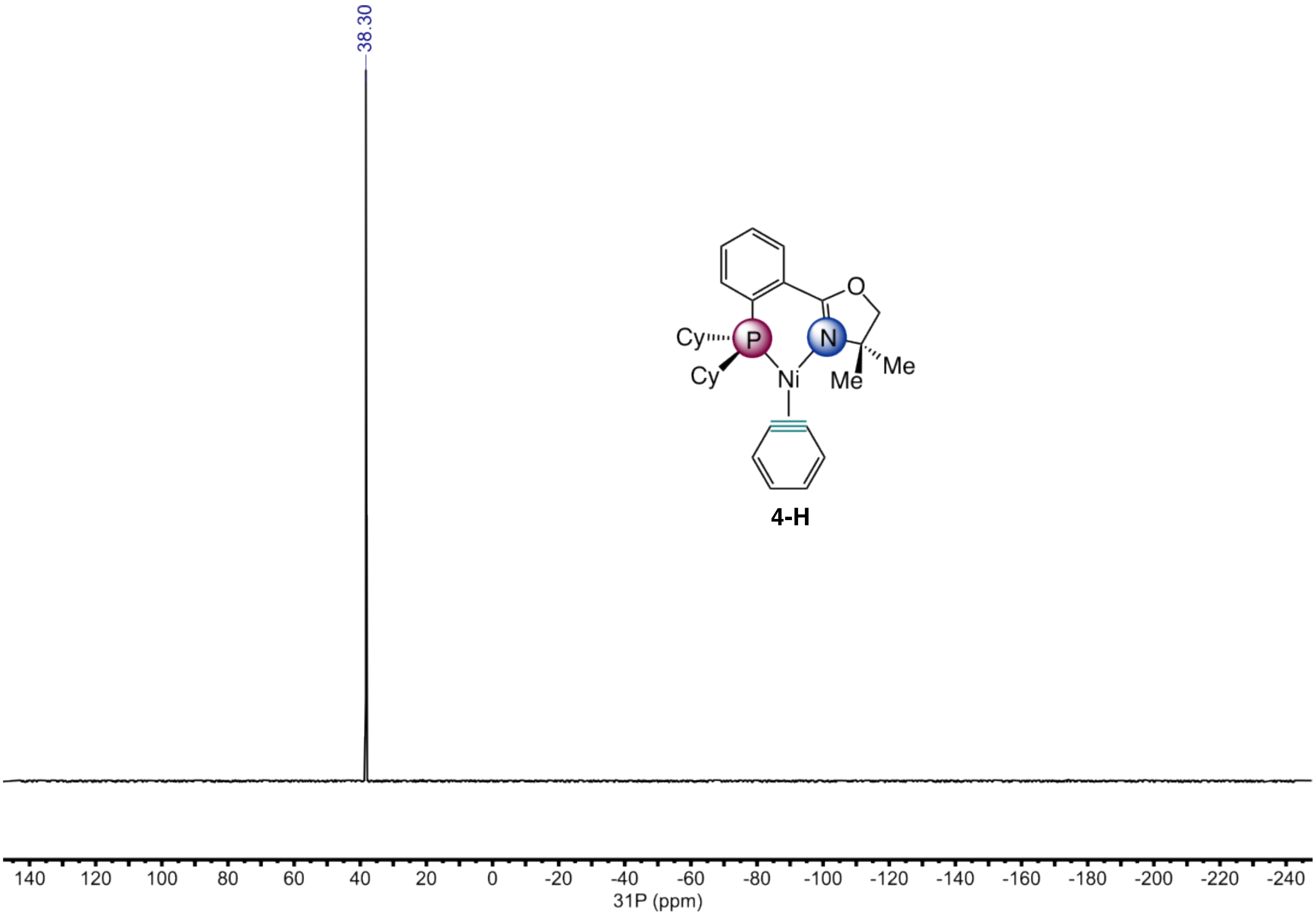

Figure S45. $^{31}P\{^{1}H\}$ NMR spectrum (162 MHz, THF–d_8, 298 K) spectrum of **4-H**.

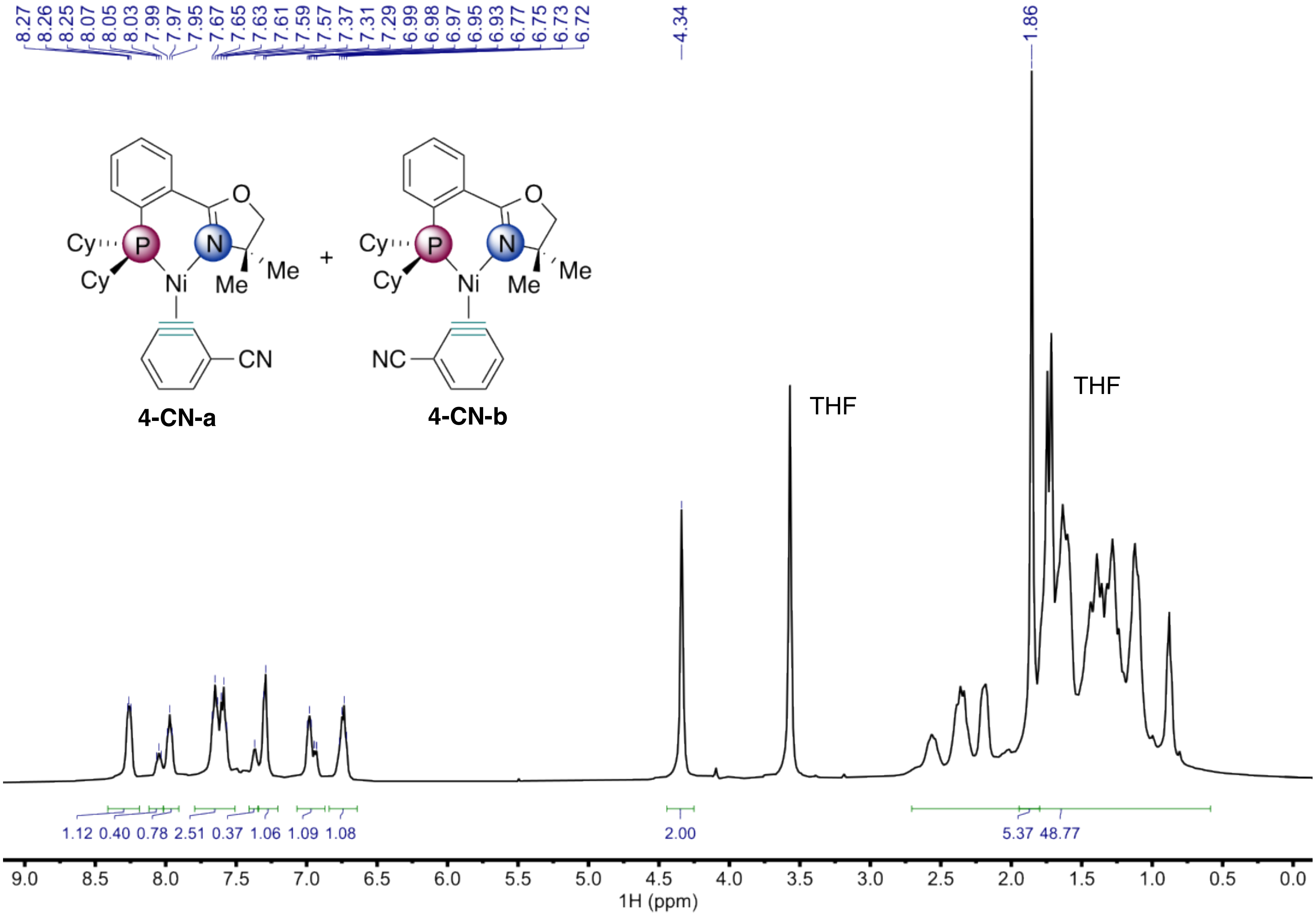

Figure S46. ^{1}H NMR spectrum (400 MHz, THF–d_8, 298 K) spectrum of **4-CN**.

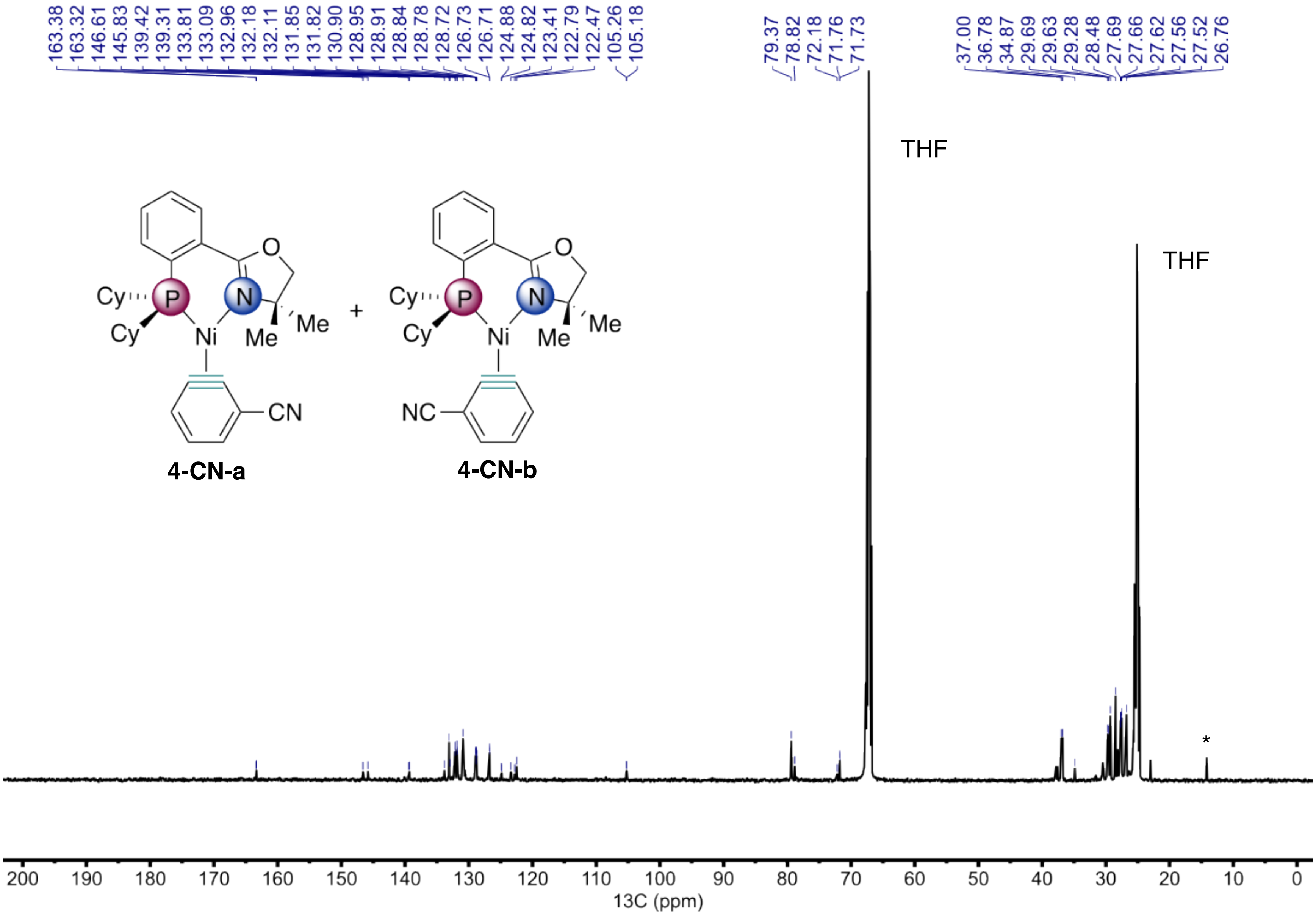

Figure S47. $^{13}C\{^{1}H\}$ NMR spectrum (101 MHz, THF–d_8, 298 K) spectrum of **4-CN**. *residual pentane.

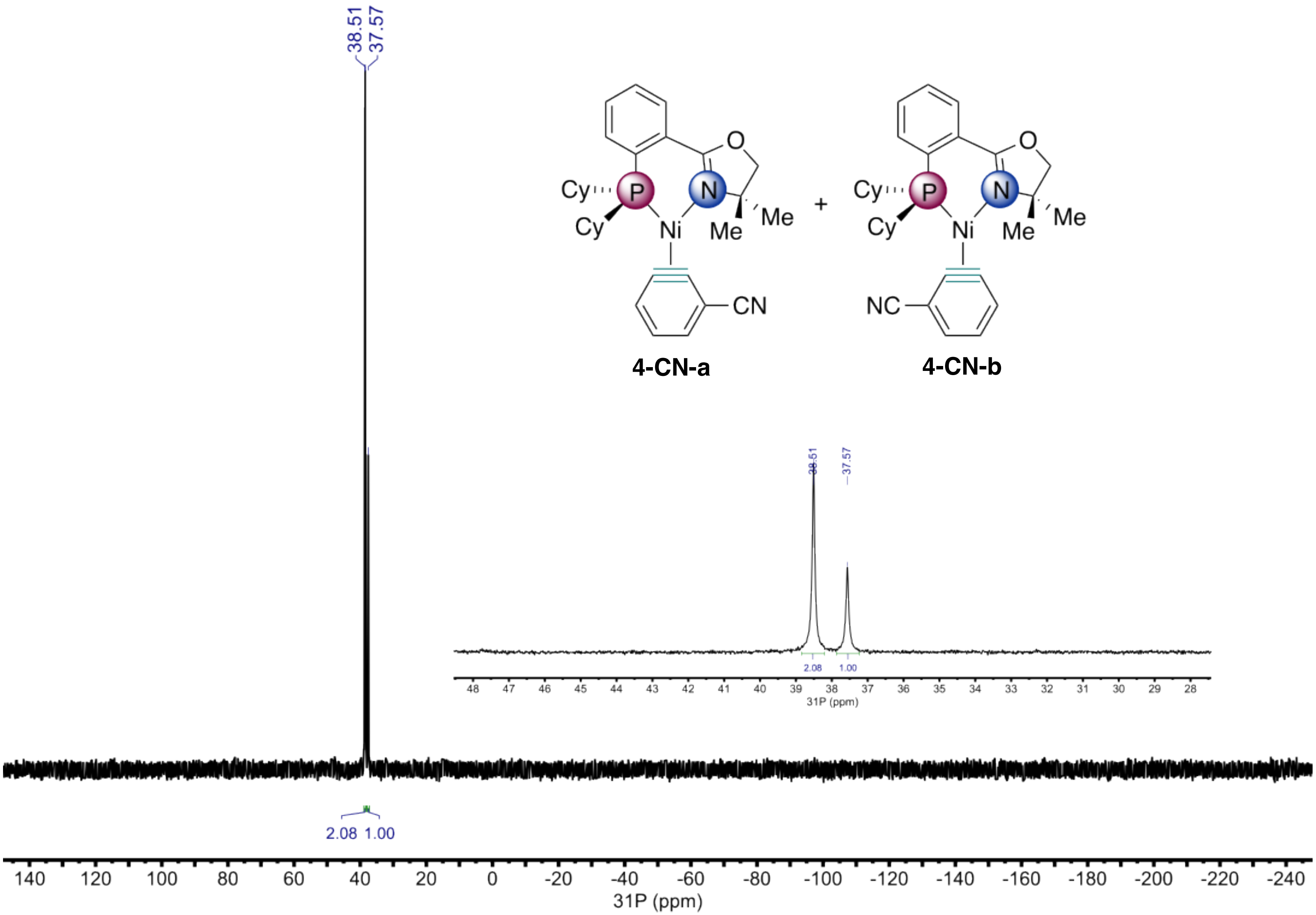

Figure S48. $^{31}P\{^1H\}$ NMR spectrum (162 MHz, THF–d_8, 298 K) of **4-CN**. d_1 = 25.4 s based on 5*T_1 for T_1 = 3.70, **5.08 s**.

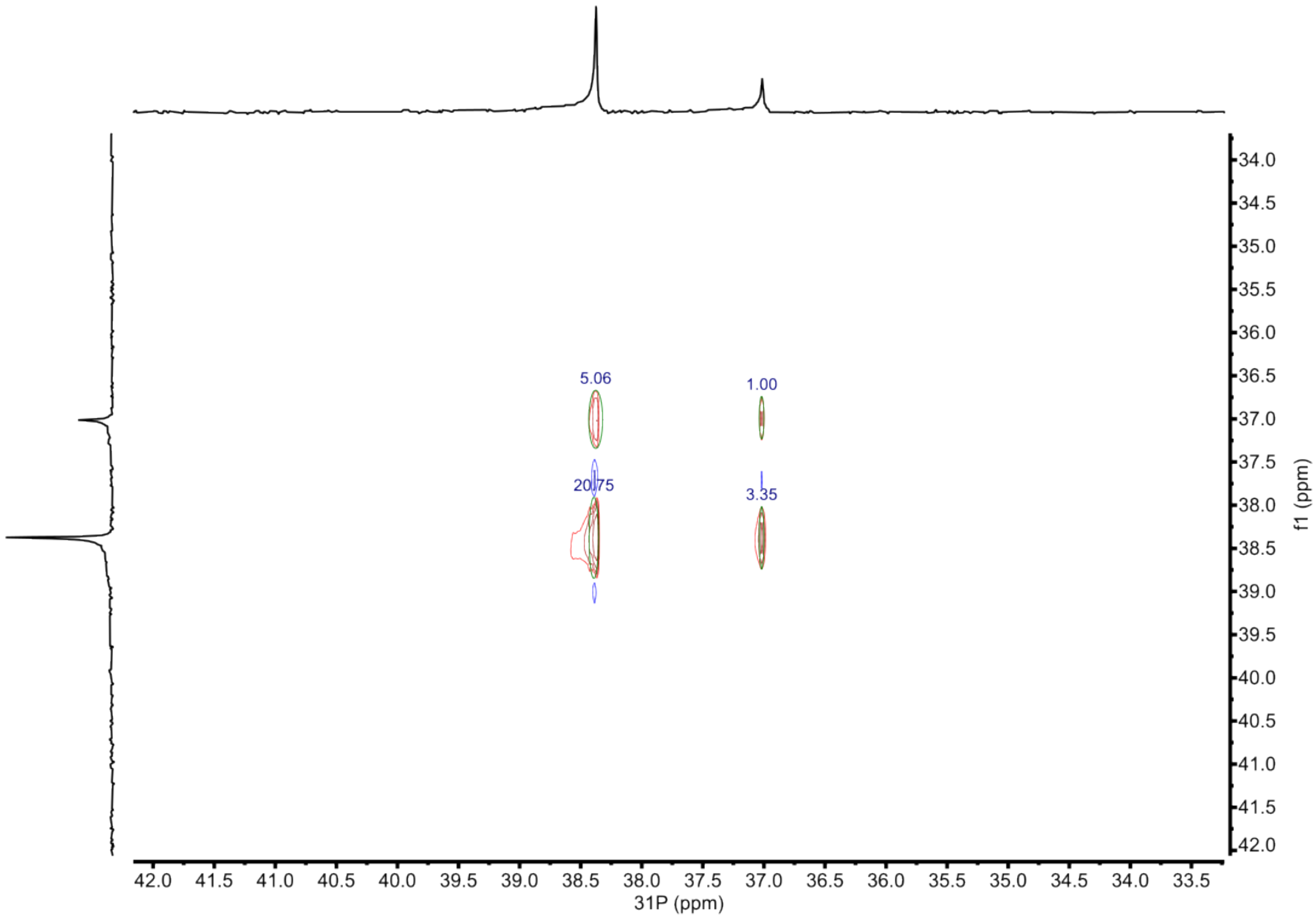

Figure S49. 2D $^{31}P\{^{1}H\}$–$^{31}P\{^{1}H\}$ NOESY spectrum (162 MHz, THF–d_8, 298 K) of **4-CN**. d_1 = 25.4 s, d_8 = 2.5 s.

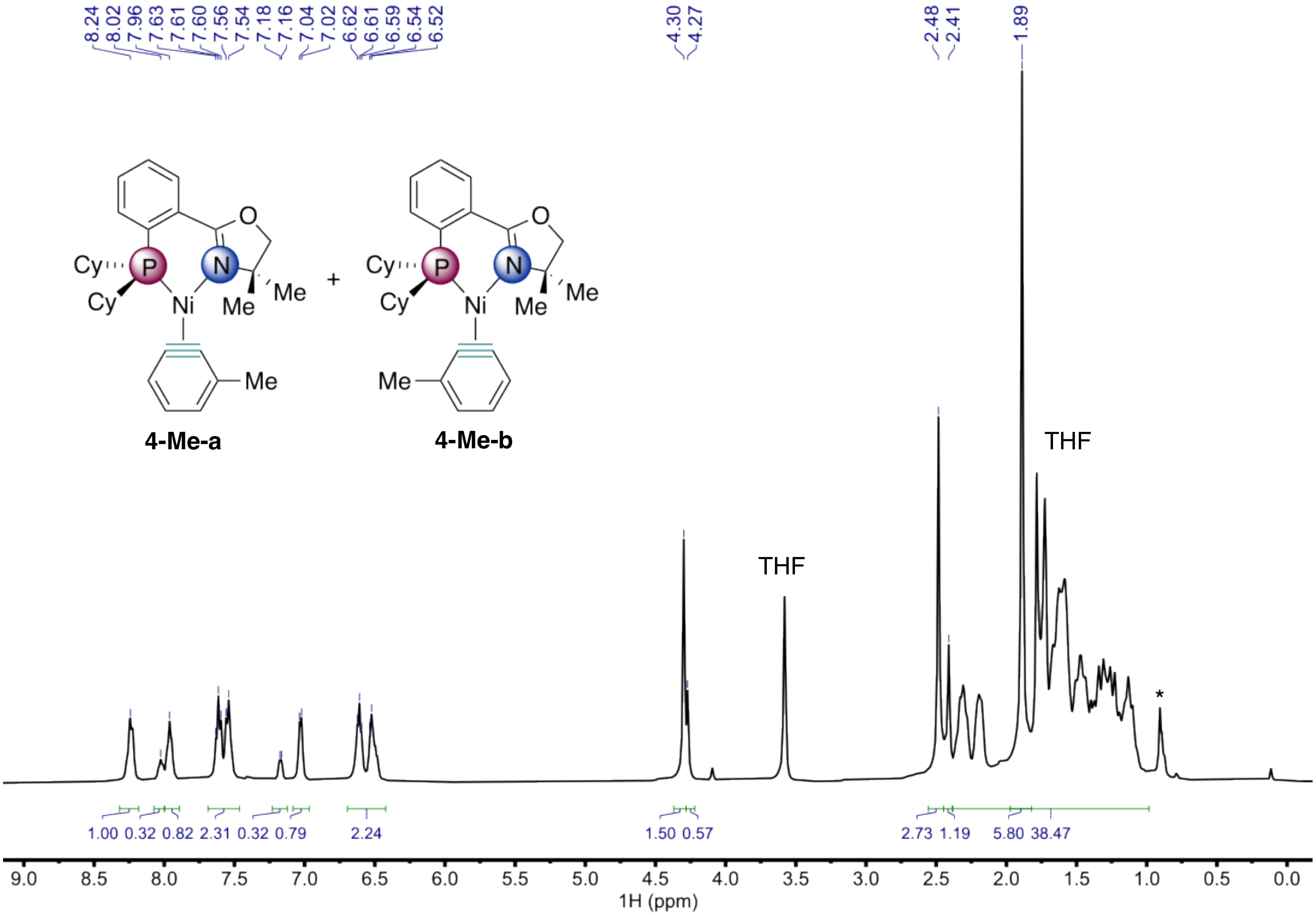

Figure S50. ^{1}H NMR spectrum (400 MHz, THF–d_8, 298 K) spectrum of **4-Me**. *residual pentane

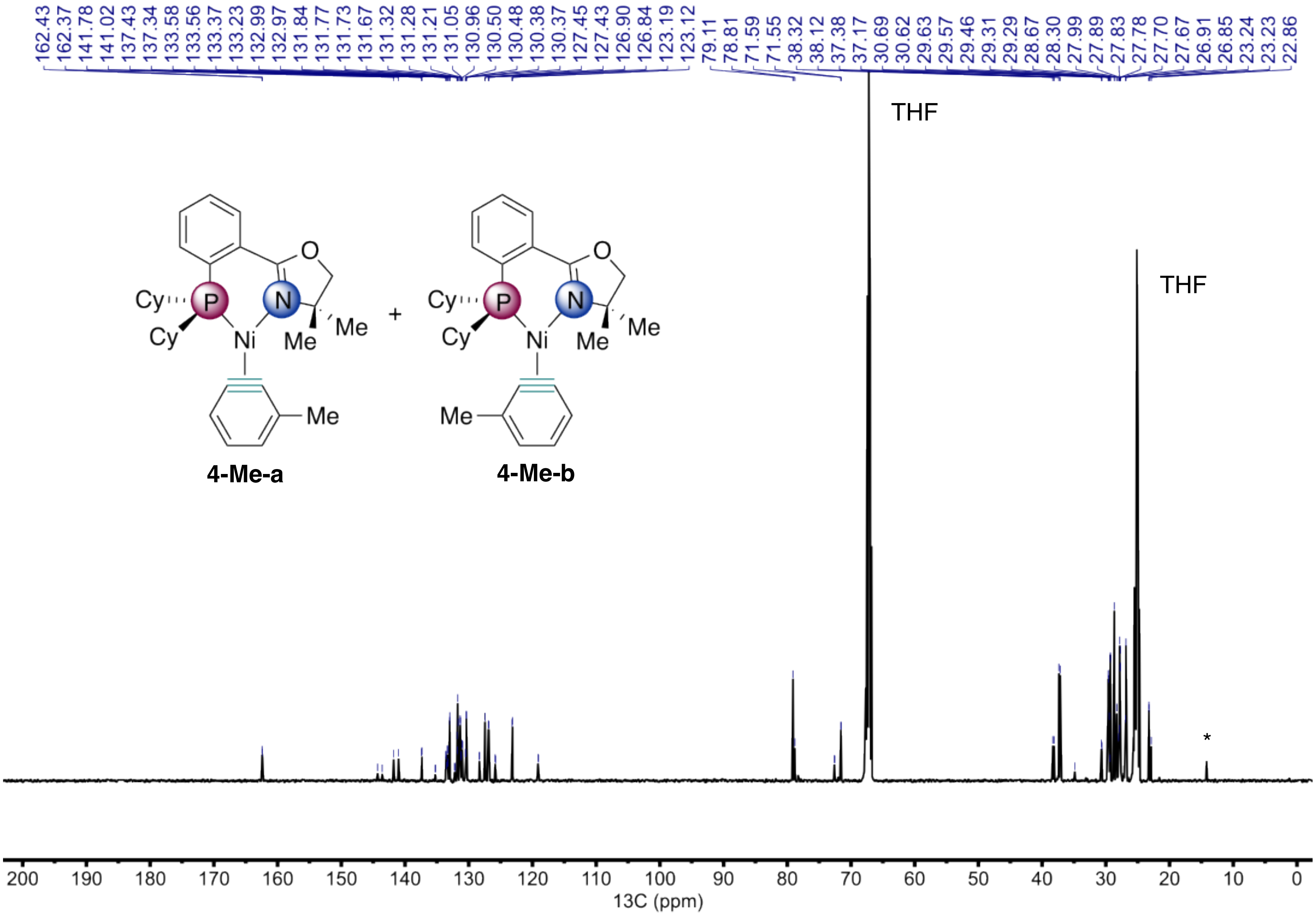

Figure S51. $^{13}C\{^{1}H\}$ NMR spectrum (101 MHz, THF–d_8, 298 K) of **4-Me.** *residual pentane

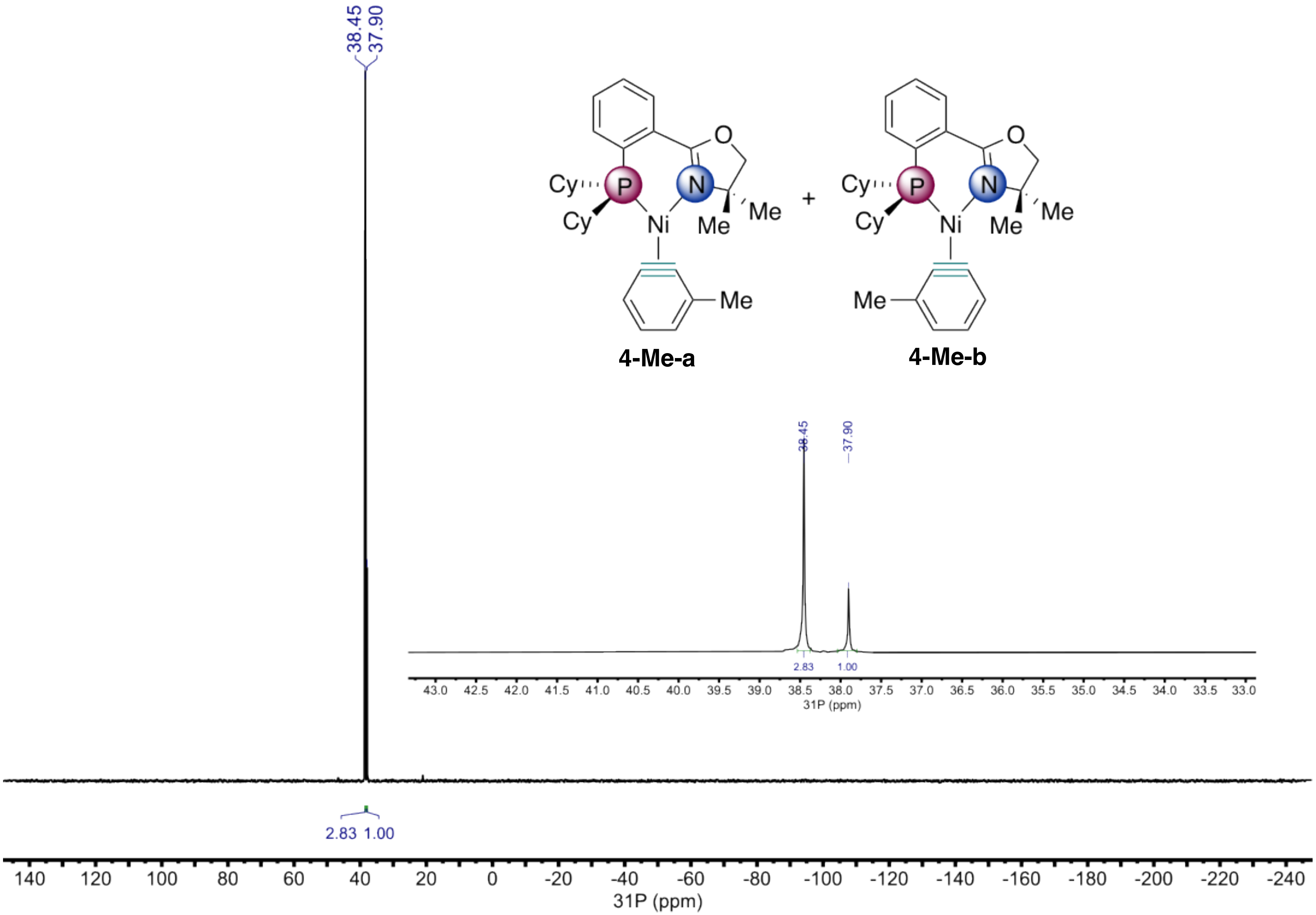

Figure S52. ^{31}P{^{1}H} NMR spectrum (162 MHz, THF–d_8, 298 K) of **4-Me**. d_1 = 20.55 s based on 5*T_1 for T_1 = 3.99, **4.11 s**.

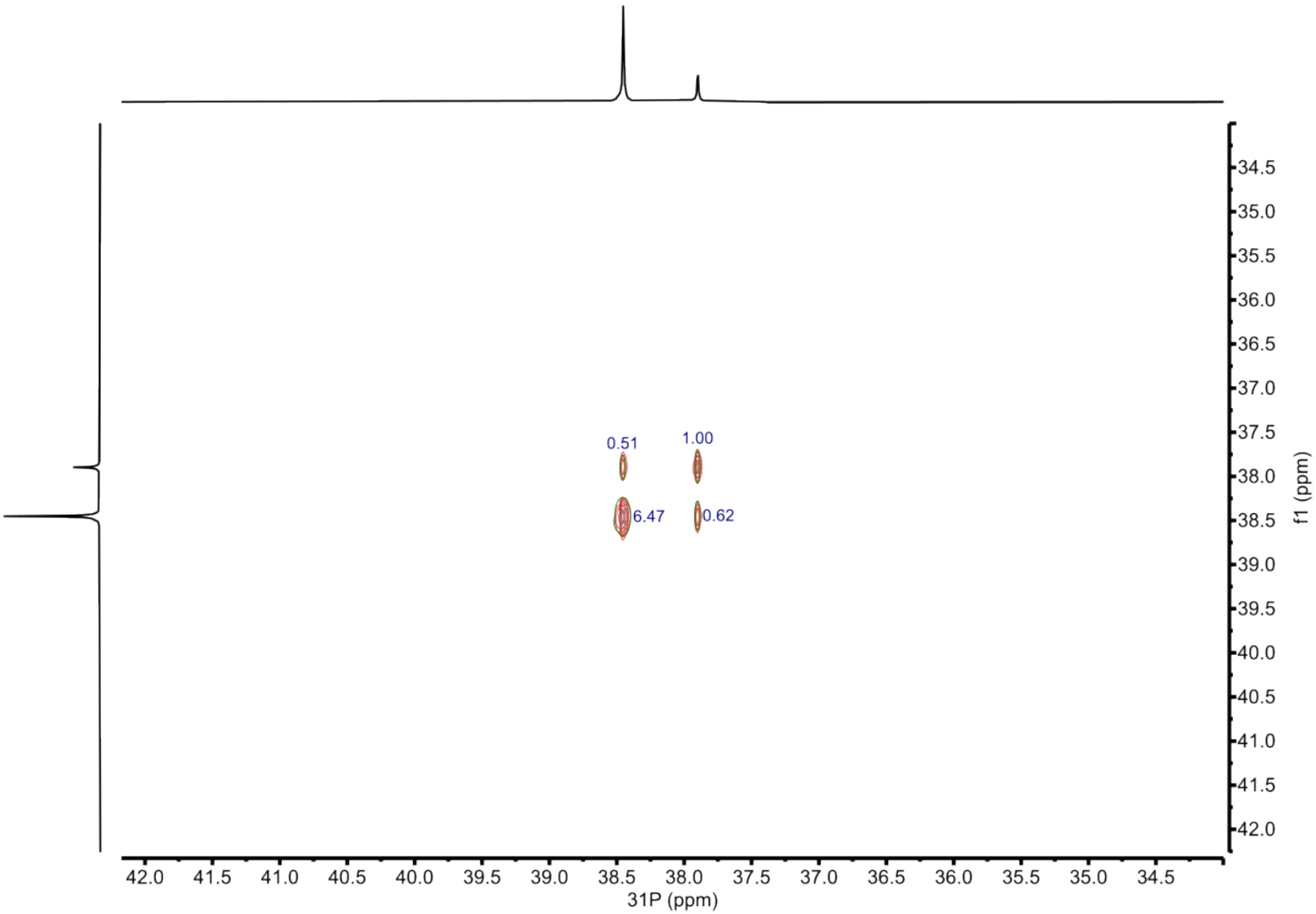

Figure S53. 2D $^{31}P\{^1H\}$–$^{31}P\{^1H\}$ NOESY spectrum (162 MHz, THF–d_8, 298 K) of **4-Me**. d_1 = 20.55 s, d_8 = 2.5 s.

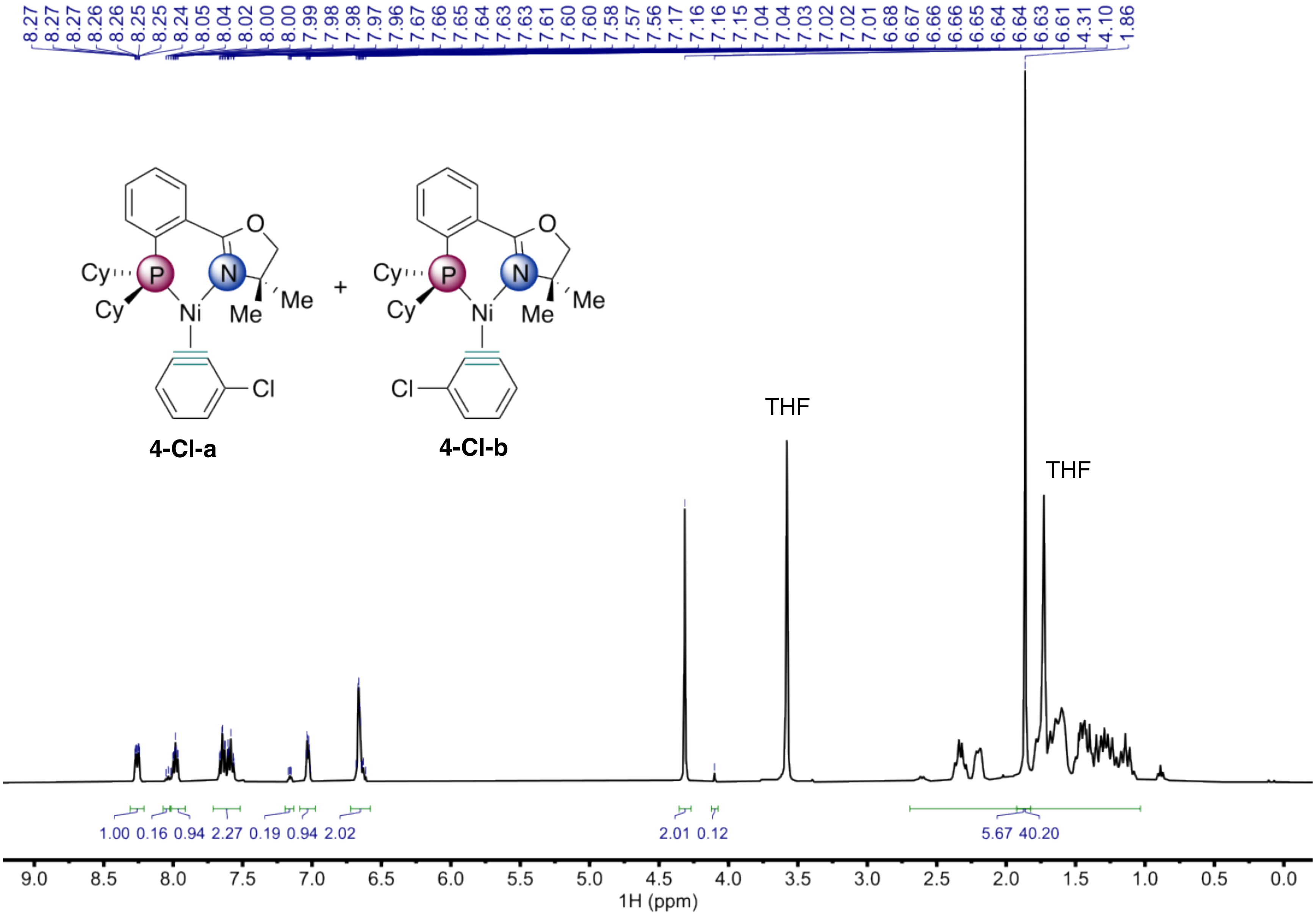

Figure S54. ^{1}H NMR spectrum (400 MHz, THF–d_8, 298 K) of **4-Cl**.

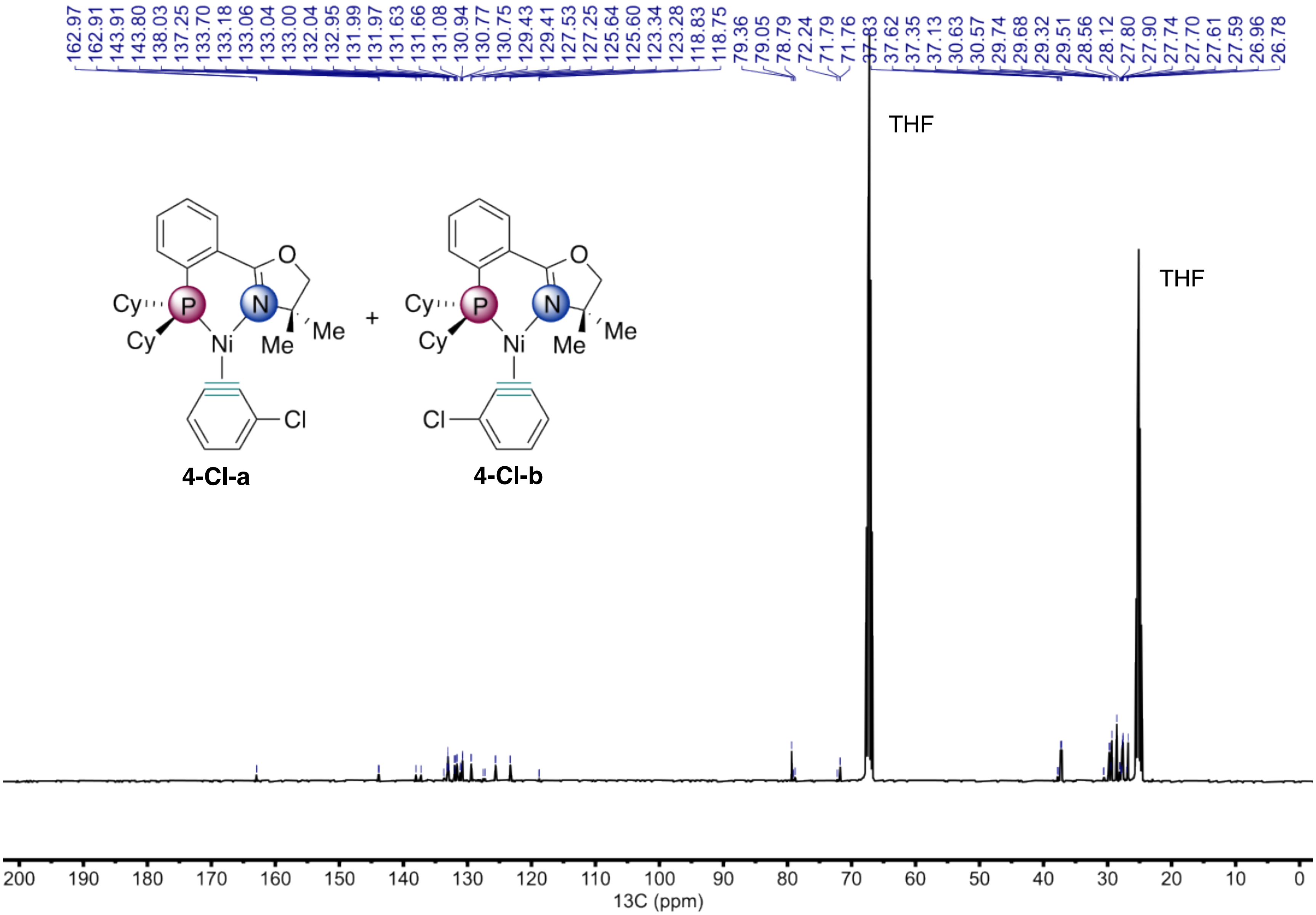

Figure S55. $^{13}C\{^{1}H\}$ NMR spectrum (101 MHz, THF–d_8, 298 K) of **4-Cl.**

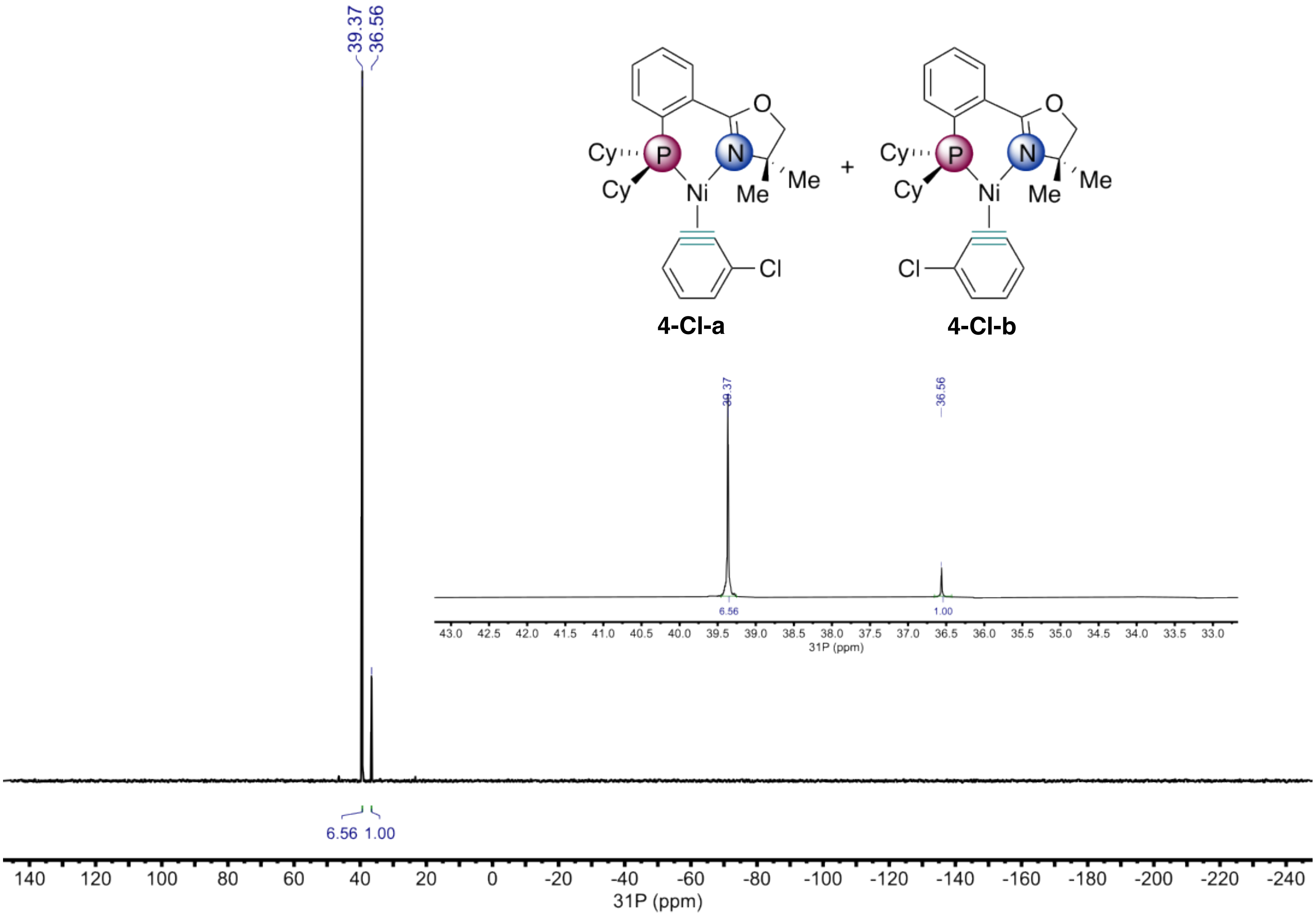

Figure S56. $^{31}P\{^{1}H\}$ NMR spectrum (162 MHz, THF–d_8, 298 K) of **4-Cl**. d_1 = 22.46 s based on 5*T_1 for T_1 = **4.49**, 4.39 s.

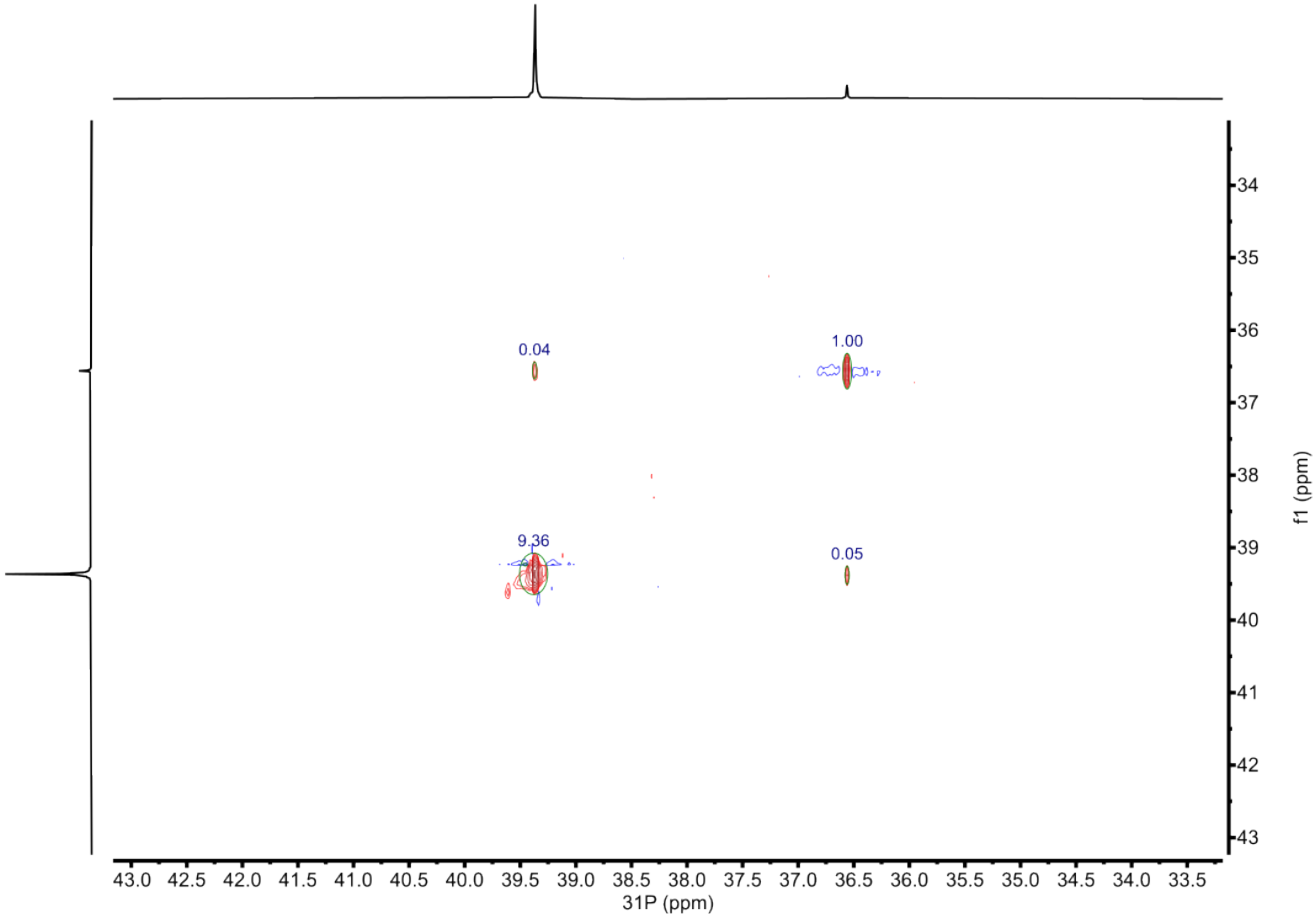

Figure S57. 2D $^{31}P\{^1H\}$–$^{31}P\{^1H\}$ NOESY spectrum (162 MHz, THF–d_8, 298 K) of **4-Cl**. d_1 = 22.46 s, d_8 = 2.5 s.

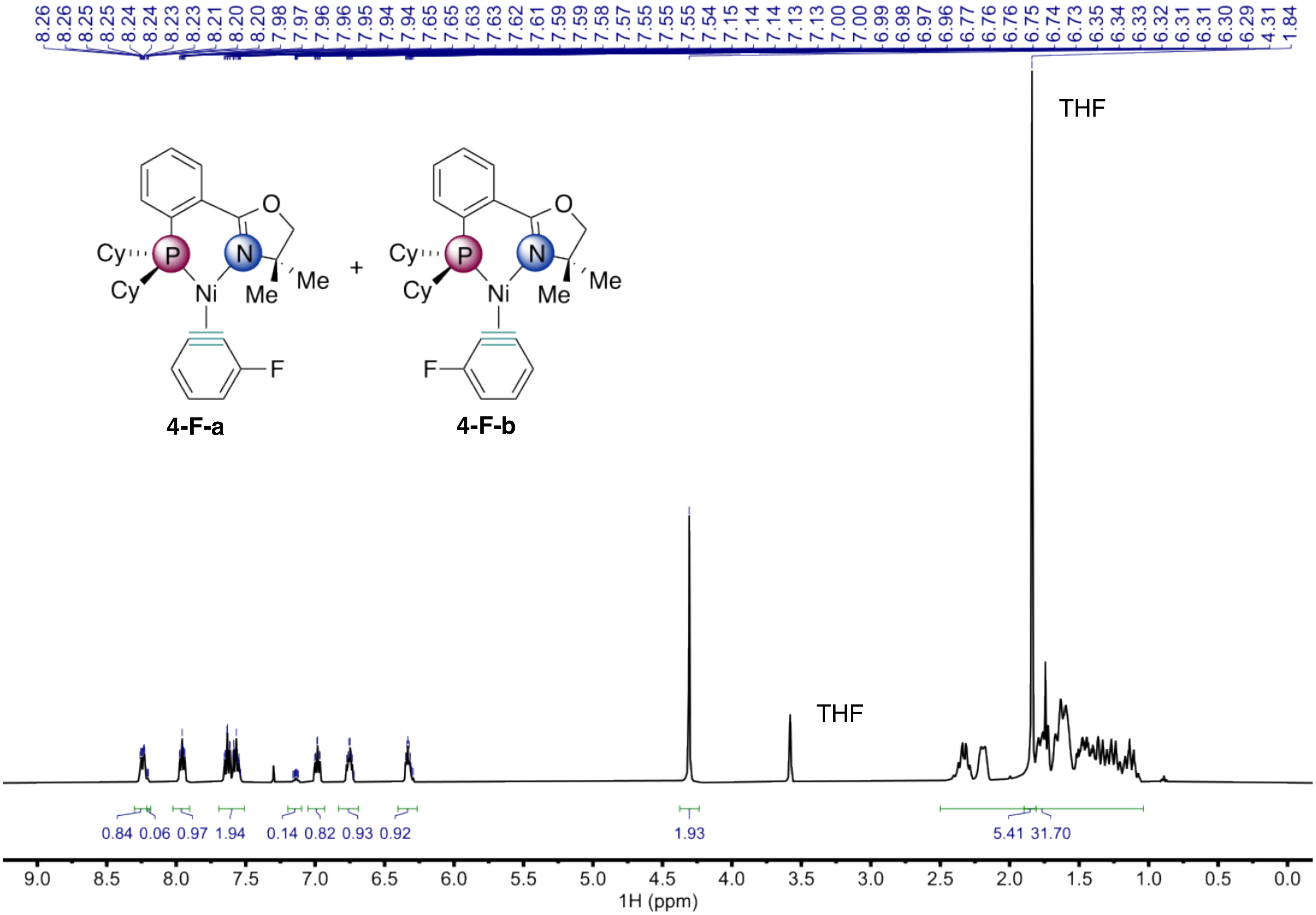

Figure S58. ^{1}H NMR spectrum (400 MHz, THF–d_8, 298 K) of **4-F**.

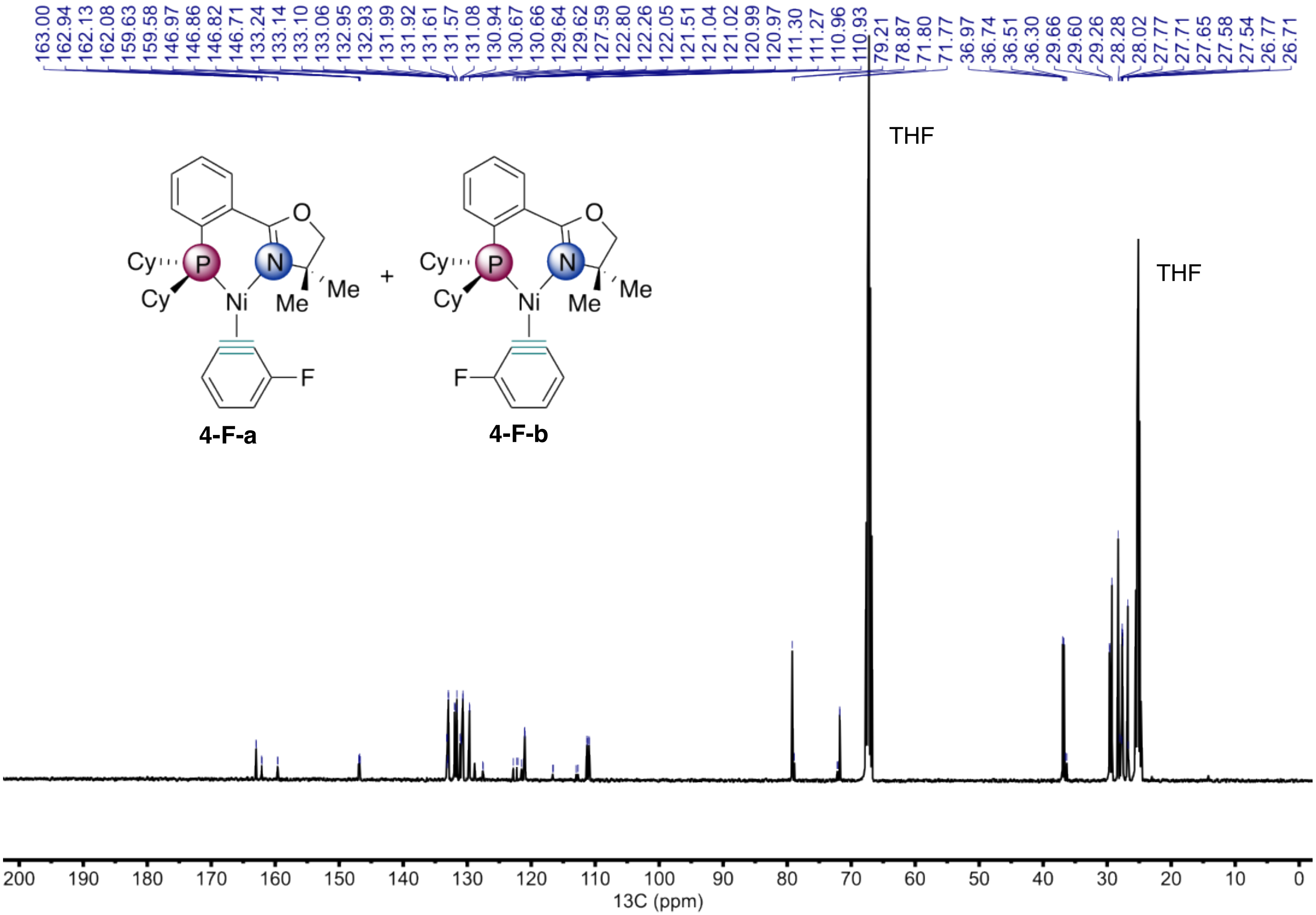

Figure S59. $^{13}C\{^{1}H\}$ NMR spectrum (101 MHz, THF–d_8, 298 K) of **4-F**.

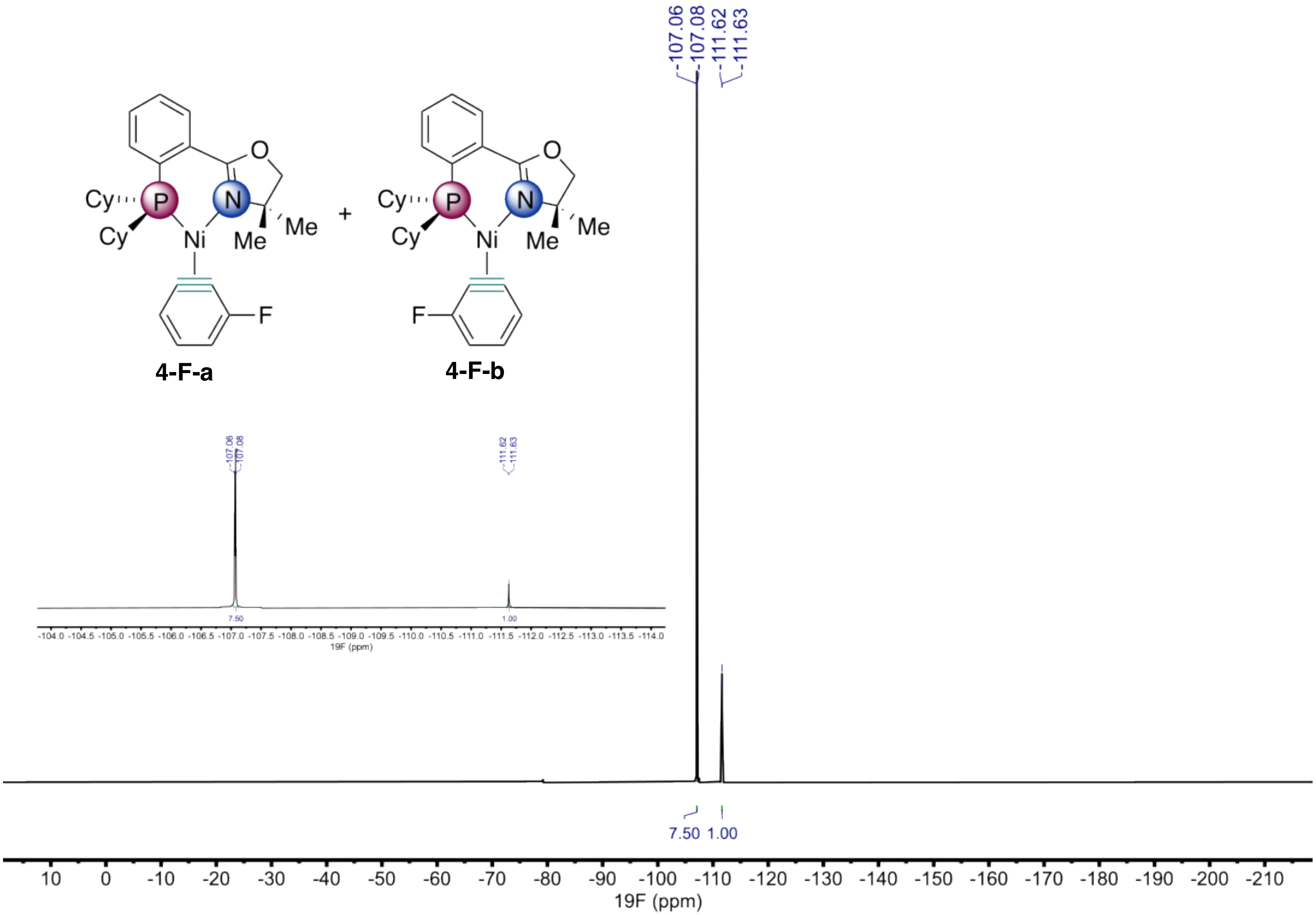

Figure S60. $^{19}F\{^{1}H\}$ NMR spectrum (376 MHz, THF–d_8, 298 K) of **4-F**.

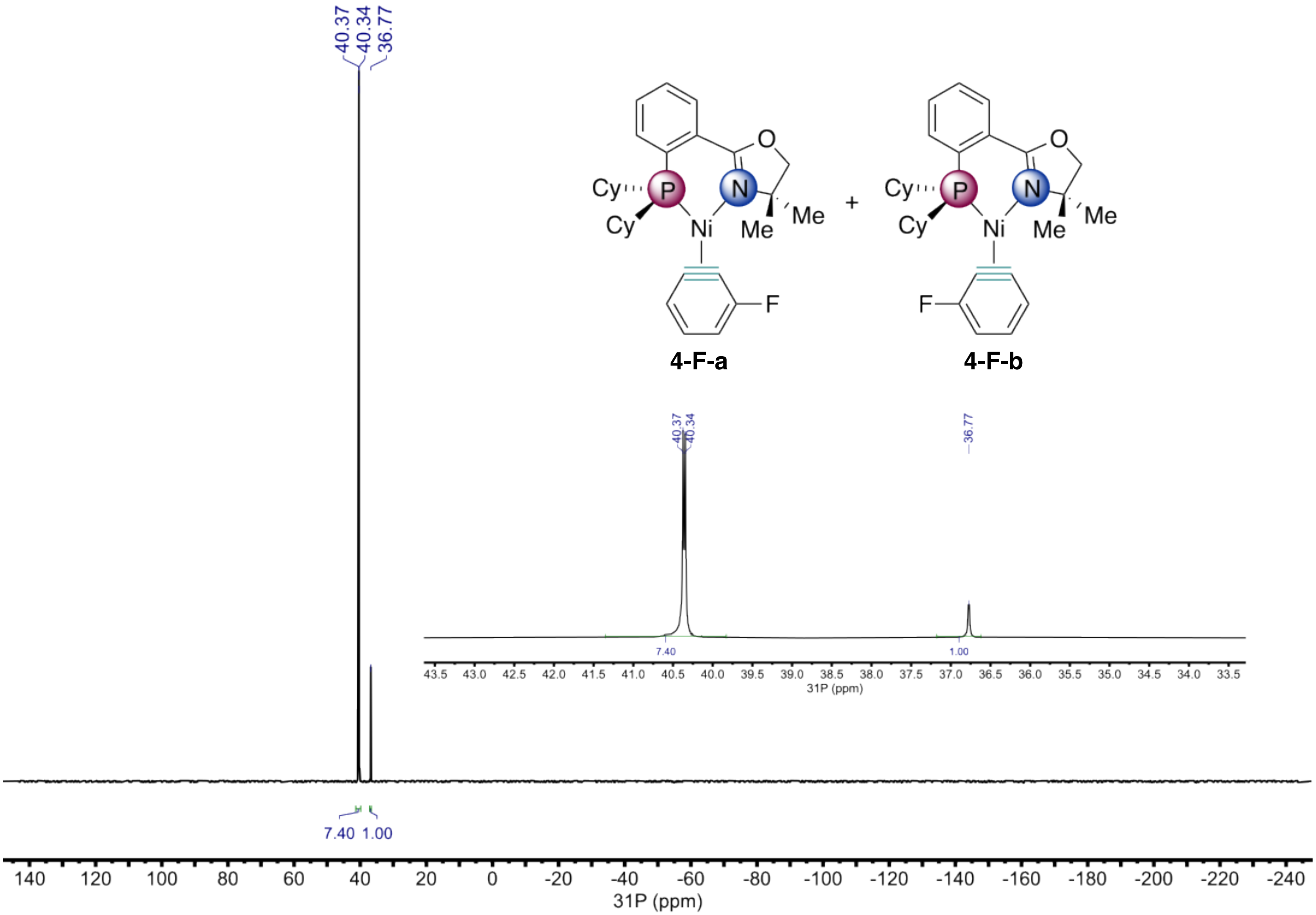

Figure S61. $^{31}P\{^{1}H\}$ NMR spectrum (162 MHz, THF–d_8, 298 K) of **4-F**. d_1 = 18.28 s based on 5*T_1 for T_1 = **3.66**, 3.20 s.

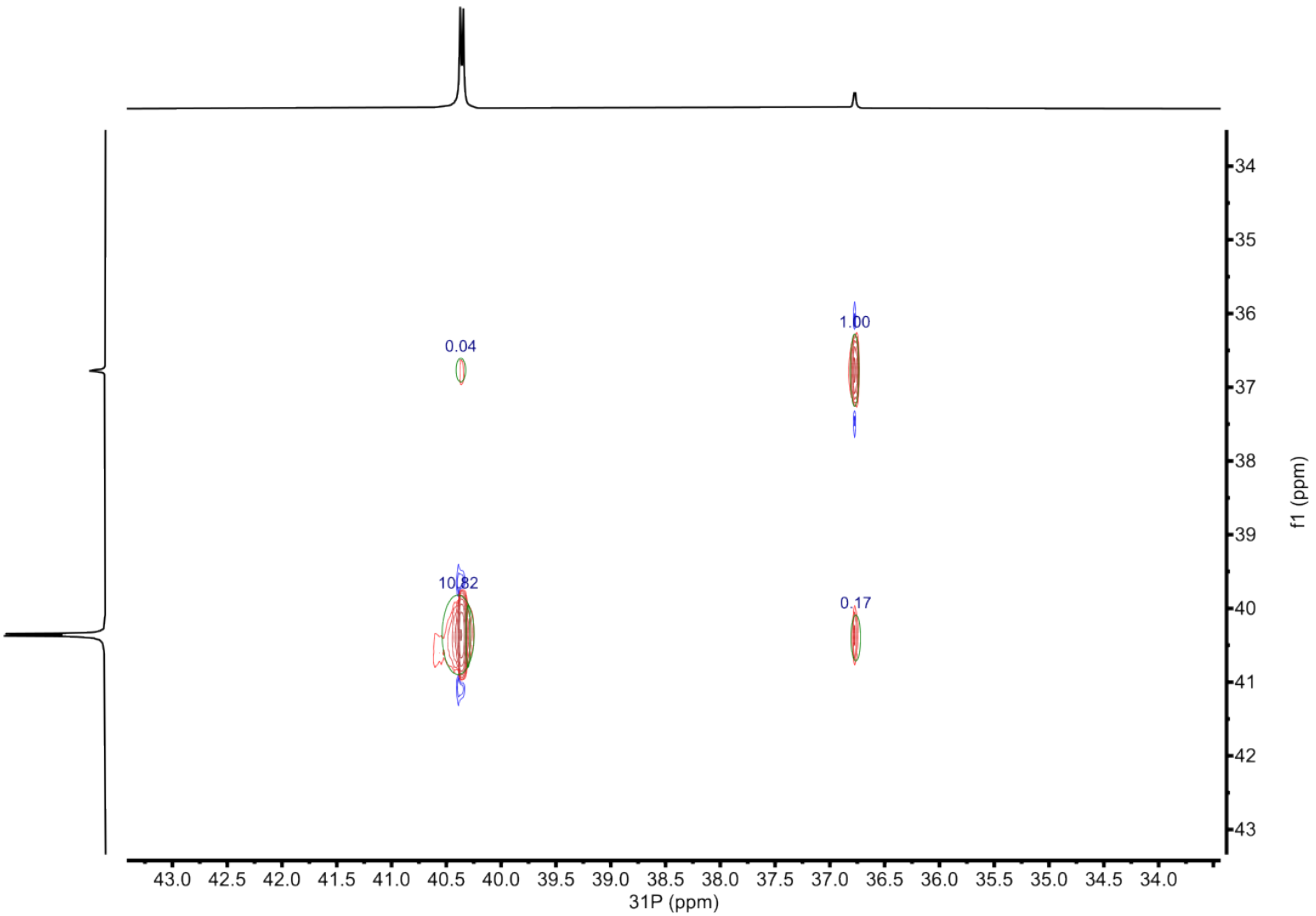

Figure S62. 2D $^{31}P\{^{1}H\}$–$^{31}P\{^{1}H\}$ NOESY spectrum (162 MHz, THF–d_8, 298 K) of **4-F**. d_1 = 18.28 s, d_8 = 2.5 s.

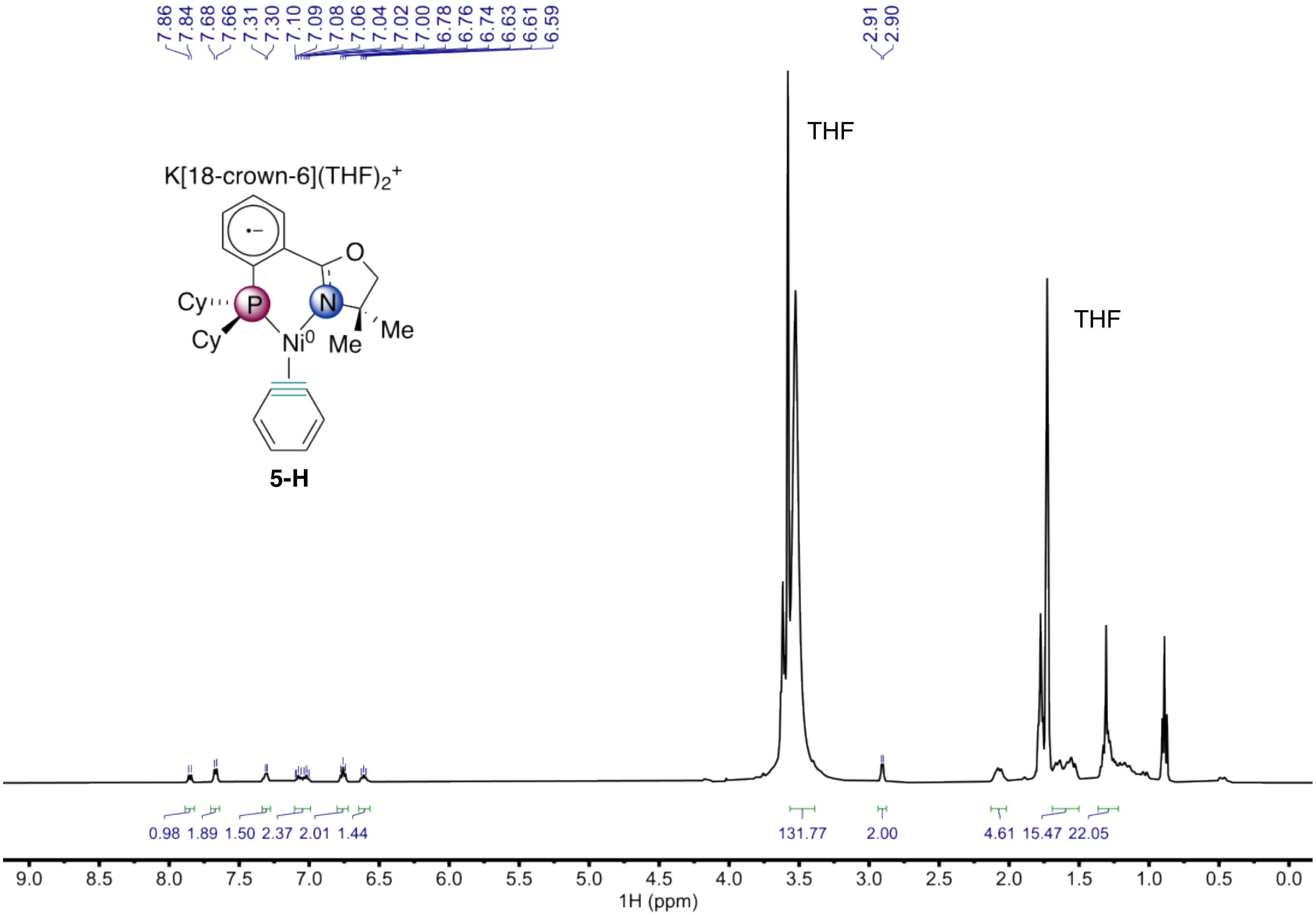

Figure S63. ^{1}H NMR spectrum (400 MHz, THF–d_8, 298 K) of **5-H**.

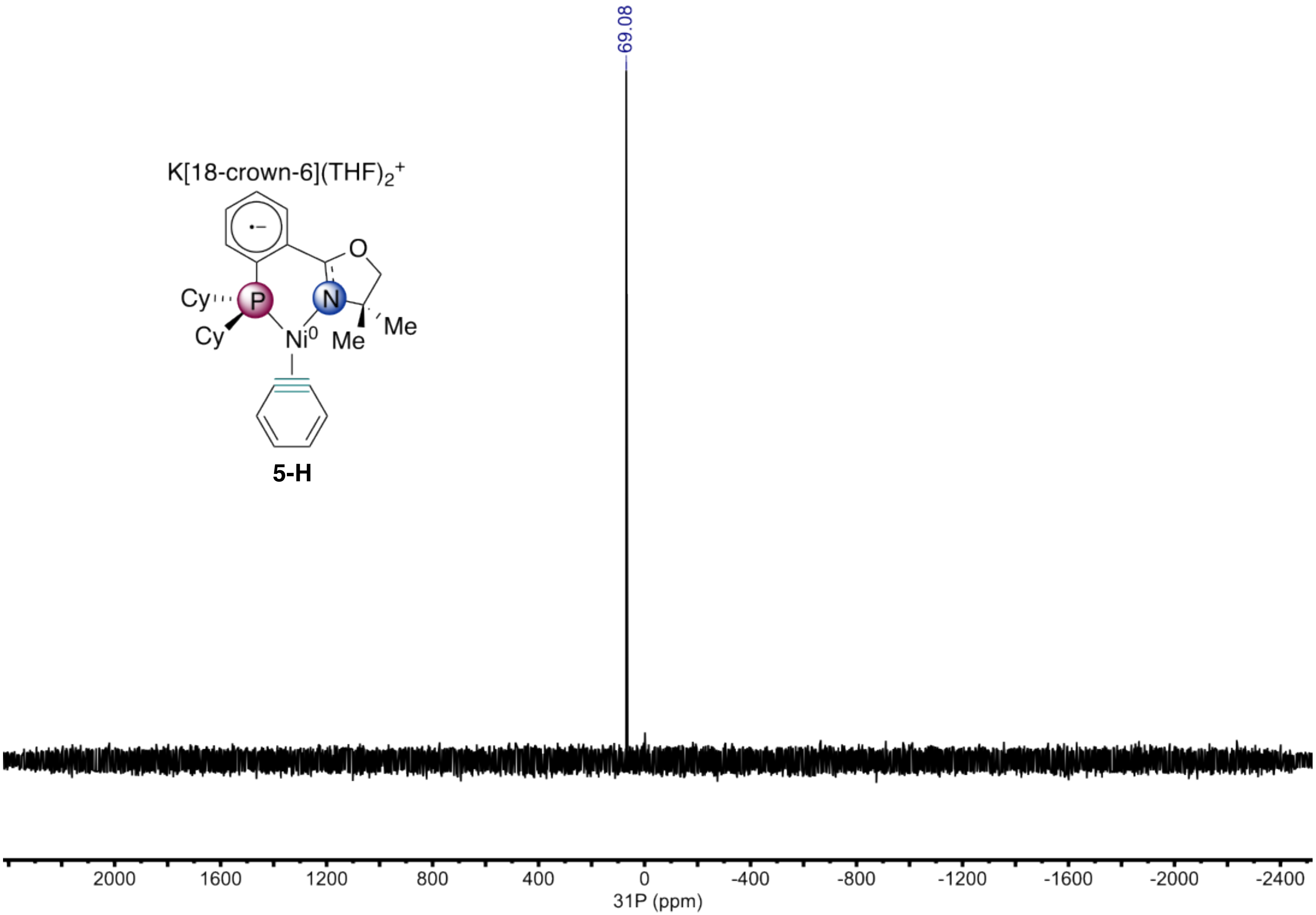

Figure S64. ${}^{31}P\{{}^{1}H\}$ NMR spectrum (162 MHz, THF–d_8, 298 K) of **5-H**.

NMR Data for 6-R-ab/Difunctionalizations of 4-R

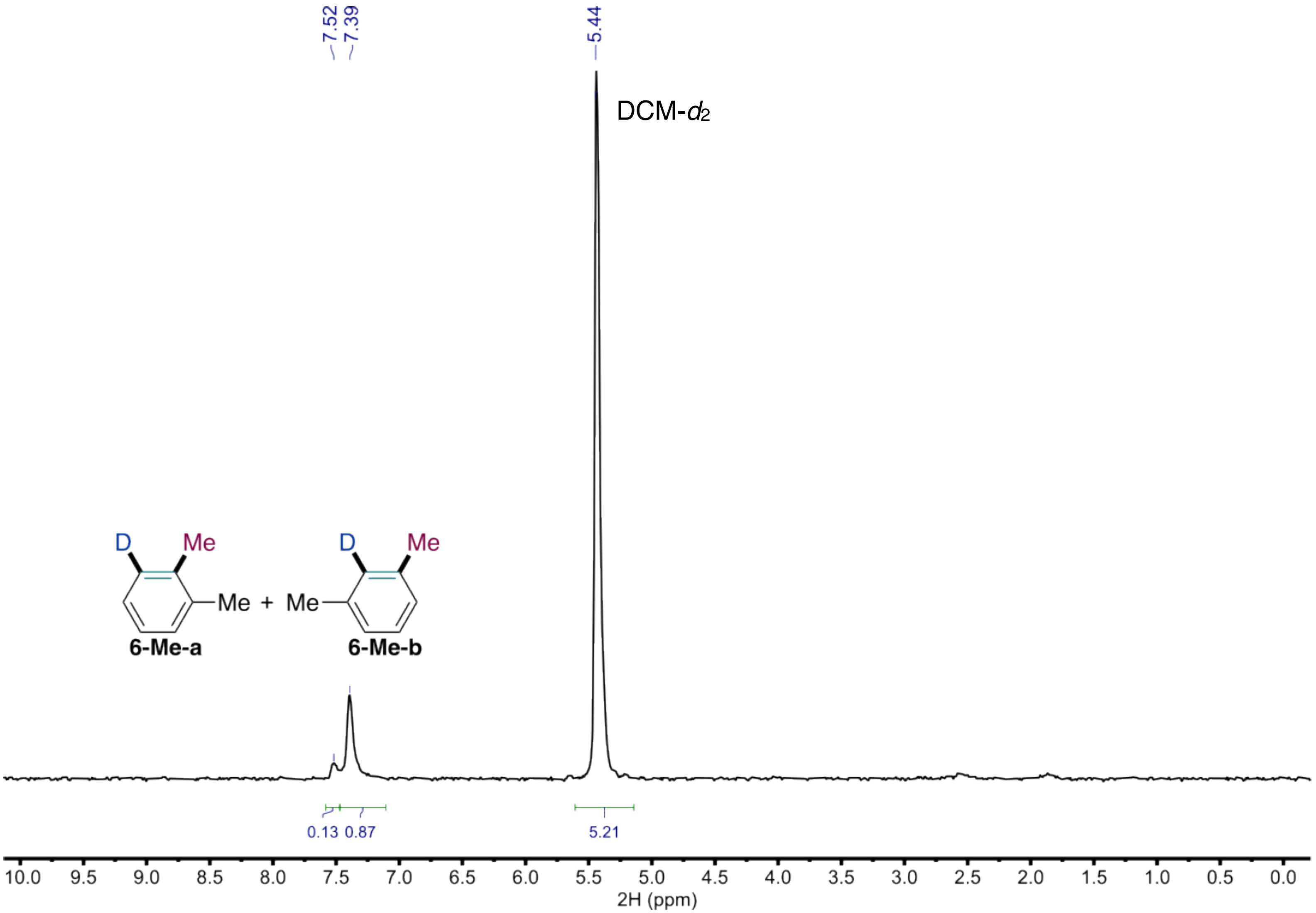

Figure S65: ^{2}H NMR spectrum (61 MHz, 1:1 PhMe:MeCN, 298 K) of difunctionalization of **4-Me** to furnish **6-Me-ab**. Whittaker Smoother baseline correction performed.

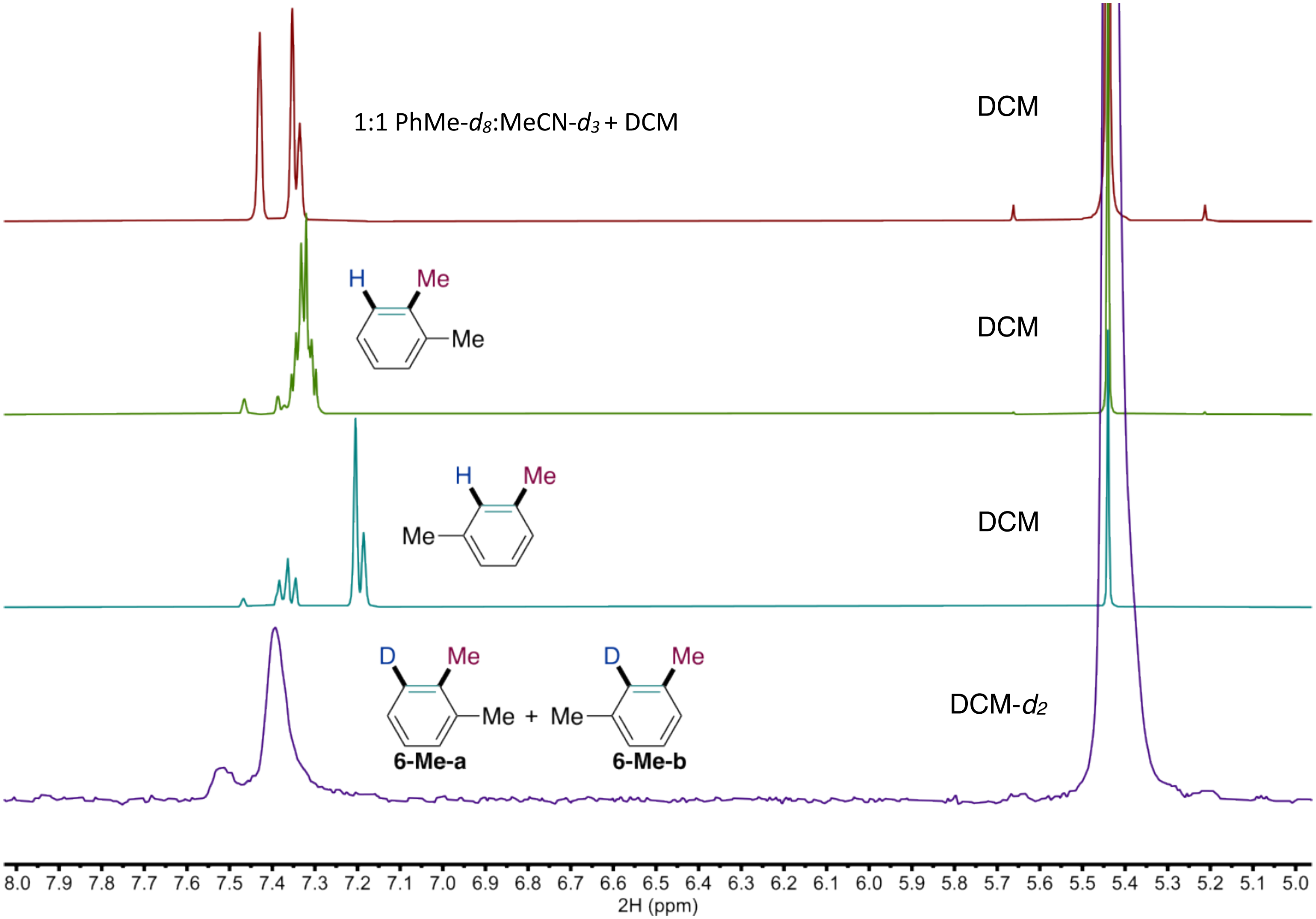

Figure S66: ^{1}H NMR spectra (400 MHz, 1:1 PhMe-d_8:MeCN-d_3, 298 K) of *o*-xylene and *m*-xylene with DCM as an internal reference and ^{2}H NMR spectrum (61 MHz, 1:1 PhMe:MeCN, 298 K) of **6-Me-ab** with DCM-d_2 as an internal reference. Spectra are referenced to the literature value for DCM in MeCN-d_3 (5.44 ppm).[8]

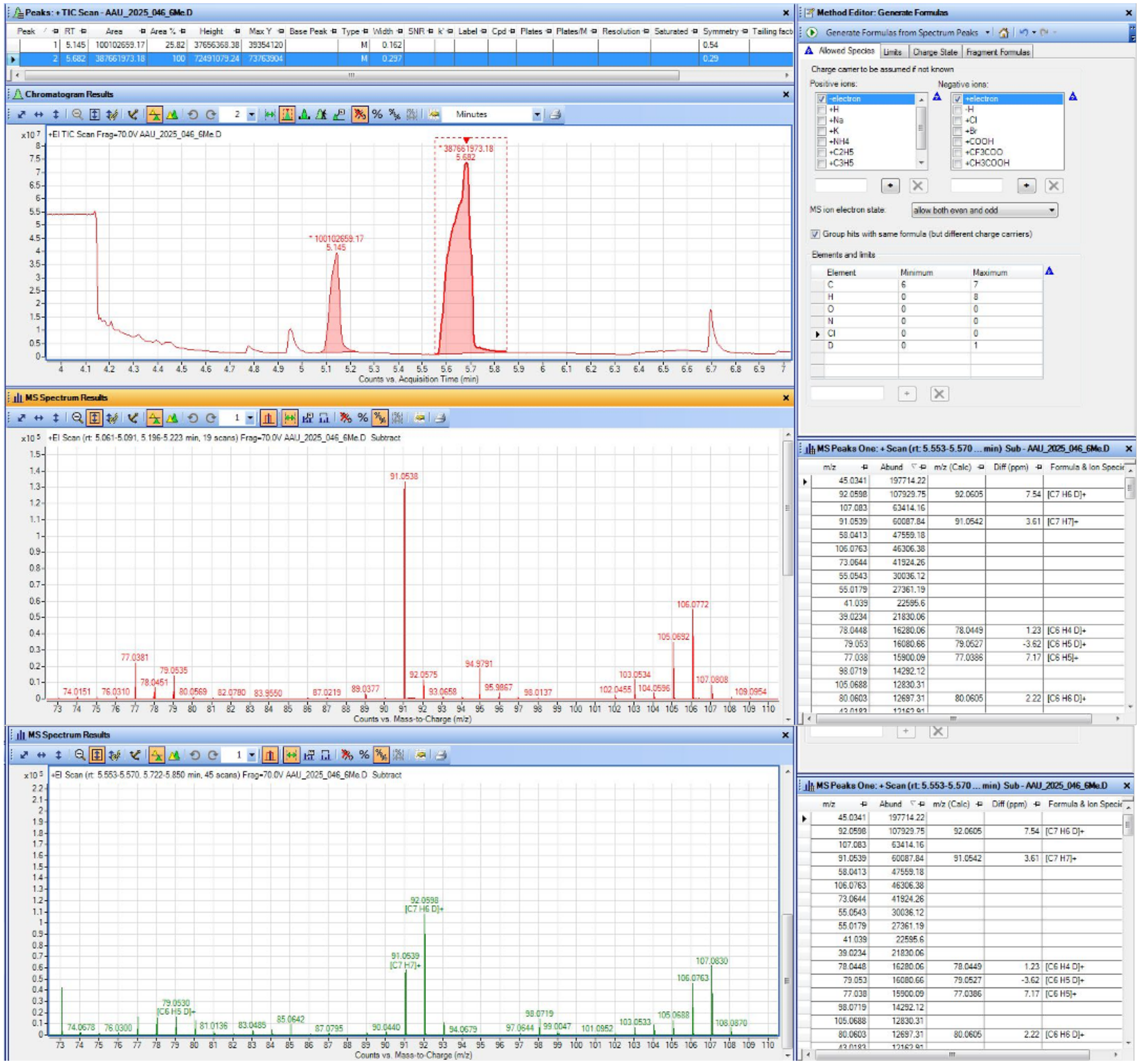

Figure S67: Chromatogram and mass spectrum of **6-Me-ab.**

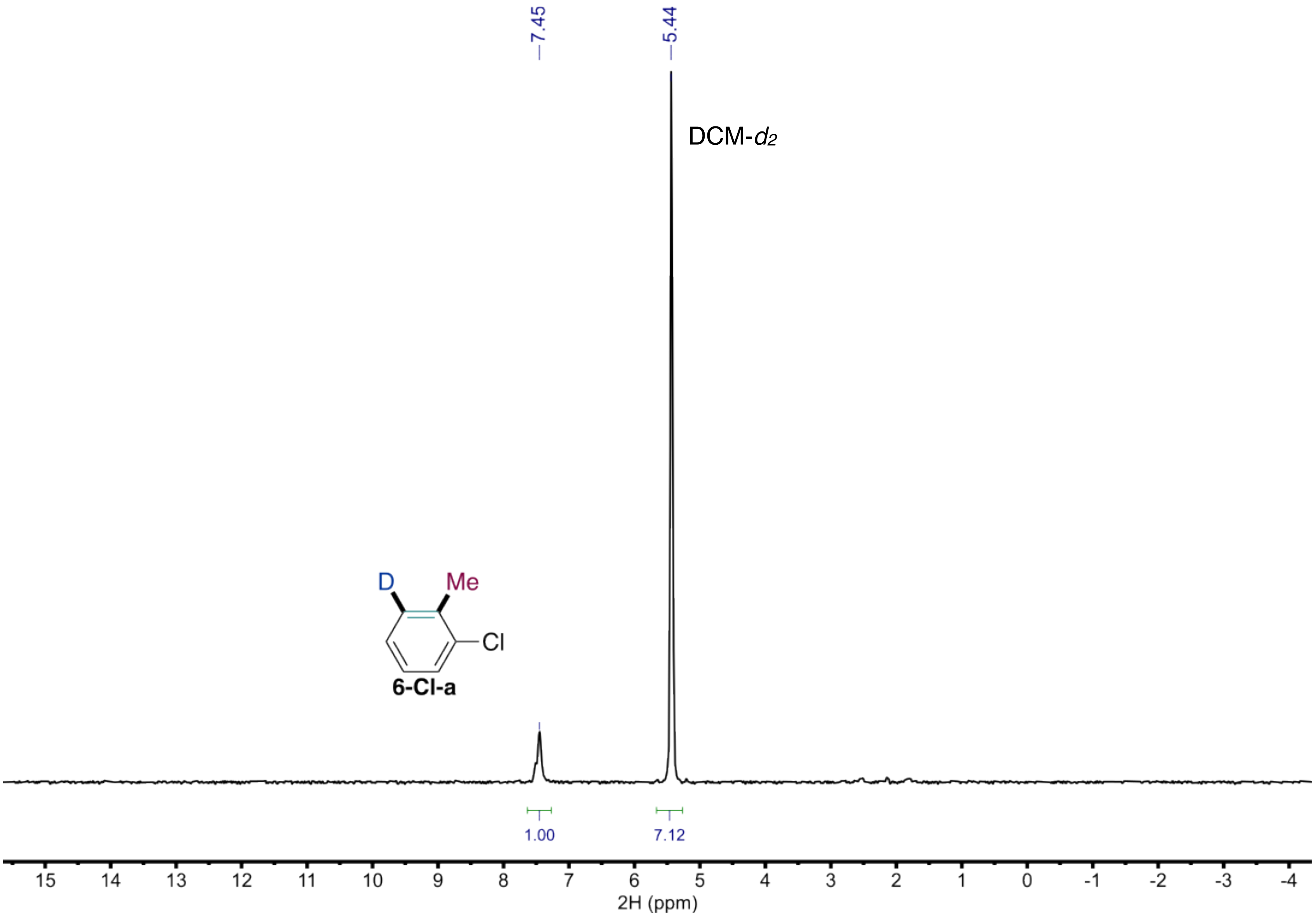

Figure S68: ^{2}H NMR spectrum (61 MHz, 1:1 PhMe:MeCN, 298 K) of difunctionalization of **4-Cl** to furnish **6-Cl-a**. Whittaker-Smoother baseline correction performed.

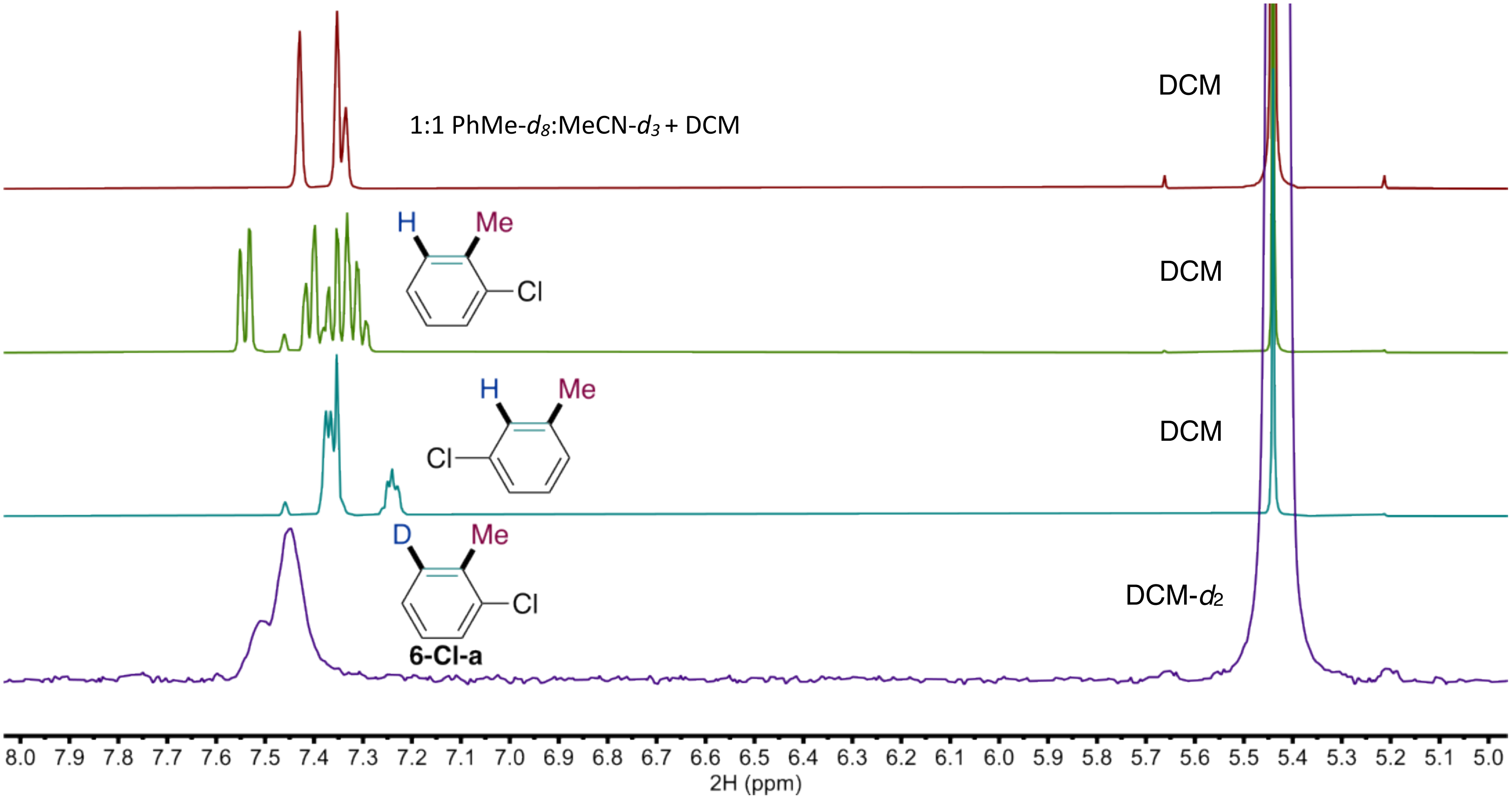

Figure S69: ^{1}H NMR spectra (400 MHz, 1:1 PhMe-d_8:MeCN-d_3, 298 K) of 2-chlorotoluene and 3-chlorotoluene with DCM as an internal reference and ^{2}H NMR spectrum (61 MHz, 1:1 PhMe:MeCN, 298 K) of **6-Cl-a** with DCM-d_2 as an internal reference. Spectra are referenced to the literature value for DCM in MeCN-d_3 (5.44 ppm).[8]

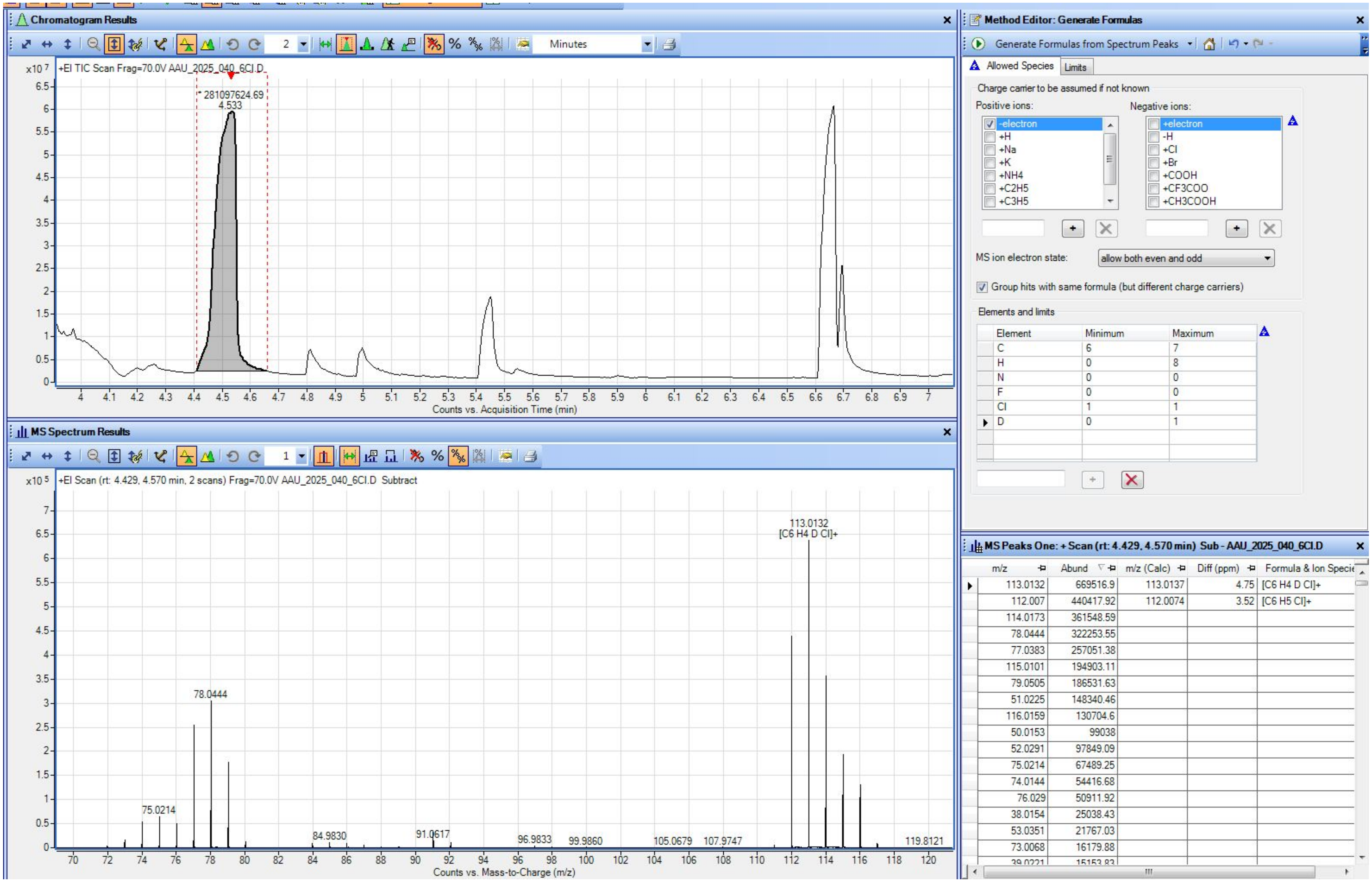

Figure S70: Chromatogram and mass spectrum of **6-Cl-a**.

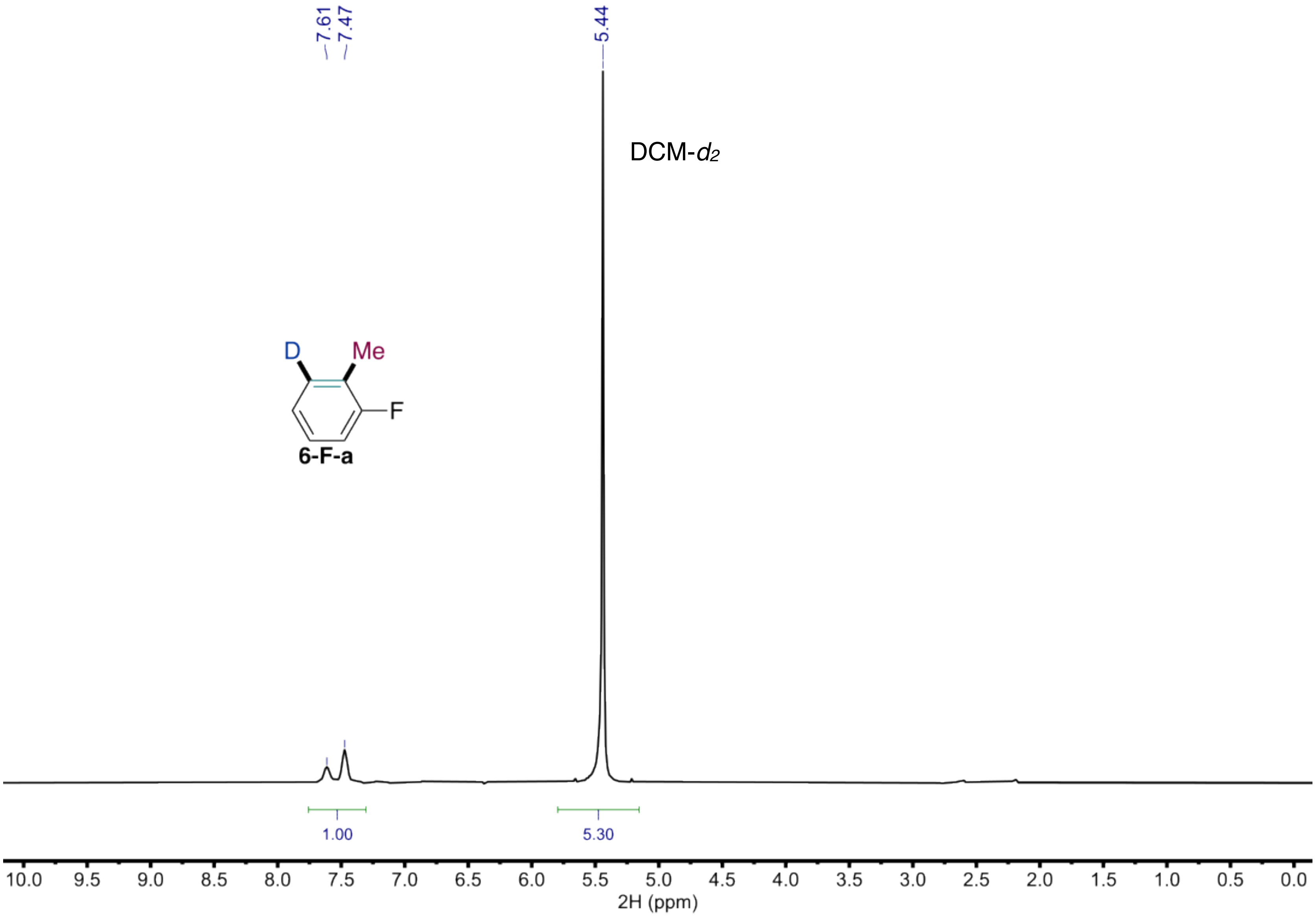

Figure S71: ^{2}H NMR spectrum (61 MHz, 1:1 PhMe:MeCN, 298 K) to furnish **6-F-a**. Whittaker-Smoother baseline correction performed.

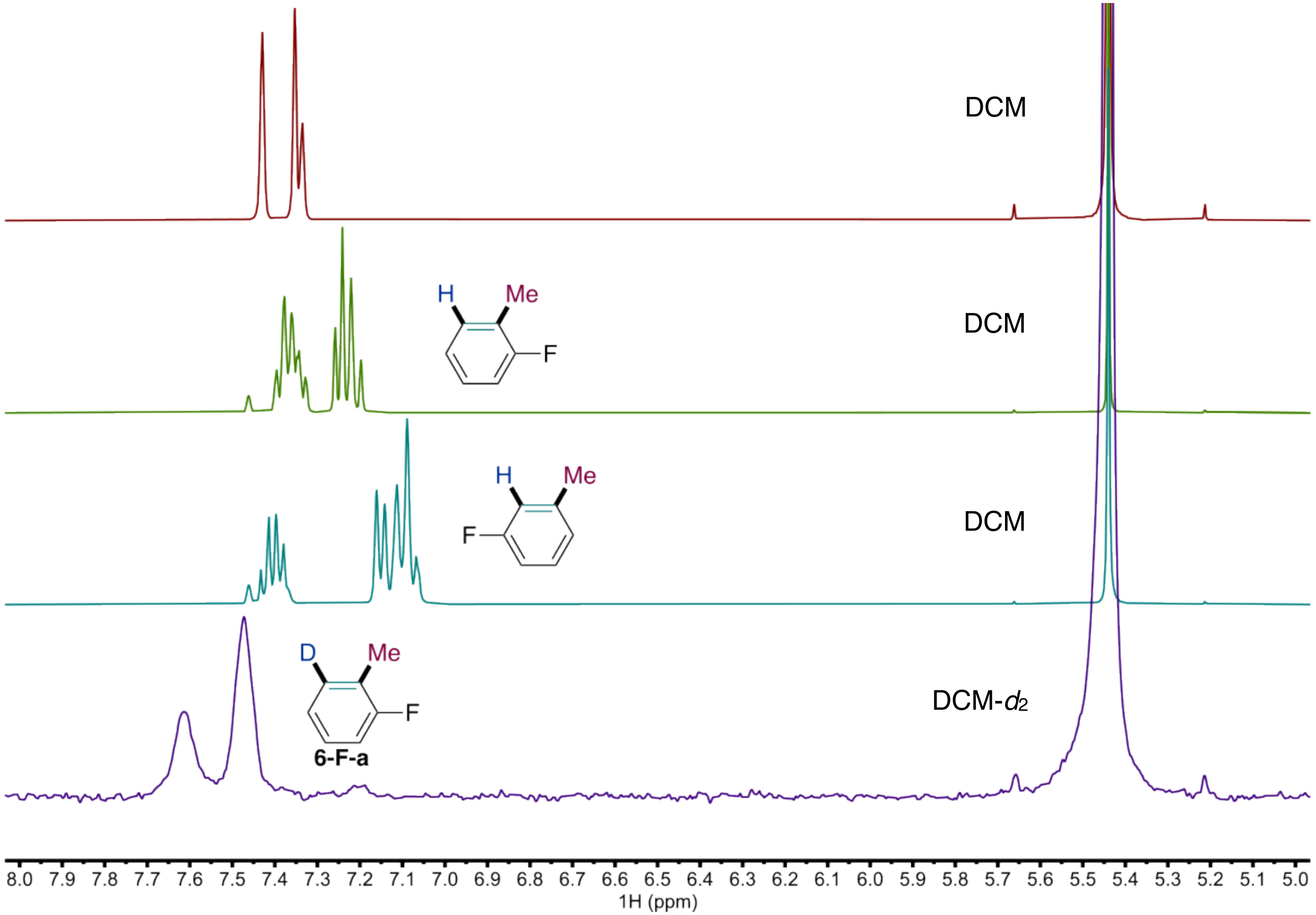

Figure S72: ^{1}H NMR spectra (400 MHz, 1:1 PhMe-d_8:MeCN-d_3, 298 K) of 2-fluorotoluene and 3-fluorotoluene with DCM as an internal reference and ^{2}H NMR spectrum (61 MHz, 1:1 PhMe:MeCN, 298 K) of **6-F-a** with DCM-d_2 as an internal reference. Spectra are referenced to the literature value for DCM in MeCN-d_3 (5.44 ppm).[8]

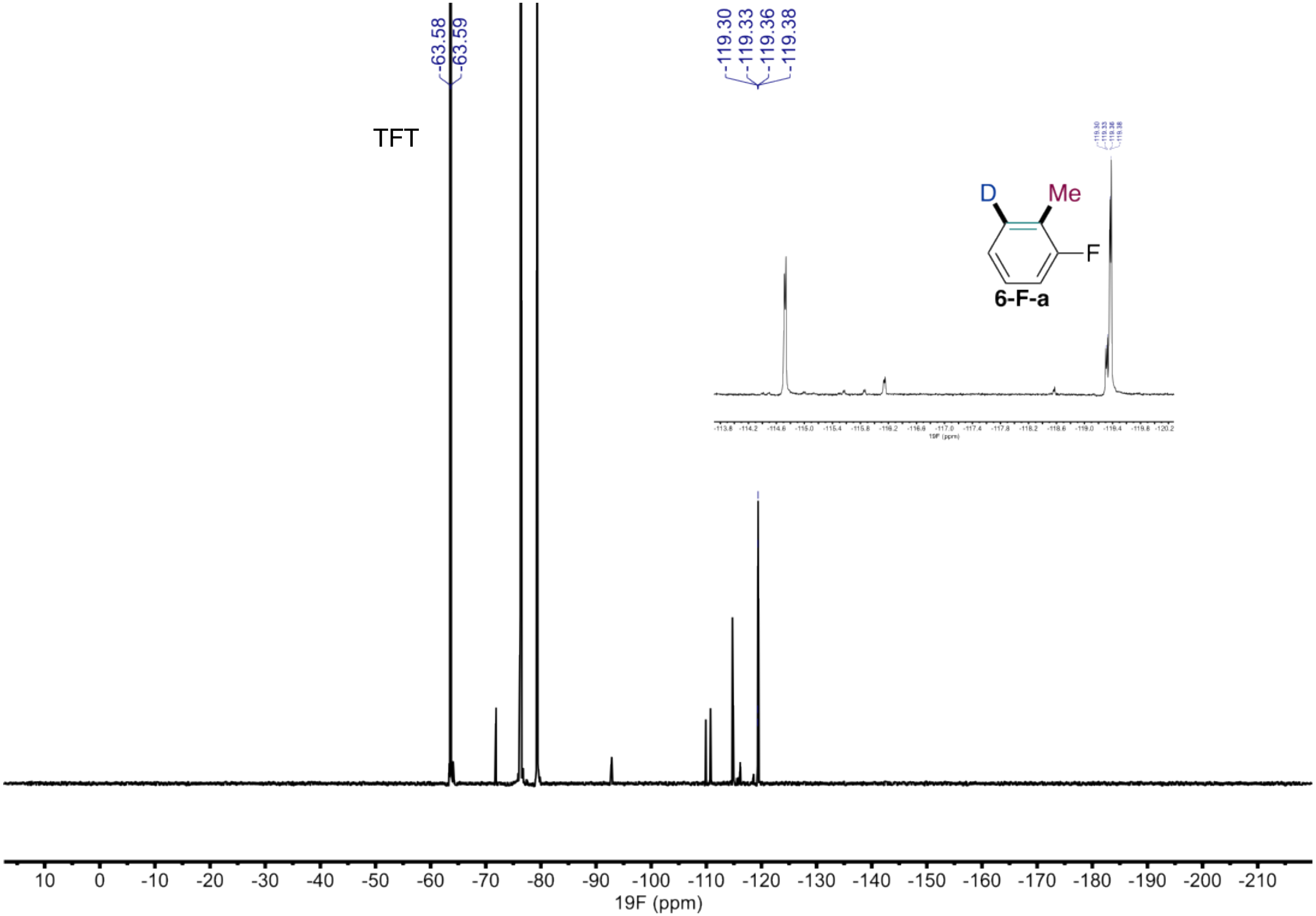

Figure S73: $^{19}F\{^{1}H\}$ NMR spectrum (376 MHz, 1:1 PhMe:MeCN, 298 K) of difunctionalization of **4-F** to furnish **6-F-a.** Whittaker-Smoother baseline correction performed. TFT = trifluorotoluene for internal reference

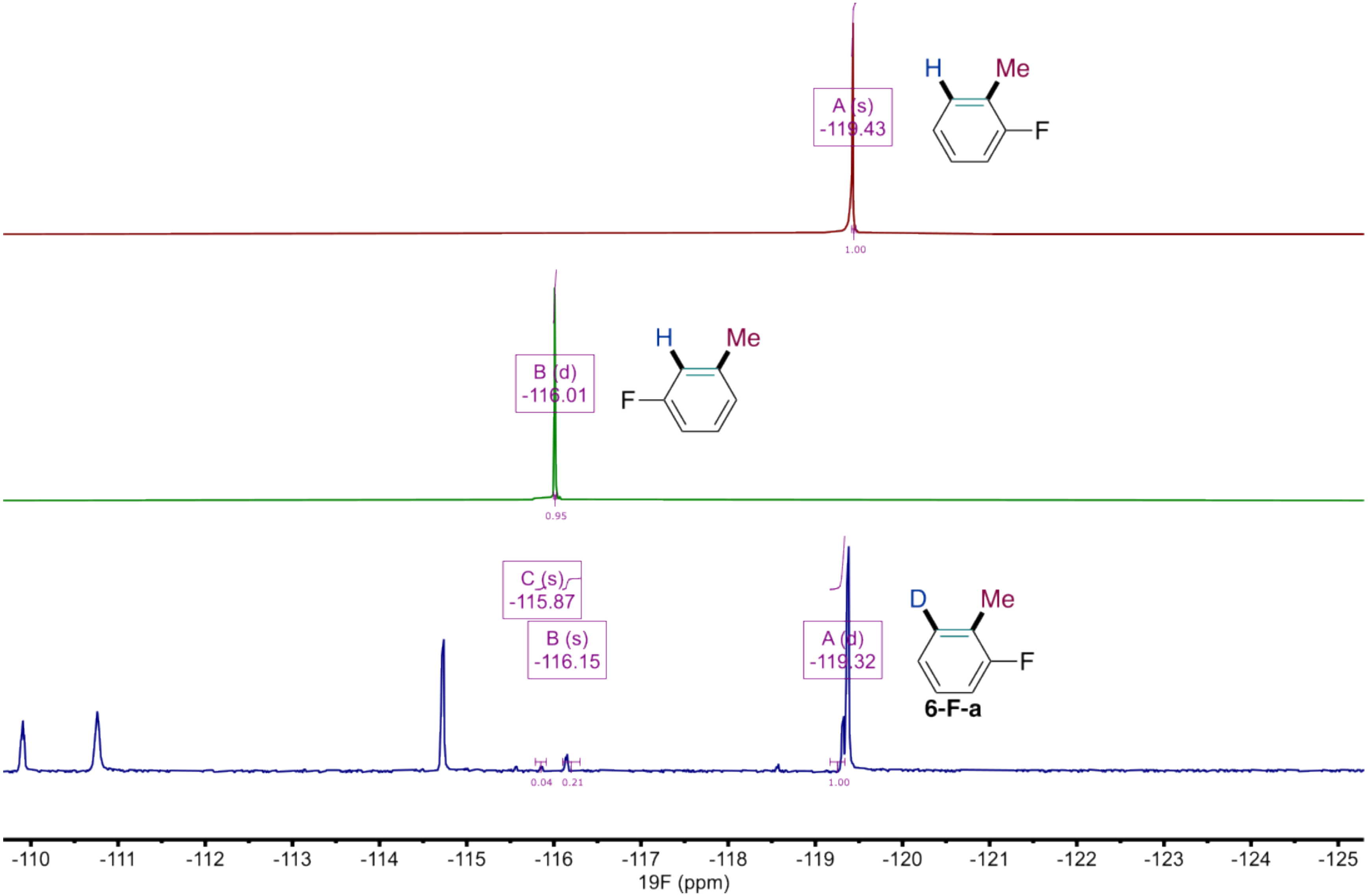

Figure S74: $^{19}F\{^{1}H\}$ NMR spectra (376 MHz, 1:1 PhMe-d_8:MeCN-d_3, 298 K) of 2- and 3-fluorotoluene and $^{19}F\{^{1}H\}$ NMR spectrum (376 MHz, 1:1 PhMe:MeCN, 298 K) of difunctionalization of **4-F** towards **6-F-a** All spectra taken with trifluorotoluene as an internal reference and referenced to the literature value for trifluorotoluene in MeCN-d_3 (-63.59 ppm).[9]

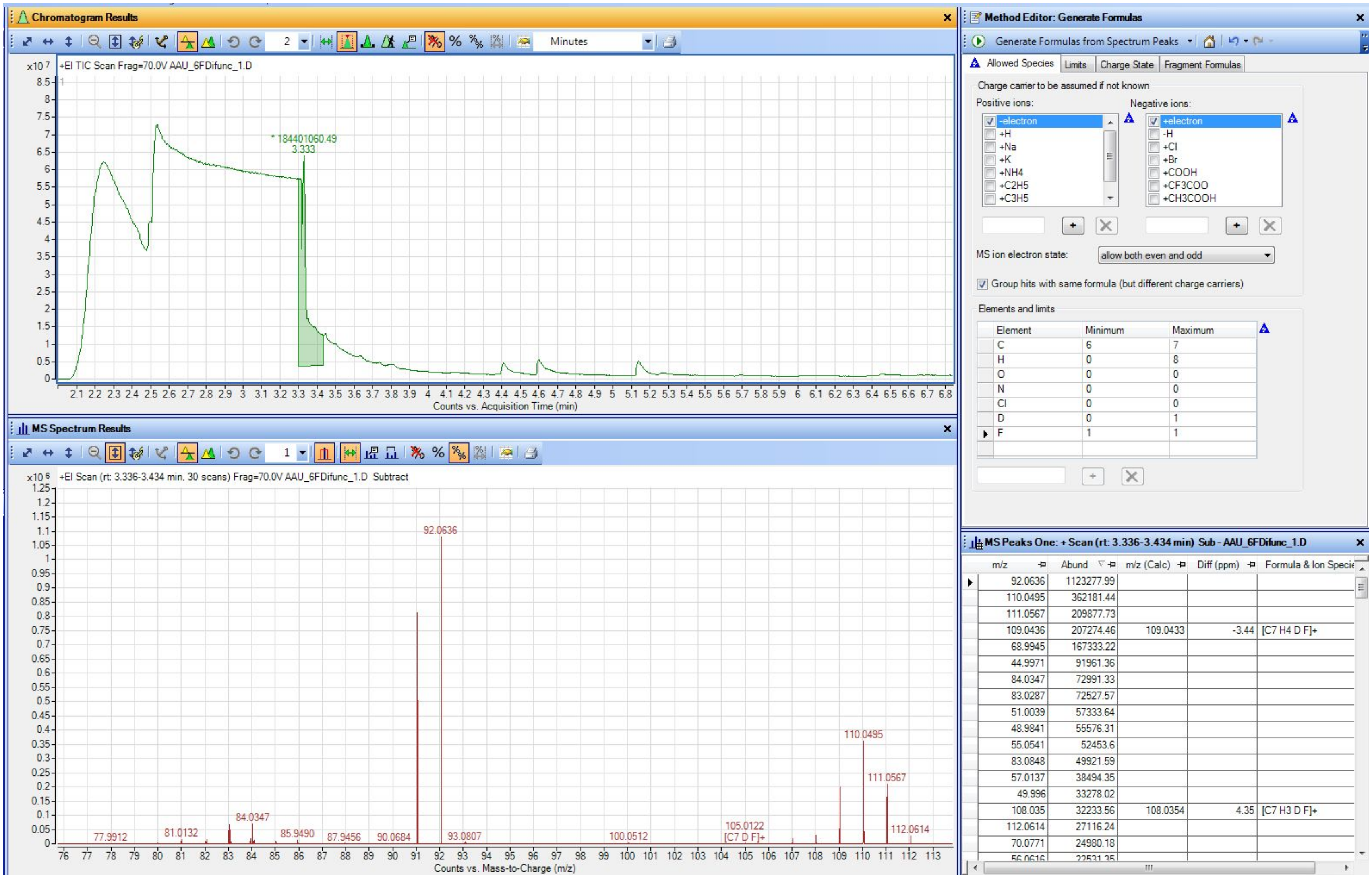

Figure S75: Chromatogram and mass spectrum of **6-F-a**. Due to its volatility, it coelutes with toluene.

X-Ray Crystallographic Details

General Procedure for the Collection and Processing of Crystal Structures

A crystal was placed onto the tip of a 0.15 mm MiTeGen loop and mounted on a Bruker D8 Venture diffractometer equipped with a Photon-III CPAD detector and IμS 3.0 Mo X-ray source for data collection. A preliminary set of cell constants was calculated from reflections harvested from three sets of frames. These initial sets of frames were oriented such that orthogonal wedges of reciprocal space were surveyed. This produced initial orientation matrices which were used to determine a data collection strategy to ensure complete data coverage to a desired resolution.[10] The data collection was carried out using MoKα radiation (graphite monochromator). All major sections of frames were collected with 1.2° steps in ω or ϕ at different detector positions in 2θ. The intensity data were corrected for absorption and decay (SADABS).[11] Final cell constants were calculated from the xyz centroids of strong reflections from the actual data collection after integration (SAINT).[12] See Tables S1 – S14 for additional information specific to each crystal. The structures were solved using SHELXT[13] and refined using SHELXL-2019/3[14] within the SHELXLE program.[15] Space groups were determined based on systematic absences and intensity statistics. A direct-methods solution was calculated which provided most non-hydrogen atoms from the E-map. Full-matrix least squares / difference Fourier cycles were performed which located the remaining non-hydrogen atoms. All non-hydrogen atoms were refined with anisotropic displacement parameters. All hydrogen atoms were placed in ideal positions and refined as riding atoms with relative isotropic displacement parameters. Tables were generated using FinalCif.[16] Crystal structure images were created using Mercury.[17]

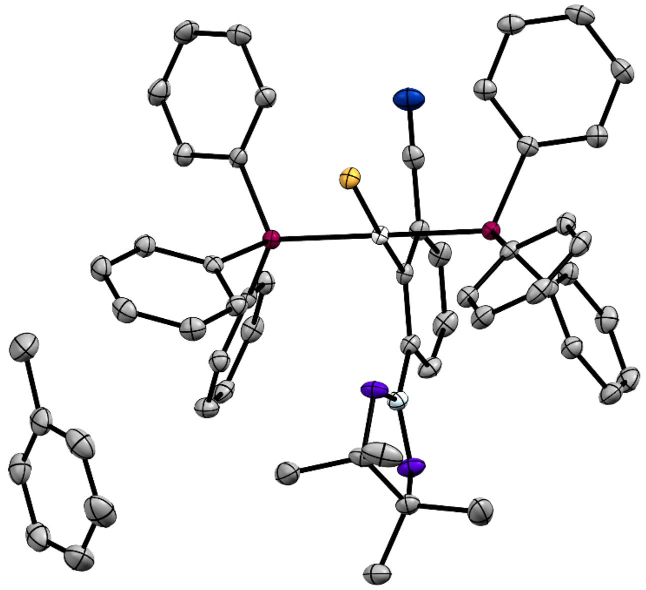

Figure S76: The crystal structure of **2-CN**. Atoms are drawn as thermal ellipsoids at the 50% probability level. Hydrogen atoms have been omitted for clarity.

Table S1. Crystal data and structure refinement for **2-CN**.

CCDC number	2404924
Empirical formula	$C_{56}H_{53}BBrNNiO_2P_2$
Formula weight	983.36
Temperature [K]	100(2)
Crystal system	monoclinic
Space group (number)	$P2_1/n$ (14)
a [Å]	14.9107(9)
b [Å]	19.1937(12)
c [Å]	16.9043(10)
α [°]	90
β [°]	99.3590(10)
γ [°]	90
Volume [Å^3]	4773.5(5)
Z	4
ρ_{calc} [gcm^{-3}]	1.368
μ [mm^{-1}]	1.354
F(000)	2040
Crystal size [mm^3]	0.100×0.100×0.050
Crystal color	yellow
Crystal shape	plate
Radiation	MoK_α (λ=0.71073 Å)
2θ range [°]	3.98 to 56.47 (0.75 Å)

Index ranges	$-13 \leq h \leq 19$<br>$-24 \leq k \leq 25$<br>$-22 \leq l \leq 22$
Reflections collected	54868
Independent reflections	11790<br>R_{int} = 0.0421<br>R_{sigma} = 0.0323
Completeness to θ = 25.242°	100.0 %
Data / Restraints / Parameters	11790/0/582
Absorption correction T_{min}/T_{max} (method)	0.5930/0.7457 (multi-scan)
Goodness-of-fit on F^2	1.028
Final *R* indexes, [$I \geq 2\sigma(I)$]	R_1 = 0.0302, wR_2 = 0.0683
Final *R* indexes, all data	R_1 = 0.0434, wR_2 = 0.0742
Largest peak/hole [eÅ^{-3}]	0.51/–0.25

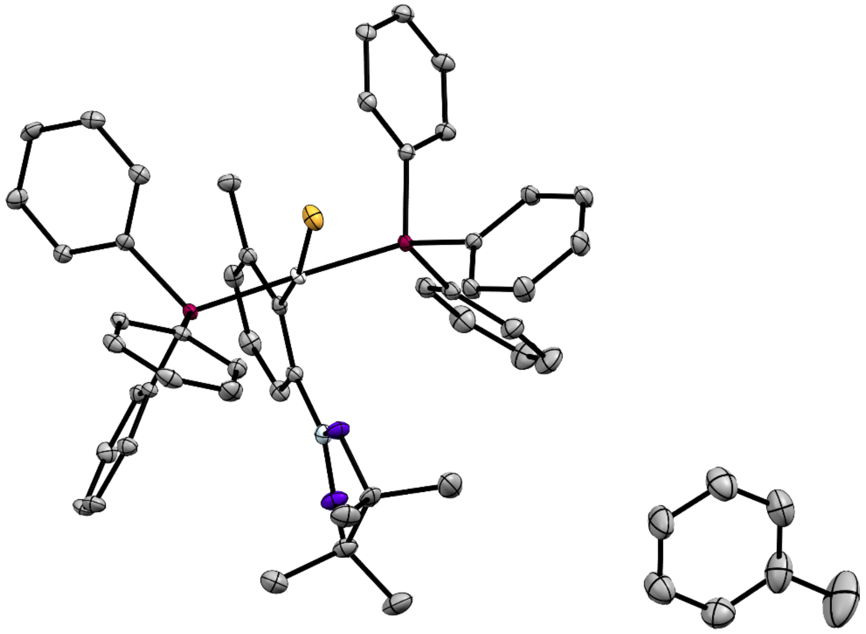

Figure S77: The crystal structure of **2-Me**. Atoms are drawn as thermal ellipsoids at the 50% probability level. Hydrogen atoms have been omitted for clarity. Only the majorly occupied portion of the disordered toluene molecule is shown.

<u>Refinement Details for **2-Me**</u>

The structure contained two disordered molecules located on inversion centers within the unit cell. One of these was successfully modeled as a 50% occupied toluene molecule. The program DSR[19] was used to build the toluene molecule and apply reasonable restraints. Another region of electron density was present around a different inversion center. Attempts to model this as a disordered toluene or pentane molecule were unsuccessful. As such, the SQUEEZE function of PLATON[18] was used to remove the

electron density associated with the disordered molecule. SQUEEZE found 39 electrons in a volume of 229 Å^3 per unit cell.

Table S2. Crystal data and structure refinement for **2-Me**.

CCDC number	2404925
Empirical formula	$C_{52.50}H_{52}BBrNiO_2P_2$, [+solvent]
Formula weight	926.31
Temperature [K]	100(2)
Crystal system	triclinic
Space group (number)	$P\overline{1}$ (2)
a [Å]	10.422(4)
b [Å]	12.857(4)
c [Å]	19.346(8)
α [°]	100.721(6)
β [°]	92.728(7)
γ [°]	109.775(6)
Volume [Å^3]	2379.5(15)
Z	2
ρ_{calc} [gcm^{-3}]	1.293
μ [mm^{-1}]	1.353
F(000)	962
Crystal size [mm^3]	0.100×0.100×0.050
Crystal colour	orange
Crystal shape	plate
Radiation	MoK_α (λ=0.71073 Å)
2θ range [°]	4.18 to 56.81 (0.75 Å)
Index ranges	$-13 \leq h \leq 13$<br>$-10 \leq k \leq 17$<br>$-25 \leq l \leq 25$
Reflections collected	61421
Independent reflections	11917<br>R_{int} = 0.0502<br>R_{sigma} = 0.0379
Completeness to θ = 25.242°	100.0 %
Data / Restraints / Parameters	11917/122/574
Absorption correction T_{min}/T_{max} (method)	0.6943/0.7457 (multi-scan)
Goodness-of-fit on F^2	1.038
Final *R* indexes [$I \geq 2\sigma(I)$]	R_1 = 0.0426, wR_2 = 0.1020
Final *R* indexes [all data]	R_1 = 0.0567, wR_2 = 0.1087
Largest peak/hole [eÅ^{-3}]	1.71/–1.06

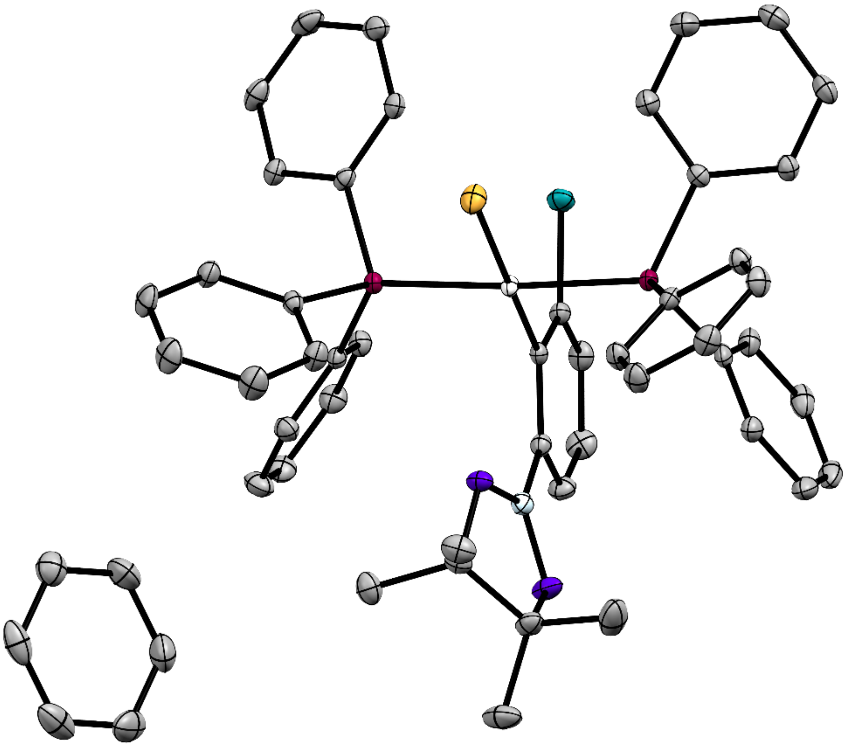

Figure S78: The crystal structure of **2-Cl**. Atoms are drawn as thermal ellipsoids at the 50% probability level. Only the majorly occupied disordered parts are shown.

<u>Refinement Details for **2-Cl**</u>

The pinacolborane group of the structure is disordered and modeled in two parts with occupancies of 90.4% and 9.6%. The boron atom was used as a pivot atom to model the disorder, and the EADP and EXYZ constraints were applied to B1 and B1'. Because of the low occupancy of the minorly occupied group, the EADP constraint was utilized on the following pairs of atoms: O1, O1'; O2, O2'; C7, C7'; C8, C8; C9, C11'; C10, C9'; C11, C12'; C12, C10'. A strong SAME restraint was applied to the minorly occupied portion to ensure the same geometry as the majorly occupied portion.

Table S3. Crystal data and structure refinement for **2-Cl**.

CCDC number	2423781
Empirical formula	$C_{54}H_{51}BBrClNiO_2P_2$
Formula weight	978.77
Temperature [K]	100(2)
Crystal system	monoclinic
Space group (number)	$P2_1/c$ (14)
a [Å]	12.0181(6)

b [Å]	18.4650(8)
c [Å]	21.0660(9)
α [°]	90
β [°]	91.206(2)
γ [°]	90
Volume [Å^3]	4673.8(4)
Z	4
ρ_{calc} [gcm^{-3}]	1.391
μ [mm^{-1}]	1.437
F(000)	2024
Crystal size [mm^3]	0.040×0.140×0.180
Crystal colour	yellow
Crystal shape	plate
Radiation	MoK_α (λ=0.71073 Å)
2θ range [°]	3.87 to 59.21 (0.72 Å)
Index ranges	−16 ≤ h ≤ 16<br>−25 ≤ k ≤ 25<br>−27 ≤ l ≤ 29
Reflections collected	86842
Independent reflections	13137<br>R_{int} = 0.0574<br>R_{sigma} = 0.0404
Completeness to θ = 25.242°	100.0 %
Data / Restraints / Parameters	13137 / 24 / 592
Absorption correction T_{min}/T_{max} (method)	0.6687 / 0.7459 (multi-scan)
Goodness-of-fit on F^2	1.044
Final *R* indexes [$I \geq 2\sigma(I)$]	R_1 = 0.0356, wR_2 = 0.0813
Final *R* indexes [all data]	R_1 = 0.0532, wR_2 = 0.0894
Largest peak/hole [eÅ^{-3}]	0.51/−0.80

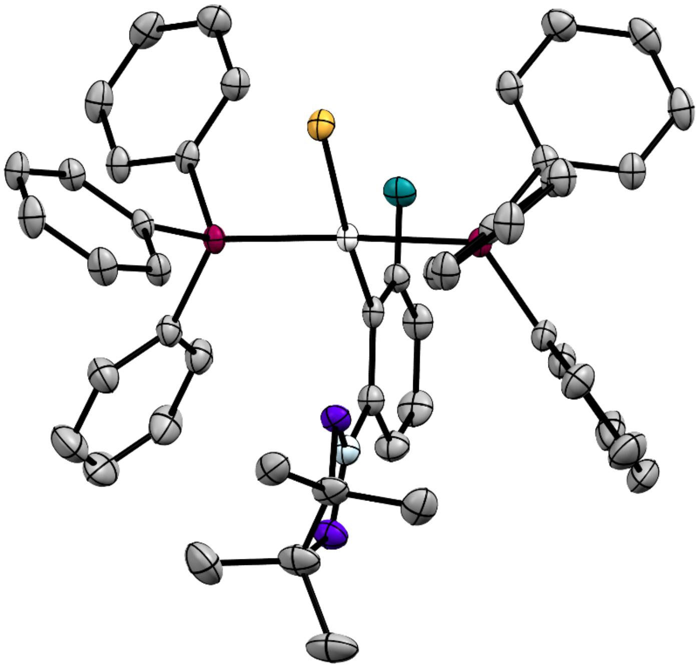

Figure S79: The crystal structure of **2-F**. Atoms are drawn as thermal ellipsoids at the 50% probability level. Hydrogen atoms have been omitted for clarity. Only one conformation of the disordered pinacolborane group is shown.

<u>Refinement Details for **2-F**</u>

The molecule is located on a crystallographic mirror. The pinacolborane functional group is disordered across the mirror. The group has been modeled in two parts, each with 50% occupancy. The SADI restraint was applied to the following groups of atoms to ensure the pinacolborane group had a realistic shape: [C7 and C10, C7 and C9, C8 and C11, C8 and C12], [C9 and C10, C11 and C12], [C9 and C12, C10 and C11]. There was disordered solvent in the structure. The SQUEEZE function of PLATON[18] was used to remove the electron density associated with the disordered solvent molecules. SQUEEZE found two voids with a volume of 1384 cubic angstroms each containing 403 electrons per unit cell.

Table S4. Crystal data and structure refinement for **2-F**.

CCDC number	2404923
Empirical formula	$C_{48}H_{45}BBrFNiO_2P2$[+solvent]
Formula weight	884.21
Temperature [K]	100(2)
Crystal system	orthorhombic
Space group (number)	$Ibam$ (72)
a [Å]	24.8592(10)
b [Å]	17.9431(7)
c [Å]	23.2286(8)
α [°]	90
β [°]	90
γ [°]	90
Volume [Å^3]	10361.1(7)
Z	8
ρ_{calc} [gcm^{-3}]	1.134
μ [mm^{-1}]	1.243
F(000)	3648
Crystal size [mm^3]	0.030×0.080×0.110
Crystal colour	yellow
Crystal shape	plate
Radiation	MoK_α (λ=0.71073 Å)
2θ range [°]	4.36 to 50.70 (0.83 Å)
Index ranges	$-29 \le h \le 24$<br>$-21 \le k \le 21$<br>$-19 \le l \le 27$
Reflections collected	34216
Independent reflections	4871<br>R_{int} = 0.0417<br>R_{sigma} = 0.0248
Completeness to θ = 25.242°	99.9 %
Data / Restraints / Parameters	4871 / 8 / 308
Absorption correction T_{min}/T_{max} (method)	0.6604 / 0.7458 (multi-scan)
Goodness-of-fit on F^2	1.042
Final *R* indexes [$I \ge 2\sigma(I)$]	R_1 = 0.0250, wR_2 = 0.0588
Final *R* indexes [all data]	R_1 = 0.0366, wR_2 = 0.0644
Largest peak/hole [eÅ^{-3}]	0.40/–0.20

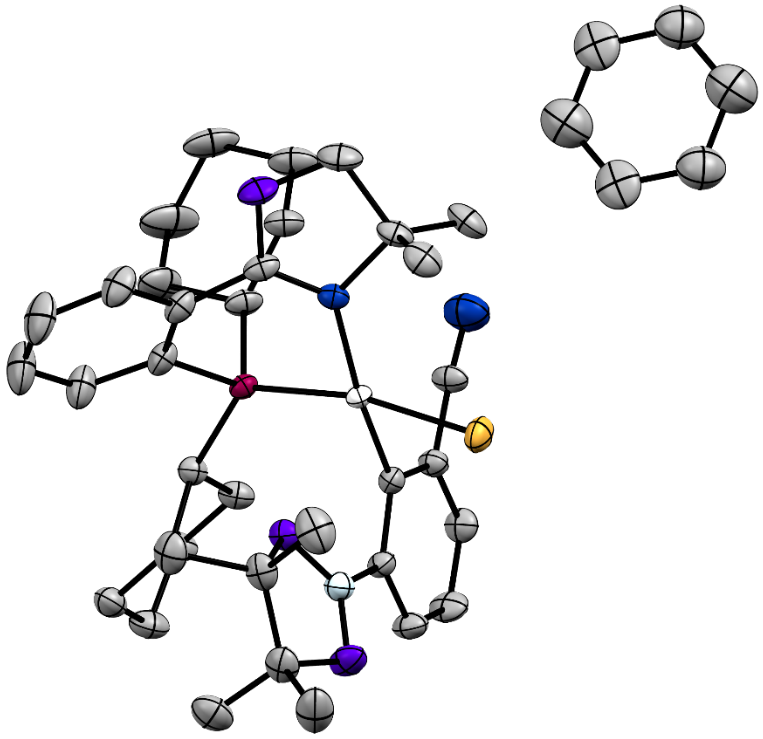

Figure S80: The crystal structure of **3-CN**. Atoms are drawn as thermal ellipsoids at the 50% probability level. Hydrogen atoms have been omitted for clarity. Only one part of the disordered benzene molecule is shown.

Refinement details for **3-CN**
The reflections -7 7 7, 3 5 2, -6 4 4, 1 3 3, and 0 4 3 were omitted due to suspected interference from the beam stop.
The co-crystallized benzene molecule is disordered on a two-fold rotation axis and has been modeled as a half-occupied full molecule on the rotation axis. Because of the disorder, the molecule is heavily restrained, using a strong DFIX restraint to restrain all the bonds in the molecule to a length of approximately 1.395 Å. A strong SAME restraint, a FLAT restraint, and a RIGU restraint were also applied to the molecule. The EADP constraint was applied to pairs of atoms related by the two-fold axis: C37 and C40, C38 and C42, C39 and C41.

Table S5. Crystal data and structure refinement for **3-CN**.

CCDC number	2404927
Empirical formula	$C_{39}H_{52}BBrN_2NiO_3P$
Formula weight	777.22
Temperature [K]	130(2)
Crystal system	monoclinic
Space group (number)	$C2/c$ (15)
a [Å]	27.8212(8)
b [Å]	18.6414(4)
c [Å]	19.8748(6)
α [°]	90
β [°]	131.5870(10)
γ [°]	90
Volume [Å^3]	7709.5(4)
Z	8
ρ_{calc} [gcm^{-3}]	1.339
μ [mm^{-1}]	1.619
F(000)	3256
Crystal size [mm^3]	0.140×0.140×0.140
Crystal color	orange
Crystal shape	plate
Radiation	MoK_α (λ=0.71073 Å)
2θ range [°]	3.91 to 52.79 (0.80 Å)
Index ranges	–34 ≤ h ≤ 34<br>–21 ≤ k ≤ 23<br>–24 ≤ l ≤ 24
Reflections collected	112421
Independent reflections	7877<br>R_{int} = 0.0425<br>R_{sigma} = 0.0206
Completeness to θ = 25.242°	99.8 %
Data / Restraints / Parameters	7877/50/448
Absorption correction T_{min}/T_{max} (method)	0.6552/0.7454 (multi-scan)
Goodness-of-fit on F^2	1.051
Final *R* indexes [$I \geq 2\sigma(I)$]	R_1 = 0.0268, wR_2 = 0.0647
Final *R* indexes [all data]	R_1 = 0.0331, wR_2 = 0.0694
Largest peak/hole [eÅ^{-3}]	1.05/–0.59

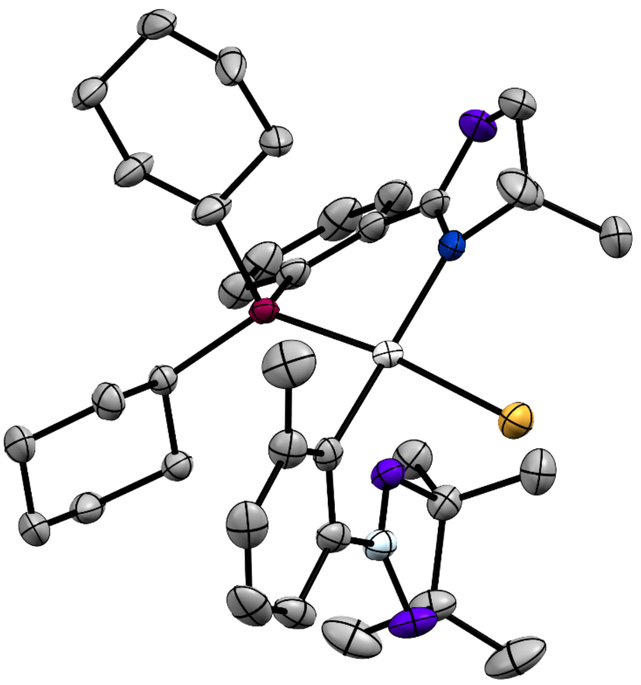

Figure S81: The crystal structure showing one of the molecules of **3-Me** present in the asymmetric unit. Atoms are drawn as thermal ellipsoids at the 50% probability level. Hydrogen atoms are omitted for clarity. Only the majorly occupied disordered parts are shown.

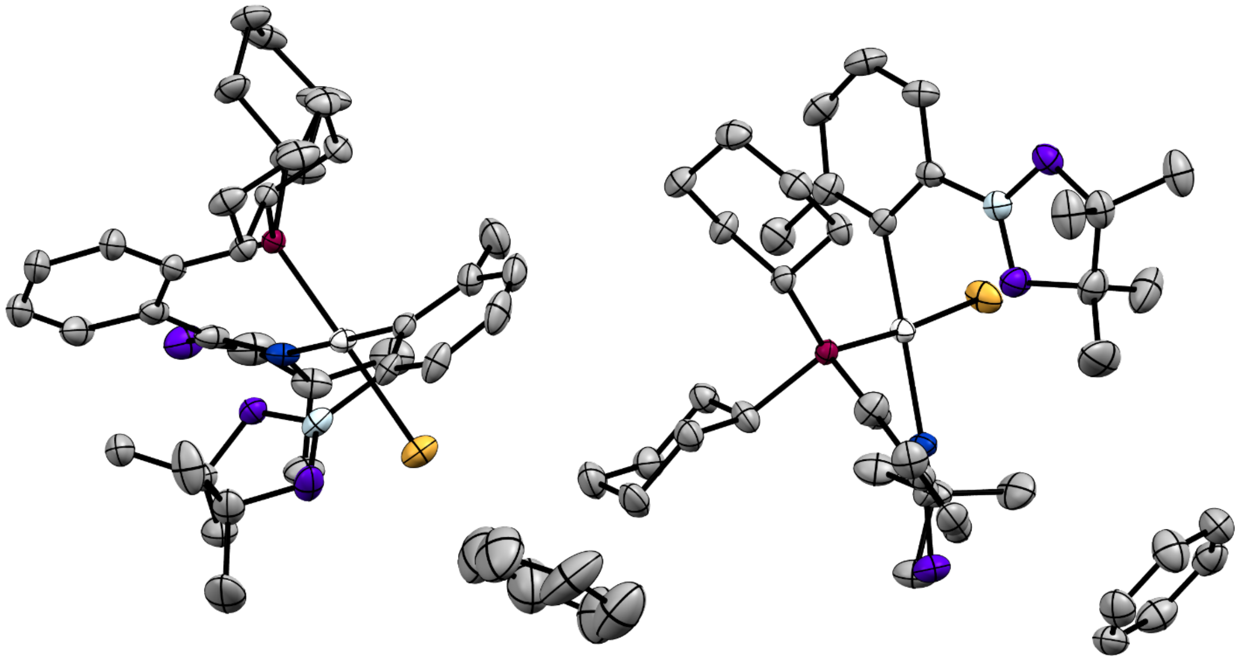

Figure S82: An image showing the full asymmetric unit of **3-Me**. Atoms are drawn as thermal ellipsoids at the 50% probability level. Hydrogen atoms are omitted for clarity. Only the majorly occupied disordered parts are shown.

Refinement Details for **3-Me**

The reflection -2 2 3 was omitted due to suspected interference from the beam stop. There is some disorder present within the structure. In each of the metal complexes, there is a disordered cyclohexyl group. Each group was modeled as rotationally disordered with the carbon atom bound to phosphorus as a pivot atom, with the EXYZ restraint being used on the following pairs of atoms: C19_1 and C19'_1, C25_2 and C25'_2. Due to the low occupancy of the minorly disordered portion of residue 1 and the closeness of some atoms in space, the EADP constraint was used on the following pairs of atoms: C19_1 and C19'_1, C20_1 and C20'_1, C21_1 and C21'_1, C22_1 and C22'_1, C23_1 and C23'_1, C24_1 and C24'_1, C25_2 and C25'_2, C30_2 and C30'_2. Strong SAME restraints were used to model all disordered cyclohexyl groups. The occupancy ratio for the disordered cyclohexyl groups are approximately 86:14 and 80:20 for residues 1 and 2 respectively. The asymmetric unit also contained two co-crystallized benzene molecules. The benzene molecule in residue 4 is partially occupied, with an occupancy of approximately 84%. Some solvent may have been lost during the preparation and mounting of the crystal. Both benzene molecules are modeled using SAME restraints to ensure symmetry, as well as RIGU restraints. Additionally, a strong DFIX restraint was applied to the partially occupied benzene to ensure realistic bond lengths. There was electron density corresponding to a solvent molecule located near one of the inversion centers in the unit cell. This solvent molecule was unable to be modeled, so the SQUEEZE function of PLATON[18] was used to remove the electron density associated with the disordered solvent. SQUEEZE found 48 electrons within a void space of 227 Å^3.

Table S6. Crystal data and structure refinement for **3-Me**.

CCDC number	2404929
Empirical formula	$C_{41.53}H_{57.53}BBrNNiO_3P$[+solvent]
Formula weight	799.21
Temperature [K]	130(2)
Crystal system	triclinic
Space group (number)	$P\bar{1}$ (2)
a [Å]	11.1103(4)
b [Å]	19.0185(7)
c [Å]	19.9616(7)
α [°]	88.9670(10)
β [°]	87.8210(10)
γ [°]	79.2640(10)
Volume [Å^3]	4140.8(3)
Z	4
ρ_{calc} [gcm^{-3}]	1.282
μ [mm^{-1}]	1.509
F(000)	1683
Crystal size [mm^3]	0.110×0.080×0.070
Crystal color	orange
Crystal shape	block
Radiation	MoK_α (λ=0.71073 Å)
2θ range [°]	3.73 to 52.96 (0.80 Å)
Index ranges	$-13 \leq h \leq 13$<br>$-23 \leq k \leq 23$<br>$-24 \leq l \leq 24$
Reflections collected	108795
Independent reflections	17008<br>R_{int} = 0.0475<br>R_{sigma} = 0.0329
Completeness to θ = 25.242°	100.0 %
Data / Restraints / Parameters	17008/138/978
Absorption correction T_{min}/T_{max} (method)	0.6911/0.7454 (multi-scan)
Goodness-of-fit on F^2	1.073
Final *R* indexes [$I \geq 2\sigma(I)$]	R_1 = 0.0442, wR_2 = 0.1083
Final *R* indexes [all data]	R_1 = 0.0592, wR_2 = 0.1159
Largest peak/hole [eÅ^{-3}]	0.77/–0.88

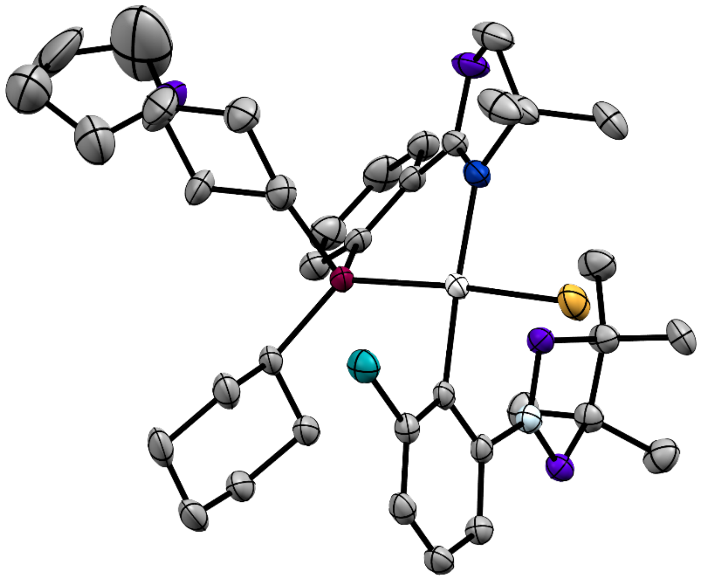

Figure S83: The crystal structure of **3-Cl**. Atoms are drawn as thermal ellipsoids at the 50% probability level. Hydrogen atoms are omitted for clarity. Only the majorly occupied portions of disordered regions are shown.

Refinement Details for **3-Cl**

The pinacolborane group is disordered and has been modeled in two parts using B1 as a pivot atom. The EADP and EXYZ constraints were applied to B1 and B1'. The SAME restraint was applied to the minorly disordered part to ensure the same geometry as the majorly occupied part. Because of their proximity in space, the EADP constraint was applied to the following pairs of atoms: C32 and C33', C35 and C34'. There was disordered solvent present in the structure. One THF molecule was disordered on a two-fold axis and was able to be modeled using a model and restraints taken from the DSR program.[19] The other solvent was unable to be modeled, and the SQUEEZE function of PLATON[18] was used to remove the electron density associated with the disordered solvent. SQUEEZE found four voids of approximately 157 cubic angstroms each containing 40 electrons.

Table S7: Crystal data and structure refinement for **3-Cl**.

CCDC number	2404926
Empirical formula	$C_{37}H_{53}BBrClNNiO_{3.50}P$ [+solvent]
Formula weight	783.65
Temperature [K]	100(2)
Crystal system	monoclinic
Space group (number)	$C2/c$ (15)
a [Å]	28.8710(15)
b [Å]	18.4649(10)
c [Å]	19.9729(11)
α [°]	90
β [°]	132.8880(10)
γ [°]	90
Volume [Å^3]	7801.3(7)
Z	8
ρ_{calc} [gcm^{-3}]	1.334
μ [mm^{-1}]	1.667
F(000)	3280
Crystal size [mm^3]	0.070×0.140×0.210
Crystal colour	orange
Crystal shape	plate
Radiation	MoK_α (λ=0.71073 Å)
2θ range [°]	3.85 to 50.70 (0.83 Å)
Index ranges	$-34 \le h \le 34$<br>$-22 \le k \le 22$<br>$-23 \le l \le 24$
Reflections collected	34451
Independent reflections	7142<br>R_{int} = 0.0292<br>R_{sigma} = 0.0226
Completeness to θ = 25.242°	99.9 %
Data / Restraints / Parameters	7142 / 144 / 513
Absorption correction T_{min}/T_{max} (method)	0.6571 / 0.7461 (multi-scan)
Goodness-of-fit on F^2	1.041
Final *R* indexes [$I \ge 2\sigma(I)$]	R_1 = 0.0351, wR_2 = 0.0924
Final *R* indexes [all data]	R_1 = 0.0455, wR_2 = 0.1009
Largest peak/hole [eÅ^{-3}]	0.77/–0.96

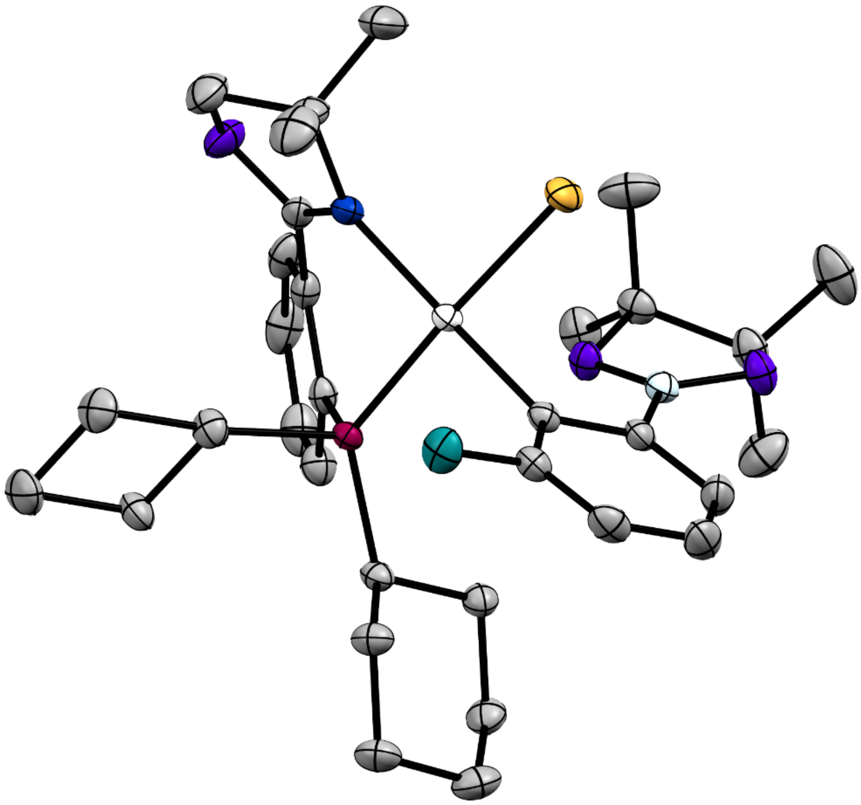

Figure S84: The crystal structure of **3-F**. Atoms are drawn as thermal ellipsoids at the 50% probability level. Hydrogen atoms are omitted for clarity.

Table S8. Crystal data and structure refinement for **3-F**.

CCDC number	2404928
Empirical formula	$C_{35}H_{49}BBrFNNiO_3P$
Formula weight	731.15
Temperature [K]	130(2)
Crystal system	monoclinic
Space group (number)	$P2_1/c$ (14)
a [Å]	18.3404(5)
b [Å]	10.9530(3)
c [Å]	19.0212(6)
α [°]	90
β [°]	114.4040(10)
γ [°]	90
Volume [Å^3]	3479.63(18)
Z	4
ρ_{calc} [gcm^{-3}]	1.396
μ [mm^{-1}]	1.792

$F(000)$	1528
Crystal size [mm^3]	0.100×0.100×0.100
Crystal color	orange
Crystal shape	block
Radiation	MoK_α (λ=0.71073 Å)
2θ range [°]	4.31 to 54.26 (0.78 Å)
Index ranges	$-22 \le h \le 23$<br>$-14 \le k \le 14$<br>$-22 \le l \le 24$
Reflections collected	32874
Independent reflections	7695<br>R_{int} = 0.0387<br>R_{sigma} = 0.0344
Completeness to θ = 25.242°	100.0 %
Data / Restraints / Parameters	7695/0/403
Absorption correction T_{min}/T_{max} (method)	0.6567/0.7455 (multi-scan)
Goodness-of-fit on F^2	1.031
Final R indexes [$I \geq 2\sigma(I)$]	R_1 = 0.0296, wR_2 = 0.0685
Final R indexes [all data]	R_1 = 0.0399, wR_2 = 0.0726
Largest peak/hole [eÅ^{-3}]	0.41/–0.29

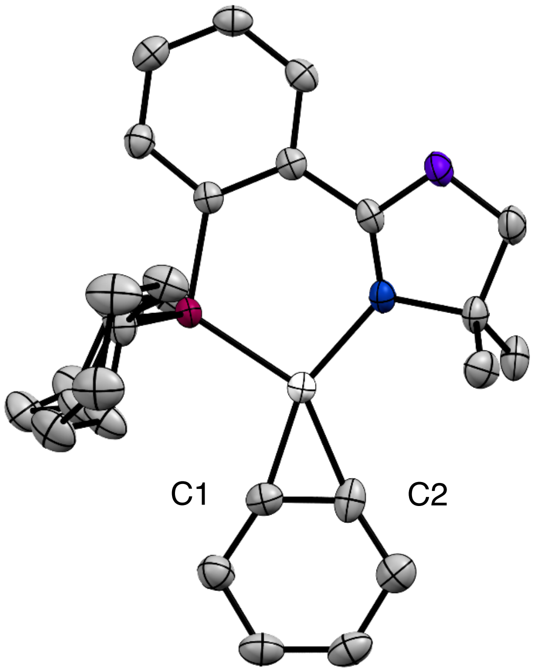

Figure S85: The crystal structure of **4-H**. The unusual ellipsoid shape of C2 caused by the unaccounted-for electron density can be seen in this image. Atoms are drawn as thermal ellipsoids at the 50% probability level. Hydrogen atoms are omitted for clarity. Only the majorly occupied portion of the disordered cyclohexane is shown.

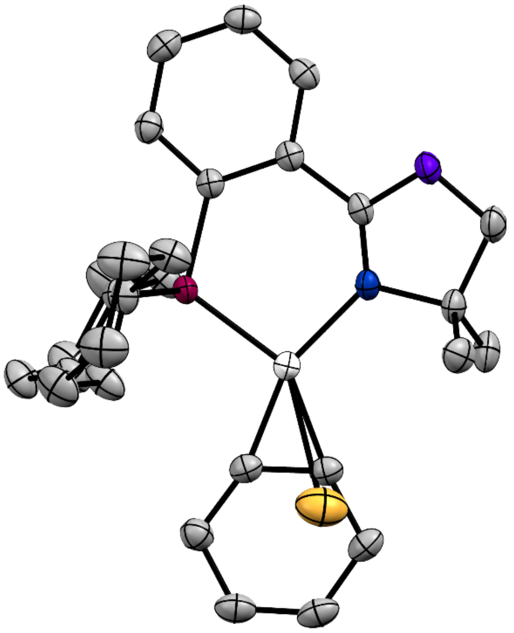

Figure S86: The crystal structure of **4-H** with the plausible co-crystallized species $C_{23}H_{35}BrNNiOP$ as they are in the asymmetric unit of the crystal. Atoms are drawn as thermal ellipsoids at the 50% probability level. Hydrogens are omitted for clarity. Only the majorly occupied portion of the disordered cyclohexane is shown.

Refinement Details for **4-H**

The reflection 0 0 2 was omitted due to suspected interference from the beam stop.

During refinement there was unaccounted for electron density located 0.78 and 2.59 angstroms from C2 and Ni1 respectively. The presence of the electron density was causing the ADP of C2 to distort in respect to neighboring atoms in an attempt to account for the electron density (**Figure S85**). Based on the distance of the electron density from Ni1 and the reaction conditions used to generate the compound, it is plausible that the density is due to a small amount of a Br containing compound. Placing an approximately 3% occupied Br atom accounted for the electron density, as well as gave C2 a more reasonable ADP in respect to its neighbors (**Figure S86**). However, because no other evidence for this bromide species has been collected, the final model does not include it.

The cyclohexyl ring containing carbons C18 > C23 is disordered and modeled in two parts with an occupancy ratio of approximately 96:4. This disorder is modeled using C18 as a pivot atom, with the EADP and EXYZ constraints applied to C18 and C18’. Because of their proximity in space, the EADP constraint was applied to C21 and C21’. The SAME restraint has been used on both the major and minorly occupied parts of the ring to ensure the rings are symmetric. A standard RIGU restraint was applied to C18 > C23, and a strong RIGU restraint was applied to C18’ > C23’. A stronger restraint was necessary on the minorly occupied portion to keep atom C22’ from being oblate, the need for this was likely due to the low occupancy of the part.

Table S9: Crystal data and structure refinement for **4-H**.

CCDC number	2404932
Empirical formula	$C_{29}H_{38}NNiOP$
Formula weight	506.28
Temperature [K]	130(2)
Crystal system	orthorhombic
Space group (number)	$Pbca$ (61)
a [Å]	15.2370(9)
b [Å]	17.1607(12)
c [Å]	20.0226(14)
α [°]	90
β [°]	90
γ [°]	90
Volume [Å^3]	5235.5(6)
Z	8
ρ_{calc} [gcm^{-3}]	1.285
μ [mm^{-1}]	0.824
F(000)	2160
Crystal size [mm^3]	0.080×0.100×0.120
Crystal colour	red
Crystal shape	block
Radiation	MoK_{α} (λ=0.71073 Å)
2θ range [°]	4.11 to 50.74 (0.83 Å)
Index ranges	$-17 \leq h \leq 18$<br>$-20 \leq k \leq 20$<br>$-24 \leq l \leq 24$
Reflections collected	140299
Independent reflections	4810<br>R_{int} = 0.0515<br>R_{sigma} = 0.0135
Completeness to θ = 25.242°	100.0 %
Data / Restraints / Parameters	4810 / 145 / 340
Absorption correction T_{min}/T_{max} (method)	0.7091 / 0.7452 (multi-scan)
Goodness-of-fit on F^2	1.083
Final R indexes [$I \geq 2\sigma(I)$]	R_1 = 0.0326, wR_2 = 0.0795
Final R indexes [all data]	R_1 = 0.0389, wR_2 = 0.0838
Largest peak/hole [eÅ^{-3}]	0.90/–0.34

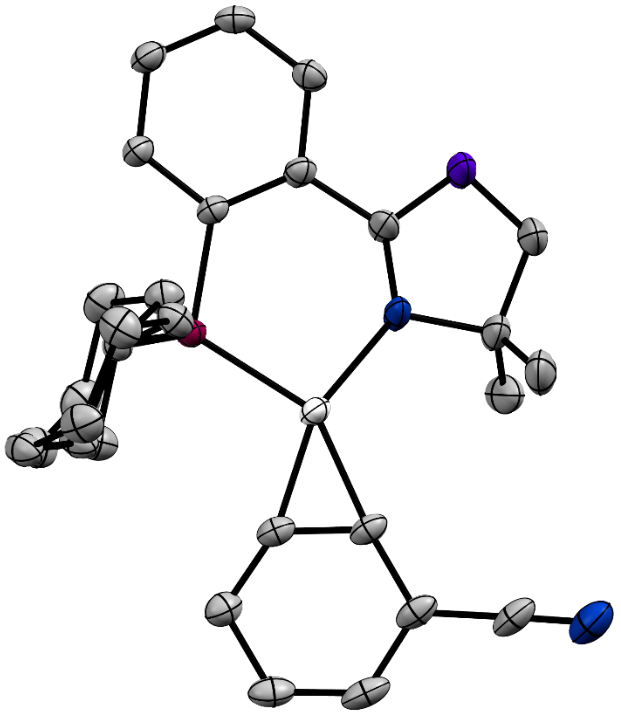

Figure S87: One of the molecules of **4-CN** present in the asymmetric unit of the crystal. Atoms are drawn as thermal ellipsoids at the 50% probability level. Hydrogen atoms are omitted for clarity. Only the majorly occupied disordered parts are shown.

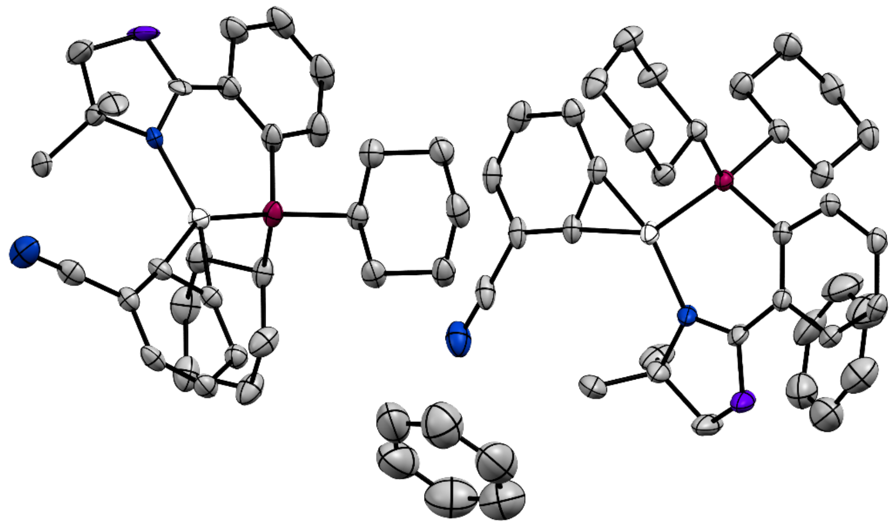

Figure S88: The asymmetric unit of **4-CN**. Atoms are drawn as thermal ellipsoids at the 50% probability level. Hydrogen atoms are omitted for clarity. Only the majorly occupied disordered parts are shown.

Refinement details for **4-CN**

The reflections 0 1 2 and 0 2 0 were omitted due to suspected interference from the beam stop.
The structure is an inversion twin with a twin ratio of approximately 55:45.
There is disorder present within the oxazoline ring of the ligand. This portion of the ligand was split into two parts, the lesser occupied part modeled using the SAME restraint to keep the geometry the same as the majorly occupied part. A RIGU restraint was applied to the atoms within each of the disordered parts. Due to their proximity in space, the EADP constraint was used on the following pairs of atoms: C26_1 and C26'_1, N2_1 and N2'_1, C28_1 and C28'_1, C30_1 and C30'_1, C26_2 and C26'_2, N2_2 and N2'_2, C28_2 and C28'_2, C30_2 and C30'_2. The occupancies of the disordered parts in each residue were initially refined using two independent free variables, however these converged to approximately the same value, so the occupancies were modeled using a single free variable. For each residue, the disordered parts have an occupancy ratio of approximately 52:48.
The asymmetric unit contains two co-crystallized benzene molecules. Restraints were applied to both molecules. A strong DFIX restraint of 1.38 Å was applied to each of the bonds in the benzene molecules. A strong SAME restraint was also used to assist in keeping the benzene molecules symmetrical. A RIGU restraint was applied to both molecules.

Table S10: Crystal data and structure refinement for **4-CN**.

CCDC number	2404935
Empirical formula	$C_{36}H_{43}N_2NiOP$
Formula weight	4875.21
Temperature [K]	130(2)
Crystal system	orthorhombic
Space group (number)	$Pca2_1$ (29)
a [Å]	17.6013(10)
b [Å]	17.6806(10)
c [Å]	20.5093(11)
α [°]	90
β [°]	90
γ [°]	90
Volume [Å^3]	6382.5(6)
Z	8
ρ_{calc} [gcm^{-3}]	1.268
μ [mm^{-1}]	0.688
F(000)	2592
Crystal size [mm^3]	0.100×0.110×0.150
Crystal colour	orange

Crystal shape	block
Radiation	MoK_α (λ=0.71073 Å)
2θ range [°]	3.82 to 56.58 (0.75 Å)
Index ranges	−23 ≤ h ≤ 23<br>−23 ≤ k ≤ 23<br>−27 ≤ l ≤ 27
Reflections collected	73999
Independent reflections	15830<br>R_{int} = 0.0457<br>R_{sigma} = 0.0370
Completeness to θ = 25.242°	99.9 %
Data / Restraints / Parameters	15830 / 335 / 827
Absorption correction T_{min}/T_{max} (method)	0.6722 / 0.7457 (multi-scan)
Goodness-of-fit on F^2	1.043
Final *R* indexes [$I \geq 2\sigma(I)$]	R_1 = 0.0326, wR_2 = 0.0785
Final *R* indexes [all data]	R_1 = 0.0412, wR_2 = 0.0838
Largest peak/hole [eÅ^{-3}]	0.52/−0.30
Flack X parameter	0.552(10)

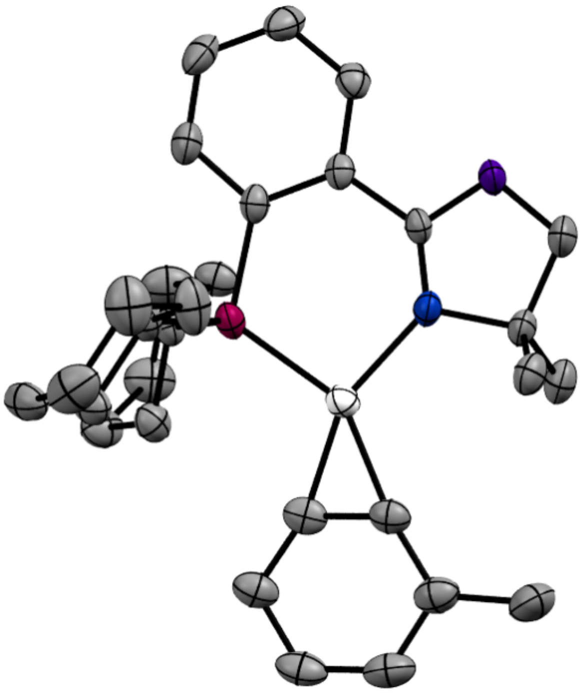

Figure S89: One of the molecules in the asymmetric unit of the crystal structure of **4-Me**. Atoms are drawn as thermal ellipsoids at the 50% probability level. Hydrogen atoms are omitted for clarity. Only the majorly occupied disordered parts are shown.

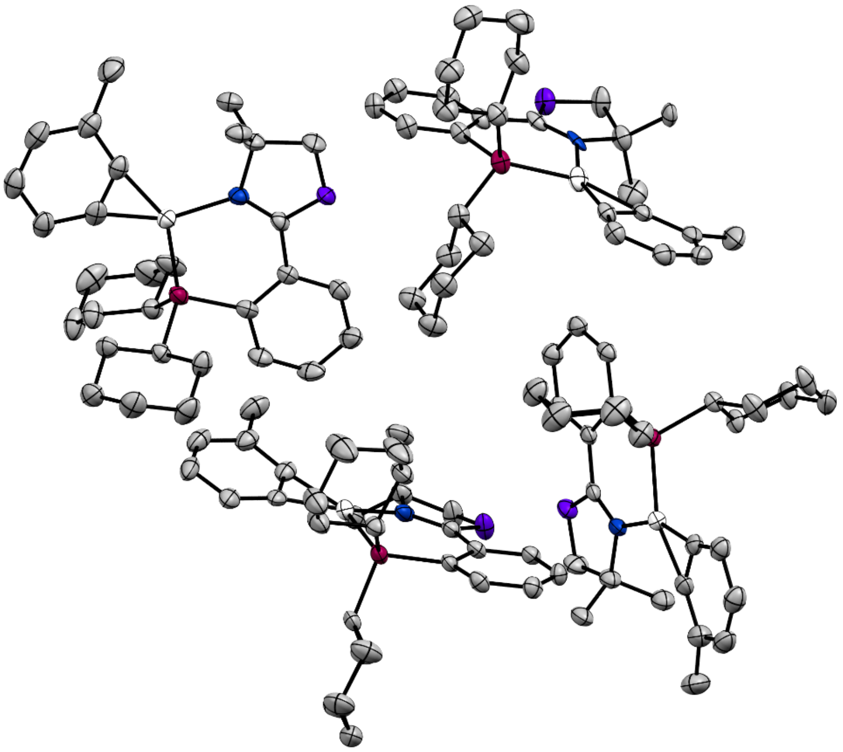

Figure S90: The asymmetric unit of the crystal structure of **4-Me**. Atoms are drawn as thermal ellipsoids at the 50% probability level. Hydrogen atoms are omitted for clarity. Only the majorly occupied disordered parts are shown.

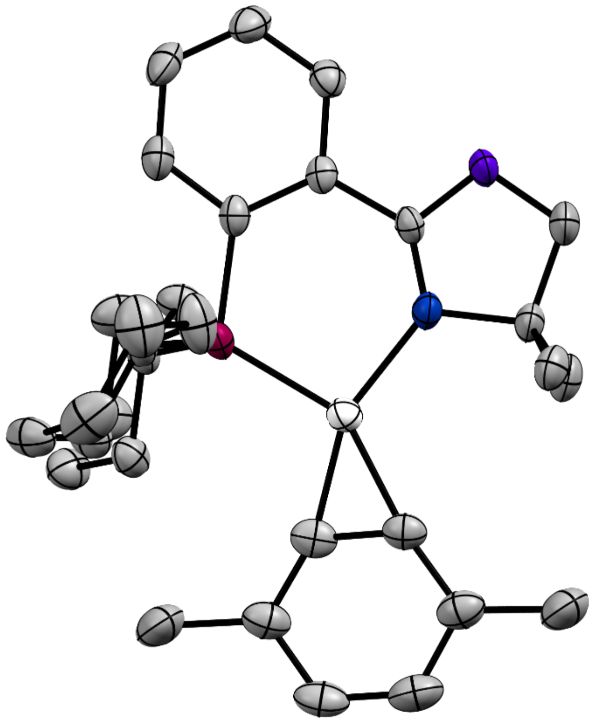

Figure S91: An image showing the first type of aryne disorder present in **4-Me**. In this case, the atoms in the ring are modeled in one position, while the methyl carbon is modeled in two positions. Atoms are drawn as thermal ellipsoids at the 50% probability level. Hydrogen atoms are omitted for clarity. Only the majorly occupied parts of the disordered cyclohexyl groups are shown.

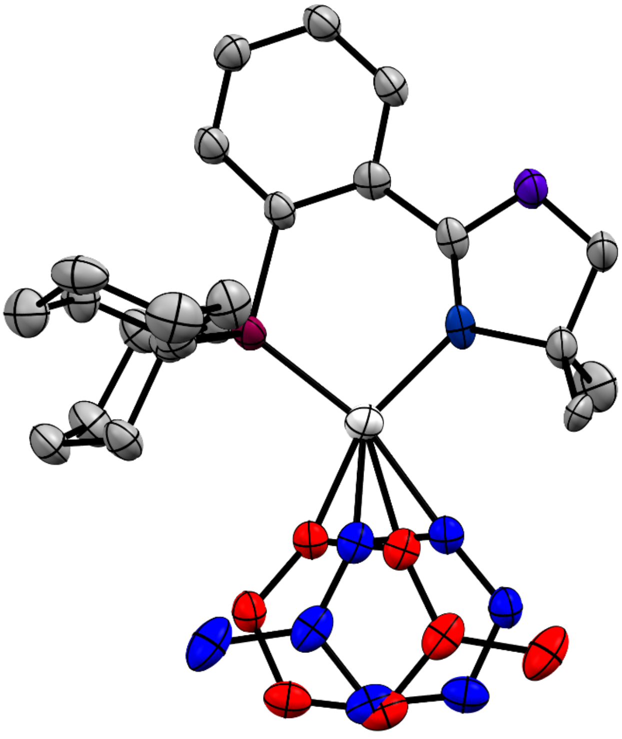

Figure S92: An image showing the second type of aryne disorder present in **4-Me**. In this case, the entire aryne unit is shifted with respect to the nickel atom. The two disordered parts have been colored red and blue. Atoms are drawn as thermal ellipsoids at the 50% probability level. Hydrogen atoms are omitted for clarity. Only the majorly occupied parts of the disordered cyclohexyl groups are shown.

Refinement details for **4-Me**

There is a systematic issue with the data for this crystal structure. The worst fitting reflections tend to have high values of K, with H and L often (but not always) related by a factor of 1, 2 or 0.5. For these reflections, the observed structure factor is much higher than the calculated structure factor. This does not appear to be due to twinning or modulation. The cause is unclear.

There is extensive disorder in the structure. There are four molecules in the asymmetric unit, and each has some level of disorder present. For all residues, the aryne is disordered. In all cases the disorder represents a rotation such that the methyl group in the minorly occupied part is pointed in the opposite direction as that of the majorly occupied part. This manifested itself in two ways. In residues 1 and 3, the ring portion of the aryne is fully occupied, with the methyl carbon C7_1/3, C7_1/3' and aromatic hydrogens H3_1/3, H6'_1/3 being modeled as partially occupied (Figure S16). Because of the low occupancy for the minorly disordered parts, the EADP constraint was used for C7_1/3 and C7'_1/3. For residue 3, strong SADI restraints were applied to C7_3 and C7'_3 to ensure the same geometry in both major and minorly occupied parts. In residues 2 and 4, the entire minorly occupied aryne has shifted relative to the majorly occupied part (Figure S17). Strong SAME restraints were applied to the minorly occupied parts to ensure geometry matches with the majorly occupied parts. Because of the close proximity of the atoms and the low occupancies of the minorly occupied parts, the EADP constraint

was used on the following pairs of atoms: C1_2/4 and C2'_2/4, C2_2/4 and C1'_2/4, C3_2/4 and C6'_2/4, C4_2/4 and C5'_2/4, C5_2/4 and C4'_2/4, C6_2/4 and C3'_2/4, C7_2/4 and C7'_2/4. Initially, the occupancies of the aryne disorder were refined using independent free variables, but during refinement these converged to similar values, so the occupancies were refined to a single free variable. The occupancy ratio is approximately 88:12. In addition to the aryne disorder, residues 1 and 3 have disorder in the cyclohexyl groups. For residue 1, both cyclohexyl groups are disordered. For the ring consisting of C19_1 > C24_1, it was modeled in two parts, with strong SAME restraints applied to the minorly occupied part to keep the geometry the same as the majorly occupied part. A RIGU restraint was applied to both the major and minorly occupied parts. Due to their proximity in space and low occupancy of the minor part, the EADP constraint was applied to the following pairs of atoms: C19_1 and C19'_1, C20_1 and C20'_1, C23_1 and C23'_1, C23_1, C24_1 and C24'_1. The cyclohexyl group consisting of C25_1 > C30_1 is also modeled in two parts. Strong SAME restraints were used to keep the geometry of the parts consistent. A strong SADI restraint was used to keep the distances between P1_1 and C25_1 and P1_1 and C25'_1 the same. A RIGU restraint was applied to both parts. The EADP constraint was used on the following pairs of atoms: C25_1 and C25'_1, C29_1 and C29'_1, C30_1 and C30'_1. The occupancies of both cyclohexyl groups refined to the same values as the disordered aryne groups, with a ratio of approximately 88:12. In residue 3, the cyclohexyl group consisting of C19_3 > C24_3 was modeled in two parts. A SADI restraint was used to ensure that the distances between P1_3 and C19_3 and P1_3 and C19'_3 are the same. A SAME restraint was used to ensure the geometries of the parts were consistent. A RIGU restraint was applied to both parts. The EADP constraint was applied to C19_3 and C19'_3 because of their proximity in space. The occupancy ratio is approximately 63:37. The final bit of disorder is present in the oxazoline portion of the PHOX ligand in residue 2. The oxazoline ring was split into two parts, with a strong SAME restraint being used to ensure the geometry of the minorly occupied part matches the majorly occupied part. Additionally, a strong SAME restraint was used on the majorly occupied part to ensure its geometry matched that of a non-disordered ligand, with the ligand in residue 3 being used as the model. A RIGU restraint was applied to both the major and minor occupied parts. A strong SADI restraint was applied to ensure that C14_2 and C14'_2 had the same distance from C13_2.The EADP constraint was applied to the following pairs of atoms due to their proximity in space: C14_2 and C14'_2, O1_2 and O1'_2, N1_2 and N1'_2, C15_2 and C15'_2, C16_2 and C16'_2, C17_2 and C17'_2, C18_2 and C18'_2. As a result of the disorder modeling, the value for R_1 [$I \geq 2\sigma(I)$] went from 8.48% to 5.91%.

Table S11: Crystal data and structure refinement for **4-Me**.

CCDC number	2404933
Empirical formula	$C_{30}H_{40}NNiOP$
Formula weight	520.31
Temperature [K]	100(2)
Crystal system	monoclinic
Space group (number)	$P2_1/c$ (14)
a [Å]	29.300(4)
b [Å]	15.072(2)
c [Å]	26.628(4)
α [°]	90
β [°]	111.800(4)
γ [°]	90
Volume [Å^3]	10918(3)
Z	16
ρ_{calc} [gcm^{-3}]	1.266
μ [mm^{-1}]	0.792
F(000)	4448
Crystal size [mm^3]	0.100×0.100×0.100
Crystal colour	red
Crystal shape	block
Radiation	MoK_α (λ=0.71073 Å)
2θ range [°]	3.75 to 52.83 (0.80 Å)
Index ranges	–36 ≤ h ≤ 34<br>–18 ≤ k ≤ 18<br>–33 ≤ l ≤ 33
Reflections collected	100811
Independent reflections	22390<br>R_{int} = 0.0547<br>R_{sigma} = 0.0473
Completeness to θ = 25.242°	100.0 %
Data / Restraints / Parameters	22390/1021/1429
Absorption correction T_{min}/T_{max} (method)	0.6462/0.7454 (multi-scan)
Goodness-of-fit on F^2	1.221
Final *R* indexes [I≥2σ(I)]	R_1 = 0.0591, wR_2 = 0.1173
Final *R* indexes [all data]	R_1 = 0.0878, wR_2 = 0.1267
Largest peak/hole [eÅ^{-3}]	1.51/–1.14

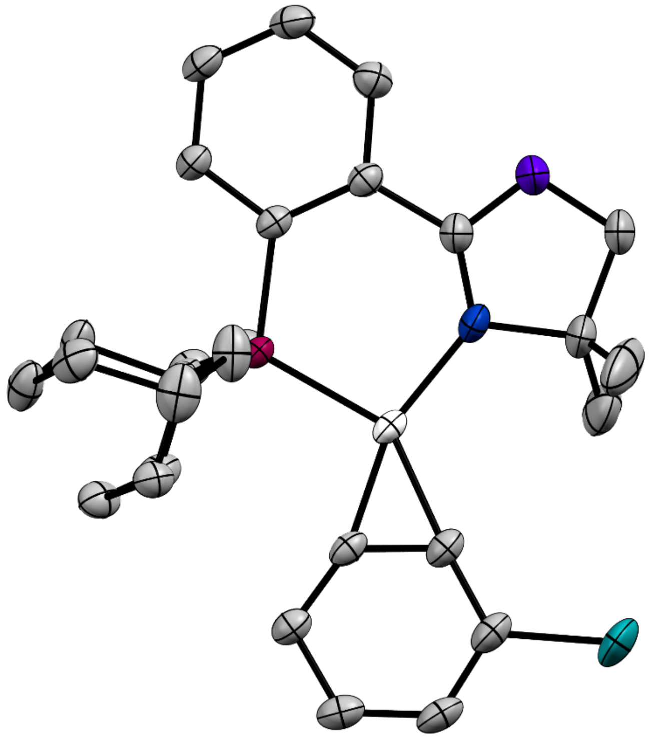

Figure S93: The crystal structure of one of the molecules of **4-Cl** present in the asymmetric unit. Atoms are drawn as thermal ellipsoids at the 50% probability level. Hydrogen atoms are omitted for clarity. Only the majorly occupied disordered parts are shown.

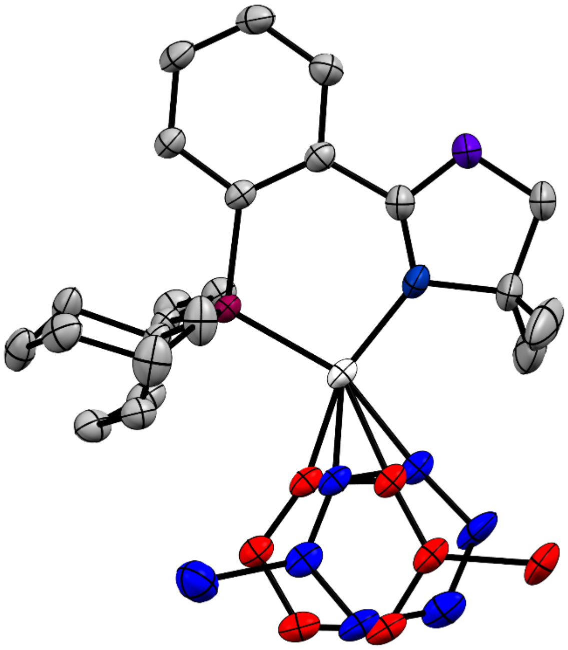

Figure S94: The crystal structure of one of the molecules of **4-Cl** present in the

asymmetric unit showing the aryne disorder. The two partially occupied arynes are colored red and blue. Atoms are drawn as thermal ellipsoids at the 50% probability level. Hydrogen atoms are omitted for clarity. Only the majorly occupied parts of the disordered cyclohexyl group are shown.

Refinement Details for **4**-Cl

The reflection 1 0 1 was omitted due to suspected interference from the beam stop. There was what appeared to be a disordered pentane molecule in the asymmetric unit. Attempts to model the molecule were unsuccessful, so the SQUEEZE function of PLATON[18] was used to remove the electron density associated with the solvent molecule. Within the asymmetric unit, SQUEEZE found a void of approximately 177 cubic angstroms containing 85 electrons.

There is a large amount of disorder present in the structure. Like compound **4**-Me, the aryne group is disordered on both molecules in the asymmetric unit. The disorder present on the aryne groups of **4**-Cl here is the same type as in Residues 2 and 4 of **4**-Me (**Figure S92**) and the disorder modeling was handled in a similar manner. A SAME restraint was applied to the minorly occupied aryne groups to ensure the same geometry as the majorly occupied part. A RIGU restraint was applied to the majorly and minorly occupied parts. Due to their proximity in space, the EADP constraint was applied to the following pairs of atoms: C1_1 and C2'_1, C2_1 and C1'_1, C5_1 and C5'_1, C1_2 and C2'_2, C2_2 and C1'_2, C5_2 and C5'_2. Initially, the occupancies of the disordered aryne parts were modeled using separate free variables, however during refinement the values converged to a similar number, so a single free variable was used to model all the aryne disorder. The ratio of major and minorly occupied parts is approximately 91:9. The cyclohexyl ring consisting of C13_2 through C18_2 is disordered and modeled in two parts using C13_2 as a pivot atom. The EADP and EXYZ constraints were applied to C13_2 and C13'_2. A SAME restraint was applied to the minorly occupied parts to ensure the same geometry as the majorly occupied part. A RIGU restraint was applied to both the majorly and minorly occupied parts. For the disordered cyclohexyl group, the ratio of the disordered parts is approximately 86:14. The final bit of disorder present in the structure is "envelope flap" disorder of C26_1 in the oxazoline ring of residue 1. C26_1 was split into two parts, with strong SADI restraints applied to ensure the same distances from O1_1 and C27_1 to C26_1 and C26'_1. The ratio of disordered parts is approximately 68:32.

Table S12: Crystal data and structure refinement for **4-Cl**.

CCDC number	2404930
Empirical formula	$C_{61}H_{77}Cl_2N_2Ni_2O_2P2$[+solvent]
Formula weight	1120.50
Temperature [K]	100(2)
Crystal system	triclinic
Space group (number)	$P\overline{1}$ (2)
a [Å]	10.5619(12)
b [Å]	17.572(2)
c [Å]	17.848(2)
α [°]	76.291(3)
β [°]	72.971(4)
γ [°]	81.654(3)
Volume [Å^3]	3066.8(6)
Z	2
ρ_{calc} [gcm^{-3}]	1.213
μ [mm^{-1}]	0.794
F(000)	1186
Crystal size [mm^3]	0.050×0.120×0.150
Crystal colour	red
Crystal shape	plate
Radiation	MoK_α (λ=0.71073 Å)
2θ range [°]	3.75 to 56.62 (0.75 Å)
Index ranges	−13 ≤ h ≤ 14<br>−23 ≤ k ≤ 23<br>−23 ≤ l ≤ 23
Reflections collected	100497
Independent reflections	15229<br>R_{int} = 0.0519<br>R_{sigma} = 0.0347
Completeness to θ = 25.242°	100.0 %
Data / Restraints / Parameters	15229 / 304 / 791
Absorption correction T_{min}/T_{max} (method)	0.7008 / 0.7457 (multi-scan)
Goodness-of-fit on F^2	1.022
Final *R* indexes , [I≥2σ(I)]	R_1 = 0.0389, wR_2 = 0.0878
Final *R* indexes, [all data]	R_1 = 0.0561, wR_2 = 0.0969
Largest peak/hole [eÅ^{-3}]	1.26/−0.88

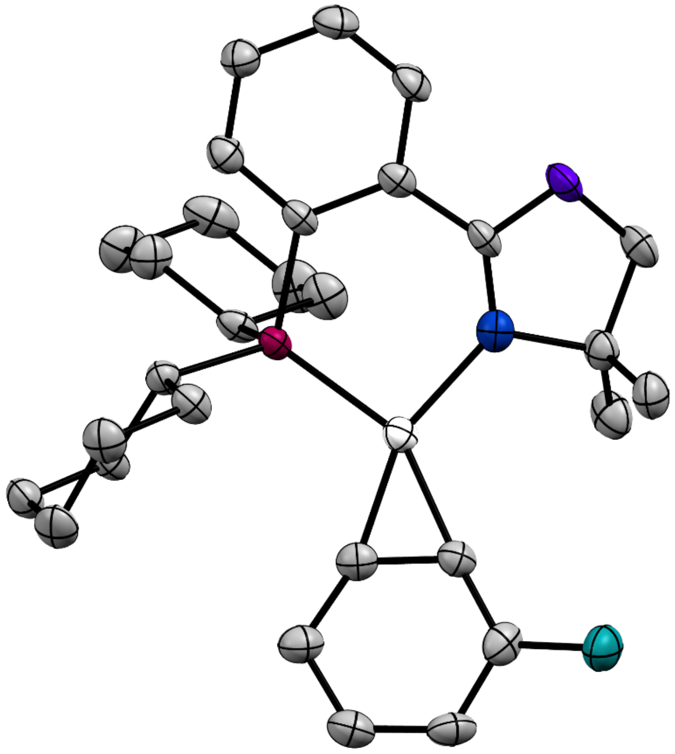

Figure 95: The crystal structure of **4-F**. Atoms are drawn as thermal ellipsoids at the 50% probability level. Hydrogen atoms are omitted for clarity.

Table S13: Crystal data and structure refinement for **4-F**.

CCDC number	2404931
Empirical formula	$C_{29}H_{37}FNNiOP$
Formula weight	524.27
Temperature [K]	130(2)
Crystal system	monoclinic
Space group (number)	Cc (9)
a [Å]	10.0154(17)
b [Å]	15.956(3)
c [Å]	16.948(3)
α [°]	90
β [°]	106.171(4)
γ [°]	90
Volume [Å^3]	2601.2(8)
Z	4
ρ_{calc} [gcm^{-3}]	1.339
μ [mm^{-1}]	0.837
F(000)	1112
Crystal size [mm^3]	0.400×0.300×0.200
Crystal colour	red
Crystal shape	block
Radiation	MoK_{α} (λ=0.71073 Å)
2θ range [°]	4.94 to 52.79 (0.80 Å)
Index ranges	$-12 \leq h \leq 12$<br>$-19 \leq k \leq 19$<br>$-21 \leq l \leq 21$
Reflections collected	22053
Independent reflections	5323<br>R_{int} = 0.0505<br>R_{sigma} = 0.0483
Completeness to θ = 25.242°	100.0 %
Data / Restraints / Parameters	5323/2/309
Absorption correction T_{min}/T_{max} (method)	0.6148/0.7454 (multi-scan)
Goodness-of-fit on F^2	1.079
Final R indexes [$I \geq 2\sigma(I)$]	R_1 = 0.0465, wR_2 = 0.1088
Final R indexes [all data]	R_1 = 0.0557, wR_2 = 0.1144
Largest peak/hole [eÅ^{-3}]	1.09/–0.48
Flack X parameter	–0.013(8)

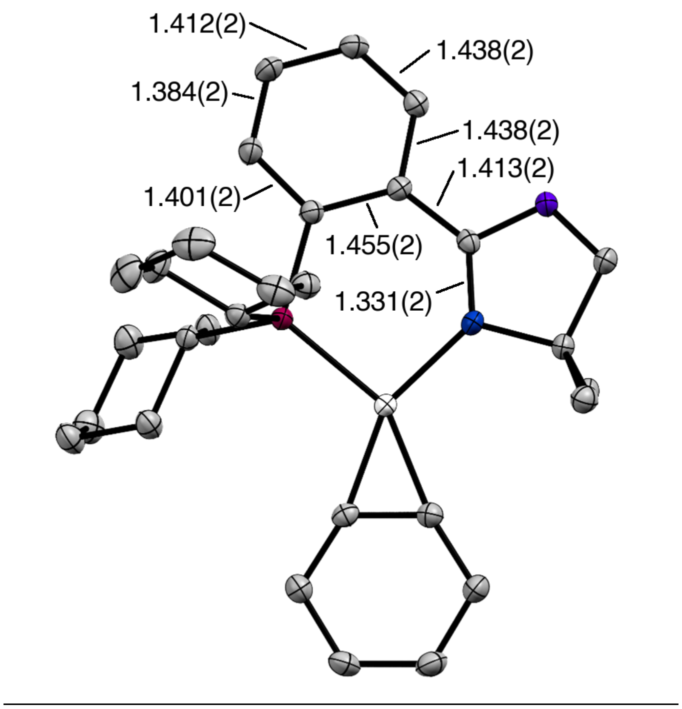

Figure S96: The crystal structure of the **5-H** anion, with alternating bond lengths shown in Å. Atoms are drawn as thermal ellipsoids at the 50% probability level. Hydrogen atoms are omitted for clarity.

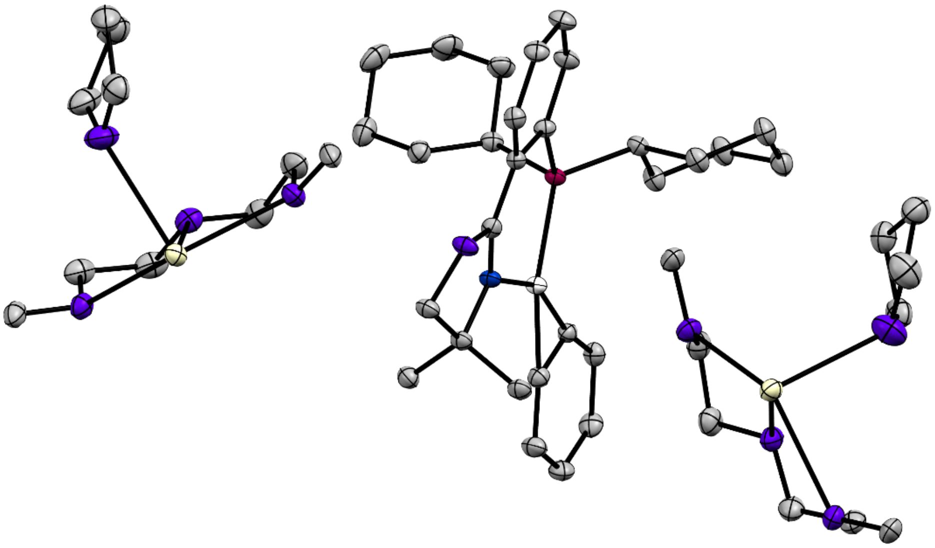

Figure S97: The asymmetric unit of **5-H**. The potassium atoms (shown in olive) are located on inversion centers and are half occupied within the asymmetric unit. Atoms are drawn as thermal ellipsoids at the 50% probability level. Hydrogen atoms are omitted for clarity. Only the majorly occupied disordered parts are shown.

Refinement details for **5-H**

The reflections 0 1 1 and 0 -1 1 were omitted due to suspected interference from the beam stop.

There is some minor “envelope flap” disorder present in both THF molecules. Each THF was modeled using oxygen as a pivot atom, with the EADP and EXYZ constraints being applied to the following pairs of atoms: O8 and O8’, O9 and O9’. Because of their proximities in space, the EADP constraint was applied to the following pairs of atoms: C42 and C42’, C43 and C43’, C45 and C45’, C46 and C46’, C47 and C47’, C48 and C48’, C49 and C49’. A strong SAME restraint was applied to the minorly occupied THF parts to ensure the same geometry as the majorly occupied portions. Initially, the occupancies of each disordered THF were modeled using two different free variables, however during refinement these converged to the same value. As such, the occupancies of both disordered THF molecules are modeled using a single free variable and having approximate occupancies of 81:19 for the majorly and minorly occupied components.

Table S14: Crystal data and structure refinement for **5-H**.

CCDC number	2404934
Empirical formula	$C_{49}H_{78}KNNiO_9P$
Formula weight	953.90
Temperature [K]	100(2)
Crystal system	triclinic
Space group (number)	$P\overline{1}$ (2)
a [Å]	10.232(2)
b [Å]	10.3984(18)
c [Å]	23.777(5)
α [°]	88.388(10)
β [°]	77.866(7)
γ [°]	84.204(7)
Volume [Å^3]	2460.6(9)
Z	2
ρ_{calc} [gcm^{-3}]	1.287
μ [mm^{-1}]	0.565
F(000)	1026
Crystal size [mm^3]	0.100×0.100×0.100
Crystal colour	red

Crystal shape	plate
Radiation	MoK_α (λ=0.71073 Å)
2θ range [°]	3.94 to 52.90 (0.80 Å)
Index ranges	$-12 \leq h \leq 12$<br>$-12 \leq k \leq 13$<br>$-29 \leq l \leq 29$
Reflections collected	75473
Independent reflections	10108<br>R_{int} = 0.0458<br>R_{sigma} = 0.0268
Completeness to θ = 25.242°	99.9 %
Data / Restraints / Parameters	10108/20/596
Absorption correction T_{min}/T_{max} (method)	0.7111/0.7454 (multi-scan)
Goodness-of-fit on F^2	1.020
Final R indexes [$I \geq 2\sigma(I)$]	R_1 = 0.0294, wR_2 = 0.0649
Final R indexes [all data]	R_1 = 0.0410, wR_2 = 0.0702
Largest peak/hole [eÅ^{-3}]	0.41/–0.24

UV–vis Spectroscopy

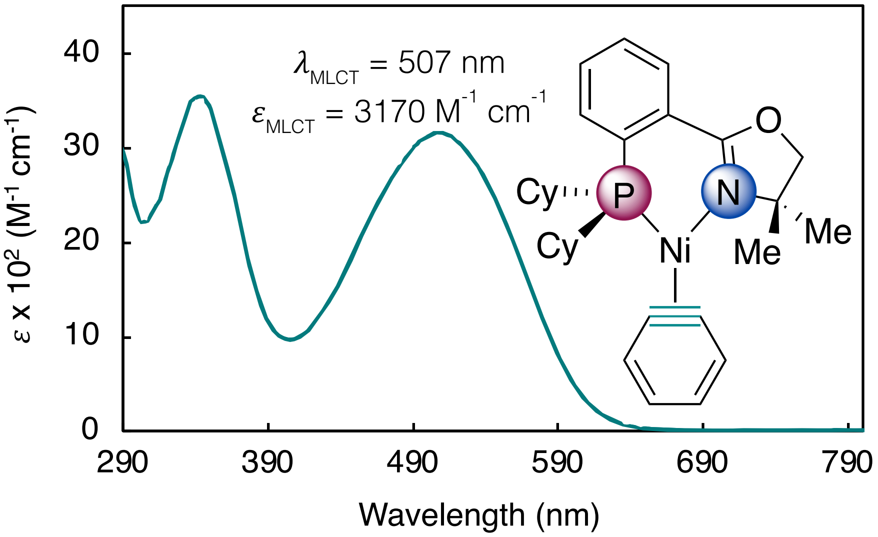

Figure S98. UV–vis/NIR spectra of 0.3 mM THF solution of complex **4–H** at 298 K.

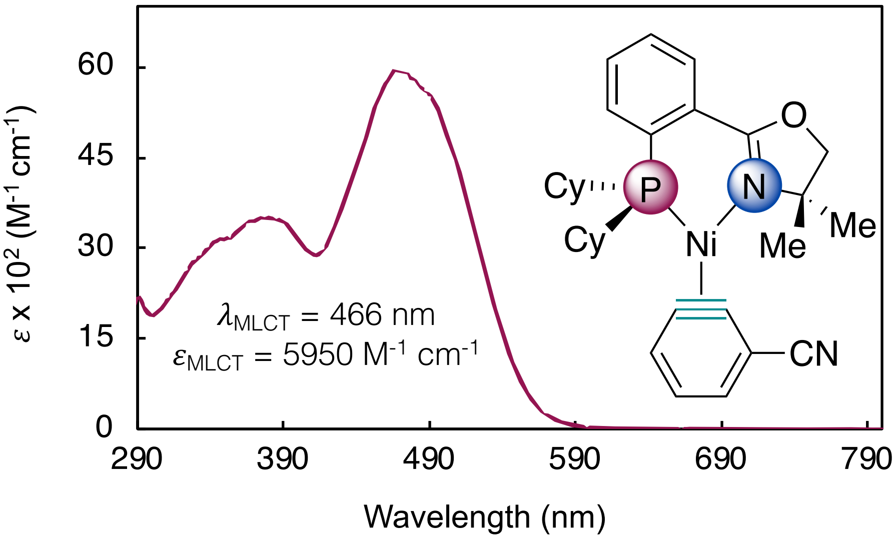

Figure S99. UV–vis/NIR spectra of 0.3 mM THF solution of complex **4–CN** at 298 K.

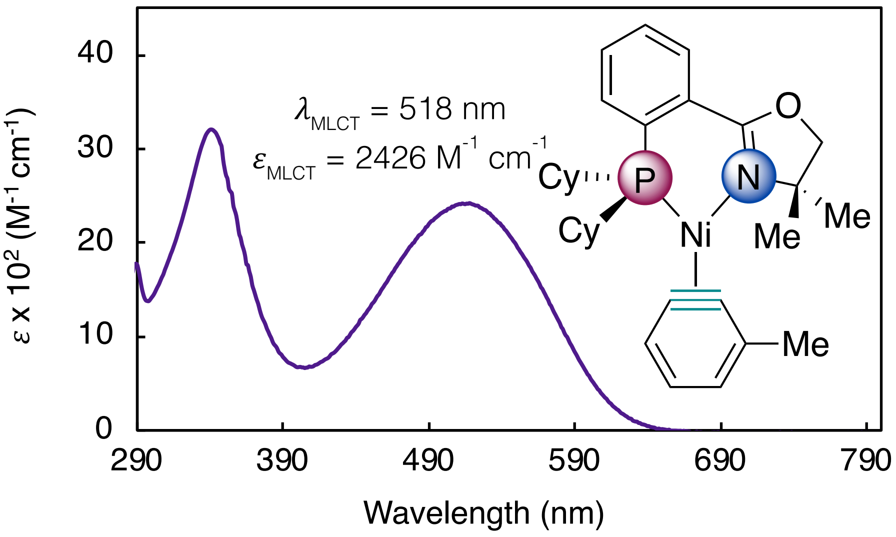

Figure S100. UV–vis/NIR spectra of 0.3 mM THF solution of complex **4–Me** at 298 K.

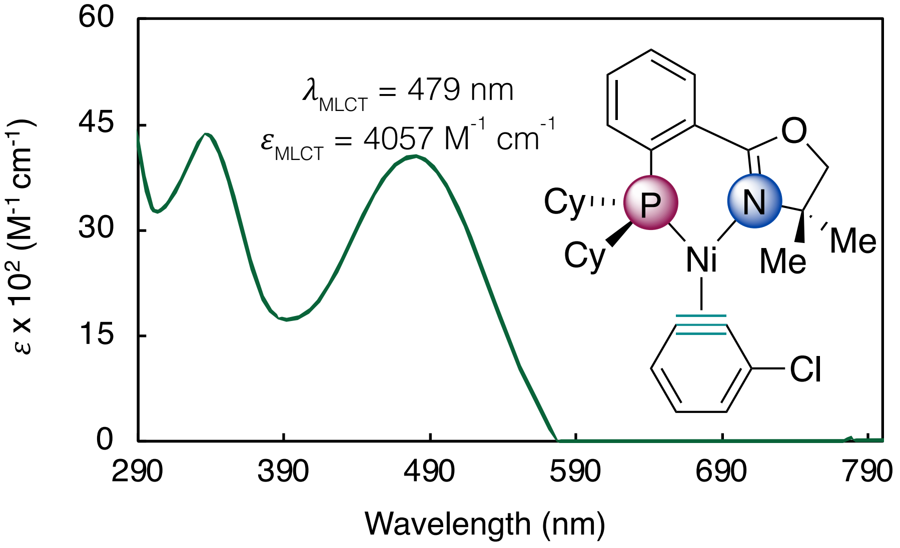

Figure S101. UV–vis/NIR spectra of 0.3 mM THF solution of complex **4–Cl** at 298 K.

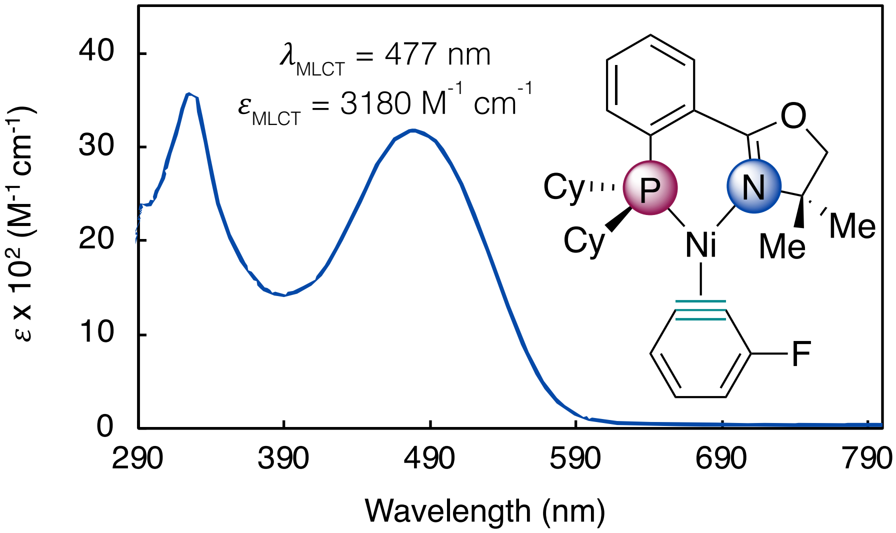

Figure S102. UV–vis/NIR spectra of 0.3 mM THF solution of complex **4–F** at 298 K.

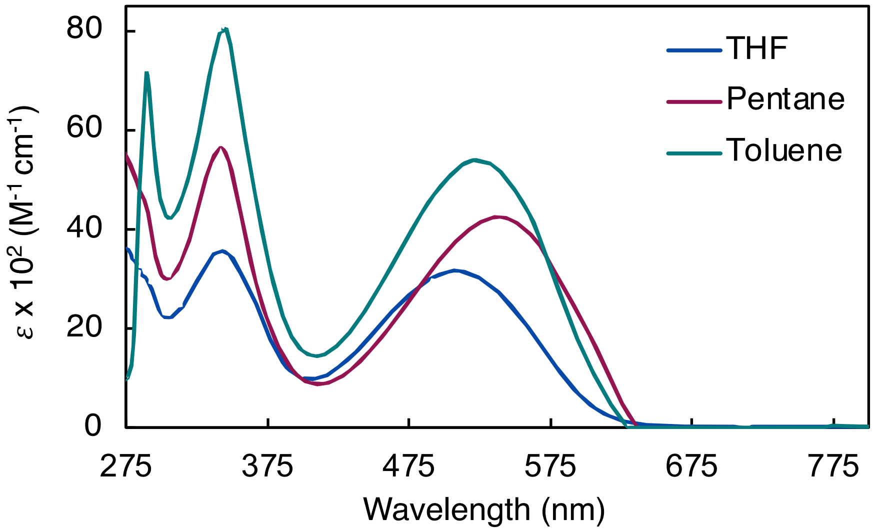

Figure S103. UV–vis spectra of complex **4–H** in various solvents (0.3 mM) under Ar at 298 K.

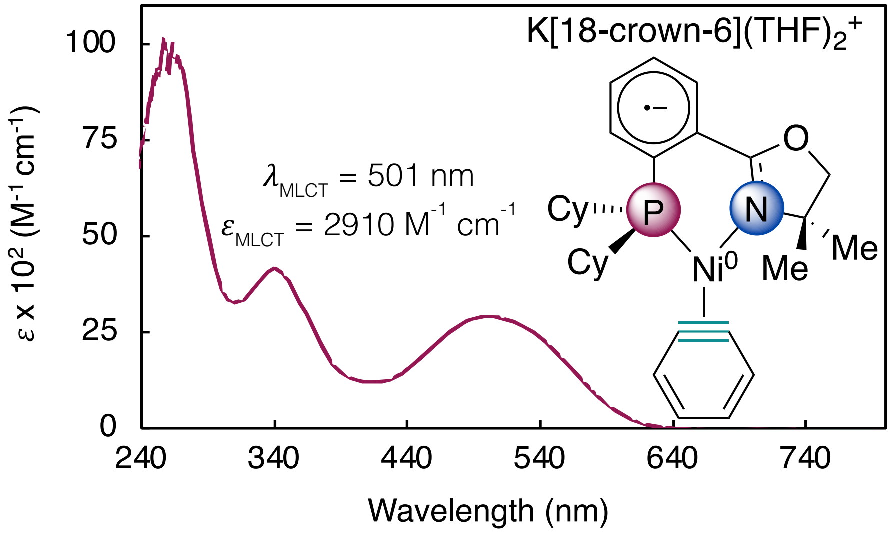

Figure S104. UV–vis/NIR spectra of 0.3 mM THF solutions of complex **5-H** at 298 K.

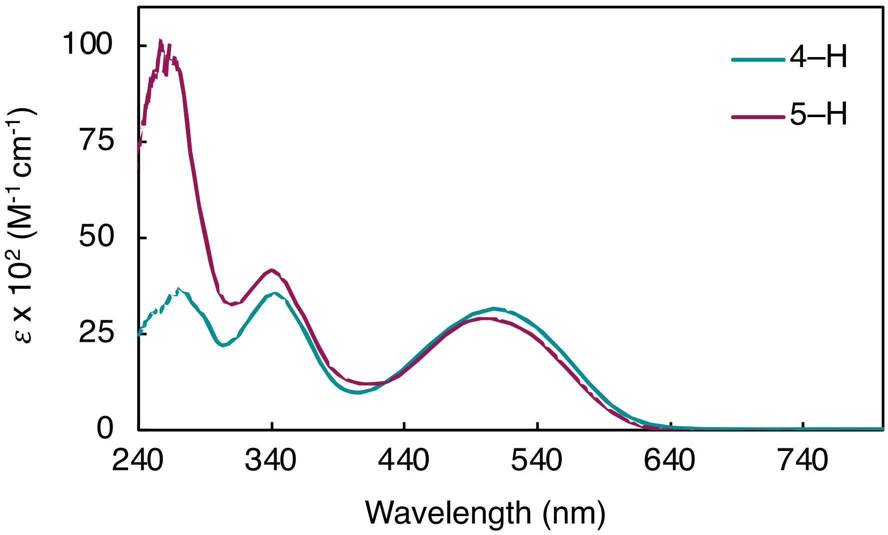

Figure S105. Overlay UV–vis spectra of complexes **4–H** and **5–H** in THF solutions under Ar at 298 K.

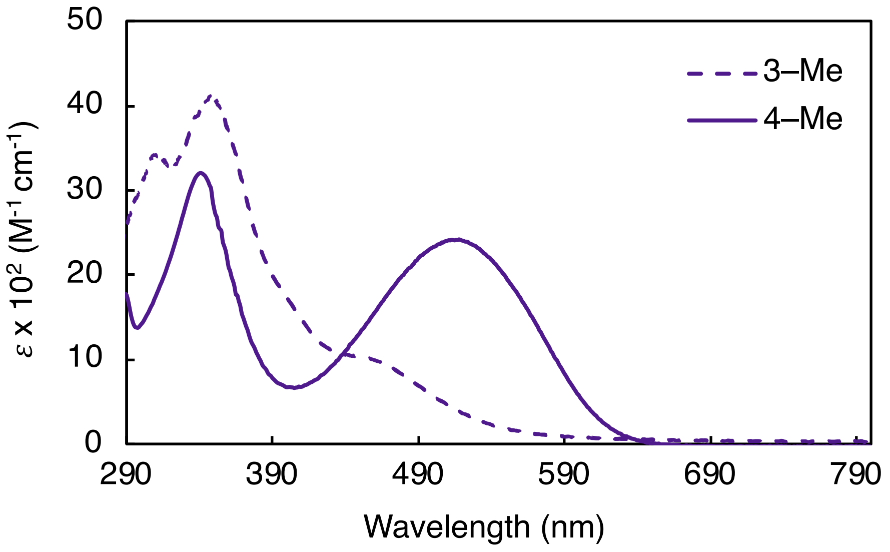

Figure S106. Overlay UV–vis spectra of complexes **3–Me** and **4–Me** in 0.3 mM THF solutions under Ar at 298 K.

Cyclic Voltammetry

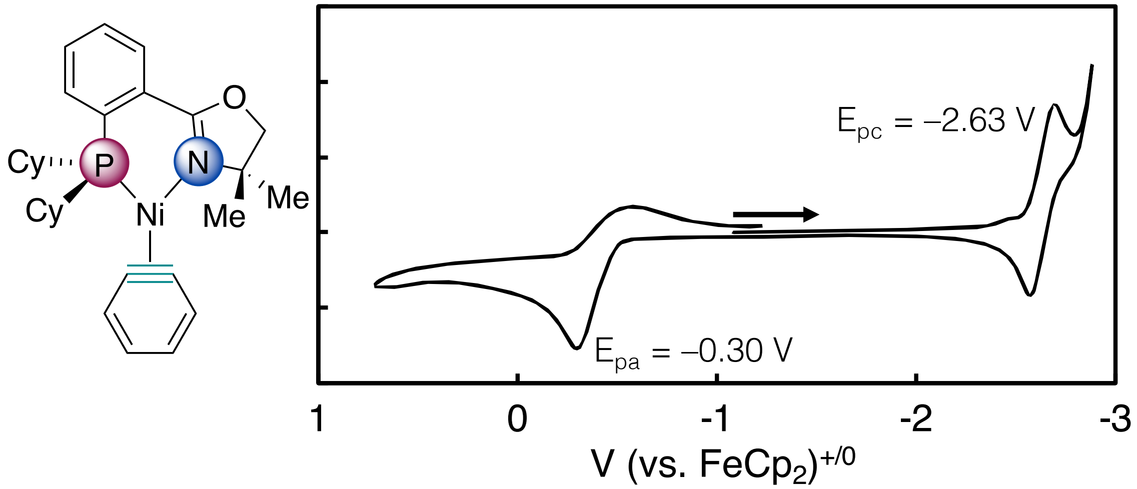

Figure S107. Full window cyclic voltammogram for **4–H** collected in 0.1 M $[^nPr_4N]BAr^F_4$ THF solutions with 3 mM analyte at 100 mV/s under Ar and ambient temperature. Ar^F: 3,5–bis(trifluoromethyl)phenyl.

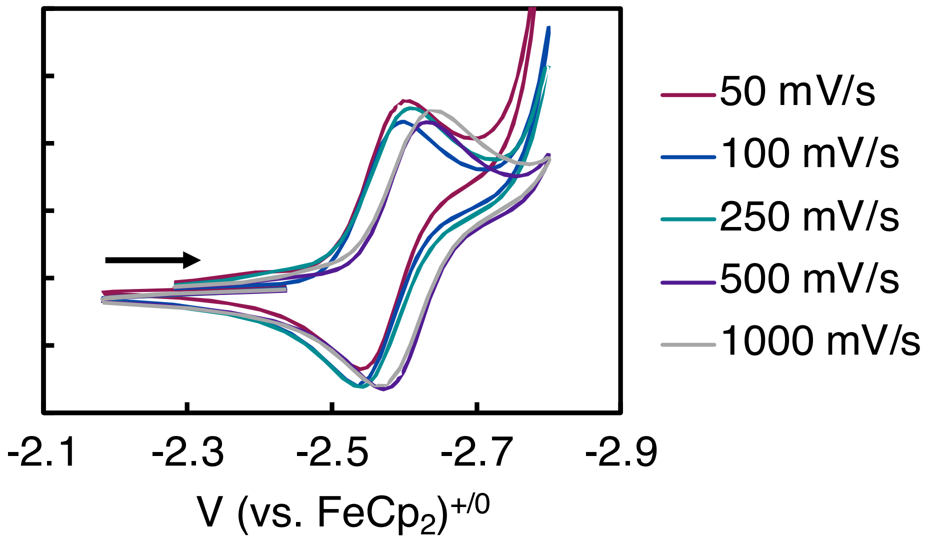

Figure S108. Scan rate dependence studies on E_{pc} for complex **4–H** in 0.1 M [^{n}Pr$_4$N]BAr$^F{}_4$ THF solutions with 3 mM analyte under Ar at ambient temperatures. Ar^F: 3,5–bis(trifluoromethyl)phenyl. Peak current potentials are normalized.

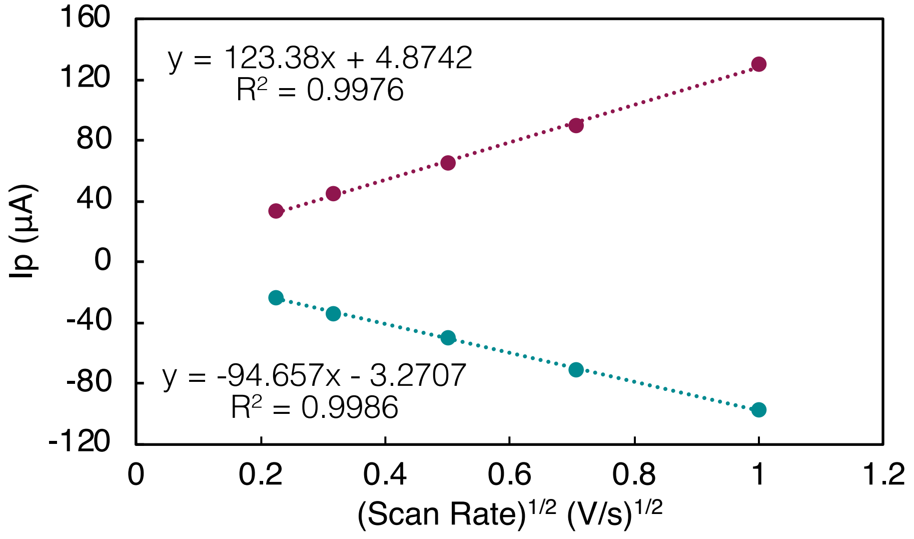

Figure S109. Anodic (I_{pa}) and cathodic (I_{pc}) peak current plotted against the square root of the scan rate $(V/s)^{1/2}$ of the observed quasi-reversible reduction E_{pc} in **4–H.**

Table S15. Anodic and cathodic peak currents for E_{pc} and ratios from scan rate dependence studies on complex **4–H**.

Scan Rate (V/s)	I_{pa} (μA)	I_{pc} (μA)	$\|I_{pc}/I_{pa}\|$
0.05	–23.34	33.67	0.69
0.10	–34.55	44.79	0.77
0.25	–49.83	64.80	0.77
0.50	–71.28	89.83	0.79
1.00	–97.37	130.20	0.75

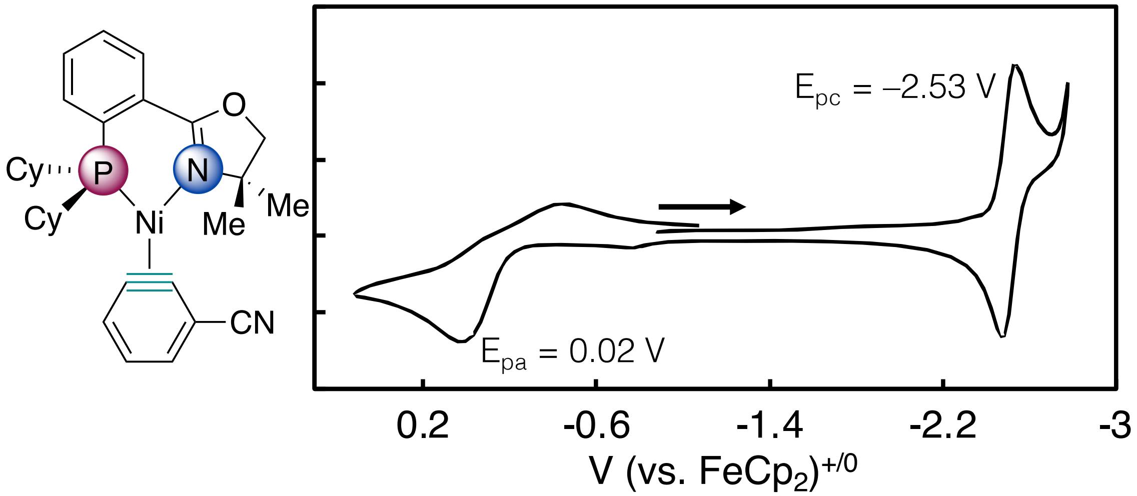

Figure S110. Full window cyclic voltammogram for **4–CN** collected in 0.1 M [$^nPr_4N$]BAr^F_4 THF solutions with 3 mM analyte at 100 mV/s under Ar and ambient temperature. Ar^F: 3,5–bis(trifluoromethyl)phenyl.

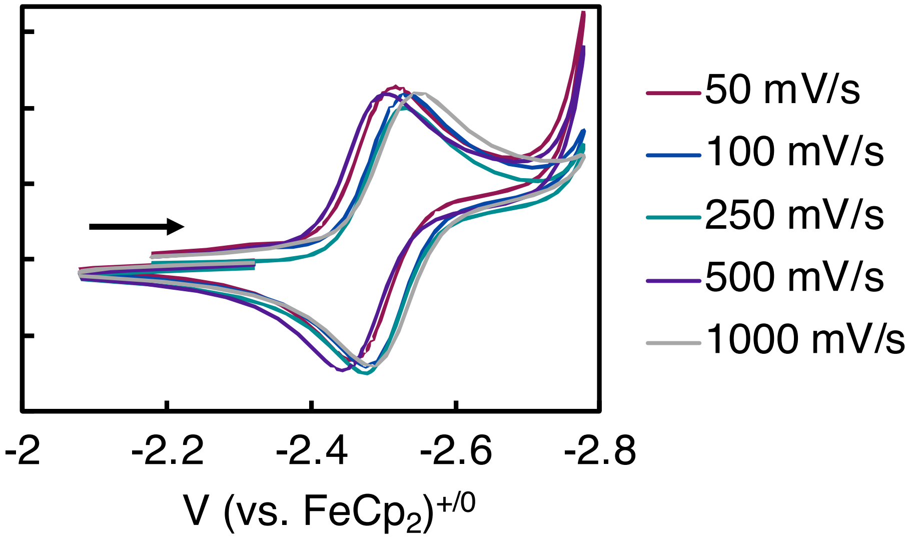

Figure S111. Scan rate dependence studies on E_{pc} for complex **4–CN** in 0.1 M $[^nPr_4N]BAr^F_4$ THF solutions with 3 mM analyte under Ar at ambient temperatures. Ar^F: 3,5–bis(trifluoromethyl)phenyl. Peak current potentials are normalized.

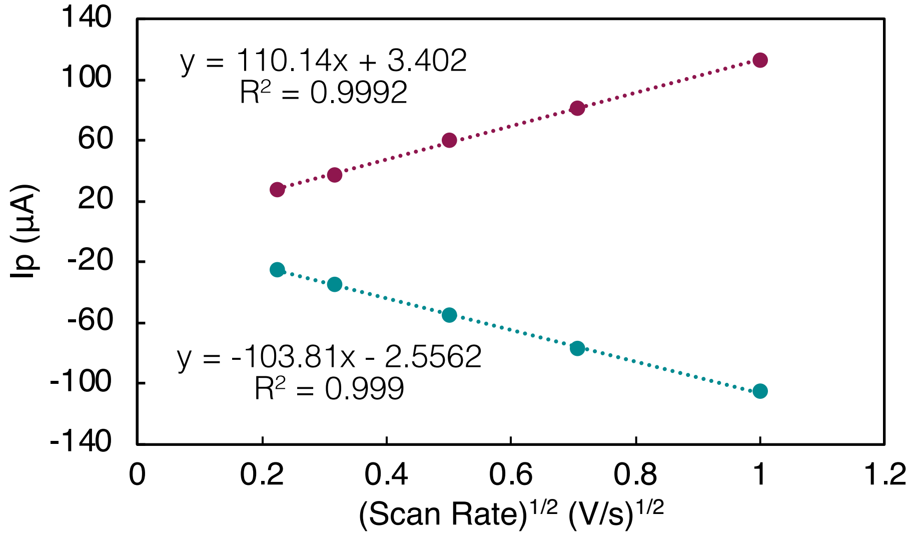

Figure S112. Anodic (I_{pa}) and cathodic (I_{pc}) peak current plotted against the square root of the scan rate $(V/s)^{1/2}$ of the observed quasi-reversible reduction E_{pc} in **4–CN.**

Table S16. Anodic and cathodic peak currents for E_{pc} and ratios from scan rate dependence studies on complex **4–CN**.

Scan Rate (V/s)	I_{pa} (μA)	I_{pc} (μA)	$\|I_{pc}/I_{pa}\|$
0.05	–25.27	27.63	0.91
0.10	–34.81	37.42	0.93
0.25	–55.20	60.17	0.92
0.50	–77.35	81.33	0.95
1.00	–105.30	113.00	0.93

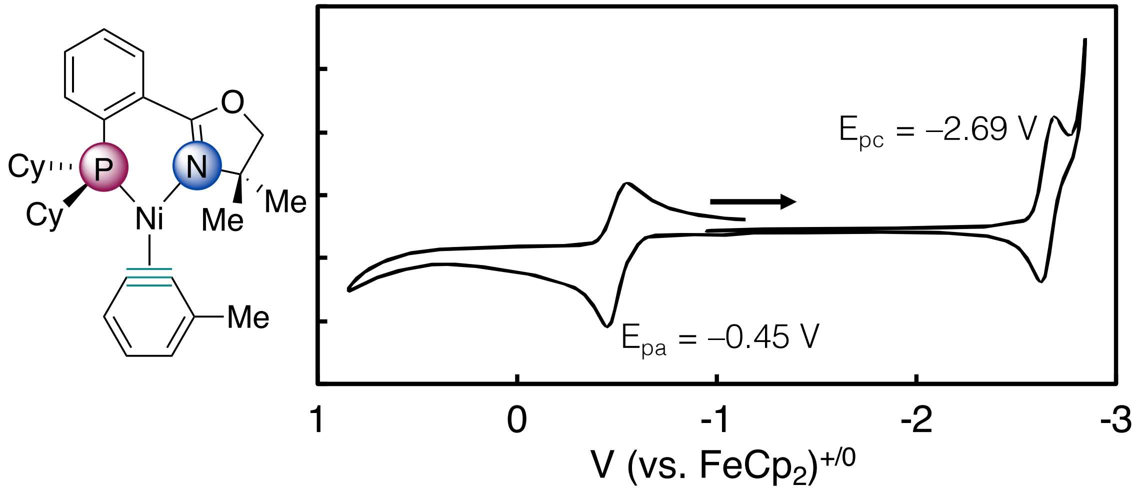

Figure S113. Full window cyclic voltammogram for **4–Me** collected in 0.1 M [$^{n}Pr_4N$]BAr^F_4 THF solutions with 3 mM analyte at 100 mV/s under Ar and ambient temperature. Ar^F: 3,5–bis(trifluoromethyl)phenyl.

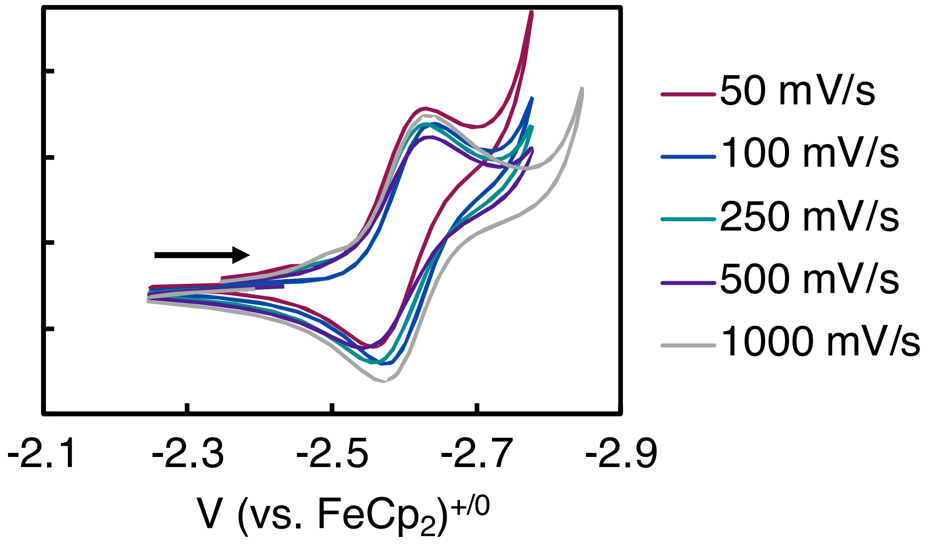

Figure S114. Scan rate dependence studies on E_{pc} for complex **4–Me** in 0.1 M $[^nPr_4N]BAr^F_4$ THF solutions with 3 mM analyte under Ar at ambient temperatures. Ar^F: 3,5–bis(trifluoromethyl)phenyl. Peak current potentials are normalized.

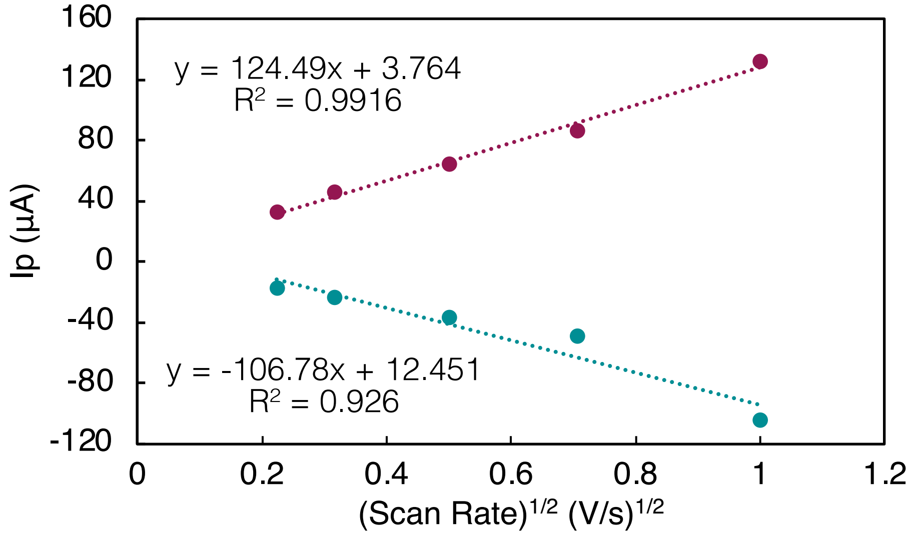

Figure S115. Anodic (I_{pa}) and cathodic (I_{pc}) peak current plotted against the square root of the scan rate $(V/s)^{1/2}$ of the observed quasi-reversible reduction E_{pc} in **4–Me.**

Table S17. Anodic and cathodic peak currents for E_{pc} and ratios from scan rate dependence studies on complex **4–Me**.

Scan Rate (V/s)	I_{pa} (μA)	I_{pc} (μA)	$\|I_{pc}/I_{pa}\|$
0.05	–17.17	32.56	0.53
0.10	–23.77	45.45	0.52
0.25	–37.00	64.55	0.57
0.50	–48.83	86.33	0.57
1.00	–104.3	131.90	0.79

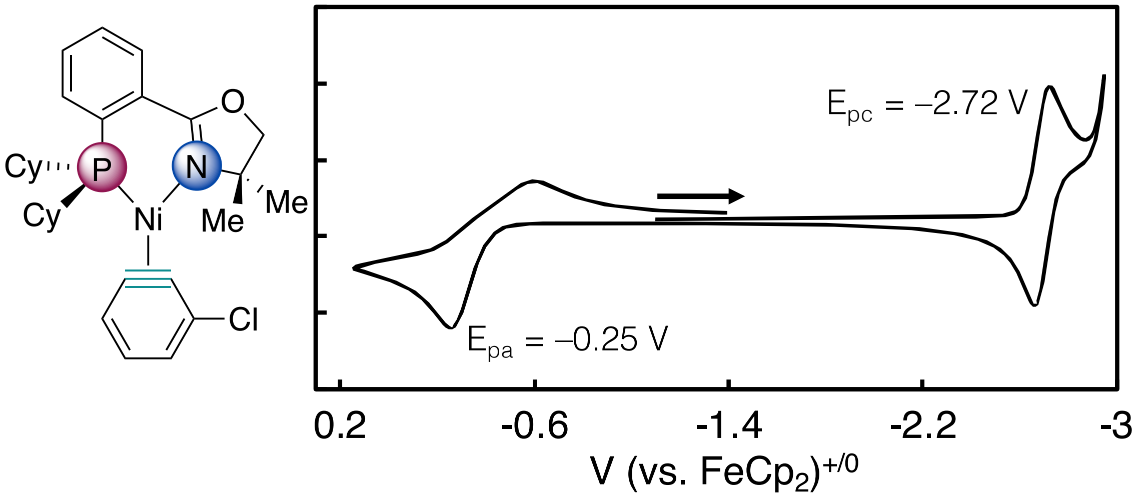

Figure S116. Full window cyclic voltammogram for **4–Cl** collected in 0.1 M [$^nPr_4N$]BAr^F_4 THF solutions with 3 mM analyte at 100 mV/s under Ar and ambient temperature. Ar^F: 3,5–bis(trifluoromethyl)phenyl.

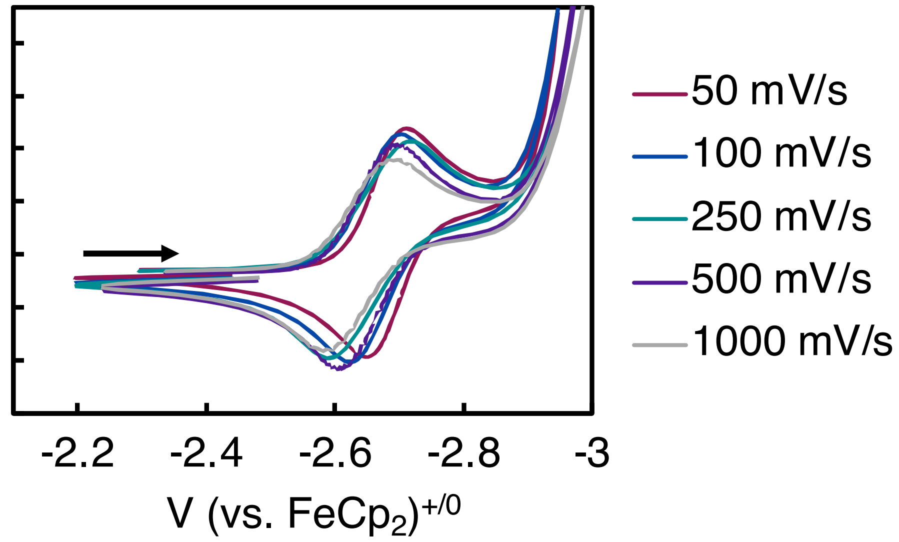

Figure S117. Scan rate dependence studies on E_{pc} for complex **4–Cl** in 0.1 M $[^nPr_4N]BAr^F_4$ THF solutions with 3 mM analyte under Ar at ambient temperatures. Ar^F: 3,5–bis(trifluoromethyl)phenyl. Peak current potentials are normalized.

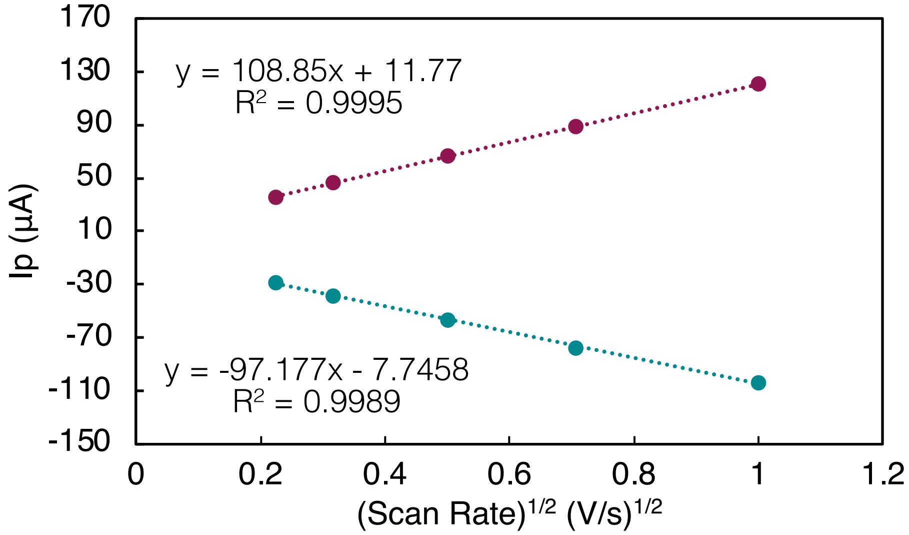

Figure S118. Anodic (I_{pa}) and cathodic (I_{pc}) peak current plotted against the square root of the scan rate $(V/s)^{1/2}$ of the observed quasi-reversible reduction E_{pc} in **4–Cl.**

Table S18. Anodic and cathodic peak currents for E_{pc} and ratios from scan rate dependence studies on complex **4–Cl**.

Scan Rate (V/s)	I_{pa} (μA)	I_{pc} (μA)	$\lvert I_{pc}/I_{pa}\rvert$
0.05	–28.79	35.03	0.82
0.10	–38.48	46.95	0.82
0.25	–56.48	66.83	0.85
0.50	–78.02	88.75	0.88
1.00	–103.90	120.30	0.86

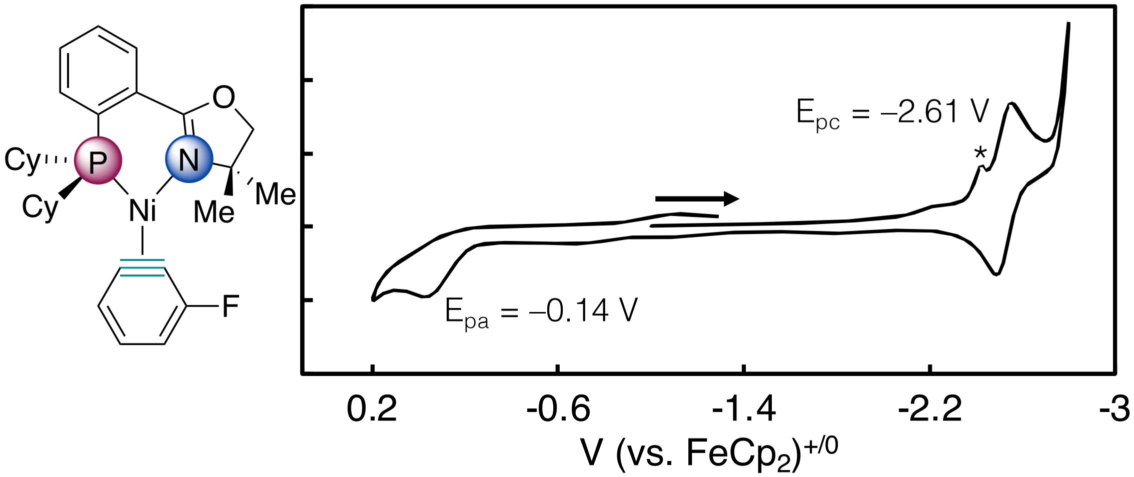

Figure S119. Full window cyclic voltammogram for **4–F** collected in 0.1 M [$^{n}Pr_4N$]BAr^F_4 THF solutions with 3 mM analyte at 100 mV/s under Ar and ambient temperature. Ar^F: 3,5–bis(trifluoromethyl)phenyl. Asterisks (*) indicate minor impurities.

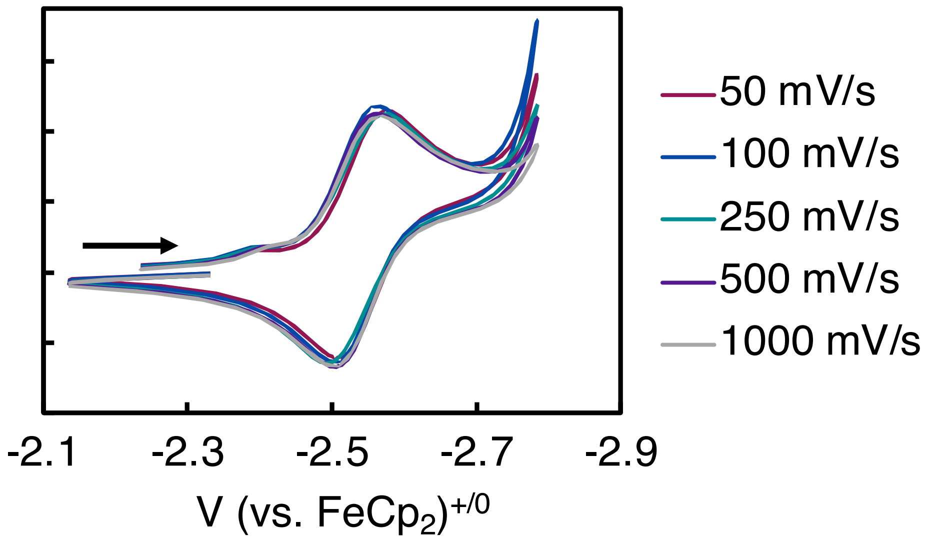

Figure S120. Scan rate dependence studies on E_{pc} for complex **4–F** in 0.1 M [^{n}Pr$_4$N]BAr$^F_4$ THF solutions with 3 mM analyte under Ar at ambient temperatures. Ar^F: 3,5–bis(trifluoromethyl)phenyl. Peak current potentials are normalized.

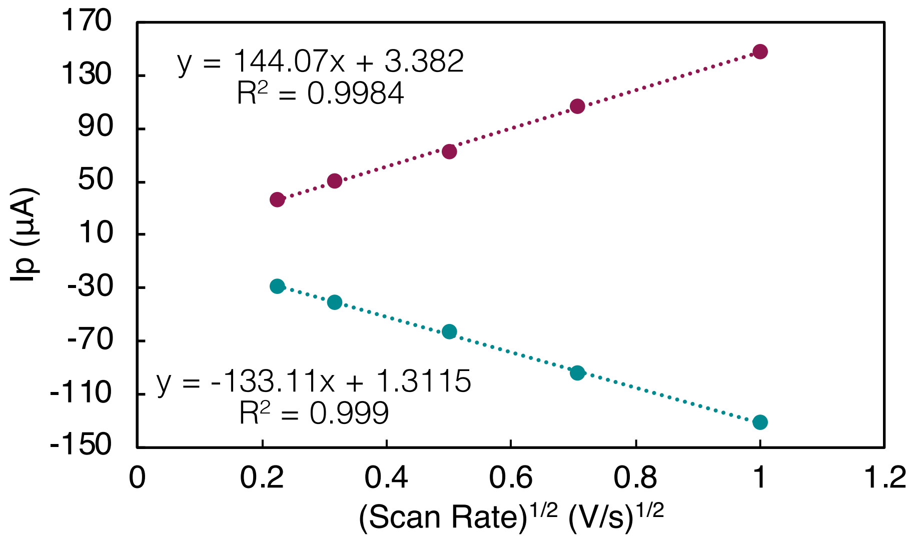

Figure S121. Anodic (I_{pa}) and cathodic (I_{pc}) peak current plotted against the square root of the scan rate $(V/s)^{1/2}$ of the observed quasi-reversible reduction E_{pc} in **4–F.**

Table S19. Anodic and cathodic peak currents for E_{pc} and ratios from scan rate dependence studies on complex **4–F**.

Scan Rate (V/s)	I_{pa} (μA)	I_{pc} (μA)	$\lvert I_{pc}/I_{pa} \rvert$
0.05	–28.94	36.28	0.80
0.10	–40.9	50.03	0.82
0.25	–63.32	72.26	0.88
0.50	–94.54	106.30	0.89
1.00	–131.40	147.80	0.89

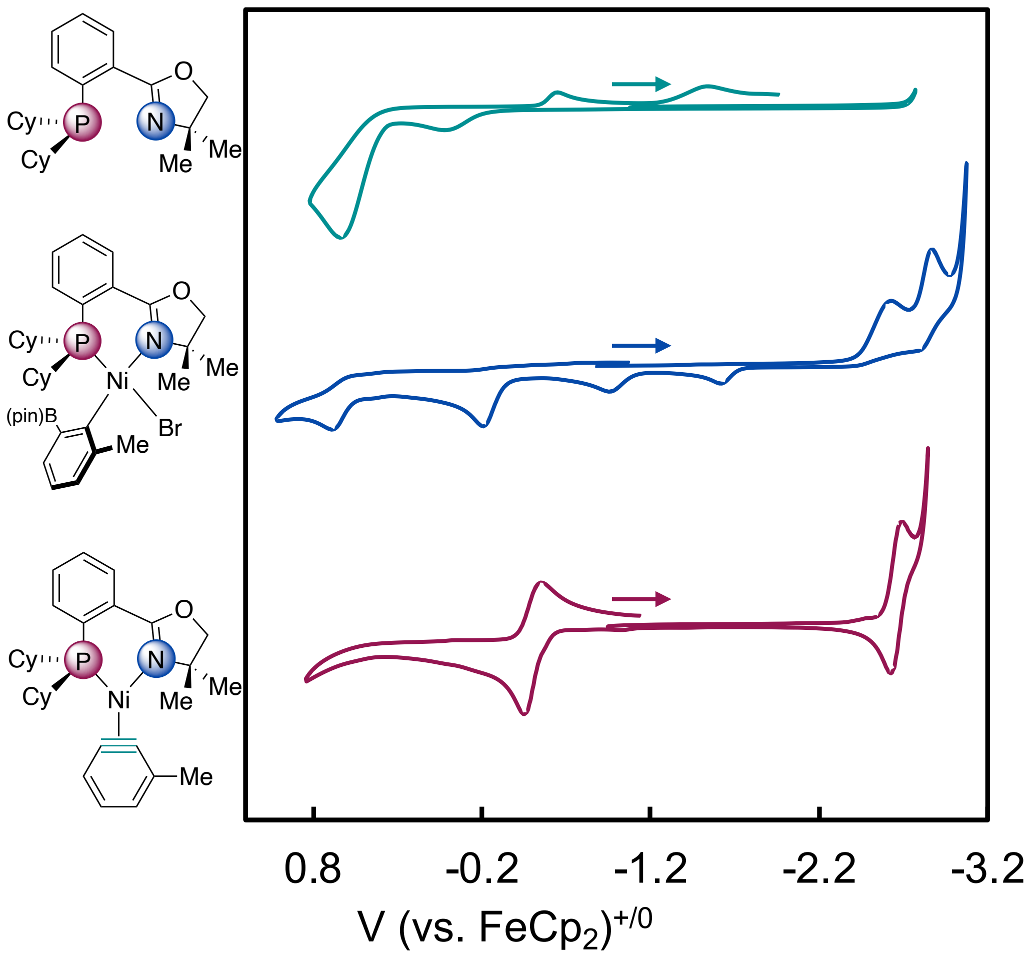

Figure S122. Stacked cyclic voltammograms of CyPHOX, **3–Me**, and **4–Me** in 0.1 M [^{n}Pr$_4$N]BAr$^F_4$ THF solutions with 3 mM analyte under Ar at ambient temperatures. Ar^F: 3,5–bis(trifluoromethyl)phenyl. Peak current potentials are normalized.

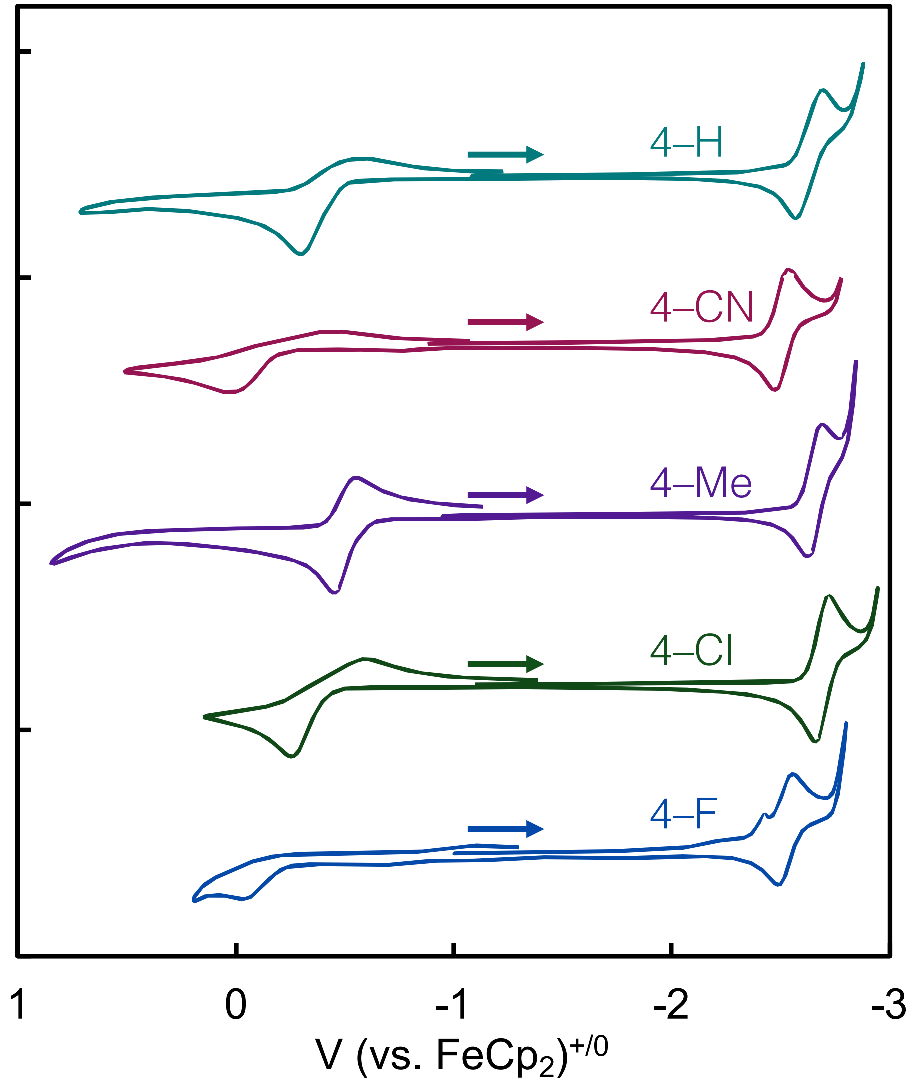

Figure S123. Stacked cyclic voltammograms of complexes **4–R** (R = H, CN, Me, Cl, and F) in 0.1 M $[^nPr_4N]BAr^F_4$ THF solutions with 3 mM analyte under Ar at ambient temperatures. Ar^F: 3,5–bis(trifluoromethyl)phenyl. Peak current potentials are normalized.

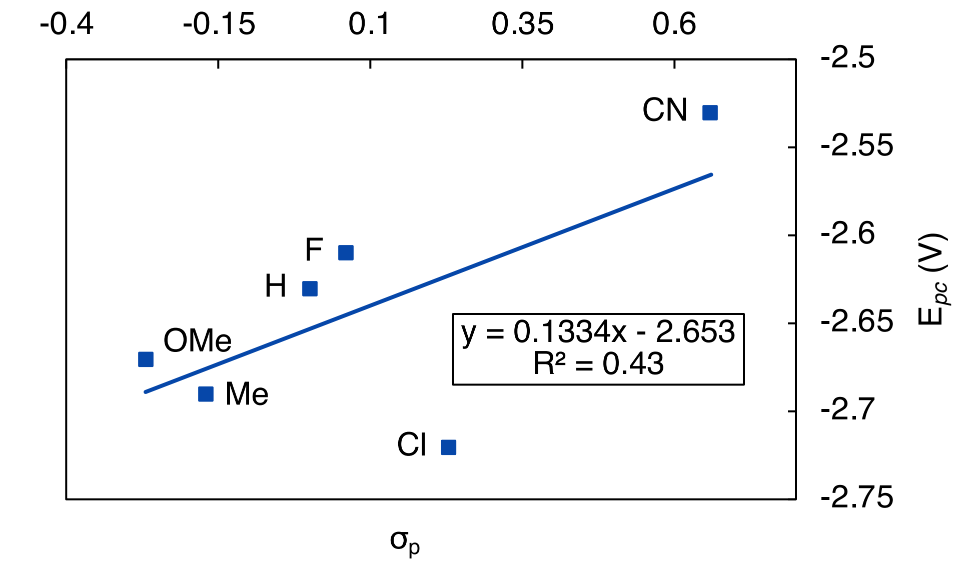

SI Figure S124: Hammett parameter trend between σ_p and E_{pc} (V) shows poor correlation.

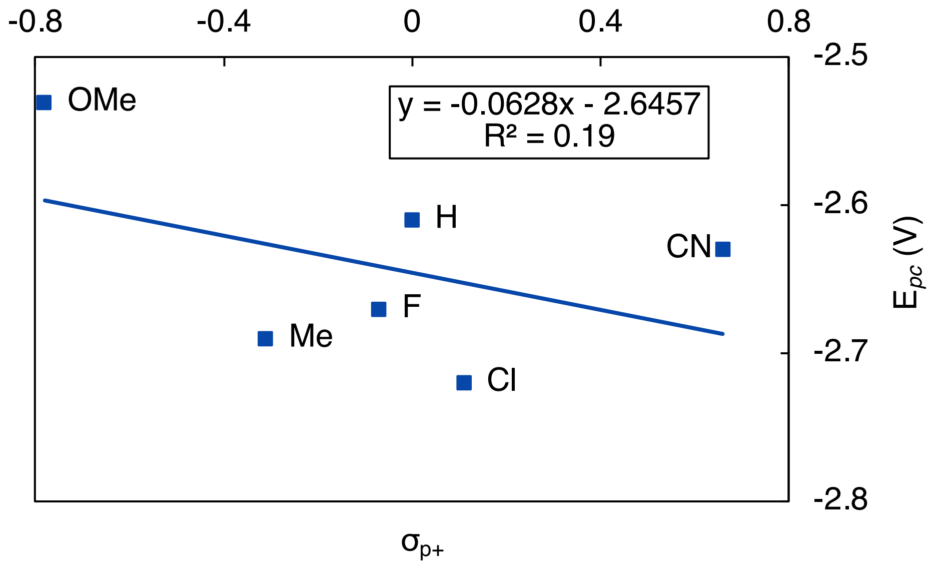

SI Figure S125: Hammett parameter trend between σ_{p+} and E_{pc} (V) shows negligible correlation.

Electron Paramagnetic Resonance (EPR) Spectroscopy

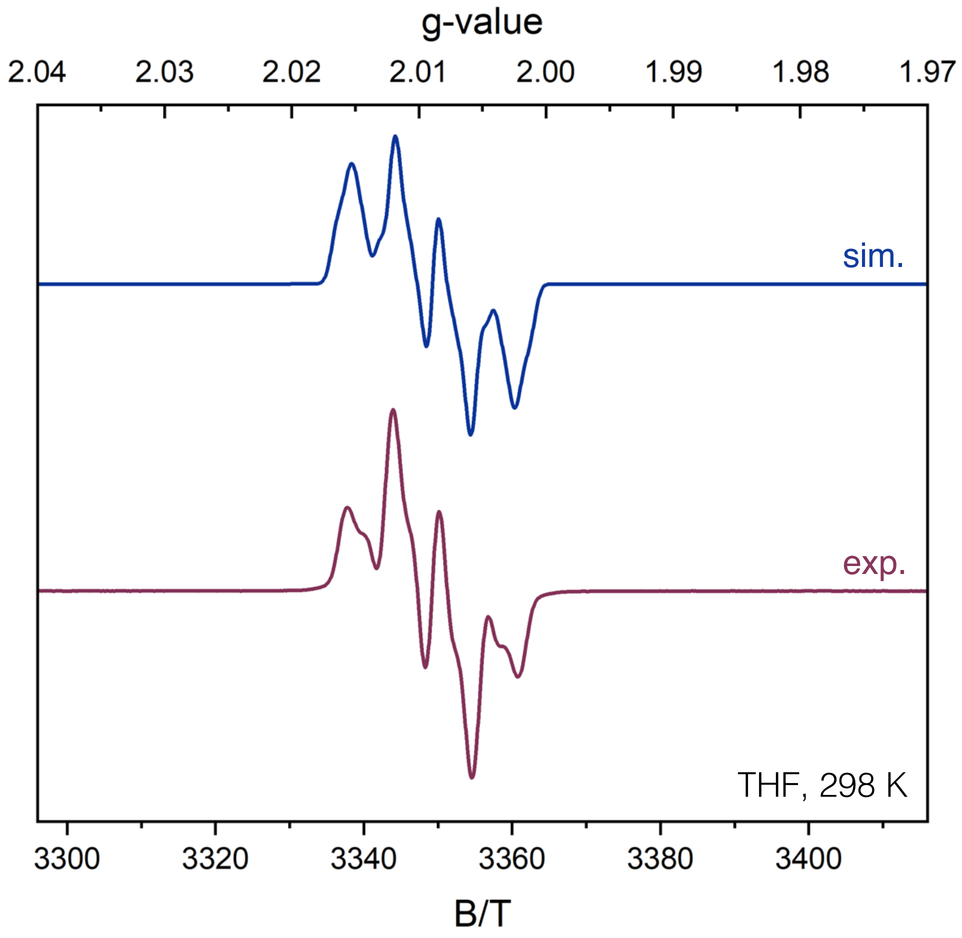

SI Figure S126: X-band EPR spectrum of 1.0 mM solution of **5-H** in THF at 298 K (exp) and simulated spectrum (sim). The spectrum was simulated using EasySpin in MATLAB.

Computational Methods

Density functional theory (DFT) calculations were performed using Gaussian 16.[20] All DFT geometry optimizations and vibrational frequency calculations were performed using the B3LYP-D3 functional[21] with the def2-SVP[22] basis set in the gas phase. Single-point energy calculations were performed using the ωB97X-D[23] functional with the def2-TZVP[22] basis set. Solvation energy corrections were calculated in tetrahydrofuran (THF) solvent with the SMD[24] continuum solvation model in single point energy calculations. The NPA charges were calculated using the NBO 3.1 program embedded in Gaussian 16. The reported Gibbs free energies include thermal corrections calculated at 298.15 K. Quasi-harmonic approximations for enthalpy were performed using the GoodVibes software package at 298.15 K with a concentration correction of 1 mol/L, vibrational scaling factor of 1.0, and frequency cutoff of 100 cm^{-1}.[25]

Optimized Geometries of Ni-Aryne Complexes

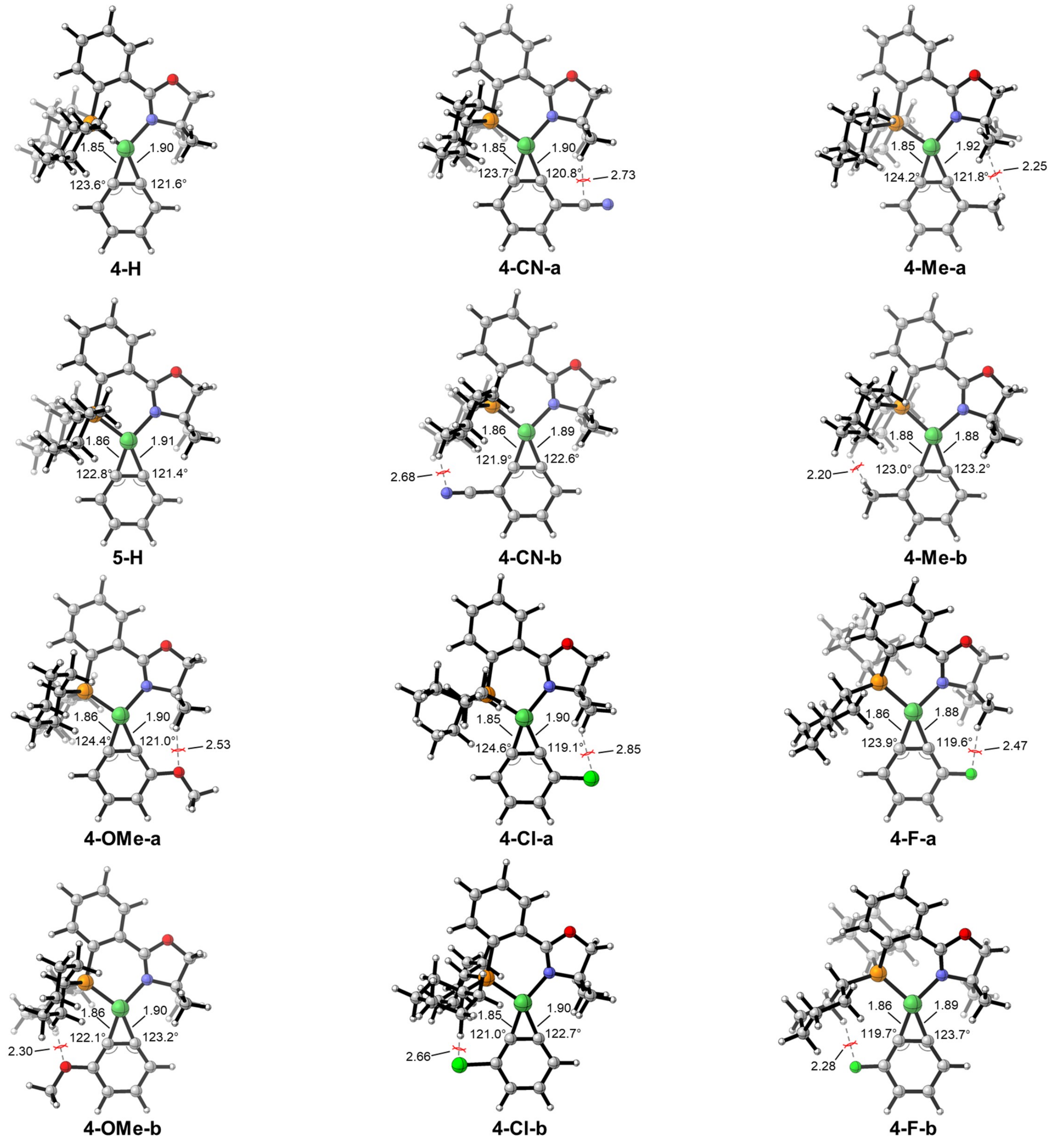

Figure S127. Computed 3D structures of key aryne complexes optimized at the B3LYP-D3/def2-SVP level of theory. Distances are in Å, with steric interactions between the aryne substituents and the ligand shown.

Distortion/Interaction Analysis of Major and Minor Regioisomers

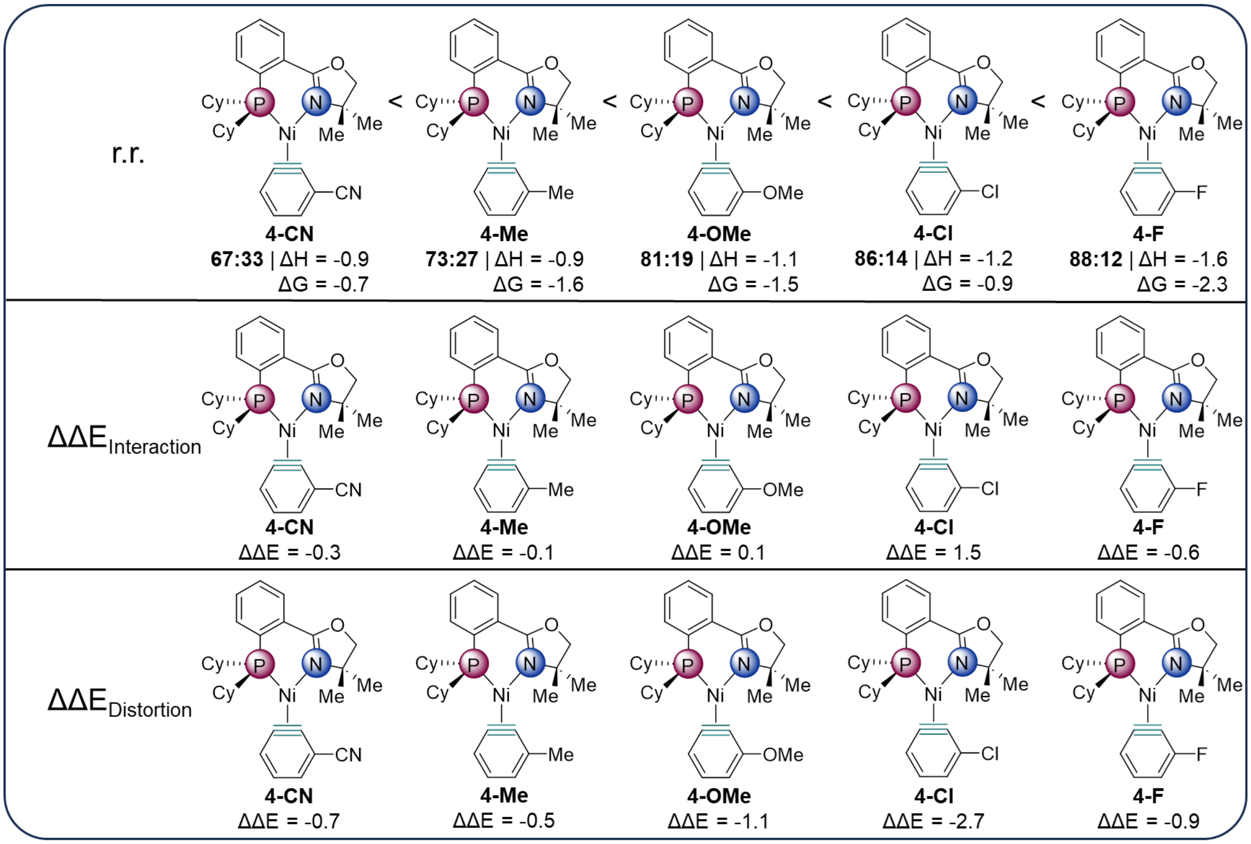

Figure S128. Distortion interaction analysis for the major and minor regioisomers of Ni-aryne complexes. *r.r.*: experimental regioisomeric ratio. Δ*G*: computed Gibbs free energy differences between the two regioisomers. Δ*H*: computed enthalpy differences between the two regioisomers. $\Delta\Delta E_{\text{Interaction}}$ and $\Delta\Delta E_{\text{Distortion}}$: interaction and distortion energy differences between the two regioisomers. All energies in kcal/mol. Level of theory: ωB97X-D/def2-TZVP/SMD(THF)//B3LYP-D3/def2-SVP.

Distortion/Interaction Analysis – Distortion Decomposition

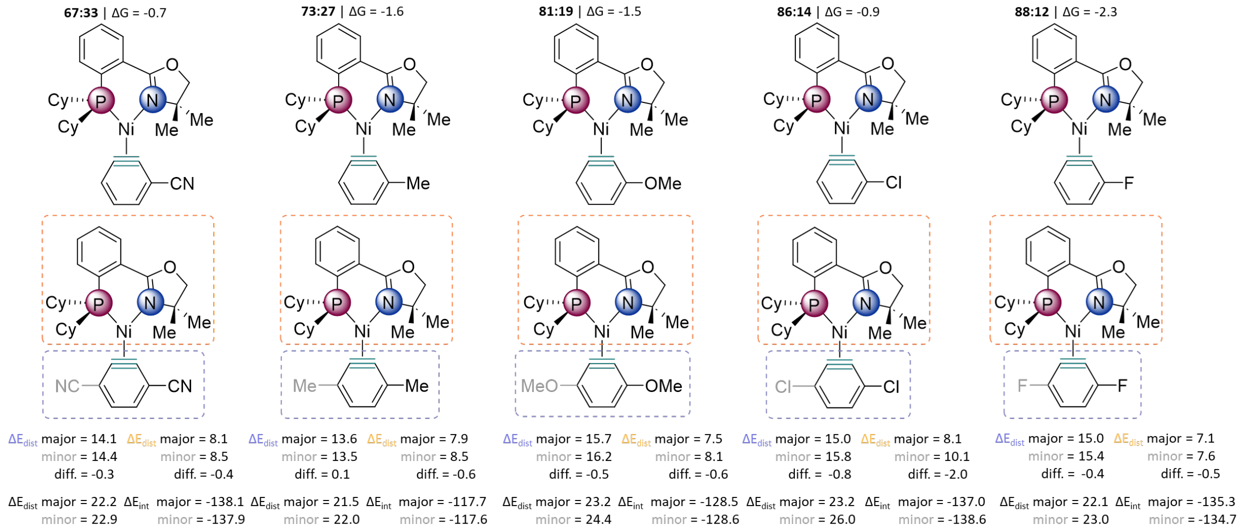

Figure S129. Distortion energies decomposed into aryne (light blue) and (CyPHOX)Ni complex (light orange) components for both major and minor regioisomers. All energies in kcal/mol.

Natural Population Analysis (NPA) Charges and Highest Occupied Molecular Orbitals (HOMOs)

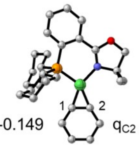

q_{C1} = -0.149 q_{C2} = -0.235

NPA Charge Difference (Δq_{NPA}) = $|q_{C2} - q_{C1}|$

4-R-a	ΔqNPA	log(r.r.)
H	0.086	n/a
CN	0.023	0.301
Me	0.099	0.431
OMe	0.161	0.653
Cl	0.145	0.788
F	0.175	0.865

4-R-b	ΔqNPA	log(r.r.)
H (red.)	0.076	n/a
CN	0.126	0.301
Me	0.051	0.431
OMe	0.005	0.653
Cl	0.010	0.788
F	0.072	0.865

4-CN | **67:33** | ΔG = -0.7

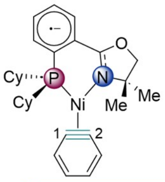

NPA – **5-H**

Ni = -0.074

C_1 = -0.162

C_2 = -0.238

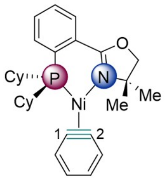

NPA – **4-H**

Ni = -0.073

C_1 = -0.149

C_2 = -0.235

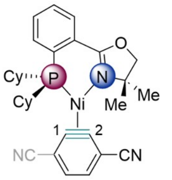

NPA – **4-CN-a**	NPA – **4-CN-b**
Ni = -0.036	Ni = -0.029
C_1 = -0.147	C_1 = -0.097
C_2 = -0.170	C_2 = -0.223

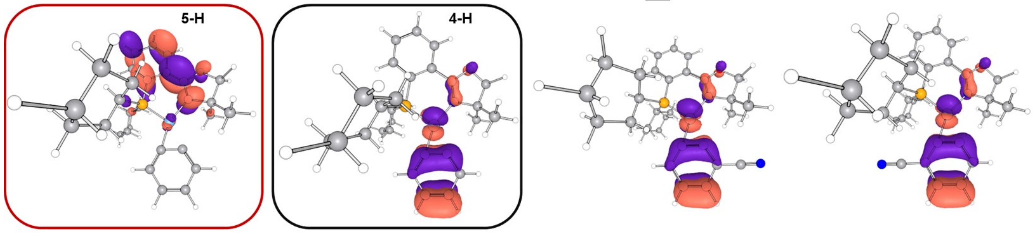

4-Me | **73:27** | ΔG = -1.6

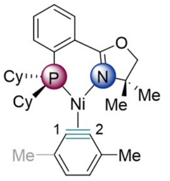

NPA – **4-Me-a**	NPA – **4-Me-b**
Ni = -0.055	Ni = -0.047
C_1 = -0.146	C_1 = -0.172
C_2 = -0.238	C_2 = -0.211

4-OMe | **81:19** | ΔG = -1.5

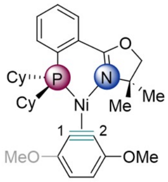

NPA – **4-OMe-a**	NPA – **4-OMe-b**
Ni = -0.078	Ni = -0.069
C_1 = -0.118	C_1 = -0.203
C_2 = -0.279	C_2 = -0.198

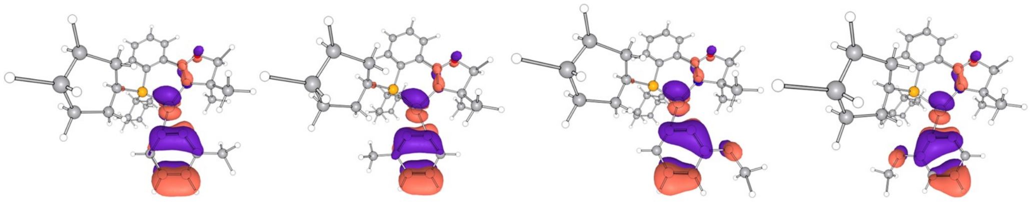

4-Cl | **86:14** | ΔG = -0.9

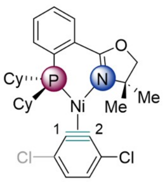

NPA – **4-Cl-a**	NPA – **4-Cl-b**
Ni = -0.046	Ni = -0.045
C_1 = -0.118	C_1 = -0.186
C_2 = -0.263	C_2 = -0.196

4-F | **88:12** | ΔG = -2.3

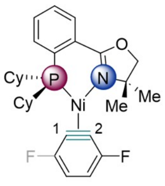

NPA – **4-F-a**	NPA – **4-F-b**
Ni = -0.040	Ni = -0.032
C_1 = -0.126	C_1 = -0.250
C_2 = -0.301	C_2 = -0.178

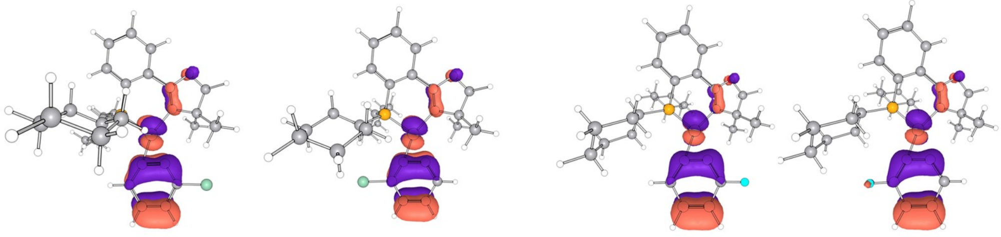

Figure S130. NPA charges are shown for nickel and both aryne carbons for each of the major and minor aryne species. The NPA charge difference was computed and is shown next to the *r.r.* in the above tables for both the major and minor isomers. Higher NPA charge difference for **4-R-a** is often associated with higher *r.r.*. Additionally, the canonical highest occupied molecular orbitals (HOMOs) are shown, and in most cases are located on the aryne (excepting **5-H**). Level of theory: ωB97X-D/def2-TZVP/SMD(THF)//B3LYP-D3/def2-SVP.

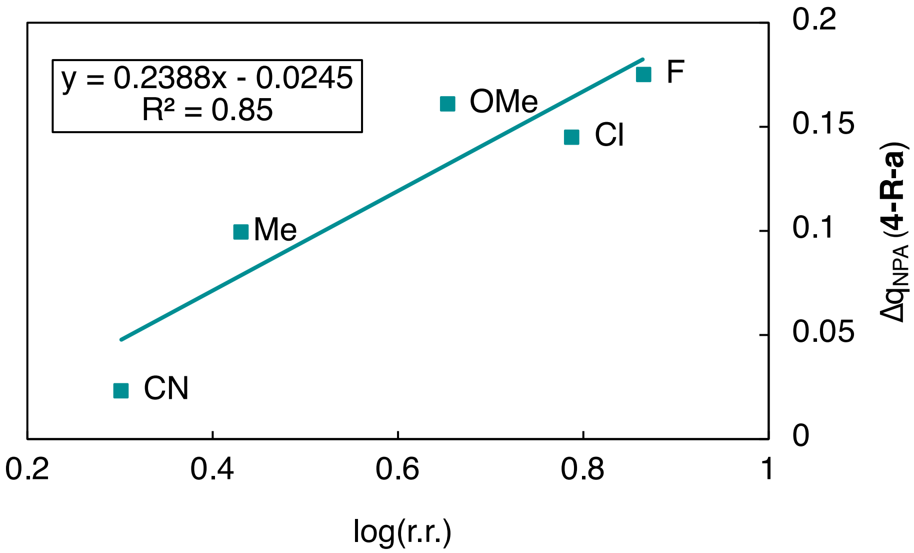

Figure S131. Correlation between experimentally observed regioisomeric ratios of aryne complexes and calculated NPA charge differences for major aryne regioisomers **4-R-a**.

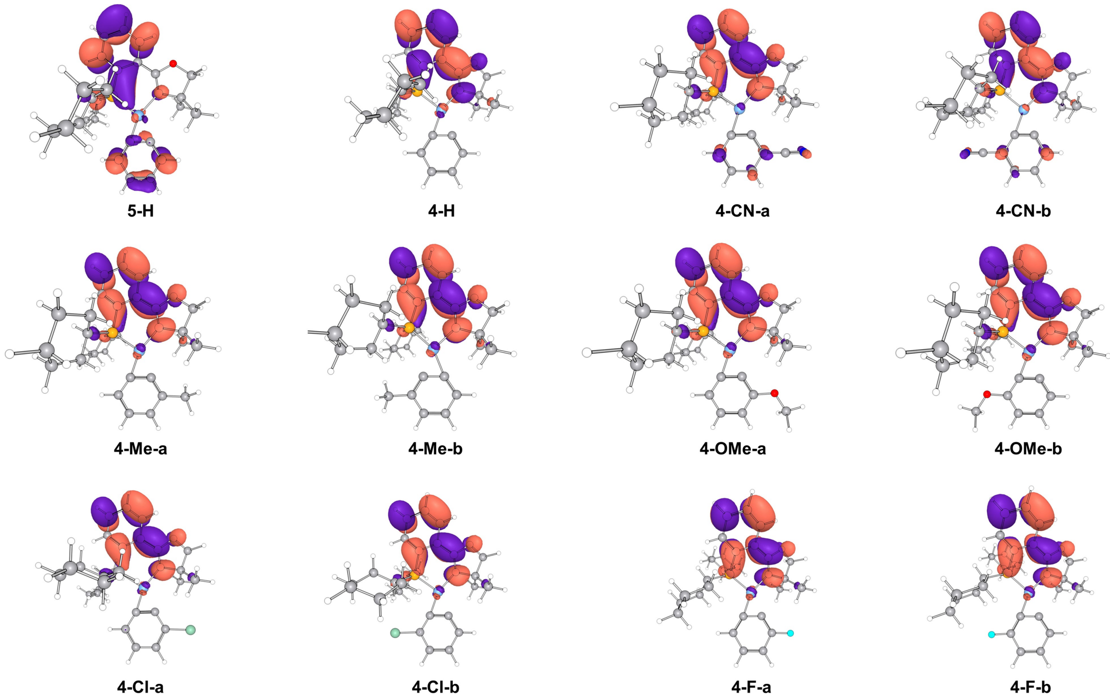

Figure S132. Canonical lowest unoccupied molecular orbitals (LUMOs), which are located on the ligand. Level of theory: ωB97X-D/def2-TZVP/SMD(THF)//B3LYP-D3/def2-SVP.

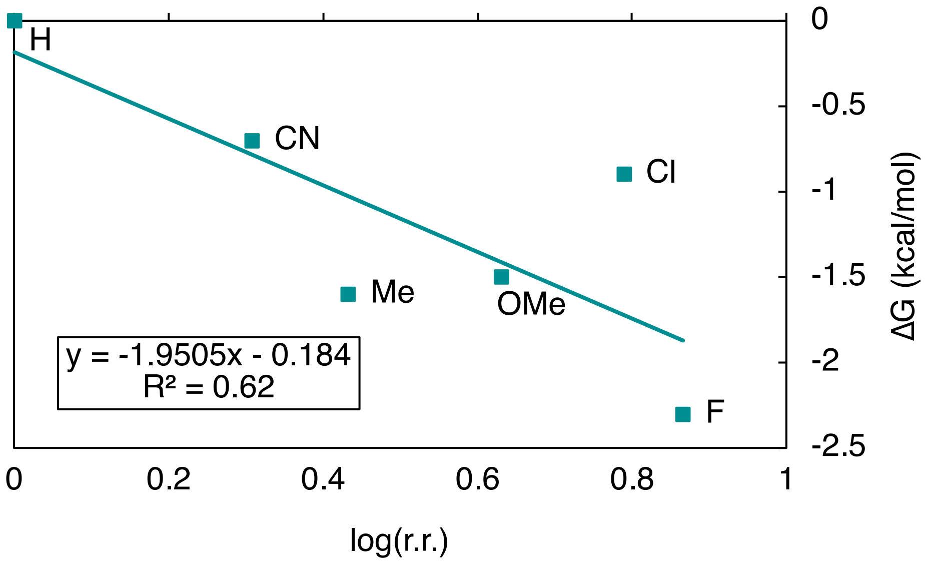

Figure S133. Correlation between experimentally observed regioisomeric ratios of aryne complexes and calculated Gibbs free energy difference between regioisomers (Δ*G*).

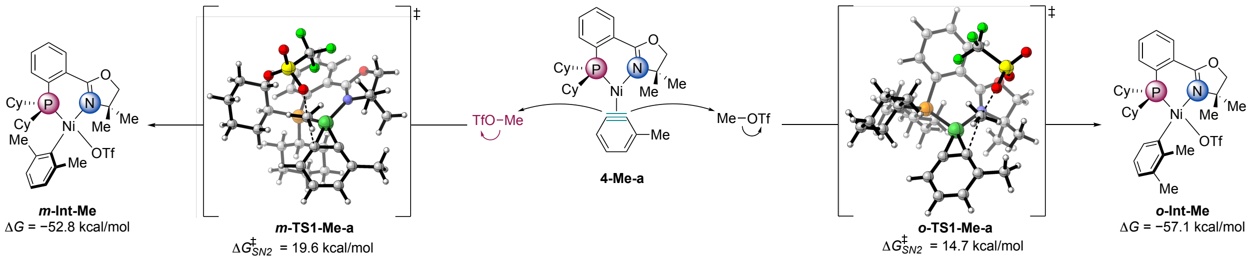

Figure S134. Transition state analysis of methylation of aryne regioisomer **4-Me-a**.

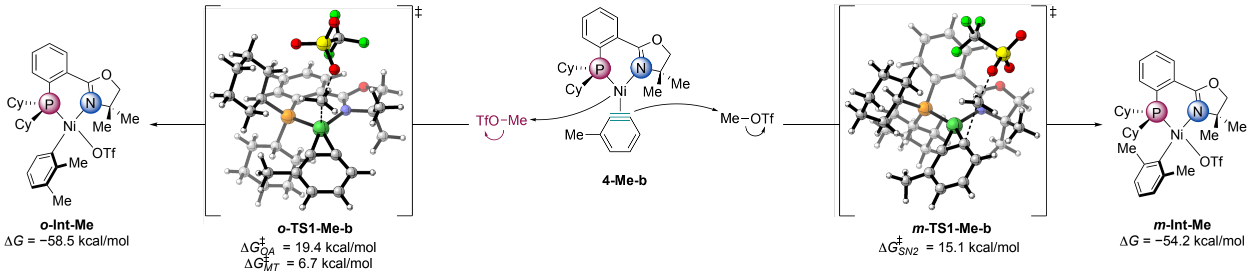

Figure S135. Transition state analysis of methylation of aryne regioisomer **4-Me-b**.

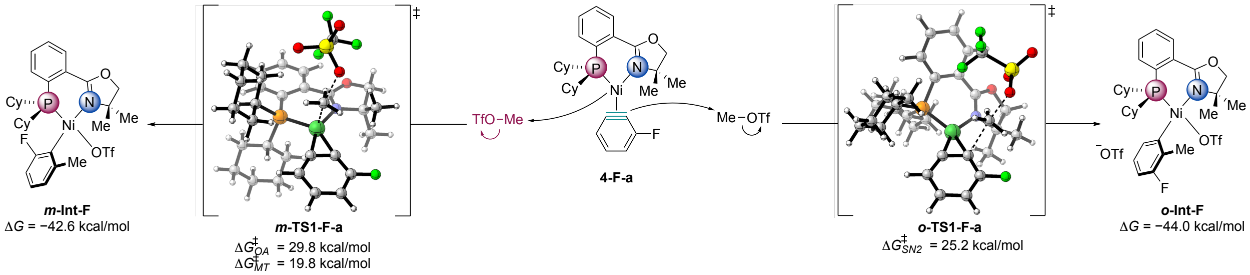

Figure S136. Transition state analysis for methylation of aryne regioisomer **4-F-a**.

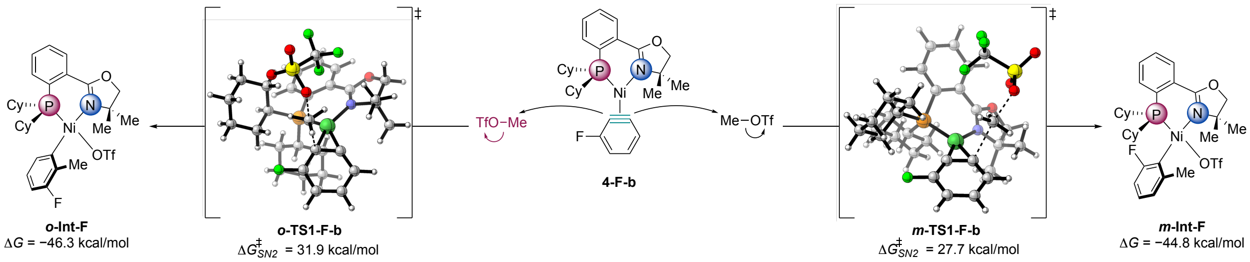

Figure S137. Transition state analysis for methylation of aryne regioisomer **4-F-b**.

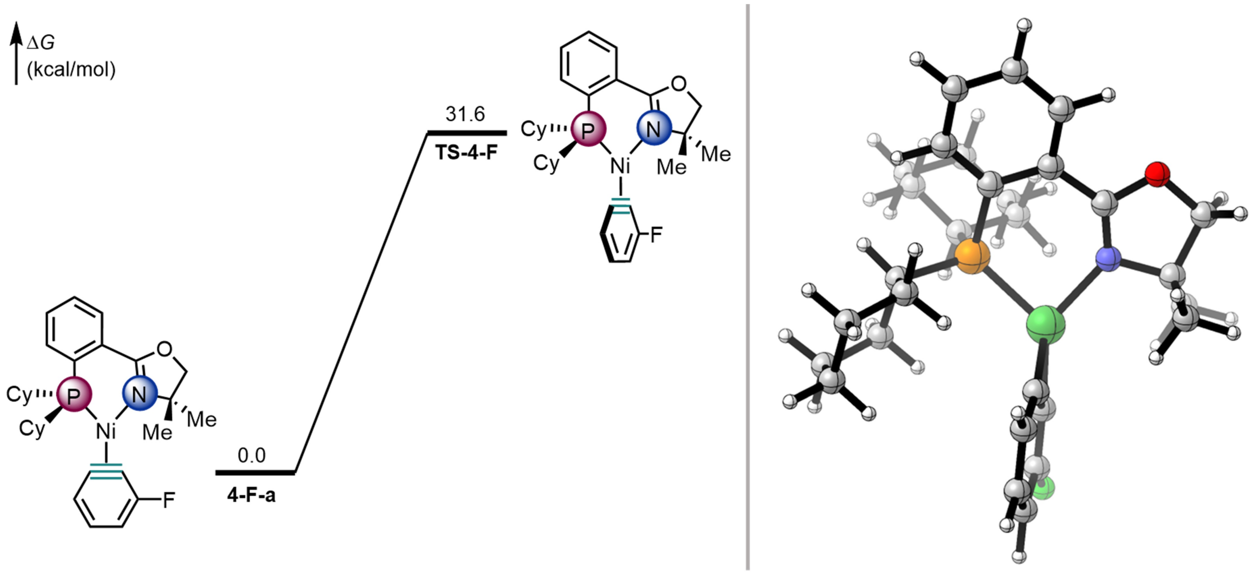

Figure S138. Transition state structure and energy barrier for **TS-4-F** corresponding to the conversion from **4-F-a** to **4-F-b**.

Cartesian Coordinates

4-CN-b

B3LYP-D3/def2-SVP SCF energy (au):	-3198.793703
B3LYP-D3/def2-SVP enthalpy (au):	-3198.148546
B3LYP-D3/def2-SVP free energy (au):	-3198.248626
ωB97X-D/def2-TZVP/SMD(THF) SCF energy (au):	-3200.071960
ωB97X-D/def2-TZVP/SMD(THF) enthalpy (au):	-3199.426803
ωB97X-D/def2-TZVP/SMD(THF) free energy (au):	-3199.526883

Cartesian coordinates

ATOM	X	Y	Z
Ni	9.879536	8.403695	11.639351
P	11.288120	10.038037	11.391122
N	10.007155	8.313574	13.605998
C	7.704608	6.177502	10.618656
C	8.648974	7.156886	10.918685
C	9.252801	7.906312	9.963190
C	8.966837	7.739375	8.600299
C	8.007880	6.747773	8.262570
H	7.755456	6.584687	7.211818
C	7.390392	5.985095	9.256981
H	6.654554	5.229022	8.967215
C	12.873102	9.627417	10.478317

H	13.453396	10.561044	10.362245
C	12.600491	9.029268	9.086860
H	12.064464	9.737804	8.444849
H	11.929747	8.162082	9.206577
C	13.892501	8.588290	8.388624
H	13.640822	8.149910	7.409320
H	14.519716	9.476059	8.183185
C	14.687060	7.596705	9.243001
H	14.099622	6.667495	9.359864
H	15.627682	7.314507	8.740824
C	14.974577	8.185848	10.627698
H	15.646198	9.057993	10.520231
H	15.509240	7.456426	11.259430
C	13.687170	8.631302	11.333418
H	13.933012	9.068351	12.313815
H	13.051880	7.747936	11.532046
C	10.554164	11.615750	10.691076
H	11.365254	12.362951	10.618293
C	9.934464	11.413466	9.298366
H	10.687537	11.092064	8.568679
H	9.199581	10.594492	9.360116
C	9.242589	12.683923	8.789554
H	9.997933	13.477670	8.637121
H	8.798059	12.480544	7.801820
C	8.180127	13.181357	9.773810
H	7.714290	14.111765	9.407869
H	7.372589	12.429754	9.844807
C	8.788314	13.396895	11.163417
H	8.014785	13.713279	11.883492
H	9.525847	14.219689	11.114979
C	9.484790	12.131109	11.679357
H	8.735628	11.330461	11.823600
H	9.929930	12.327192	12.667597
C	11.968922	10.684699	12.988711
C	12.884501	11.749146	12.944678
H	13.179934	12.161446	11.977611
C	13.432604	12.301436	14.101398
H	14.141723	13.129850	14.029117
C	13.066361	11.786792	15.347323
H	13.483835	12.206558	16.265497
C	12.164501	10.729877	15.418375
H	11.882921	10.327311	16.389890
C	11.602607	10.161224	14.252523
C	10.663950	9.031542	14.458818
C	9.195794	7.306011	14.364307
C	9.557486	5.890051	13.912820

H	9.004259	5.150324	14.513854
H	10.636223	5.708614	14.040057
H	9.308933	5.757485	12.852845
C	7.706944	7.606422	14.166442
H	7.096407	6.886631	14.734988
H	7.452499	7.539295	13.100982
H	7.468283	8.622823	14.517793
O	10.483495	8.713706	15.758443
C	9.637298	7.559393	15.828836
H	10.223797	6.727485	16.250513
H	8.798994	7.783965	16.504873
H	7.207511	5.564557	11.380473
C	9.585542	8.505997	7.555686
N	10.092656	9.134196	6.718567

4-CN-a

B3LYP-D3/def2-SVP SCF energy (au): -3198.793202
B3LYP-D3/def2-SVP enthalpy (au): -3198.148072
B3LYP-D3/def2-SVP free energy (au): -3198.247706
ωB97X-D/def2-TZVP/SMD(THF) SCF energy (au): -3200.073443
ωB97X-D/def2-TZVP/SMD(THF) enthalpy (au): -3199.428313
ωB97X-D/def2-TZVP/SMD(THF) free energy (au): -3199.527947

Cartesian coordinates

ATOM	X	Y	Z
Ni	9.834002	8.402735	11.709768
P	11.183613	10.064648	11.444266
N	7.030094	4.283289	12.288955
N	9.939457	8.346036	13.677888
C	8.026743	5.918989	10.529547
C	8.810540	7.011560	10.926641
C	9.331221	7.863443	10.008802
C	9.132427	7.737176	8.637120
H	9.541025	8.432433	7.895770
C	8.347964	6.642659	8.215609
H	8.157114	6.489993	7.149265
C	7.803988	5.744340	9.138881
H	7.199866	4.902334	8.791434
C	7.464647	5.000133	11.482764
C	12.763144	9.592993	10.546877
H	13.471792	10.432476	10.662696
C	12.567353	9.328945	9.045031
H	12.225604	10.244042	8.537352
H	11.776419	8.572136	8.915879
C	13.863564	8.836428	8.384932
H	13.677089	8.627393	7.318263

H	14.619471	9.643270	8.418780
C	14.422415	7.594759	9.087020
H	13.706742	6.760449	8.971394
H	15.363464	7.272778	8.610610
C	14.639796	7.866061	10.579297
H	15.425374	8.635025	10.700857
H	15.006619	6.960006	11.089817
C	13.348700	8.350114	11.249662
H	13.530326	8.563822	12.315849
H	12.591256	7.546359	11.215325
C	10.511452	11.621123	10.641772
H	11.370532	12.238644	10.319251
C	9.651666	11.269740	9.410056
H	10.235270	10.709205	8.667451
H	8.849090	10.586267	9.732980
C	9.044496	12.521008	8.763970
H	9.854830	13.161638	8.368168
H	8.427969	12.226013	7.898625
C	8.211179	13.321364	9.769601
H	7.810007	14.236327	9.302466
H	7.339484	12.715687	10.078078
C	9.042428	13.671881	11.007740
H	8.426276	14.204311	11.751678
H	9.852079	14.367235	10.717545
C	9.664684	12.425260	11.651107
H	8.862028	11.768144	12.034641
H	10.268701	12.721936	12.521466
C	11.912292	10.698557	13.024177
C	12.819258	11.769999	12.966090
H	13.055798	12.218185	11.998265
C	13.426472	12.288061	14.109095
H	14.126981	13.122784	14.026562
C	13.130037	11.729646	15.354685
H	13.597127	12.119219	16.262219
C	12.230171	10.671783	15.440492
H	11.995954	10.241042	16.412589
C	11.606047	10.139168	14.289808
C	10.653048	9.023581	14.516028
C	9.171854	7.307604	14.443613
C	9.738214	5.926607	14.098270
H	9.168465	5.138305	14.612935
H	10.796922	5.860101	14.396826
H	9.667993	5.757559	13.017111
C	7.679125	7.416216	14.135760
H	7.115747	6.673498	14.721234
H	7.499001	7.229670	13.071287

```
H     7.308270     8.423797    14.381643
O    10.522724     8.677409    15.814252
C     9.476764     7.698163    15.910900
H     9.840966     6.859491    16.521215
H     8.616067     8.165106    16.416838
```

4-F-b

```
B3LYP-D3/def2-SVP SCF energy (au):                         -3205.778936
B3LYP-D3/def2-SVP enthalpy (au):                           -3205.141318
B3LYP-D3/def2-SVP free energy (au):                        -3205.236474
ωB97X-D/def2-TZVP/SMD(THF) SCF energy (au):                    -3207.083169
ωB97X-D/def2-TZVP/SMD(THF) enthalpy (au):                      -3206.445551
ωB97X-D/def2-TZVP/SMD(THF) free energy (au):                   -3206.540707
```

```
Cartesian coordinates
ATOM  X              Y           Z
P     3.011810    11.600174     8.820182
C     0.891141    10.520755     6.481499
C    -0.419958    10.835780     6.187888
Ni     2.614185    10.272560     7.136552
C    -1.070078    10.190001     5.128285
H    -2.107405    10.449842     4.904021
C    -0.381653     9.223295     4.378725
H    -0.898487     8.724206     3.552781
C     1.569887    12.180884     9.843531
H     1.944405    12.648299    10.770238
O     6.403258     8.737170     7.893353
N     4.394234     9.449607     7.204967
C     0.952522     8.891615     4.681237
C     0.701879    10.961267    10.211482
H     0.424014    10.447466     9.275697
H     1.291576    10.241644    10.804456
C    -0.563975    11.370673    10.973392
H    -0.281732    11.797101    11.954936
H    -1.174049    10.476412    11.184294
C    -1.375598    12.402952    10.183250
H    -2.272040    12.704793    10.750821
H    -1.723649    11.947258     9.241398
C    -0.518571    13.629211     9.849551
H    -1.098904    14.352995     9.253506
H    -0.237952    14.145732    10.787455
C     0.753581    13.243087     9.081063
H     1.363147    14.145006     8.908802
H     0.471417    12.844529     8.095830
C     3.964134    13.153146     8.379142
H     3.250959    13.692536     7.730066
```

C	4.347521	14.061295	9.557078
H	5.011606	13.504431	10.241542
H	3.455840	14.341723	10.140580
C	5.071057	15.326567	9.071544
H	4.369478	15.935674	8.472409
H	5.364475	15.946566	9.935362
C	6.296438	14.981899	8.216640
H	7.041668	14.461291	8.846641
H	6.782495	15.902397	7.852414
C	5.913709	14.075854	7.040043
H	6.808321	13.802603	6.455346
H	5.248072	14.630711	6.353798
C	5.193951	12.808733	7.518714
H	5.897534	12.199478	8.115541
H	4.882178	12.184802	6.666756
C	4.147712	10.763061	10.026215
C	4.118264	11.076656	11.394091
H	3.397575	11.806373	11.763608
C	4.990280	10.482349	12.309128
H	4.929148	10.746877	13.367751
C	5.938758	9.561184	11.863494
H	6.631580	9.092556	12.566250
C	5.998040	9.237688	10.509976
H	6.736908	8.519845	10.154824
C	5.106368	9.812536	9.582053
C	5.239083	9.363105	8.177970
C	6.428125	8.514206	6.473747
H	6.817199	7.504318	6.284144
H	7.106939	9.257612	6.022129
C	4.960246	8.708900	6.033037
C	4.221041	7.370947	5.913443
H	3.144481	7.558178	5.810024
H	4.382188	6.757796	6.814405
H	4.583693	6.810506	5.037078
C	4.846280	9.535808	4.754959
H	5.343135	9.014217	3.920983
H	5.321545	10.520481	4.885062
H	3.788734	9.693499	4.504260
C	1.535927	9.573411	5.749633
H	1.474492	8.132145	4.088148
F	-1.130483	11.767576	6.890102

4-F-a

B3LYP-D3/def2-SVP SCF energy (au): -3205.778081
B3LYP-D3/def2-SVP enthalpy (au): -3205.140661
B3LYP-D3/def2-SVP free energy (au): -3205.236878

ωB97X-D/def2-TZVP/SMD(THF) SCF energy (au): -3207.085561
ωB97X-D/def2-TZVP/SMD(THF) enthalpy (au): -3206.448141
ωB97X-D/def2-TZVP/SMD(THF) free energy (au): -3206.544358

Cartesian coordinates

ATOM	X	Y	Z
P	3.025899	11.597821	8.818732
C	0.906129	10.553240	6.479748
C	-0.407836	10.956745	6.237406
H	-0.933750	11.719637	6.820165
Ni	2.625718	10.284376	7.144949
C	-1.065657	10.318956	5.168533
H	-2.098624	10.587811	4.925927
C	-0.419234	9.336446	4.400778
H	-0.927047	8.840947	3.569686
C	1.575736	12.162336	9.840622
H	1.936724	12.655974	10.758874
O	6.401566	8.709079	7.906717
N	4.396661	9.428772	7.212759
F	1.512426	8.033679	3.946144
C	0.901689	8.985269	4.707941
C	0.736897	10.928439	10.228240
H	0.486373	10.382383	9.302458
H	1.339430	10.241463	10.845764
C	-0.549995	11.317623	10.964631
H	-0.295047	11.777770	11.938031
H	-1.136770	10.411700	11.190220
C	-1.381198	12.306453	10.140316
H	-2.296152	12.592816	10.685435
H	-1.708116	11.811865	9.208017
C	-0.557694	13.550765	9.790384
H	-1.150289	14.247225	9.173762
H	-0.307994	14.092518	10.722059
C	0.739550	13.190101	9.052935
H	1.325480	14.106644	8.876984
H	0.503187	12.771447	8.062619
C	3.962842	13.158933	8.375251
H	3.249429	13.687767	7.717720
C	4.332641	14.079015	9.548091
H	4.996638	13.532708	10.240977
H	3.435427	14.358010	10.124203
C	5.050246	15.345446	9.056494
H	4.348108	15.944816	8.448178
H	5.334194	15.974391	9.917001
C	6.283030	15.002286	8.211598
H	7.027873	14.491769	8.850196

H	6.764951	15.923301	7.843284
C	5.913659	14.084572	7.039750
H	6.813584	13.812816	6.462756
H	5.248602	14.629494	6.345018
C	5.199830	12.816286	7.523865
H	5.903577	12.215935	8.129376
H	4.896751	12.183615	6.675093
C	4.166346	10.771236	10.025421
C	4.147007	11.102983	11.389336
H	3.432791	11.841822	11.753619
C	5.021198	10.515360	12.306435
H	4.968876	10.793511	13.362019
C	5.960744	9.582655	11.865784
H	6.655006	9.118181	12.569880
C	6.010051	9.242298	10.515941
H	6.742744	8.516032	10.165321
C	5.116850	9.810921	9.585609
C	5.242178	9.345545	8.184773
C	6.421875	8.468393	6.489681
H	6.799829	7.452120	6.312592
H	7.108510	9.198833	6.028635
C	4.955503	8.671856	6.045658
C	4.210583	7.337280	5.924306
H	3.148872	7.519054	5.719236
H	4.305111	6.753869	6.854010
H	4.632907	6.748470	5.094312
C	4.851486	9.486767	4.758725
H	5.374865	8.965185	3.941153
H	5.305080	10.481820	4.888060
H	3.797147	9.612397	4.481779
C	1.551819	9.601805	5.755702

TS-4-F

B3LYP-D3/Def2-SVP SCF energy (au): -3205.722901
B3LYP-D3/Def2-SVP enthalpy (au): -3205.088000
B3LYP-D3/Def2-SVP free energy (au): -3205.187853
?B97XD/Def2-TZVP/SMD(THF) SCF energy (au): -3207.029096
?B97XD/Def2-TZVP/SMD(THF) enthalpy (au): -3206.394195
?B97XD/Def2-TZVP/SMD(THF) free energy (au): -3206.494048
Imaginary Frequency (cm^{-1}): -214.05

Cartesian coordinates

ATOM	X	Y	Z
P	3.144938	11.714562	8.723190
Ni	2.798051	10.540327	6.803905
C	1.605658	12.150872	9.678011

C	4.069528	13.322381	8.443931
C	4.244471	10.827728	9.925567
C	1.141783	9.772282	6.484427
C	1.406586	10.679318	5.526352
H	1.830520	12.748217	10.579811
C	0.906069	10.838597	10.090244
C	0.704131	12.982568	8.739486
H	3.372676	13.887768	7.798945
C	4.390045	14.175864	9.679138
C	5.338747	13.043243	7.614912
C	4.198796	11.163247	11.289306
C	5.159935	9.814031	9.516791
C	-0.024763	9.044691	6.648296
C	0.432803	10.946763	4.589388
H	0.814072	10.204139	9.191590
H	1.529968	10.283887	10.809879
C	-0.488989	11.086068	10.676082
C	-0.692855	13.208149	9.331567
H	1.175388	13.954832	8.518581
H	0.618258	12.445589	7.778401
H	5.041211	13.600053	10.359650
H	3.469158	14.406671	10.239787
C	5.106203	15.477173	9.285572
C	6.059251	14.342701	7.233681
H	6.023307	12.406814	8.205646
H	5.078818	12.464137	6.712640
H	3.502017	11.933132	11.623734
C	5.019078	10.548473	12.236559
C	5.996796	9.216544	10.483562
C	5.293429	9.283489	8.133269
H	-0.197897	8.313856	7.443128
C	-1.025187	9.329799	5.683004
C	-0.801985	10.265110	4.668673
F	0.601097	11.841590	3.585330
H	-0.396556	11.644136	11.627039
H	-0.962430	10.121111	10.923694
C	-1.365204	11.882434	9.703122
H	-1.311367	13.767271	8.609975
H	-0.611888	13.841599	10.235067
H	4.419338	16.101834	8.685384
H	5.350700	16.060553	10.189276
C	6.373707	15.194083	8.469824
H	6.983426	14.112329	6.677386
H	5.415687	14.921936	6.546632
H	4.948878	10.837410	13.288326
C	5.930018	9.573725	11.827961

H	6.701993	8.449850	10.165460
O	6.330515	8.430708	7.953016
N	4.549876	9.478046	7.100391
H	-1.989101	8.813727	5.732718
H	-1.573051	10.488116	3.927119
H	-2.361461	12.064614	10.139991
H	-1.520286	11.286053	8.785922
H	7.099707	14.654429	9.106195
H	6.859142	16.138705	8.172810
H	6.585977	9.086329	12.552993
C	6.373695	8.087672	6.555196
C	5.028184	8.611733	5.992956
H	6.495682	6.998701	6.469269
H	7.248527	8.587184	6.106517
C	4.004091	7.489623	5.783397
C	5.188862	9.452947	4.725967
H	3.021074	7.928081	5.558216
H	3.906046	6.878764	6.694604
H	4.312248	6.837237	4.950915
H	5.556639	8.838134	3.889186
H	5.896140	10.280507	4.893109
H	4.212335	9.880839	4.449306

4-H

B3LYP-D3/def2-SVP SCF energy (au): -3106.607437
B3LYP-D3/def2-SVP enthalpy (au): -3105.963177
B3LYP-D3/def2-SVP free energy (au): -3106.058954
ωB97X-D/def2-TZVP/SMD(THF) SCF energy (au): -3107.818717
ωB97X-D/def2-TZVP/SMD(THF) enthalpy (au): -3107.174457
ωB97X-D/def2-TZVP/SMD(THF) free energy (au): -3107.270234

Cartesian coordinates

ATOM	X	Y	Z
Ni	5.003178	11.198386	12.890848
P	6.775572	11.444659	14.085388
O	6.522807	7.533562	11.611766
N	5.506179	9.472144	12.093338
C	3.254404	11.795110	12.438025
C	1.954710	12.053589	11.998465
H	1.395971	11.385063	11.331030
C	7.970860	10.033548	13.953071
C	9.162340	10.082435	14.696091
H	9.369140	10.958310	15.315498
C	10.097848	9.049325	14.665310
H	11.014230	9.122202	15.256390

C	9.850545	7.925171	13.873465
H	10.569801	7.103554	13.836967
C	8.681207	7.853308	13.123108
H	8.492260	6.978759	12.502687
C	7.725580	8.894993	13.144057
C	6.528364	8.704772	12.289439
C	5.399569	7.543763	10.720507
H	4.860792	6.591199	10.827752
H	5.778885	7.628193	9.688617
C	4.568575	8.776476	11.154267
C	3.309056	8.371776	11.928030
H	2.605265	7.848398	11.261032
H	2.827139	9.269989	12.337178
H	3.566774	7.700803	12.763078
C	4.235169	9.677990	9.965875
H	5.155635	9.995738	9.451533
H	3.707810	10.574932	10.315203
H	3.600482	9.134520	9.247128
C	6.442557	11.383058	15.932945
H	7.417801	11.298379	16.445354
C	5.619981	10.108942	16.218281
H	4.692709	10.158354	15.619479
H	6.167385	9.215545	15.875158
C	5.264478	9.973294	17.703442
H	4.651751	9.069346	17.858490
H	6.191177	9.828894	18.289798
C	4.530389	11.217480	18.214105
H	3.561071	11.305855	17.690510
H	4.302514	11.119473	19.288847
C	5.357875	12.480139	17.953794
H	6.287124	12.433870	18.552306
H	4.812327	13.377018	18.291994
C	5.718159	12.629067	16.468470
H	4.800850	12.788965	15.878264
H	6.347066	13.522667	16.335651
C	7.864068	12.938602	13.758264
H	8.543906	13.068467	14.621170
C	7.002380	14.208305	13.598021
H	6.405575	14.397356	14.500248
H	6.269770	14.029348	12.793800
C	7.855345	15.438917	13.267161
H	7.201133	16.318461	13.146874
H	8.527698	15.661604	14.117078
C	8.693263	15.215985	12.004504
H	9.327762	16.094096	11.797100
H	8.015934	15.103367	11.138236

C	9.551379	13.953974	12.137980
H	10.302609	14.107842	12.935127
H	10.116645	13.768051	11.209077
C	8.705596	12.720765	12.482994
H	9.360457	11.843122	12.590187
H	8.017615	12.499026	11.645915
C	3.909370	12.648618	13.261524
C	3.360412	13.840839	13.730224
H	3.888975	14.542094	14.386065
C	2.048652	14.124639	13.303914
H	1.557868	15.045215	13.636002
C	1.356634	13.244833	12.453904
H	0.337703	13.495896	12.141267

5a
B3LYP-D3/def2-SVP SCF energy (au): -3106.636692
B3LYP-D3/def2-SVP enthalpy (au): -3105.996626
B3LYP-D3/def2-SVP free energy (au): -3106.093341
ωB97X-D/def2-TZVP/SMD(THF) SCF energy (au): -3107.890155
ωB97X-D/def2-TZVP/SMD(THF) enthalpy (au): -3107.250089
ωB97X-D/def2-TZVP/SMD(THF) free energy (au): -3107.346804

Cartesian coordinates

ATOM	X	Y	Z
Ni	4.989516	11.198132	12.911293
P	6.806226	11.405949	14.081728
O	6.512569	7.561843	11.503757
N	5.399204	9.393029	12.256734
C	3.308797	11.881871	12.326499
C	2.060940	12.211581	11.797949
H	1.508886	11.557792	11.107636
C	8.008990	10.053145	13.892748
C	9.227000	10.110179	14.590988
H	9.414850	10.965989	15.248930
C	10.219926	9.137926	14.476017
H	11.154572	9.223528	15.036314
C	9.981835	8.038260	13.605487
H	10.744197	7.260936	13.486892
C	8.798945	7.935802	12.909341
H	8.631041	7.081868	12.252267
C	7.747462	8.916944	13.018719
C	6.543814	8.701000	12.298051
C	5.155803	7.318326	11.185092
H	4.737558	6.565618	11.883843
H	5.091983	6.918588	10.160044
C	4.460549	8.686589	11.357670

C	3.081183	8.526731	12.001258
H	2.415075	7.925278	11.357504
H	2.634229	9.515064	12.168929
H	3.176317	8.027663	12.978703
C	4.358801	9.435590	10.018883
H	5.349438	9.483098	9.538322
H	4.015227	10.462028	10.211563
H	3.655128	8.934138	9.330913
C	6.463110	11.348021	15.934871
H	7.440645	11.342278	16.452189
C	5.748952	10.009531	16.214977
H	4.839678	9.969077	15.587652
H	6.385183	9.172383	15.887912
C	5.364436	9.848519	17.689745
H	4.828559	8.894388	17.836834
H	6.283007	9.790365	18.304500
C	4.509784	11.024912	18.174112
H	3.554449	11.029117	17.617711
H	4.255250	10.910753	19.243042
C	5.233708	12.353315	17.931438
H	6.144975	12.384351	18.559526
H	4.603017	13.201116	18.252387
C	5.627163	12.527587	16.457139
H	4.720663	12.619818	15.834386
H	6.184592	13.470071	16.344187
C	7.841854	12.962102	13.801702
H	8.531272	13.065344	14.661832
C	6.976021	14.230609	13.694850
H	6.378694	14.382439	14.603465
H	6.242595	14.079776	12.886475
C	7.816492	15.482855	13.412887
H	7.156237	16.363831	13.329171
H	8.491690	15.675979	14.269045
C	8.654988	15.320066	12.140934
H	9.283048	16.212409	11.968052
H	7.974888	15.237883	11.273051
C	9.518574	14.056514	12.219483
H	10.271323	14.182553	13.021157
H	10.083926	13.915471	11.281398
C	8.679236	12.806620	12.515561
H	9.330660	11.923981	12.584346
H	7.988107	12.618605	11.672874
C	3.965192	12.723577	13.177408
C	3.442089	13.952070	13.576876
H	3.961837	14.638300	14.258167
C	2.177010	14.305004	13.059446

H	1.718679	15.259506	13.343951
C	1.497000	13.448693	12.180750
H	0.517465	13.748164	11.789099

4-Me-b

B3LYP-D3/def2-SVP SCF energy (au): -3145.898132
B3LYP-D3/def2-SVP enthalpy (au): -3145.224490
B3LYP-D3/def2-SVP free energy (au): -3145.322745
ωB97X-D/def2-TZVP/SMD(THF) SCF energy (au): -3147.137868
ωB97X-D/def2-TZVP/SMD(THF) enthalpy (au): -3146.464226
ωB97X-D/def2-TZVP/SMD(THF) free energy (au): -3146.562481

Cartesian coordinates

ATOM	X	Y	Z
Ni	9.857725	8.452214	11.617967
P	11.219682	10.123856	11.414268
N	9.958945	8.360238	13.590207
C	8.120434	5.867946	10.654487
C	8.863912	7.022635	10.902714
C	9.265036	7.868047	9.926863
C	8.959165	7.669537	8.569315
C	8.216188	6.498891	8.297541
H	7.944219	6.274849	7.260300
C	7.807564	5.615135	9.308693
H	7.234671	4.722355	9.037121
C	12.821856	9.648115	10.551871
H	13.500254	10.519811	10.585361
C	12.614161	9.233893	9.086728
H	12.230262	10.081416	8.499974
H	11.842290	8.446950	9.057450
C	13.908426	8.717195	8.444022
H	13.703530	8.405107	7.406303
H	14.642991	9.542230	8.384544
C	14.514507	7.560789	9.244438
H	13.820300	6.701107	9.220048
H	15.456589	7.221019	8.782282
C	14.747687	7.976551	10.700204
H	15.513543	8.773882	10.734164
H	15.148728	7.132622	11.286391
C	13.457014	8.488633	11.350643
H	13.658041	8.800508	12.387889
H	12.719721	7.666801	11.406433
C	10.585298	11.725286	10.669819
H	11.445516	12.385317	10.453797
C	9.818021	11.448830	9.364260
H	10.478093	10.996610	8.613851

H	9.043607	10.692904	9.577399
C	9.174878	12.715880	8.787994
H	9.967603	13.425694	8.485512
H	8.618145	12.460193	7.870841
C	8.253715	13.393243	9.806529
H	7.826096	14.320442	9.389346
H	7.402801	12.722725	10.026264
C	9.009887	13.687108	11.105843
H	8.336517	14.136915	11.854912
H	9.799617	14.435327	10.906131
C	9.655033	12.422098	11.686597
H	8.863498	11.706675	11.978188
H	10.202924	12.674972	12.606898
C	11.935430	10.725205	13.015364
C	12.849752	11.791767	12.989376
H	13.099434	12.258855	12.034187
C	13.452234	12.278581	14.148265
H	14.159770	13.109534	14.090044
C	13.143068	11.693595	15.378940
H	13.605995	12.059047	16.298642
C	12.236550	10.639919	15.433146
H	11.992345	10.188061	16.393058
C	11.618915	10.138027	14.264390
C	10.659944	9.023999	14.451740
C	9.179917	7.315714	14.334962
C	9.765003	5.939460	14.000446
H	9.204175	5.149361	14.525056
H	10.820932	5.884781	14.310499
H	9.711648	5.769643	12.917721
C	7.691891	7.407627	13.999661
H	7.124205	6.690516	14.614884
H	7.526333	7.188094	12.938502
H	7.314035	8.421415	14.204968
O	10.506916	8.658310	15.745790
C	9.443757	7.701063	15.810629
H	9.770689	6.856189	16.433789
H	8.573432	8.183099	16.286404
H	7.785672	5.175991	11.436872
C	9.319741	8.605845	7.440687
H	10.377823	8.902135	7.474126
H	9.129478	8.144511	6.459160
H	8.727136	9.535093	7.487141

4-Me-a

B3LYP-D3/def2-SVP SCF energy (au): -3145.899421

B3LYP-D3/def2-SVP enthalpy (au): -3145.225921

B3LYP-D3/def2-SVP free energy (au): -3145.325289
ωB97X-D/def2-TZVP/SMD(THF) SCF energy (au): -3147.139122
ωB97X-D/def2-TZVP/SMD(THF) enthalpy (au): -3146.465622
ωB97X-D/def2-TZVP/SMD(THF) free energy (au): -3146.564989

Cartesian coordinates

ATOM	X	Y	Z
Ni	9.829689	8.389293	11.732708
P	11.172590	10.047075	11.447026
N	9.941351	8.364573	13.700483
C	8.048237	5.873370	10.503524
C	8.817744	6.974408	10.917955
C	9.321940	7.865954	10.034236
C	9.130911	7.796770	8.654570
H	9.527589	8.526216	7.940525
C	8.363466	6.709068	8.205563
H	8.165206	6.586398	7.135812
C	7.839366	5.771167	9.110876
H	7.247211	4.935946	8.720840
C	12.746365	9.572940	10.535857
H	13.460130	10.408666	10.647460
C	12.541395	9.310175	9.035253
H	12.199718	10.226589	8.529987
H	11.747648	8.556219	8.909715
C	13.832343	8.814877	8.367055
H	13.639097	8.607649	7.301170
H	14.591775	9.618775	8.397780
C	14.390873	7.570139	9.063922
H	13.670925	6.739178	8.951275
H	15.328223	7.245189	8.581822
C	14.617164	7.838678	10.555411
H	15.406443	8.604550	10.673598
H	14.983676	6.930396	11.062641
C	13.331186	8.326544	11.232793
H	13.518714	8.537085	12.298721
H	12.569742	7.526651	11.199995
C	10.508998	11.615734	10.657338
H	11.368602	12.236740	10.342768
C	9.648761	11.280363	9.422070
H	10.235173	10.737044	8.669338
H	8.851882	10.585062	9.733596
C	9.032934	12.537517	8.795823
H	9.838444	13.188230	8.406305
H	8.414921	12.251318	7.928521
C	8.199317	13.320449	9.814689
H	7.790926	14.239240	9.361120

H	7.332571	12.705765	10.119402
C	9.034573	13.659176	11.053369
H	8.419812	14.180689	11.806411
H	9.840463	14.360878	10.767771
C	9.663577	12.406656	11.678135
H	8.864372	11.742173	12.056311
H	10.270046	12.694269	12.550064
C	11.929673	10.686135	13.015674
C	12.849865	11.745508	12.942267
H	13.081943	12.184610	11.969283
C	13.477017	12.262445	14.074881
H	14.187966	13.086949	13.978934
C	13.187544	11.716079	15.327806
H	13.670849	12.104636	16.227334
C	12.273183	10.672715	15.430478
H	12.042392	10.252427	16.408032
C	11.629102	10.141736	14.289466
C	10.657282	9.049281	14.531379
C	9.146022	7.364531	14.480945
C	9.687217	5.966270	14.166458
H	9.101378	5.198061	14.695785
H	10.741245	5.883087	14.476175
H	9.626604	5.792995	13.084569
C	7.661968	7.498054	14.141077
H	7.069174	6.779039	14.729131
H	7.512204	7.308804	13.070523
H	7.305634	8.515744	14.364723
O	10.505012	8.734234	15.838826
C	9.439525	7.780450	15.943348
H	9.777359	6.947644	16.577067
H	8.580535	8.274275	16.426995
C	7.481394	4.833778	11.442966
H	6.817189	4.127804	10.920175
H	8.283418	4.243095	11.918603
H	6.905426	5.295937	12.261010

4-OMe-b

B3LYP-D3/def2-SVP SCF energy (au): -3221.055572
B3LYP-D3/def2-SVP enthalpy (au): -3220.376097
B3LYP-D3/def2-SVP free energy (au): -3220.477018
ωB97X-D/def2-TZVP/SMD(THF) SCF energy (au): -3222.355998
ωB97X-D/def2-TZVP/SMD(THF) enthalpy (au): -3221.676523
ωB97X-D/def2-TZVP/SMD(THF) free energy (au): -3221.777444

Cartesian coordinates

ATOM X Y Z

N	9.302536	9.945963	7.260552
C	11.532712	8.236973	4.792604
C	11.918435	8.460981	6.068547
Ni	10.163520	9.094429	5.714556
P	8.569112	9.472353	4.305573
O	7.951095	11.146986	8.585668
C	13.167593	8.079243	6.568453
C	8.212592	10.635435	7.360096
C	8.922643	10.621466	9.499019
H	8.417988	9.898077	10.161049
H	9.315604	11.449794	10.105954
C	9.988450	9.958296	8.593340
C	10.303368	8.533348	9.047045
H	9.386478	7.923819	9.068480
H	10.739679	8.547947	10.059102
H	11.011315	8.066140	8.350966
C	11.254907	10.815008	8.487450
H	11.922205	10.391609	7.725142
H	11.776373	10.842960	9.457704
H	11.000248	11.846480	8.195731
C	7.663311	7.987748	3.599500
H	7.152579	8.308428	2.671986
C	6.609905	7.449391	4.588948
H	7.115087	7.213456	5.544212
H	5.853448	8.215339	4.816371
C	5.917874	6.189787	4.049069
H	5.191521	5.817586	4.791451
H	5.333482	6.461680	3.150112
C	6.923734	5.093596	3.681802
H	7.427922	4.743312	4.601177
H	6.401992	4.219877	3.255807
C	7.979721	5.625927	2.707626
H	7.496726	5.871882	1.742934
H	8.732416	4.849483	2.490956
C	8.675973	6.872754	3.264363
H	9.218352	6.615549	4.190198
H	9.453798	7.221824	2.574446
C	9.064311	10.554788	2.853514
H	8.136774	10.962856	2.414573
C	9.825966	9.805512	1.749720
H	9.188428	9.020929	1.311790
H	10.697712	9.292958	2.182969
C	10.288523	10.763433	0.642220
H	10.858967	10.201223	-0.116405
H	9.405442	11.182435	0.123529
C	11.131633	11.913686	1.203385

H	12.059719	11.501255	1.639562
H	11.437996	12.599219	0.395193
C	10.363042	12.672923	2.290584
H	9.481564	13.165391	1.838803
H	10.986448	13.476509	2.717545
C	9.901997	11.725940	3.404764
H	10.782997	11.303978	3.920096
H	9.328399	12.279968	4.167238
C	14.020571	7.433378	5.660865
H	15.015060	7.105253	5.981499
C	13.629812	7.187896	4.330908
H	14.330164	6.678625	3.666867
C	12.358169	7.591885	3.872134
C	7.195967	10.506004	5.002529
C	6.121770	10.857319	4.167212
H	6.110070	10.497468	3.135677
C	5.061697	11.644929	4.613093
H	4.243518	11.897801	3.934050
C	5.058059	12.104446	5.932567
H	4.238961	12.726669	6.300921
C	6.101946	11.760873	6.785330
H	6.092612	12.111011	7.816226
C	7.180732	10.959221	6.345933
O	11.911285	7.384070	2.589119
C	12.749144	6.738908	1.662966
H	13.689709	7.296296	1.493489
H	13.006699	5.711269	1.981570
H	12.193495	6.686294	0.715446
H	13.492268	8.256238	7.600311

4-OMe-a

B3LYP-D3/def2-SVP SCF energy (au): -3221.055204
B3LYP-D3/def2-SVP enthalpy (au): -3220.375827
B3LYP-D3/def2-SVP free energy (au): -3220.477402
ωB97X-D/def2-TZVP/SMD(THF) SCF energy (au): -3222.357607
ωB97X-D/def2-TZVP/SMD(THF) enthalpy (au): -3221.678230
ωB97X-D/def2-TZVP/SMD(THF) free energy (au): -3221.779805

Cartesian coordinates

ATOM	X	Y	Z
N	9.288649	9.939300	7.130281
C	11.407055	8.106510	4.647772
C	11.843525	8.367013	5.899090
Ni	10.094262	9.025728	5.586276
P	8.513702	9.432314	4.191429
O	7.969470	11.183493	8.448016

C	13.112252	7.966528	6.313935
C	8.200845	10.630275	7.235142
C	8.971755	10.698538	9.351044
H	8.489836	10.004901	10.060141
H	9.382489	11.553196	9.907769
C	10.011344	9.995797	8.443490
C	10.332890	8.589160	8.948106
H	9.422197	7.970386	8.973257
H	10.746885	8.648404	9.968135
H	11.074025	8.112980	8.295985
C	11.281958	10.836130	8.276621
H	11.956569	10.349021	7.561759
H	11.799736	10.926435	9.245083
H	11.033208	11.846314	7.913484
C	7.598110	7.974395	3.442772
H	7.034185	8.333008	2.561295
C	6.607276	7.383431	4.468054
H	7.170552	7.117320	5.381899
H	5.860299	8.133279	4.768691
C	5.895061	6.137772	3.924016
H	5.216820	5.732271	4.693781
H	5.257144	6.433700	3.070219
C	6.889229	5.067683	3.462306
H	7.452954	4.695528	4.337300
H	6.354549	4.200669	3.039045
C	7.875230	5.646595	2.443036
H	7.328613	5.919512	1.520662
H	8.620732	4.887870	2.152037
C	8.593363	6.882950	2.997426
H	9.210272	6.602681	3.867171
H	9.299045	7.272177	2.251379
C	9.034350	10.558783	2.781333
H	8.118166	10.876758	2.251883
C	9.971607	9.881419	1.768213
H	9.456658	9.044756	1.271737
H	10.832601	9.455441	2.308999
C	10.468129	10.872206	0.705276
H	11.161201	10.358387	0.018192
H	9.611661	11.210346	0.092025
C	11.144545	12.092398	1.338003
H	12.054399	11.764639	1.873068
H	11.472765	12.800126	0.558204
C	10.201211	12.782249	2.328929
H	9.333051	13.194088	1.781067
H	10.702249	13.638567	2.810900
C	9.706904	11.802500	3.399626

```
H    10.561302    11.461590     4.011728
H     9.011586    12.310661     4.087974
C    14.780899     7.858780     8.030973
H    14.909976     6.760288     7.991229
H    14.882812     8.188003     9.075327
H    15.587658     8.323404     7.432502
O    13.504785     8.257974     7.598989
C    13.916702     7.283165     5.377369
H    14.921059     6.943335     5.636351
C    13.431341     7.022115     4.080852
H    14.078388     6.485160     3.379285
C    12.150833     7.429780     3.677229
H    11.789790     7.209040     2.667691
C     7.139120    10.455341     4.897765
C     6.054543    10.795979     4.072015
H     6.034037    10.432777     3.041858
C     4.995180    11.580028     4.526144
H     4.167670    11.824852     3.855516
C     5.004755    12.046540     5.843017
H     4.185425    12.664265     6.218350
C     6.063622    11.718768     6.683848
H     6.066627    12.078620     7.711392
C     7.143038    10.922835     6.236386
```

Cartesian Coordinates – Distortion/Interaction Analysis

distorted 4-CN-a complex

```
ωB97X-D/def2-TZVP/SMD(THF) SCF energy (au):               -2876.727556
Cartesian coordinates
ATOM  X             Y           Z
Ni     9.834002     8.402735    11.709768
P    11.183613    10.064648    11.444266
N     9.939457     8.346036    13.677888
C    12.763144     9.592993    10.546877
H    13.471792    10.432476    10.662696
C    12.567353     9.328945     9.045031
H    12.225604    10.244042     8.537352
H    11.776419     8.572136     8.915879
C    13.863564     8.836428     8.384932
H    13.677089     8.627393     7.318263
H    14.619471     9.643270     8.418780
C    14.422415     7.594759     9.087020
H    13.706742     6.760449     8.971394
H    15.363464     7.272778     8.610610
C    14.639796     7.866061    10.579297
```

H	15.425374	8.635025	10.700857
H	15.006619	6.960006	11.089817
C	13.348700	8.350114	11.249662
H	13.530326	8.563822	12.315849
H	12.591256	7.546359	11.215325
C	10.511452	11.621123	10.641772
H	11.370532	12.238644	10.319251
C	9.651666	11.269740	9.410056
H	10.235270	10.709205	8.667451
H	8.849090	10.586267	9.732980
C	9.044496	12.521008	8.763970
H	9.854830	13.161638	8.368168
H	8.427969	12.226013	7.898625
C	8.211179	13.321364	9.769601
H	7.810007	14.236327	9.302466
H	7.339484	12.715687	10.078078
C	9.042428	13.671881	11.007740
H	8.426276	14.204311	11.751678
H	9.852079	14.367235	10.717545
C	9.664684	12.425260	11.651107
H	8.862028	11.768144	12.034641
H	10.268701	12.721936	12.521466
C	11.912292	10.698557	13.024177
C	12.819258	11.769999	12.966090
H	13.055798	12.218185	11.998265
C	13.426472	12.288061	14.109095
H	14.126981	13.122784	14.026562
C	13.130037	11.729646	15.354685
H	13.597127	12.119219	16.262219
C	12.230171	10.671783	15.440492
H	11.995954	10.241042	16.412589
C	11.606047	10.139168	14.289808
C	10.653048	9.023581	14.516028
C	9.171854	7.307604	14.443613
C	9.738214	5.926607	14.098270
H	9.168465	5.138305	14.612935
H	10.796922	5.860101	14.396826
H	9.667993	5.757559	13.017111
C	7.679125	7.416216	14.135760
H	7.115747	6.673498	14.721234
H	7.499001	7.229670	13.071287
H	7.308270	8.423797	14.381643
O	10.522724	8.677409	15.814252
C	9.476764	7.698163	15.910900
H	9.840966	6.859491	16.521215
H	8.616067	8.165106	16.416838

distorted 4-CN-b complex

ωB97X-D/def2-TZVP/SMD(THF) SCF energy (au): -2876.726905

Cartesian coordinates

ATOM	X	Y	Z
Ni	9.879536	8.403695	11.639351
P	11.288120	10.038037	11.391122
N	10.007155	8.313574	13.605998
C	12.873102	9.627417	10.478317
H	13.453396	10.561044	10.362245
C	12.600491	9.029268	9.086860
H	12.064464	9.737804	8.444849
H	11.929747	8.162082	9.206577
C	13.892501	8.588290	8.388624
H	13.640822	8.149910	7.409320
H	14.519716	9.476059	8.183185
C	14.687060	7.596705	9.243001
H	14.099622	6.667495	9.359864
H	15.627682	7.314507	8.740824
C	14.974577	8.185848	10.627698
H	15.646198	9.057993	10.520231
H	15.509240	7.456426	11.259430
C	13.687170	8.631302	11.333418
H	13.933012	9.068351	12.313815
H	13.051880	7.747936	11.532046
C	10.554164	11.615750	10.691076
H	11.365254	12.362951	10.618293
C	9.934464	11.413466	9.298366
H	10.687537	11.092064	8.568679
H	9.199581	10.594492	9.360116
C	9.242589	12.683923	8.789554
H	9.997933	13.477670	8.637121
H	8.798059	12.480544	7.801820
C	8.180127	13.181357	9.773810
H	7.714290	14.111765	9.407869
H	7.372589	12.429754	9.844807
C	8.788314	13.396895	11.163417
H	8.014785	13.713279	11.883492
H	9.525847	14.219689	11.114979
C	9.484790	12.131109	11.679357
H	8.735628	11.330461	11.823600
H	9.929930	12.327192	12.667597
C	11.968922	10.684699	12.988711
C	12.884501	11.749146	12.944678
H	13.179934	12.161446	11.977611
C	13.432604	12.301436	14.101398

H	14.141723	13.129850	14.029117
C	13.066361	11.786792	15.347323
H	13.483835	12.206558	16.265497
C	12.164501	10.729877	15.418375
H	11.882921	10.327311	16.389890
C	11.602607	10.161224	14.252523
C	10.663950	9.031542	14.458818
C	9.195794	7.306011	14.364307
C	9.557486	5.890051	13.912820
H	9.004259	5.150324	14.513854
H	10.636223	5.708614	14.040057
H	9.308933	5.757485	12.852845
C	7.706944	7.606422	14.166442
H	7.096407	6.886631	14.734988
H	7.452499	7.539295	13.100982
H	7.468283	8.622823	14.517793
O	10.483495	8.713706	15.758443
C	9.637298	7.559393	15.828836
H	10.223797	6.727485	16.250513
H	8.798994	7.783965	16.504873

distorted 4-CN-b aryne
ωB97X-D/def2-TZVP/SMD(THF) SCF energy (au): -323.125352
Cartesian coordinates

ATOM	X	Y	Z
C	-0.651026	0.000000	-0.441787
C	-1.640585	0.863219	0.023684
C	-1.869917	1.066736	1.344746
C	-1.123032	0.412750	2.335493
C	-0.106558	-0.474423	1.892052
H	0.499184	-1.004892	2.631020
C	0.121881	-0.672265	0.528052
H	0.912617	-1.360938	0.215749
H	-0.453514	-0.173849	-1.506652
C	-1.334959	0.593856	3.744041
N	-1.515373	0.749807	4.882328

distorted 4-CN-a aryne
ωB97X-D/def2-TZVP/SMD(THF) SCF energy (au): -323.125777
Cartesian coordinates

ATOM	X	Y	Z
N	-1.454761	0.000000	-5.139551
C	-1.643662	0.195781	-2.552985
C	-2.521336	1.116918	-1.963979
C	-2.620569	1.217709	-0.615188
C	-1.877576	0.446821	0.274007

H	-1.946814	0.536923	1.363513
C	-0.989298	-0.488461	-0.298032
H	-0.378572	-1.124049	0.349935
C	-0.871037	-0.619135	-1.685208
H	-0.177615	-1.350552	-2.108116
C	-1.521130	0.068045	-3.980409

distorted 4-F-a complex

ωB97X-D/def2-TZVP/SMD(THF) SCF energy (au): -2876.729020

Cartesian coordinates

ATOM	X	Y	Z
P	3.025899	11.597821	8.818732
Ni	2.625718	10.284376	7.144949
C	1.575736	12.162336	9.840622
H	1.936724	12.655974	10.758874
O	6.401566	8.709079	7.906717
N	4.396661	9.428772	7.212759
C	0.736897	10.928439	10.228240
H	0.486373	10.382383	9.302458
H	1.339430	10.241463	10.845764
C	-0.549995	11.317623	10.964631
H	-0.295047	11.777770	11.938031
H	-1.136770	10.411700	11.190220
C	-1.381198	12.306453	10.140316
H	-2.296152	12.592816	10.685435
H	-1.708116	11.811865	9.208017
C	-0.557694	13.550765	9.790384
H	-1.150289	14.247225	9.173762
H	-0.307994	14.092518	10.722059
C	0.739550	13.190101	9.052935
H	1.325480	14.106644	8.876984
H	0.503187	12.771447	8.062619
C	3.962842	13.158933	8.375251
H	3.249429	13.687767	7.717720
C	4.332641	14.079015	9.548091
H	4.996638	13.532708	10.240977
H	3.435427	14.358010	10.124203
C	5.050246	15.345446	9.056494
H	4.348108	15.944816	8.448178
H	5.334194	15.974391	9.917001
C	6.283030	15.002286	8.211598
H	7.027873	14.491769	8.850196
H	6.764951	15.923301	7.843284
C	5.913659	14.084572	7.039750
H	6.813584	13.812816	6.462756
H	5.248602	14.629494	6.345018

C	5.199830	12.816286	7.523865
H	5.903577	12.215935	8.129376
H	4.896751	12.183615	6.675093
C	4.166346	10.771236	10.025421
C	4.147007	11.102983	11.389336
H	3.432791	11.841822	11.753619
C	5.021198	10.515360	12.306435
H	4.968876	10.793511	13.362019
C	5.960744	9.582655	11.865784
H	6.655006	9.118181	12.569880
C	6.010051	9.242298	10.515941
H	6.742744	8.516032	10.165321
C	5.116850	9.810921	9.585609
C	5.242178	9.345545	8.184773
C	6.421875	8.468393	6.489681
H	6.799829	7.452120	6.312592
H	7.108510	9.198833	6.028635
C	4.955503	8.671856	6.045658
C	4.210583	7.337280	5.924306
H	3.148872	7.519054	5.719236
H	4.305111	6.753869	6.854010
H	4.632907	6.748470	5.094312
C	4.851486	9.486767	4.758725
H	5.374865	8.965185	3.941153
H	5.305080	10.481820	4.888060
H	3.797147	9.612397	4.481779

distorted 4-F-b complex
ωB97X-D/def2-TZVP/SMD(THF) SCF energy (au): -2876.728244
Cartesian coordinates

ATOM	X	Y	Z
P	3.011810	11.600174	8.820182
Ni	2.614185	10.272560	7.136552
C	1.569887	12.180884	9.843531
H	1.944405	12.648299	10.770238
O	6.403258	8.737170	7.893353
N	4.394234	9.449607	7.204967
C	0.701879	10.961267	10.211482
H	0.424014	10.447466	9.275697
H	1.291576	10.241644	10.804456
C	-0.563975	11.370673	10.973392
H	-0.281732	11.797101	11.954936
H	-1.174049	10.476412	11.184294
C	-1.375598	12.402952	10.183250
H	-2.272040	12.704793	10.750821
H	-1.723649	11.947258	9.241398

C	-0.518571	13.629211	9.849551
H	-1.098904	14.352995	9.253506
H	-0.237952	14.145732	10.787455
C	0.753581	13.243087	9.081063
H	1.363147	14.145006	8.908802
H	0.471417	12.844529	8.095830
C	3.964134	13.153146	8.379142
H	3.250959	13.692536	7.730066
C	4.347521	14.061295	9.557078
H	5.011606	13.504431	10.241542
H	3.455840	14.341723	10.140580
C	5.071057	15.326567	9.071544
H	4.369478	15.935674	8.472409
H	5.364475	15.946566	9.935362
C	6.296438	14.981899	8.216640
H	7.041668	14.461291	8.846641
H	6.782495	15.902397	7.852414
C	5.913709	14.075854	7.040043
H	6.808321	13.802603	6.455346
H	5.248072	14.630711	6.353798
C	5.193951	12.808733	7.518714
H	5.897534	12.199478	8.115541
H	4.882178	12.184802	6.666756
C	4.147712	10.763061	10.026215
C	4.118264	11.076656	11.394091
H	3.397575	11.806373	11.763608
C	4.990280	10.482349	12.309128
H	4.929148	10.746877	13.367751
C	5.938758	9.561184	11.863494
H	6.631580	9.092556	12.566250
C	5.998040	9.237688	10.509976
H	6.736908	8.519845	10.154824
C	5.106368	9.812536	9.582053
C	5.239083	9.363105	8.177970
C	6.428125	8.514206	6.473747
H	6.817199	7.504318	6.284144
H	7.106939	9.257612	6.022129
C	4.960246	8.708900	6.033037
C	4.221041	7.370947	5.913443
H	3.144481	7.558178	5.810024
H	4.382188	6.757796	6.814405
H	4.583693	6.810506	5.037078
C	4.846280	9.535808	4.754959
H	5.343135	9.014217	3.920983
H	5.321545	10.520481	4.885062
H	3.788734	9.693499	4.504260

distorted 4-F-b aryne

ωB97X-D/def2-TZVP/SMD(THF) SCF energy (au): -330.140313

Cartesian coordinates

ATOM	X	Y	Z
C	0.876071	0.000000	-0.890418
C	1.724117	0.697000	-0.054093
C	1.578898	0.584686	1.334699
H	2.254837	1.143434	1.986440
C	0.574248	-0.241736	1.861951
H	0.468965	-0.322902	2.948559
C	-0.286413	-0.962376	1.012546
C	-0.083352	-0.803476	-0.358656
H	-1.063477	-1.605780	1.440571
F	2.716820	1.511149	-0.520885

distorted 4-F-a aryne

ωB97X-D/def2-TZVP/SMD(THF) SCF energy (au): -330.140910

Cartesian coordinates

ATOM	X	Y	Z
C	-0.843922	0.000000	-1.280301
C	0.039759	0.750755	-0.503433
H	0.813308	1.407456	-0.913998
C	-0.103096	0.628689	0.891804
H	0.559432	1.189488	1.558680
C	-1.085683	-0.204788	1.450798
H	-1.199644	-0.300174	2.533398
F	-2.885640	-1.726143	1.166482
C	-1.934799	-0.927843	0.603033
C	-1.798935	-0.817440	-0.764425

distorted 4-H complex

ωB97X-D/def2-TZVP/SMD(THF) SCF energy (au): -2876.728164

Cartesian coordinates

ATOM	X	Y	Z
Ni	5.003178	11.198386	12.890848
P	6.775572	11.444659	14.085388
O	6.522807	7.533562	11.611766
N	5.506179	9.472144	12.093338
C	7.970860	10.033548	13.953071
C	9.162340	10.082435	14.696091
H	9.369140	10.958310	15.315498
C	10.097848	9.049325	14.665310
H	11.014230	9.122202	15.256390
C	9.850545	7.925171	13.873465
H	10.569801	7.103554	13.836967

C	8.681207	7.853308	13.123108
H	8.492260	6.978759	12.502687
C	7.725580	8.894993	13.144057
C	6.528364	8.704772	12.289439
C	5.399569	7.543763	10.720507
H	4.860792	6.591199	10.827752
H	5.778885	7.628193	9.688617
C	4.568575	8.776476	11.154267
C	3.309056	8.371776	11.928030
H	2.605265	7.848398	11.261032
H	2.827139	9.269989	12.337178
H	3.566774	7.700803	12.763078
C	4.235169	9.677990	9.965875
H	5.155635	9.995738	9.451533
H	3.707810	10.574932	10.315203
H	3.600482	9.134520	9.247128
C	6.442557	11.383058	15.932945
H	7.417801	11.298379	16.445354
C	5.619981	10.108942	16.218281
H	4.692709	10.158354	15.619479
H	6.167385	9.215545	15.875158
C	5.264478	9.973294	17.703442
H	4.651751	9.069346	17.858490
H	6.191177	9.828894	18.289798
C	4.530389	11.217480	18.214105
H	3.561071	11.305855	17.690510
H	4.302514	11.119473	19.288847
C	5.357875	12.480139	17.953794
H	6.287124	12.433870	18.552306
H	4.812327	13.377018	18.291994
C	5.718159	12.629067	16.468470
H	4.800850	12.788965	15.878264
H	6.347066	13.522667	16.335651
C	7.864068	12.938602	13.758264
H	8.543906	13.068467	14.621170
C	7.002380	14.208305	13.598021
H	6.405575	14.397356	14.500248
H	6.269770	14.029348	12.793800
C	7.855345	15.438917	13.267161
H	7.201133	16.318461	13.146874
H	8.527698	15.661604	14.117078
C	8.693263	15.215985	12.004504
H	9.327762	16.094096	11.797100
H	8.015934	15.103367	11.138236
C	9.551379	13.953974	12.137980
H	10.302609	14.107842	12.935127

H	10.116645	13.768051	11.209077
C	8.705596	12.720765	12.482994
H	9.360457	11.843122	12.590187
H	8.017615	12.499026	11.645915

distorted 4-H aryne
ωB97X-D/def2-TZVP/SMD(THF) SCF energy (au): -230.887343
Cartesian coordinates

ATOM	X	Y	Z
C	0.699250	0.000000	0.640517
C	-0.237973	-0.655995	1.440837
H	-1.076616	-1.238900	1.038985
C	1.719770	0.710834	1.177975
C	1.919821	0.855180	2.549678
H	2.735439	1.439101	2.991342
C	0.989680	0.200853	3.380154
H	1.089354	0.273081	4.468024
C	-0.070290	-0.543903	2.834932
H	-0.775938	-1.040252	3.509241

distorted 4-Me-a complex
ωB97X-D/def2-TZVP/SMD(THF) SCF energy (au): -2876.727772
Cartesian coordinates

ATOM	X	Y	Z
Ni	9.829689	8.389293	11.732708
P	11.172590	10.047075	11.447026
N	9.941351	8.364573	13.700483
C	12.746365	9.572940	10.535857
H	13.460130	10.408666	10.647460
C	12.541395	9.310175	9.035253
H	12.199718	10.226589	8.529987
H	11.747648	8.556219	8.909715
C	13.832343	8.814877	8.367055
H	13.639097	8.607649	7.301170
H	14.591775	9.618775	8.397780
C	14.390873	7.570139	9.063922
H	13.670925	6.739178	8.951275
H	15.328223	7.245189	8.581822
C	14.617164	7.838678	10.555411
H	15.406443	8.604550	10.673598
H	14.983676	6.930396	11.062641
C	13.331186	8.326544	11.232793
H	13.518714	8.537085	12.298721
H	12.569742	7.526651	11.199995
C	10.508998	11.615734	10.657338
H	11.368602	12.236740	10.342768

C	9.648761	11.280363	9.422070
H	10.235173	10.737044	8.669338
H	8.851882	10.585062	9.733596
C	9.032934	12.537517	8.795823
H	9.838444	13.188230	8.406305
H	8.414921	12.251318	7.928521
C	8.199317	13.320449	9.814689
H	7.790926	14.239240	9.361120
H	7.332571	12.705765	10.119402
C	9.034573	13.659176	11.053369
H	8.419812	14.180689	11.806411
H	9.840463	14.360878	10.767771
C	9.663577	12.406656	11.678135
H	8.864372	11.742173	12.056311
H	10.270046	12.694269	12.550064
C	11.929673	10.686135	13.015674
C	12.849865	11.745508	12.942267
H	13.081943	12.184610	11.969283
C	13.477017	12.262445	14.074881
H	14.187966	13.086949	13.978934
C	13.187544	11.716079	15.327806
H	13.670849	12.104636	16.227334
C	12.273183	10.672715	15.430478
H	12.042392	10.252427	16.408032
C	11.629102	10.141736	14.289466
C	10.657282	9.049281	14.531379
C	9.146022	7.364531	14.480945
C	9.687217	5.966270	14.166458
H	9.101378	5.198061	14.695785
H	10.741245	5.883087	14.476175
H	9.626604	5.792995	13.084569
C	7.661968	7.498054	14.141077
H	7.069174	6.779039	14.729131
H	7.512204	7.308804	13.070523
H	7.305634	8.515744	14.364723
O	10.505012	8.734234	15.838826
C	9.439525	7.780450	15.943348
H	9.777359	6.947644	16.577067
H	8.580535	8.274275	16.426995

distorted 4-Me-b complex

ωB97X-D/def2-TZVP/SMD(THF) SCF energy (au): -2876.726835

Cartesian coordinates

ATOM	X	Y	Z
Ni	9.857725	8.452214	11.617967
P	11.219682	10.123856	11.414268

N	9.958945	8.360238	13.590207
C	12.821856	9.648115	10.551871
H	13.500254	10.519811	10.585361
C	12.614161	9.233893	9.086728
H	12.230262	10.081416	8.499974
H	11.842290	8.446950	9.057450
C	13.908426	8.717195	8.444022
H	13.703530	8.405107	7.406303
H	14.642991	9.542230	8.384544
C	14.514507	7.560789	9.244438
H	13.820300	6.701107	9.220048
H	15.456589	7.221019	8.782282
C	14.747687	7.976551	10.700204
H	15.513543	8.773882	10.734164
H	15.148728	7.132622	11.286391
C	13.457014	8.488633	11.350643
H	13.658041	8.800508	12.387889
H	12.719721	7.666801	11.406433
C	10.585298	11.725286	10.669819
H	11.445516	12.385317	10.453797
C	9.818021	11.448830	9.364260
H	10.478093	10.996610	8.613851
H	9.043607	10.692904	9.577399
C	9.174878	12.715880	8.787994
H	9.967603	13.425694	8.485512
H	8.618145	12.460193	7.870841
C	8.253715	13.393243	9.806529
H	7.826096	14.320442	9.389346
H	7.402801	12.722725	10.026264
C	9.009887	13.687108	11.105843
H	8.336517	14.136915	11.854912
H	9.799617	14.435327	10.906131
C	9.655033	12.422098	11.686597
H	8.863498	11.706675	11.978188
H	10.202924	12.674972	12.606898
C	11.935430	10.725205	13.015364
C	12.849752	11.791767	12.989376
H	13.099434	12.258855	12.034187
C	13.452234	12.278581	14.148265
H	14.159770	13.109534	14.090044
C	13.143068	11.693595	15.378940
H	13.605995	12.059047	16.298642
C	12.236550	10.639919	15.433146
H	11.992345	10.188061	16.393058
C	11.618915	10.138027	14.264390
C	10.659944	9.023999	14.451740

C	9.179917	7.315714	14.334962
C	9.765003	5.939460	14.000446
H	9.204175	5.149361	14.525056
H	10.820932	5.884781	14.310499
H	9.711648	5.769643	12.917721
C	7.691891	7.407627	13.999661
H	7.124205	6.690516	14.614884
H	7.526333	7.188094	12.938502
H	7.314035	8.421415	14.204968
O	10.506916	8.658310	15.745790
C	9.443757	7.701063	15.810629
H	9.770689	6.856189	16.433789
H	8.573432	8.183099	16.286404

distorted 4-Me-b aryne

ωB97X-D/def2-TZVP/SMD(THF) SCF energy (au): -270.209450

Cartesian coordinates

ATOM	X	Y	Z
C	0.217009	0.000000	-1.907819
C	-0.564752	1.009663	-1.344691
C	-0.712280	1.173789	-0.010823
C	-0.080414	0.348980	0.935928
C	0.706680	-0.686365	0.383814
H	1.226540	-1.372953	1.060846
C	0.852751	-0.863931	-1.000946
H	1.474987	-1.685694	-1.370461
H	0.348042	-0.141869	-2.987493
C	-0.150897	0.517150	2.435007
H	-1.184425	0.647235	2.786206
H	0.276023	-0.352856	2.957743
H	0.411848	1.406850	2.764541

distorted 4-Me-a aryne

ωB97X-D/def2-TZVP/SMD(THF) SCF energy (au): -270.209324

Cartesian coordinates

ATOM	X	Y	Z
C	0.281307	0.000000	-2.791179
C	-0.541332	1.136046	-2.697146
C	-0.908315	1.654659	-1.502830
C	-0.513974	1.138325	-0.268873
H	-0.799903	1.566446	0.697699
C	0.311775	0.003178	-0.325931
H	0.667118	-0.459620	0.600499
C	0.695225	-0.550852	-1.558793
H	1.338857	-1.437476	-1.560886
C	0.701941	-0.636195	-4.096145

H	1.436588	-1.441887	-3.941121
H	-0.161967	-1.073632	-4.625948
H	1.149676	0.101008	-4.782142

distorted 4-OMe-a complex

ωB97X-D/def2-TZVP/SMD(THF) SCF energy (au): -2876.728512

Cartesian coordinates

ATOM	X	Y	Z
N	9.288649	9.939300	7.130281
Ni	10.094262	9.025728	5.586276
P	8.513702	9.432314	4.191429
O	7.969470	11.183493	8.448016
C	8.200845	10.630275	7.235142
C	8.971755	10.698538	9.351044
H	8.489836	10.004901	10.060141
H	9.382489	11.553196	9.907769
C	10.011344	9.995797	8.443490
C	10.332890	8.589160	8.948106
H	9.422197	7.970386	8.973257
H	10.746885	8.648404	9.968135
H	11.074025	8.112980	8.295985
C	11.281958	10.836130	8.276621
H	11.956569	10.349021	7.561759
H	11.799736	10.926435	9.245083
H	11.033208	11.846314	7.913484
C	7.598110	7.974395	3.442772
H	7.034185	8.333008	2.561295
C	6.607276	7.383431	4.468054
H	7.170552	7.117320	5.381899
H	5.860299	8.133279	4.768691
C	5.895061	6.137772	3.924016
H	5.216820	5.732271	4.693781
H	5.257144	6.433700	3.070219
C	6.889229	5.067683	3.462306
H	7.452954	4.695528	4.337300
H	6.354549	4.200669	3.039045
C	7.875230	5.646595	2.443036
H	7.328613	5.919512	1.520662
H	8.620732	4.887870	2.152037
C	8.593363	6.882950	2.997426
H	9.210272	6.602681	3.867171
H	9.299045	7.272177	2.251379
C	9.034350	10.558783	2.781333
H	8.118166	10.876758	2.251883
C	9.971607	9.881419	1.768213
H	9.456658	9.044756	1.271737

H	10.832601	9.455441	2.308999
C	10.468129	10.872206	0.705276
H	11.161201	10.358387	0.018192
H	9.611661	11.210346	0.092025
C	11.144545	12.092398	1.338003
H	12.054399	11.764639	1.873068
H	11.472765	12.800126	0.558204
C	10.201211	12.782249	2.328929
H	9.333051	13.194088	1.781067
H	10.702249	13.638567	2.810900
C	9.706904	11.802500	3.399626
H	10.561302	11.461590	4.011728
H	9.011586	12.310661	4.087974
C	7.139120	10.455341	4.897765
C	6.054543	10.795979	4.072015
H	6.034037	10.432777	3.041858
C	4.995180	11.580028	4.526144
H	4.167670	11.824852	3.855516
C	5.004755	12.046540	5.843017
H	4.185425	12.664265	6.218350
C	6.063622	11.718768	6.683848
H	6.066627	12.078620	7.711392
C	7.143038	10.922835	6.236386

distorted 4-OMe-b complex
ωB97X-D/def2-TZVP/SMD(THF) SCF energy (au): -2876.727437
Cartesian coordinates

ATOM	X	Y	Z
N	9.302536	9.945963	7.260552
Ni	10.163520	9.094429	5.714556
P	8.569112	9.472353	4.305573
O	7.951095	11.146986	8.585668
C	8.212592	10.635435	7.360096
C	8.922643	10.621466	9.499019
H	8.417988	9.898077	10.161049
H	9.315604	11.449794	10.105954
C	9.988450	9.958296	8.593340
C	10.303368	8.533348	9.047045
H	9.386478	7.923819	9.068480
H	10.739679	8.547947	10.059102
H	11.011315	8.066140	8.350966
C	11.254907	10.815008	8.487450
H	11.922205	10.391609	7.725142
H	11.776373	10.842960	9.457704
H	11.000248	11.846480	8.195731
C	7.663311	7.987748	3.599500

H	7.152579	8.308428	2.671986
C	6.609905	7.449391	4.588948
H	7.115087	7.213456	5.544212
H	5.853448	8.215339	4.816371
C	5.917874	6.189787	4.049069
H	5.191521	5.817586	4.791451
H	5.333482	6.461680	3.150112
C	6.923734	5.093596	3.681802
H	7.427922	4.743312	4.601177
H	6.401992	4.219877	3.255807
C	7.979721	5.625927	2.707626
H	7.496726	5.871882	1.742934
H	8.732416	4.849483	2.490956
C	8.675973	6.872754	3.264363
H	9.218352	6.615549	4.190198
H	9.453798	7.221824	2.574446
C	9.064311	10.554788	2.853514
H	8.136774	10.962856	2.414573
C	9.825966	9.805512	1.749720
H	9.188428	9.020929	1.311790
H	10.697712	9.292958	2.182969
C	10.288523	10.763433	0.642220
H	10.858967	10.201223	-0.116405
H	9.405442	11.182435	0.123529
C	11.131633	11.913686	1.203385
H	12.059719	11.501255	1.639562
H	11.437996	12.599219	0.395193
C	10.363042	12.672923	2.290584
H	9.481564	13.165391	1.838803
H	10.986448	13.476509	2.717545
C	9.901997	11.725940	3.404764
H	10.782997	11.303978	3.920096
H	9.328399	12.279968	4.167238
C	7.195967	10.506004	5.002529
C	6.121770	10.857319	4.167212
H	6.110070	10.497468	3.135677
C	5.061697	11.644929	4.613093
H	4.243518	11.897801	3.934050
C	5.058059	12.104446	5.932567
H	4.238961	12.726669	6.300921
C	6.101946	11.760873	6.785330
H	6.092612	12.111011	7.816226
C	7.180732	10.959221	6.345933

distorted 4-OMe-b aryne
ωB97X-D/def2-TZVP/SMD(THF) SCF energy (au): -345.423585

Cartesian coordinates

ATOM	X	Y	Z
C	-1.117192	0.000000	-0.706268
C	-2.436170	0.109067	-0.980845
C	-3.419236	0.197096	0.010042
C	-2.959783	0.162045	1.335223
H	-3.672395	0.225919	2.164430
C	-1.589764	0.042481	1.636044
H	-1.285991	0.016341	2.683822
C	-0.634389	-0.043385	0.601326
O	0.716965	-0.162404	0.821999
C	1.199647	-0.199525	2.141712
H	0.953656	0.722705	2.701217
H	0.801823	-1.064463	2.705188
H	2.293449	-0.292843	2.076936
H	-4.491307	0.286965	-0.199861

distorted 4-OMe-a aryne

ωB97X-D/def2-TZVP/SMD(THF) SCF energy (au): -345.424334

Cartesian coordinates

ATOM	X	Y	Z
C	0.217009	-0.000000	-2.650837
C	-0.671552	0.456066	-1.741646
C	-0.426926	0.332388	-0.375259
C	-1.133032	0.694761	1.886226
H	-1.036240	-0.361256	2.203062
H	-2.000488	1.137763	2.396833
H	-0.220907	1.235614	2.203099
O	-1.366164	0.814169	0.505710
C	0.780746	-0.284068	0.015629
H	1.035309	-0.411099	1.069360
C	1.685511	-0.753876	-0.956666
H	2.611480	-1.232207	-0.620560
C	1.425387	-0.624182	-2.329371
H	2.137986	-1.004074	-3.068377

Ni Complex

ωB97X-D/def2-TZVP/SMD(THF) SCF energy (au): -2876.740413

Cartesian coordinates

ATOM	X	Y	Z
Ni	10.091830	8.420242	11.967286
P	11.263875	9.947547	11.346480
N	10.022156	8.160976	13.769427
C	12.930374	9.655774	10.507232
H	13.540850	10.574540	10.562924
C	12.753874	9.273995	9.027606

H	12.324040	10.116345	8.463708
H	12.020558	8.448347	8.968786
C	14.075337	8.848349	8.371782
H	13.892740	8.546496	7.326253
H	14.755753	9.719363	8.331078
C	14.758060	7.717679	9.147726
H	14.121477	6.814272	9.107161
H	15.717607	7.448370	8.674530
C	14.969839	8.116224	10.611918
H	15.682611	8.960604	10.659154
H	15.429803	7.287563	11.176923
C	13.651380	8.530464	11.275065
H	13.830448	8.838418	12.317528
H	12.967417	7.662619	11.323943
C	10.558038	11.519779	10.566695
H	11.380364	12.194086	10.263226
C	9.736479	11.125483	9.321583
H	10.372918	10.639581	8.567733
H	9.008281	10.356471	9.638452
C	9.005818	12.319065	8.693821
H	9.749777	13.039422	8.305049
H	8.415951	11.980568	7.824898
C	8.106122	13.024871	9.712761
H	7.615387	13.901958	9.257590
H	7.298365	12.334791	10.019656
C	8.909679	13.437653	10.949777
H	8.251184	13.906001	11.701247
H	9.649459	14.207439	10.660387
C	9.647039	12.247102	11.575797
H	8.912724	11.513883	11.960636
H	10.227041	12.586723	12.446188
C	11.896581	10.605161	13.006852
C	12.762782	11.711292	12.953035
H	13.001267	12.145499	11.979489
C	13.329252	12.292462	14.086879
H	13.994533	13.154161	13.988526
C	13.033703	11.756727	15.343244
H	13.467598	12.188251	16.248784
C	12.178283	10.666536	15.440025
H	11.948824	10.253414	16.421219
C	11.585907	10.065893	14.298965
C	10.684960	8.935750	14.585854
C	9.180896	7.233997	14.567229
C	9.349702	5.816195	14.025731
H	8.734313	5.095333	14.588052
H	10.403622	5.503442	14.077054

H	9.044835	5.796936	12.967219
C	7.726926	7.706361	14.447562
H	7.041724	7.023560	14.975444
H	7.449447	7.747373	13.382749
H	7.613175	8.718329	14.866319
O	10.485809	8.655198	15.911735
C	9.755163	7.427094	15.988223
H	10.450876	6.616105	16.268043
H	8.985960	7.523985	16.768294

4-H aryne

ωB97X-D/def2-TZVP/SMD(THF) SCF energy (au): -230.908707

Cartesian coordinates

ATOM	X	Y	Z
C	-0.345032	-0.017070	-0.604756
C	-1.276330	0.994501	-0.417896
C	-1.688683	1.448429	0.676137
C	-1.309529	1.044906	1.948082
H	-1.667737	1.439023	2.901424
C	-0.351467	0.005800	1.871200
H	0.042085	-0.414723	2.801661
C	0.112882	-0.505263	0.642376
H	0.854652	-1.309712	0.650533
H	0.016711	-0.415500	-1.554838

4-CN aryne

ωB97X-D/def2-TZVP/SMD(THF) SCF energy (au): -323.148296

Cartesian coordinates

ATOM	X	Y	Z
C	-0.339679	-0.029418	-0.607245
C	-1.278789	0.991734	-0.451033
C	-1.667467	1.424175	0.658249
C	-1.304822	1.041784	1.936233
H	-1.668436	1.445215	2.882755
C	-0.346284	-0.000053	1.868563
H	0.043791	-0.415236	2.802317
C	0.120727	-0.518065	0.649269
H	0.860960	-1.321992	0.654941
C	0.142108	-0.563847	-1.847863
N	0.539998	-1.004863	-2.845133

4-F aryne

ωB97X-D/def2-TZVP/SMD(THF) SCF energy (au): -330.164776

Cartesian coordinates

ATOM	X	Y	Z
C	-0.373260	0.014294	-0.556134

C	-1.311391	1.032152	-0.451419
C	-1.655398	1.412008	0.696265
C	-1.312384	1.048089	1.979016
H	-1.679688	1.452177	2.922020
C	-0.352953	0.007746	1.897194
H	0.041976	-0.414600	2.826181
C	0.105590	-0.497463	0.667534
H	0.845863	-1.300293	0.638838
F	0.079199	-0.483719	-1.705571

4-Me aryne

ωB97X-D/def2-TZVP/SMD(THF) SCF energy (au): -270.231004

Cartesian coordinates

ATOM	X	Y	Z
C	-0.344063	-0.041680	-0.578341
C	-1.309235	0.945795	-0.383576
C	-1.730611	1.409133	0.701825
C	-1.332654	1.045031	1.981542
H	-1.697029	1.445871	2.929397
C	-0.343780	0.037173	1.911636
H	0.072570	-0.356408	2.844217
C	0.126646	-0.480021	0.687296
H	0.894526	-1.260199	0.707813
C	0.147401	-0.583196	-1.889452
H	0.915178	-1.356809	-1.742564
H	-0.681228	-1.024532	-2.467325
H	0.580427	0.219841	-2.508271

4-OMe aryne

ωB97X-D/def2-TZVP/SMD(THF) SCF energy (au): -345.449440

Cartesian coordinates

ATOM	X	Y	Z
C	-0.470828	-1.725828	-1.176517
C	-1.818287	-1.432894	-1.425648
C	-2.629433	-1.367906	-0.467893
C	-2.523588	-1.526302	0.895711
H	-3.300714	-1.448325	1.655343
C	-1.166589	-1.825866	1.189764
H	-0.884523	-1.987299	2.235082
C	-0.178207	-1.922592	0.199436
H	0.854108	-2.155198	0.470750
O	0.517435	-1.831253	-2.080386
C	0.166796	-1.624696	-3.436778
H	-0.587475	-2.359216	-3.770308
H	1.085819	-1.747722	-4.025230
H	-0.240336	-0.610201	-3.593875

MeOTf

B3LYP-D3/Def2-SVP SCF energy (au):	-1000.741073
B3LYP-D3/Def2-SVP enthalpy (au):	-1000.664851
B3LYP-D3/Def2-SVP free energy (au):	-1000.709316
ωB97XD/Def2-TZVP/SMD(THF) SCF energy (au):	-1001.474457
ωB97XD/Def2-TZVP/SMD(THF) enthalpy (au):	-1001.398235
ωB97XD/Def2-TZVP/SMD(THF) free energy (au):	-1001.442700

Cartesian coordinates

ATOM	X	Y	Z
S	2.496620	12.103452	-0.005663
O	3.949018	12.074497	-0.057436
O	1.751934	13.350989	-0.054495
O	1.959807	11.177732	1.221874
C	2.230894	11.677212	2.547011
H	1.741741	12.651983	2.698089
H	1.806911	10.934219	3.233224
H	3.316246	11.764257	2.710789
C	1.834423	10.966002	-1.347455
F	2.151897	11.510678	-2.515021
F	0.518665	10.861614	-1.245353
F	2.392547	9.769966	-1.245054

m-Int-Me

B3LYP-D3/Def2-SVP SCF energy (au):	-4146.760864
B3LYP-D3/Def2-SVP enthalpy (au):	-4146.005773
B3LYP-D3/Def2-SVP free energy (au):	-4146.122042
ωB97XD/Def2-TZVP/SMD(THF) SCF energy (au):	-4148.716208
ωB97XD/Def2-TZVP/SMD(THF) enthalpy (au):	-4147.961117
ωB97XD/Def2-TZVP/SMD(THF) free energy (au):	-4148.077386

Cartesian coordinates

ATOM	X	Y	Z
P	2.935929	11.284598	8.608691
C	0.997722	10.124278	6.597159
C	-0.263725	9.607017	6.975686
Ni	2.697629	9.574583	7.267752
C	-1.426259	10.136845	6.389267
H	-2.400893	9.750564	6.703771
C	-1.361254	11.131869	5.413123
H	-2.277998	11.541369	4.980362
C	1.454015	11.946949	9.531472
H	1.856264	12.644199	10.284733
O	6.422934	8.272714	8.399867
N	4.631723	9.132768	7.396750

C	-0.115356	11.577857	4.976959
C	0.700837	10.829357	10.270489
H	0.342044	10.104229	9.533057
H	1.379227	10.278051	10.940121
C	-0.492834	11.384516	11.056684
H	-0.127067	12.035246	11.873645
H	-1.034712	10.553362	11.537426
C	-1.431631	12.188892	10.150627
H	-2.274168	12.597608	10.733027
H	-1.864862	11.515763	9.389249
C	-0.673356	13.316011	9.441163
H	-1.347781	13.872967	8.770106
H	-0.306505	14.040338	10.193164
C	0.509493	12.769237	8.630137
H	1.050449	13.602742	8.154110
H	0.126609	12.137457	7.818708
C	3.787131	12.839628	7.992750
H	3.112738	13.183328	7.188632
C	3.932194	13.971269	9.026217
H	4.555725	13.617284	9.865741
H	2.955696	14.252120	9.448118
C	4.587741	15.211895	8.399346
H	3.911870	15.623117	7.627247
H	4.701418	15.997190	9.165143
C	5.941153	14.881162	7.761443
H	6.648095	14.568496	8.552084
H	6.374250	15.778846	7.290048
C	5.799734	13.749906	6.738071
H	6.782251	13.481023	6.315468
H	5.178283	14.096539	5.892016
C	5.146807	12.510350	7.359731
H	5.816192	12.093569	8.133801
H	5.023264	11.728393	6.604081
C	4.068564	10.682602	9.971499
C	3.962415	11.247578	11.252388
H	3.233317	12.033846	11.441991
C	4.758841	10.824414	12.320223
H	4.633269	11.286727	13.302312
C	5.697407	9.812141	12.129030
H	6.317674	9.463224	12.957550
C	5.836889	9.243311	10.865398
H	6.564712	8.450008	10.697898
C	5.044241	9.665869	9.783865
C	5.315707	9.023701	8.482854
C	6.326881	7.578930	7.123709
H	5.806983	6.623321	7.297067

H	7.343979	7.423522	6.743392
C	5.468212	8.538176	6.288323
C	4.658663	7.867519	5.184003
H	3.909137	8.571446	4.791427
H	4.139819	6.973936	5.537599
H	5.333151	7.590033	4.359086
C	6.330730	9.661359	5.684481
H	7.031425	9.231754	4.952031
H	6.913549	10.188213	6.454444
H	5.699613	10.391290	5.157916
C	1.065037	11.074103	5.548053
S	2.419735	6.296321	7.643531
O	1.150979	5.576007	7.728494
O	2.282092	7.647120	6.978429
O	3.613703	5.554572	7.200973
C	2.835848	6.687833	9.459110
F	2.311435	7.861077	9.863587
F	4.165854	6.766844	9.632275
F	2.371066	5.730531	10.249987
H	-0.049412	12.327036	4.181169
C	-0.422981	8.457120	7.940788
H	-1.362992	8.533729	8.510050
H	-0.426863	7.499286	7.398804
H	0.411925	8.387806	8.642635
C	2.384652	11.529840	4.956176
H	3.233867	11.329057	5.623010
H	2.590666	10.986285	4.017813
H	2.383493	12.605429	4.714328

o-Int-Me

B3LYP-D3/Def2-SVP SCF energy (au): -4146.763101
B3LYP-D3/Def2-SVP enthalpy (au): -4146.008347
B3LYP-D3/Def2-SVP free energy (au): -4146.125313
ωB97XD/Def2-TZVP/SMD(THF) SCF energy (au): -4148.721982
ωB97XD/Def2-TZVP/SMD(THF) enthalpy (au): -4147.967228
ωB97XD/Def2-TZVP/SMD(THF) free energy (au): -4148.084194

Cartesian coordinates

ATOM	X	Y	Z
P	2.979109	11.296658	8.755330
C	1.312453	10.529998	6.388325
C	-0.054705	10.230740	6.204945
Ni	2.632020	9.644972	7.425002
C	-0.726349	10.702341	5.048106
C	-0.035088	11.470064	4.100505
H	-0.563449	11.829311	3.212572

C	1.517137	11.637982	9.858238
H	1.860589	12.329053	10.646220
O	6.528826	8.431392	8.282073
N	4.446293	8.883917	7.619019
C	1.311425	11.780224	4.282010
C	1.070969	10.320707	10.523790
H	0.846529	9.571382	9.749681
H	1.896632	9.895087	11.114374
C	-0.159055	10.523206	11.415050
H	0.119426	11.141690	12.289509
H	-0.483936	9.545736	11.806112
C	-1.302796	11.207092	10.658564
H	-2.162302	11.372776	11.329269
H	-1.651742	10.537572	9.854049
C	-0.842242	12.534049	10.044131
H	-1.663797	13.006501	9.480309
H	-0.571651	13.238130	10.853734
C	0.363563	12.333768	9.115270
H	0.693445	13.304079	8.708826
H	0.063394	11.724351	8.253423
C	3.545261	12.927332	8.057622
H	2.761811	13.145091	7.310625
C	3.630953	14.113525	9.031553
H	4.394940	13.903640	9.799648
H	2.675468	14.261666	9.558832
C	4.018882	15.398259	8.282539
H	3.212021	15.658575	7.573513
H	4.093944	16.237595	8.993844
C	5.335001	15.227695	7.513867
H	6.157980	15.076604	8.236869
H	5.574599	16.147190	6.954638
C	5.275595	14.024372	6.565014
H	6.245630	13.881788	6.060263
H	4.532531	14.219227	5.770363
C	4.880138	12.743915	7.310706
H	5.668192	12.486359	8.041635
H	4.815435	11.894298	6.614318
C	4.308800	10.846511	9.984577
C	4.369071	11.542532	11.203475
H	3.641101	12.323691	11.419063
C	5.334554	11.260050	12.172439
H	5.339019	11.820897	13.110207
C	6.281162	10.262672	11.939579
H	7.038142	10.027264	12.690985
C	6.251570	9.561991	10.736784
H	6.985120	8.780285	10.540541

C	5.278089	9.835997	9.757838
C	5.353304	9.044213	8.516222
C	6.323355	7.581522	7.132879
H	6.138926	6.557422	7.493734
H	7.230467	7.608651	6.514902
C	5.081583	8.183833	6.449560
C	4.204393	7.121885	5.793276
H	3.323982	7.579821	5.324737
H	3.839608	6.383657	6.514726
H	4.792649	6.610156	5.014498
C	5.467176	9.267936	5.429618
H	5.952733	8.811820	4.552955
H	6.159157	10.001301	5.872337
H	4.565277	9.797608	5.089167
C	1.971776	11.318766	5.424059
S	1.383460	6.762123	8.295334
O	0.291472	7.195879	9.179000
O	1.818267	7.809941	7.291635
O	1.327930	5.428748	7.697376
C	2.824823	6.638873	9.505007
F	3.054212	7.817350	10.114413
F	3.970751	6.270378	8.900095
F	2.549272	5.737675	10.441433
H	3.024673	11.585113	5.549036
H	1.846503	12.383030	3.542443
C	-0.826955	9.406024	7.199579
H	-1.768495	9.899324	7.492774
H	-1.098193	8.423681	6.779307
H	-0.246276	9.201019	8.099078
C	-2.182052	10.368463	4.827304
H	-2.544382	10.761689	3.865913
H	-2.352002	9.278981	4.835213
H	-2.820404	10.786429	5.624619

m-Int-F

B3LYP-D3/Def2-SVP SCF energy (au): -4206.638797
B3LYP-D3/Def2-SVP enthalpy (au): -4205.920230
B3LYP-D3/Def2-SVP free energy (au): -4206.035300
ωB97XD/Def2-TZVP/SMD(THF) SCF energy (au): -4208.658373
ωB97XD/Def2-TZVP/SMD(THF) enthalpy (au): -4207.939806
ωB97XD/Def2-TZVP/SMD(THF) free energy (au): -4208.054876

Cartesian coordinates

ATOM	X	Y	Z
P	3.005429	11.298684	8.705610

C	1.254766	10.450594	6.366810
C	-0.134274	10.178591	6.270993
Ni	2.639381	9.603941	7.399941
C	-0.902313	10.787431	5.260917
H	-1.972873	10.568927	5.212997
C	-0.337946	11.652339	4.322360
H	-0.957800	12.113509	3.549468
C	1.521800	11.654422	9.774475
H	1.859072	12.349190	10.561752
O	6.531869	8.421675	8.310740
N	4.461817	8.883962	7.618472
C	1.029655	11.916329	4.376496
C	1.057807	10.345676	10.445386
H	0.842941	9.588876	9.675714
H	1.871381	9.920092	11.052700
C	-0.183808	10.564217	11.317406
H	0.090846	11.180233	12.194683
H	-0.525144	9.591281	11.705805
C	-1.310608	11.262624	10.547773
H	-2.172475	11.442947	11.211606
H	-1.665688	10.597376	9.742073
C	-0.825643	12.580563	9.932592
H	-1.635605	13.062421	9.360303
H	-0.550151	13.283904	10.741193
C	0.382818	12.355015	9.013632
H	0.727727	13.314675	8.594685
H	0.077437	11.738318	8.159189
C	3.620128	12.915476	8.020625
H	2.904855	13.114467	7.205604
C	3.621955	14.120050	8.976316
H	4.331336	13.935012	9.801174
H	2.630504	14.271318	9.430794
C	4.049628	15.392046	8.226213
H	3.291037	15.625149	7.456925
H	4.064656	16.248325	8.921054
C	5.417950	15.221923	7.554420
H	6.190950	15.106035	8.336772
H	5.680904	16.130400	6.987493
C	5.444247	13.991349	6.639666
H	6.450962	13.850324	6.212089
H	4.758551	14.144494	5.787127
C	5.013247	12.726286	7.390870
H	5.743098	12.508451	8.191807
H	5.005014	11.863231	6.713354
C	4.294291	10.838022	9.973315
C	4.331661	11.538113	11.190659

H	3.604932	12.324958	11.388808
C	5.274840	11.252083	12.180480
H	5.262761	11.815869	13.116401
C	6.220685	10.248547	11.971342
H	6.959736	10.011511	12.739850
C	6.213635	9.544956	10.769917
H	6.946791	8.758852	10.590033
C	5.262960	9.823729	9.770921
C	5.356496	9.036162	8.529725
C	6.343215	7.580454	7.151321
H	6.148594	6.555055	7.502096
H	7.261087	7.609261	6.549737
C	5.119130	8.197083	6.449866
C	4.252482	7.147428	5.761599
H	3.382235	7.615026	5.283204
H	3.873919	6.398362	6.464644
H	4.853860	6.648091	4.984990
C	5.536009	9.293018	5.455411
H	6.024366	8.841469	4.577827
H	6.240402	9.999805	5.921372
H	4.656295	9.858618	5.119333
C	1.767495	11.305648	5.385278
S	1.444742	6.726383	8.301927
O	0.366187	7.174061	9.193438
O	1.876161	7.762492	7.280477
O	1.374214	5.391786	7.709899
C	2.894457	6.604901	9.498450
F	3.112141	7.780268	10.116929
F	4.039253	6.254426	8.881862
F	2.633425	5.691842	10.426687
H	1.534109	12.570040	3.662027
C	-0.826936	9.222509	7.204596
H	-1.900426	9.450298	7.291441
H	-0.728125	8.190412	6.835944
H	-0.384963	9.215855	8.203541
F	3.112931	11.587694	5.381519

o-Int-F

B3LYP-D3/Def2-SVP SCF energy (au): -4206.634167
B3LYP-D3/Def2-SVP enthalpy (au): -4205.915703
B3LYP-D3/Def2-SVP free energy (au): -4206.031665
ωB97XD/Def2-TZVP/SMD(THF) SCF energy (au): -4208.659645
ωB97XD/Def2-TZVP/SMD(THF) enthalpy (au): -4207.941181
ωB97XD/Def2-TZVP/SMD(THF) free energy (au): -4208.057143

Cartesian coordinates

ATOM	X	Y	Z
P	2.988441	11.295177	8.749691
C	1.315791	10.510206	6.392215
C	-0.053479	10.204685	6.234416
Ni	2.644238	9.632004	7.424332
C	-0.711224	10.738105	5.116000
C	-0.097008	11.540515	4.159258
H	-0.685246	11.915096	3.319634
C	1.515590	11.635907	9.838219
H	1.853813	12.319571	10.634814
O	6.543460	8.440540	8.291579
N	4.459599	8.880635	7.625895
C	1.256352	11.833978	4.320749
C	1.057841	10.314682	10.488128
H	0.845791	9.571074	9.705151
H	1.874202	9.884077	11.088020
C	-0.186350	10.509623	11.361431
H	0.079607	11.115828	12.248237
H	-0.518997	9.527652	11.734129
C	-1.316783	11.206629	10.596580
H	-2.184919	11.367104	11.257196
H	-1.658099	10.550151	9.778055
C	-0.842324	12.539255	10.005900
H	-1.654275	13.023102	9.438163
H	-0.577023	13.231433	10.827430
C	0.372769	12.342744	9.088604
H	0.710813	13.315121	8.693918
H	0.077290	11.741064	8.219389
C	3.557845	12.923312	8.050668
H	2.770984	13.146755	7.308955
C	3.653198	14.105600	9.028613
H	4.419272	13.889006	9.792698
H	2.700521	14.255856	9.560399
C	4.044308	15.391221	8.282947
H	3.235957	15.658519	7.578239
H	4.126385	16.227220	8.997302
C	5.356528	15.216791	7.508562
H	6.181450	15.057922	8.227634
H	5.599128	16.137288	6.952384
C	5.286594	14.018154	6.554578
H	6.253497	13.872602	6.044776
H	4.541115	14.220927	5.764136
C	4.887449	12.736082	7.295660
H	5.677949	12.469607	8.020781
H	4.813612	11.890810	6.594938
C	4.309046	10.845311	9.987359

C	4.359197	11.539622	11.207596
H	3.627963	12.318682	11.419697
C	5.319610	11.258232	12.181998
H	5.316467	11.817399	13.120742
C	6.270977	10.264461	11.953265
H	7.024136	10.030449	12.708902
C	6.251034	9.565415	10.749247
H	6.988075	8.786133	10.556336
C	5.282429	9.837817	9.765259
C	5.364957	9.046625	8.523978
C	6.344870	7.589404	7.141826
H	6.167117	6.564122	7.502576
H	7.252445	7.622731	6.524882
C	5.099729	8.183186	6.457013
C	4.230659	7.114224	5.801121
H	3.349005	7.564986	5.328113
H	3.868115	6.375724	6.523378
H	4.824617	6.603841	5.025838
C	5.478421	9.268742	5.436069
H	5.965150	8.814920	4.558924
H	6.166780	10.006266	5.877379
H	4.573052	9.793231	5.096358
C	1.943832	11.331090	5.431182
S	1.399577	6.758168	8.294056
O	0.308741	7.196959	9.175600
O	1.835394	7.802433	7.284725
O	1.344021	5.424162	7.698663
C	2.839793	6.637938	9.505707
F	3.066197	7.817449	10.114410
F	3.987461	6.270401	8.903761
F	2.562784	5.737519	10.441833
H	2.999316	11.593877	5.536571
H	1.774128	12.460383	3.589168
F	-2.020397	10.454987	4.954253
C	-0.843322	9.332221	7.169748
H	-1.853684	9.737612	7.331209
H	-0.959315	8.318941	6.753829
H	-0.340656	9.209294	8.130153

m-TS1-Me-a

B3LYP-D3/Def2-SVP SCF energy (au):	-4146.625794
B3LYP-D3/Def2-SVP enthalpy (au):	-4145.875256
B3LYP-D3/Def2-SVP free energy (au):	-4145.998686
ωB97XD/Def2-TZVP/SMD(THF) SCF energy (au):	-4148.589121
ωB97XD/Def2-TZVP/SMD(THF) enthalpy (au):	-4147.838583
ωB97XD/Def2-TZVP/SMD(THF) free energy (au):	-4147.962013

Cartesian coordinates

ATOM	X	Y	Z
P	2.801696	11.385289	8.913886
C	1.169164	10.842046	6.091828
C	0.225231	11.765992	5.605610
H	-0.493175	12.281160	6.250816
Ni	2.601651	10.146819	7.114071
C	0.190265	11.937801	4.220507
H	-0.533433	12.623897	3.771139
C	1.068732	11.221835	3.381523
H	1.014258	11.393516	2.301080
C	1.394341	11.820902	10.047063
H	1.753858	12.587946	10.755031
O	5.690708	7.774780	8.660251
N	4.083834	8.900950	7.592301
C	2.026104	10.314762	3.878307
C	0.940666	10.583598	10.842824
H	0.643236	9.787316	10.145808
H	1.776058	10.187316	11.442768
C	-0.252242	10.906487	11.752027
H	0.073699	11.597824	12.552936
H	-0.590686	9.978571	12.238585
C	-1.411183	11.535706	10.969888
H	-2.230020	11.809206	11.656229
H	-1.812212	10.777538	10.278268
C	-0.951175	12.765305	10.178950
H	-1.783664	13.178041	9.584433
H	-0.639127	13.564758	10.877433
C	0.223626	12.426459	9.252016
H	0.545775	13.323571	8.697716
H	-0.102621	11.693799	8.497648
C	3.422481	13.008006	8.201379
H	2.632580	13.236519	7.462937
C	3.559555	14.200884	9.158763
H	4.328470	13.975925	9.917795
H	2.616771	14.384054	9.698383
C	3.976027	15.468562	8.396148
H	3.168343	15.748744	7.695409
H	4.085548	16.310047	9.100589
C	5.275038	15.251402	7.611162
H	6.101867	15.073413	8.323757
H	5.539185	16.160774	7.046097
C	5.157539	14.048790	6.667643
H	6.112653	13.872043	6.145322
H	4.407968	14.268243	5.885633

C	4.731936	12.781215	7.419110
H	5.528376	12.487191	8.127053
H	4.609267	11.942678	6.714571
C	4.142245	10.850150	10.065362
C	4.346551	11.567534	11.256599
H	3.693888	12.407455	11.494894
C	5.357973	11.235193	12.157559
H	5.487059	11.819859	13.071624
C	6.194767	10.152521	11.882780
H	6.992547	9.876863	12.576203
C	5.999052	9.408985	10.723089
H	6.638072	8.551200	10.520369
C	4.980202	9.734757	9.800513
C	4.853526	8.833652	8.630169
C	5.299731	6.887431	7.601377
H	4.717040	6.061550	8.042739
H	6.205910	6.486408	7.127384
C	4.447438	7.773381	6.672076
C	3.213438	7.031699	6.164243
H	2.605670	7.693956	5.533724
H	2.594034	6.677933	7.000257
H	3.521806	6.159924	5.565498
C	5.274559	8.368790	5.527464
H	5.524236	7.596052	4.783547
H	6.210217	8.806090	5.910567
H	4.693092	9.161838	5.041576
C	2.069105	10.205420	5.277343
S	-1.160651	7.243918	9.033436
O	-2.027517	6.137766	9.427916
O	-1.236056	8.509270	9.789150
O	-1.089032	7.455328	7.535548
C	0.004362	9.141761	6.935584
H	-0.420351	9.716138	7.752180
H	0.860859	8.507752	7.111686
H	-0.504227	9.076399	5.980644
C	0.578228	6.622742	9.419993
F	0.694613	6.280164	10.699568
F	1.499380	7.587423	9.167901
F	0.901771	5.569253	8.661426
C	2.961034	9.584014	2.945757
H	3.979210	10.007476	2.990631
H	3.045016	8.520287	3.217116
H	2.623340	9.643789	1.899863

o-TS1-Me-a

B3LYP-D3/Def2-SVP SCF energy (au): -4146.641885

B3LYP-D3/Def2-SVP enthalpy (au): -4145.890357
B3LYP-D3/Def2-SVP free energy (au): -4146.010960
ωB97XD/Def2-TZVP/SMD(THF) SCF energy (au): -4148.600685
ωB97XD/Def2-TZVP/SMD(THF) enthalpy (au): -4147.849157
ωB97XD/Def2-TZVP/SMD(THF) free energy (au): -4147.969760

Cartesian coordinates

ATOM	X	Y	Z
P	2.832956	11.437902	8.607370
C	0.766230	11.004361	6.119359
C	-0.114690	11.983940	5.658094
H	-0.156597	13.002442	6.054880
Ni	2.304901	10.298912	6.831370
C	-0.938632	11.615033	4.584275
H	-1.631452	12.346140	4.156298
C	-0.874642	10.326828	4.030287
H	-1.537889	10.076014	3.196164
C	1.378828	11.970283	9.647925
H	1.751970	12.271479	10.640263
O	5.882735	8.258196	7.566815
N	3.980155	9.194118	6.860612
C	0.015545	9.348549	4.514858
C	0.421686	10.774134	9.822564
H	0.119559	10.429961	8.818687
H	0.944148	9.929963	10.299091
C	-0.824010	11.156960	10.629282
H	-0.528029	11.407272	11.665202
H	-1.501226	10.289774	10.698915
C	-1.544264	12.356554	10.003725
H	-2.425316	12.636188	10.605000
H	-1.918947	12.071474	9.003756
C	-0.594485	13.551146	9.860307
H	-1.110533	14.401649	9.384236
H	-0.286583	13.894598	10.865645
C	0.654083	13.185697	9.044840
H	1.329696	14.054820	8.992861
H	0.355336	12.947611	8.014763
C	3.909794	12.949058	8.409658
H	3.273358	13.628471	7.813680
C	4.317809	13.661253	9.708290
H	4.895095	12.961792	10.337459
H	3.428520	13.951923	10.291196
C	5.178817	14.899211	9.414013
H	4.568389	15.643870	8.870736
H	5.484761	15.375838	10.360301
C	6.408252	14.541395	8.570117

H	7.066839	13.873664	9.155938
H	6.997682	15.446227	8.347275
C	6.001519	13.833660	7.272038
H	6.893846	13.545155	6.691728
H	5.428653	14.535381	6.638438
C	5.139236	12.597008	7.552888
H	5.747750	11.841849	8.084045
H	4.807123	12.129005	6.614335
C	3.797922	10.342858	9.760115
C	3.744094	10.545619	11.148294
H	3.157995	11.368814	11.555476
C	4.415472	9.710619	12.044209
H	4.333249	9.893155	13.118521
C	5.176350	8.646135	11.563743
H	5.687922	7.971161	12.253092
C	5.271161	8.438659	10.190682
H	5.848701	7.601731	9.801097
C	4.590760	9.268794	9.282048
C	4.759256	8.932848	7.853501
C	5.765221	7.804161	6.207316
H	5.488200	6.739888	6.237395
H	6.736825	7.935856	5.711431
C	4.637151	8.680543	5.616928
C	3.682529	7.854873	4.763426
H	2.838801	8.479065	4.443042
H	3.309751	6.995606	5.337515
H	4.207167	7.479187	3.870837
C	5.172358	9.885983	4.833087
H	5.644819	9.558920	3.893564
H	5.916390	10.440639	5.424857
H	4.342019	10.567340	4.591378
C	0.830752	9.744850	5.595951
S	2.276521	5.670000	8.496485
O	3.431036	6.347428	7.872847
O	2.321712	4.225083	8.680203
O	0.937100	6.161028	7.945317
C	0.994309	7.871077	7.035110
H	-0.087341	7.940087	6.995793
H	1.518804	8.363828	7.845624
H	1.551069	7.448888	6.216045
C	2.216712	6.383132	10.231390
F	3.349179	6.119938	10.886972
F	1.196722	5.882831	10.919466
F	2.068086	7.722538	10.173716
C	0.011565	7.956767	3.929488
H	1.028532	7.603951	3.699555

H	-0.422890	7.224583	4.632393
H	-0.580012	7.910177	3.002866

m-TS1-Me-b

B3LYP-D3/Def2-SVP SCF energy (au): -4146.640385
B3LYP-D3/Def2-SVP enthalpy (au): -4145.888899
B3LYP-D3/Def2-SVP free energy (au): -4146.010646
ωB97XD/Def2-TZVP/SMD(THF) SCF energy (au): -4148.598926
ωB97XD/Def2-TZVP/SMD(THF) enthalpy (au): -4147.847440
ωB97XD/Def2-TZVP/SMD(THF) free energy (au): -4147.969187

Cartesian coordinates

ATOM	X	Y	Z
P	2.805975	11.423197	8.686900
C	0.801539	11.107025	6.103844
C	0.124369	12.161019	5.472934
Ni	2.227308	10.297424	6.931928
C	-0.670743	11.778997	4.372868
H	-1.206486	12.552890	3.812835
C	-0.759808	10.445905	3.944532
H	-1.388878	10.201678	3.083178
C	1.381881	11.854135	9.816874
H	1.795597	12.303666	10.735074
O	5.755082	8.140950	7.563934
N	3.831553	9.096173	6.948548
C	-0.034186	9.433851	4.589507
C	0.611893	10.573955	10.194343
H	0.273347	10.090136	9.260837
H	1.274466	9.854445	10.694103
C	-0.597181	10.881903	11.085773
H	-0.239686	11.267791	12.058749
H	-1.143098	9.948840	11.302459
C	-1.527179	11.916404	10.443346
H	-2.368982	12.150685	11.115982
H	-1.966323	11.486602	9.524525
C	-0.758715	13.191374	10.078941
H	-1.423660	13.914165	9.577112
H	-0.402168	13.682565	11.003560
C	0.437620	12.878261	9.170705
H	0.973546	13.807500	8.919670
H	0.070402	12.452769	8.225758
C	3.762681	12.999360	8.408613
H	3.045827	13.620013	7.840871
C	4.201646	13.775654	9.658461
H	4.870746	13.140056	10.263812

H	3.332687	14.019715	10.291543
C	4.948002	15.062457	9.273900
H	4.250340	15.743484	8.752616
H	5.278759	15.589979	10.184129
C	6.143236	14.766371	8.359849
H	6.885383	14.166585	8.918679
H	6.649256	15.703404	8.074018
C	5.707955	13.990499	7.110671
H	6.580524	13.747656	6.481640
H	5.046839	14.629375	6.496810
C	4.956239	12.705884	7.480197
H	5.648246	12.010868	7.991207
H	4.598452	12.186164	6.577316
C	3.890904	10.392862	9.791680
C	3.962016	10.679930	11.165151
H	3.427697	11.540603	11.567104
C	4.685340	9.879499	12.051595
H	4.702311	10.128645	13.115483
C	5.369273	8.760951	11.575569
H	5.919587	8.111983	12.260265
C	5.342843	8.470998	10.214575
H	5.867181	7.597245	9.829927
C	4.614681	9.270736	9.314258
C	4.672479	8.858491	7.896037
C	5.542594	7.648817	6.226831
H	5.251227	6.591583	6.306590
H	6.483005	7.750962	5.667834
C	4.393233	8.526901	5.680587
C	3.378518	7.688155	4.908700
H	2.536405	8.312414	4.583613
H	3.011304	6.867788	5.537851
H	3.863639	7.260347	4.016739
C	4.897897	9.702488	4.832395
H	5.330152	9.339763	3.886587
H	5.666520	10.276281	5.373498
H	4.060112	10.377798	4.599557
C	0.740882	9.803674	5.694445
S	2.074509	5.600766	8.520790
O	3.254166	6.177311	7.843836
O	2.025565	4.163662	8.754038
O	0.752217	6.161239	7.993366
C	0.829544	7.863412	7.081735
H	-0.090059	8.223542	7.531444
H	1.783655	8.111430	7.523552
H	0.804729	7.384911	6.112425
C	2.144386	6.386737	10.221101

F	3.302366	6.107003	10.822367
F	1.146590	5.970967	10.992998
F	2.052227	7.725920	10.097426
H	-0.104489	8.400126	4.230041
C	0.256544	13.615971	5.854383
H	0.191366	14.267757	4.969007
H	-0.539097	13.934504	6.550018
H	1.219371	13.806478	6.349919

o-TS1-Me-b

B3LYP-D3/Def2-SVP SCF energy (au):	-4146.634410
B3LYP-D3/Def2-SVP enthalpy (au):	-4145.883467
B3LYP-D3/Def2-SVP free energy (au):	-4146.004462
ωB97XD/Def2-TZVP/SMD(THF) SCF energy (au):	-4148.592229
ωB97XD/Def2-TZVP/SMD(THF) enthalpy (au):	-4147.841286
ωB97XD/Def2-TZVP/SMD(THF) free energy (au):	-4147.962281

Cartesian coordinates

ATOM	X	Y	Z
P	2.665329	11.486168	8.882068
C	1.484499	11.348523	5.801183
C	0.696401	12.321211	5.170688
Ni	2.439566	10.303608	7.015142
C	0.646097	12.181271	3.767309
H	0.066716	12.905838	3.185892
C	1.304326	11.140542	3.085531
H	1.220677	11.087675	1.995626
C	1.289118	11.836996	10.085196
H	1.639682	12.612531	10.788578
O	5.288763	7.668575	8.573738
N	3.767916	8.904714	7.504053
C	2.056686	10.174601	3.774638
C	0.946311	10.565348	10.883497
H	0.718578	9.737974	10.199412
H	1.815499	10.244046	11.478514
C	-0.268192	10.767582	11.796129
H	-0.014760	11.486269	12.598897
H	-0.511840	9.806974	12.274278
C	-1.477044	11.282340	11.009118
H	-2.335320	11.441971	11.682663
H	-1.778351	10.501681	10.289708
C	-1.136974	12.578785	10.266360
H	-2.001963	12.935238	9.682084
H	-0.905741	13.373809	11.000021
C	0.066270	12.392949	9.333089
H	0.319448	13.346782	8.843858

H	-0.202976	11.696263	8.522174
C	3.264931	13.140817	8.241735
H	2.449290	13.399005	7.544911
C	3.433772	14.298722	9.235573
H	4.245121	14.063924	9.945158
H	2.516765	14.446864	9.828395
C	3.791726	15.596687	8.494212
H	2.946841	15.883488	7.841477
H	3.921225	16.418353	9.218257
C	5.055439	15.425061	7.642708
H	5.917257	15.240326	8.310712
H	5.278470	16.355557	7.094711
C	4.914073	14.250283	6.667751
H	5.847448	14.105100	6.098926
H	4.127423	14.479937	5.926344
C	4.539259	12.952733	7.395168
H	5.369582	12.642277	8.055399
H	4.390927	12.141960	6.663054
C	4.021384	10.869359	9.971385
C	4.316184	11.581925	11.146764
H	3.736523	12.471566	11.392823
C	5.320617	11.178556	12.025690
H	5.522537	11.761312	12.927683
C	6.053471	10.024281	11.746247
H	6.842429	9.689243	12.423373
C	5.763470	9.285003	10.603848
H	6.321115	8.372876	10.399502
C	4.753315	9.684688	9.700645
C	4.535799	8.784989	8.539347
C	4.809295	6.802344	7.531895
H	4.122293	6.071229	7.988789
H	5.667684	6.285819	7.082528
C	4.078908	7.759954	6.575254
C	2.840301	7.099363	5.976269
H	2.315691	7.796137	5.311607
H	2.142415	6.749676	6.748792
H	3.153698	6.226207	5.382187
C	5.009268	8.301726	5.481602
H	5.267089	7.502958	4.768564
H	5.940841	8.693561	5.920023
H	4.508672	9.116746	4.940947
C	2.120077	10.351865	5.156745
S	-0.552287	6.912445	9.378414
O	-1.302806	5.675637	9.570009
O	-0.855803	8.074697	10.228311
O	-0.374782	7.273137	7.912346

C	0.646307	8.997487	7.444862
H	-0.068904	9.658945	7.925177
H	1.458916	8.584676	8.021424
H	0.472591	8.701046	6.419076
C	1.210899	6.446664	9.884150
F	1.231820	5.899937	11.092518
F	2.019111	7.535793	9.907566
F	1.754349	5.582267	9.011821
H	2.548964	9.359649	3.233696
C	-0.054163	13.418707	5.879842
H	-0.563861	14.086482	5.169365
H	-0.815584	13.005037	6.560308
H	0.620244	14.036150	6.496223

o-TS2-Me-b

B3LYP-D3/Def2-SVP SCF energy (au): -4146.658020
B3LYP-D3/Def2-SVP enthalpy (au): -4145.906971
B3LYP-D3/Def2-SVP free energy (au): -4146.027208
ωB97XD/Def2-TZVP/SMD(THF) SCF energy (au): -4148.613372
ωB97XD/Def2-TZVP/SMD(THF) enthalpy (au): -4147.862323
ωB97XD/Def2-TZVP/SMD(THF) free energy (au): -4147.982560

Cartesian coordinates

ATOM	X	Y	Z
P	2.443304	11.321352	8.890437
C	1.228835	11.130481	5.707882
C	0.458765	12.123750	5.093933
Ni	2.134701	10.105660	7.004234
C	0.612253	12.121391	3.690743
H	0.081999	12.880859	3.107977
C	1.406740	11.179288	3.012530
H	1.468390	11.229774	1.921432
C	1.104022	11.984747	10.012379
H	1.628819	12.632884	10.733826
O	5.550691	8.017094	8.211898
N	3.762536	9.010146	7.327874
C	2.111530	10.177341	3.700497
C	0.380424	10.884362	10.812131
H	-0.115271	10.187656	10.120390
H	1.104092	10.264294	11.357348
C	-0.648307	11.496994	11.769472
H	-0.124230	12.098049	12.536684
H	-1.172096	10.691378	12.309273
C	-1.650352	12.390499	11.030326
H	-2.366982	12.841351	11.736856
H	-2.243479	11.769046	10.334258

C	-0.927397	13.483210	10.235430
H	-1.648304	14.099484	9.672342
H	-0.413750	14.165885	10.937670
C	0.106271	12.889102	9.268624
H	0.628334	13.702704	8.741843
H	-0.417019	12.304645	8.499216
C	3.259811	12.854391	8.166806
H	2.493435	13.234412	7.467475
C	3.610043	13.961854	9.174644
H	4.338794	13.567287	9.903920
H	2.721941	14.270417	9.748340
C	4.213677	15.185719	8.468922
H	3.447856	15.635630	7.810355
H	4.474961	15.954457	9.215264
C	5.440280	14.807219	7.631877
H	6.242901	14.454300	8.305509
H	5.836874	15.692855	7.108185
C	5.100245	13.698259	6.630853
H	5.997097	13.398928	6.063489
H	4.374577	14.081491	5.890191
C	4.496793	12.472145	7.327100
H	5.254351	12.014458	7.988324
H	4.233826	11.710623	6.577978
C	3.711939	10.650535	10.033679
C	3.833666	11.188716	11.324170
H	3.197906	12.017699	11.632379
C	4.745270	10.680390	12.250285
H	4.807447	11.121482	13.248106
C	5.554994	9.602103	11.897784
H	6.255899	9.173668	12.617583
C	5.456884	9.059296	10.620270
H	6.067523	8.201648	10.348028
C	4.555384	9.575095	9.668723
C	4.553128	8.893883	8.355322
C	5.211662	7.175318	7.098831
H	4.691219	6.296507	7.506557
H	6.137484	6.890502	6.582174
C	4.280310	8.068246	6.263549
C	3.173274	7.230947	5.631169
H	2.525650	7.837484	4.988415
H	2.549062	6.773544	6.411598
H	3.628771	6.437868	5.015511
C	5.085954	8.889643	5.248804
H	5.451345	8.239866	4.437836
H	5.955756	9.358560	5.736524
H	4.468797	9.683718	4.812595

C	1.984899	10.219530	5.088171
S	1.946734	7.053710	9.429967
O	1.196989	6.677715	8.209948
O	1.726469	8.459864	9.866880
O	3.356909	6.604588	9.485327
C	0.198637	9.747223	7.268827
H	-0.423957	10.540111	7.689952
H	0.491685	8.993106	8.015133
H	-0.264253	9.295511	6.387279
C	1.110437	6.080671	10.789589
F	-0.182084	6.417872	10.873635
F	1.685449	6.335625	11.970403
F	1.189613	4.768309	10.556810
H	2.706093	9.436362	3.159314
C	-0.441032	13.106538	5.789622
H	-1.219559	12.591058	6.374607
H	0.122177	13.739867	6.491972
H	-0.944373	13.765445	5.067450

m-TS1-F-a

B3LYP-D3/Def2-SVP SCF energy (au): -4206.507752
B3LYP-D3/Def2-SVP enthalpy (au): -4205.793268
B3LYP-D3/Def2-SVP free energy (au): -4205.914182
ωB97XD/Def2-TZVP/SMD(THF) SCF energy (au): -4208.533104
ωB97XD/Def2-TZVP/SMD(THF) enthalpy (au): -4207.818620
ωB97XD/Def2-TZVP/SMD(THF) free energy (au): -4207.939534

Cartesian coordinates

ATOM	X	Y	Z
P	2.680339	11.501892	8.863069
C	1.442928	11.385979	5.890977
C	0.597433	12.365386	5.377163
H	0.088258	13.118967	5.983443
Ni	2.450143	10.317624	7.016783
C	0.425622	12.319349	3.981066
H	-0.213432	13.058058	3.488351
C	1.054670	11.335954	3.195028
H	0.913402	11.307894	2.112087
C	1.246676	11.936596	9.964360
H	1.569971	12.741348	10.647722
O	5.353510	7.723654	8.506814
N	3.808008	8.946094	7.462057
F	2.448053	9.419300	3.034770
C	1.870540	10.372185	3.801933

C	0.828133	10.712634	10.801272
H	0.638642	9.849342	10.151236
H	1.647532	10.412367	11.472114
C	-0.444091	10.973416	11.613781
H	-0.239881	11.729190	12.396247
H	-0.732147	10.039317	12.118652
C	-1.585170	11.461028	10.716928
H	-2.489746	11.657867	11.315704
H	-1.842373	10.651352	10.011526
C	-1.175227	12.720037	9.944873
H	-1.992043	13.061039	9.287046
H	-0.983997	13.542237	10.659716
C	0.085666	12.477659	9.104880
H	0.388774	13.407431	8.594296
H	-0.141392	11.752227	8.306930
C	3.394598	13.113196	8.230328
H	2.596615	13.451387	7.545275
C	3.663965	14.223720	9.256310
H	4.443578	13.890696	9.962479
H	2.760648	14.439435	9.849476
C	4.147933	15.504686	8.558681
H	3.339255	15.891874	7.912271
H	4.349474	16.285750	9.310572
C	5.395770	15.241113	7.707852
H	6.231216	14.952139	8.372309
H	5.710842	16.163182	7.191919
C	5.147552	14.118740	6.693716
H	6.064916	13.903653	6.121138
H	4.389564	14.447866	5.960012
C	4.648552	12.838403	7.375790
H	5.444598	12.428573	8.024104
H	4.430011	12.070738	6.615408
C	3.956780	10.825981	10.005257
C	4.179519	11.484256	11.226898
H	3.585921	12.363936	11.477026
C	5.129155	11.037327	12.144968
H	5.275139	11.575549	13.084550
C	5.879335	9.897133	11.854817
H	6.625666	9.528537	12.562091
C	5.662224	9.214266	10.661881
H	6.234079	8.313145	10.448378
C	4.706956	9.655559	9.719989
C	4.559664	8.809130	8.507127
C	4.933460	6.886588	7.415466
H	4.288639	6.094475	7.829790
H	5.824616	6.444275	6.951068

C	4.154220	7.844515	6.494943
C	2.929630	7.153929	5.897739
H	2.399044	7.832107	5.218486
H	2.240038	6.795575	6.674279
H	3.264193	6.283981	5.310407
C	5.039133	8.445133	5.395293
H	5.357580	7.656382	4.695802
H	5.936735	8.916805	5.825802
H	4.471722	9.198310	4.832089
C	2.066158	10.429083	5.167780
S	-0.445467	6.920435	9.463030
O	-1.133830	5.655884	9.705033
O	-0.790486	8.089849	10.288240
O	-0.304460	7.246745	7.988266
C	0.725739	9.006929	7.474232
H	-0.023564	9.651857	7.924048
H	1.511164	8.596706	8.088842
H	0.562978	8.652150	6.464959
C	1.347278	6.550153	9.956914
F	1.406348	5.963202	11.144765
F	2.084921	7.688179	10.021291
F	1.947719	5.755191	9.055074

m-TS2-F-a

B3LYP-D3/Def2-SVP SCF energy (au): -4206.530141
B3LYP-D3/Def2-SVP enthalpy (au): -4205.815508
B3LYP-D3/Def2-SVP free energy (au): -4205.934244
ωB97XD/Def2-TZVP/SMD(THF) SCF energy (au): -4208.551393
ωB97XD/Def2-TZVP/SMD(THF) enthalpy (au): -4207.836760
ωB97XD/Def2-TZVP/SMD(THF) free energy (au): -4207.955496

Cartesian coordinates

ATOM	X	Y	Z
P	2.451439	11.311542	8.851403
C	1.166379	11.090649	5.829547
C	0.372548	12.126431	5.354957
H	-0.250999	12.769738	5.978101
Ni	2.135988	10.050895	7.035800
C	0.446431	12.292032	3.959448
H	-0.118739	13.097081	3.481653
C	1.234258	11.445870	3.159057
H	1.283203	11.584313	2.076649
C	1.078793	12.019172	9.891099
H	1.576225	12.708672	10.594376
O	5.601680	8.076332	8.206829
N	3.785962	9.020816	7.332264

F	2.716148	9.609393	2.951103
C	1.975765	10.408382	3.743062
C	0.338593	10.953620	10.723205
H	-0.133354	10.220557	10.054072
H	1.049069	10.366105	11.320032
C	-0.723366	11.608082	11.614027
H	-0.228072	12.254300	12.363347
H	-1.259253	10.828891	12.179960
C	-1.707136	12.450909	10.794461
H	-2.449198	12.933131	11.452194
H	-2.273467	11.786474	10.116043
C	-0.969674	13.506736	9.962948
H	-1.679275	14.083565	9.346537
H	-0.484467	14.232012	10.642201
C	0.100090	12.874124	9.060998
H	0.633660	13.663239	8.507788
H	-0.392001	12.242204	8.306036
C	3.295750	12.807041	8.084028
H	2.543413	13.175979	7.364726
C	3.638778	13.932997	9.074898
H	4.343646	13.545263	9.831061
H	2.741053	14.267139	9.618613
C	4.275452	15.132662	8.356771
H	3.530358	15.579976	7.673364
H	4.532156	15.912126	9.093442
C	5.513116	14.719350	7.553587
H	6.296128	14.366896	8.250141
H	5.934185	15.588488	7.021597
C	5.174760	13.597305	6.567134
H	6.076726	13.273761	6.021749
H	4.468352	13.977402	5.806591
C	4.541695	12.392299	7.273947
H	5.281810	11.936520	7.955738
H	4.281588	11.622124	6.533745
C	3.688168	10.656373	10.027988
C	3.768102	11.191598	11.322215
H	3.110489	12.008798	11.617461
C	4.667129	10.690898	12.264876
H	4.700000	11.125228	13.266997
C	5.503074	9.628404	11.923644
H	6.193947	9.207809	12.657571
C	5.444130	9.089797	10.641577
H	6.072894	8.242275	10.378288
C	4.555457	9.596279	9.674605
C	4.581376	8.921677	8.358408
C	5.276685	7.231552	7.088756

H	4.770752	6.343565	7.495991
H	6.207277	6.963535	6.572402
C	4.327758	8.106988	6.254716
C	3.241779	7.255538	5.603823
H	2.634490	7.851510	4.912640
H	2.590656	6.814987	6.372270
H	3.719532	6.448287	5.024570
C	5.105146	8.954836	5.241128
H	5.527133	8.307284	4.456783
H	5.930729	9.493720	5.733054
H	4.442907	9.682150	4.757556
C	1.932694	10.250429	5.116510
S	2.036832	7.043280	9.481087
O	1.325014	6.658315	8.241793
O	1.741130	8.428724	9.944502
O	3.466808	6.662130	9.546404
C	0.217471	9.621577	7.348443
H	-0.481325	10.396871	7.670343
H	0.532326	8.958994	8.166991
H	-0.160768	9.070694	6.482835
C	1.233036	5.998263	10.806453
F	-0.076183	6.264744	10.877536
F	1.777463	6.255797	12.000849
F	1.385512	4.697919	10.545077

o-TS1-F-a

B3LYP-D3/Def2-SVP SCF energy (au): -4206.518455
B3LYP-D3/Def2-SVP enthalpy (au): -4205.803068
B3LYP-D3/Def2-SVP free energy (au): -4205.922182
ωB97XD/Def2-TZVP/SMD(THF) SCF energy (au): -4208.543176
ωB97XD/Def2-TZVP/SMD(THF) enthalpy (au): -4207.827789
ωB97XD/Def2-TZVP/SMD(THF) free energy (au): -4207.946903

Cartesian coordinates

ATOM	X	Y	Z
P	2.798460	11.433366	8.666260
C	0.591986	10.840990	6.262886
C	-0.437588	11.695202	5.867906
H	-0.650234	12.649979	6.355894
Ni	2.236244	10.312835	6.893727
C	-1.190538	11.303308	4.747208
H	-1.991153	11.950872	4.377510
C	-0.915901	10.105470	4.070250
H	-1.493470	9.797634	3.196080
C	1.387798	12.058954	9.708397

H	1.783563	12.368901	10.689841
O	5.927385	8.460612	7.460430
N	3.968670	9.319469	6.820835
F	0.356552	8.122736	3.878931
C	0.123135	9.292026	4.528378
C	0.364388	10.925357	9.919432
H	0.066668	10.544382	8.927704
H	0.832089	10.081632	10.448607
C	-0.879505	11.409735	10.672641
H	-0.598977	11.700411	11.702353
H	-1.599186	10.580067	10.767506
C	-1.523623	12.606899	9.966173
H	-2.410888	12.954380	10.520935
H	-1.876399	12.290211	8.967705
C	-0.514536	13.748733	9.805753
H	-0.972104	14.602007	9.277610
H	-0.224998	14.119643	10.806605
C	0.744491	13.296099	9.053030
H	1.464696	14.128813	9.012395
H	0.484861	13.051678	8.012145
C	3.952265	12.888453	8.452718
H	3.351358	13.599985	7.858102
C	4.386844	13.572689	9.758840
H	4.926438	12.841420	10.385744
H	3.509932	13.900833	10.339931
C	5.302844	14.773766	9.478990
H	4.726490	15.550439	8.943327
H	5.626994	15.225955	10.431111
C	6.516987	14.372954	8.632892
H	7.146579	13.672561	9.212287
H	7.143817	15.254333	8.418603
C	6.081171	13.694919	7.328612
H	6.960860	13.375207	6.745339
H	5.537225	14.424897	6.701482
C	5.170761	12.490875	7.600242
H	5.750004	11.713686	8.131014
H	4.826120	12.038519	6.658798
C	3.741278	10.283885	9.773254
C	3.650583	10.393907	11.169226
H	3.025844	11.167759	11.615055
C	4.335455	9.523134	12.019496
H	4.227620	9.627878	13.101818
C	5.142930	8.520280	11.484257
H	5.664445	7.819083	12.138975
C	5.265809	8.401132	10.102670
H	5.871743	7.606529	9.669814

C	4.573633	9.267800	9.239920
C	4.768494	9.038927	7.792308
C	5.959006	8.319590	6.029031
H	6.141211	7.261524	5.795482
H	6.793739	8.928176	5.646553
C	4.567277	8.816746	5.545966
C	3.742567	7.655580	4.978729
H	2.754493	8.001181	4.651877
H	3.622752	6.867330	5.735085
H	4.264831	7.234817	4.104483
C	4.660332	9.961761	4.535200
H	5.104347	9.606313	3.592125
H	5.277717	10.786882	4.921367
H	3.652622	10.349287	4.321614
C	0.860110	9.636580	5.651612
S	2.381696	5.693159	8.641602
O	3.570268	6.433599	8.163428
O	2.535979	4.290382	9.005992
O	1.136930	5.965067	7.806341
C	1.068940	7.721999	6.887172
H	0.236162	8.058035	7.495735
H	2.083810	7.925841	7.195011
H	0.889647	7.219875	5.944177
C	1.932939	6.571805	10.236474
F	2.911359	6.457191	11.136212
F	0.812994	6.081828	10.758459
F	1.740721	7.884942	9.990462

m-TS1-F-b

B3LYP-D3/Def2-SVP SCF energy (au): -4206.515516
B3LYP-D3/Def2-SVP enthalpy (au): -4205.800167
B3LYP-D3/Def2-SVP free energy (au): -4205.919167
ωB97XD/Def2-TZVP/SMD(THF) SCF energy (au): -4208.539264
ωB97XD/Def2-TZVP/SMD(THF) enthalpy (au): -4207.823915
ωB97XD/Def2-TZVP/SMD(THF) free energy (au): -4207.942915

Cartesian coordinates

ATOM	X	Y	Z
P	2.773929	11.424861	8.672976
C	0.539163	10.778777	6.278663
C	-0.549025	11.534735	5.876567
Ni	2.207010	10.283499	6.894745
C	-1.285508	11.152574	4.751224
H	-2.119816	11.780219	4.429443
C	-0.928951	9.993328	4.045230

H	-1.507500	9.705634	3.162788
C	1.377474	12.078064	9.716992
H	1.776636	12.329881	10.713448
O	5.943204	8.513784	7.457611
N	3.973741	9.348129	6.818933
C	0.150480	9.205157	4.468460
C	0.303426	10.982715	9.874791
H	0.003848	10.650606	8.866945
H	0.728831	10.100783	10.379268
C	-0.931112	11.493450	10.626037
H	-0.657087	11.732426	11.670864
H	-1.687176	10.692520	10.677106
C	-1.508779	12.743032	9.953285
H	-2.392716	13.105502	10.504043
H	-1.844702	12.488180	8.933114
C	-0.448580	13.845090	9.861093
H	-0.859513	14.736875	9.359525
H	-0.162324	14.161495	10.881979
C	0.801892	13.370853	9.106744
H	1.558781	14.171120	9.123927
H	0.540761	13.192929	8.054198
C	3.949828	12.863884	8.460552
H	3.348280	13.593541	7.889488
C	4.411817	13.517539	9.773453
H	4.958657	12.769869	10.374166
H	3.548244	13.837085	10.378258
C	5.329023	14.719553	9.502436
H	4.747479	15.509883	8.993132
H	5.672099	15.150333	10.457852
C	6.526260	14.330117	8.627495
H	7.162777	13.616066	9.182190
H	7.153318	15.212692	8.418759
C	6.064480	13.678779	7.318570
H	6.932450	13.365816	6.714267
H	5.513518	14.422929	6.714709
C	5.152379	12.474231	7.583353
H	5.737109	11.686049	8.091271
H	4.791186	12.040706	6.639700
C	3.708886	10.266003	9.778263
C	3.599622	10.351275	11.174438
H	2.953981	11.104357	11.625506
C	4.294304	9.482570	12.018835
H	4.171639	9.568160	13.101259
C	5.131137	8.506904	11.478550
H	5.662026	7.808695	12.128871
C	5.269324	8.409592	10.096855

H	5.895892	7.634083	9.658871
C	4.566129	9.273592	9.240350
C	4.773047	9.067980	7.792026
C	5.991294	8.409276	6.024121
H	6.217775	7.365782	5.765135
H	6.803455	9.060402	5.663379
C	4.585875	8.865480	5.543734
C	3.786494	7.681692	4.988078
H	2.791841	8.019463	4.672622
H	3.683087	6.897173	5.750996
H	4.307414	7.266575	4.110628
C	4.643986	10.006516	4.526452
H	5.091186	9.656542	3.582767
H	5.243561	10.848358	4.903510
H	3.625952	10.368748	4.317374
C	0.847470	9.618901	5.611099
S	2.427122	5.683644	8.650123
O	3.609034	6.441099	8.181021
O	2.597491	4.280818	9.007281
O	1.185782	5.945196	7.806428
C	1.122323	7.719815	6.898875
H	0.230712	8.015555	7.440993
H	2.103978	7.954429	7.284291
H	1.035398	7.204420	5.952060
C	1.951909	6.547907	10.245753
F	2.923284	6.440692	11.153677
F	0.834575	6.037264	10.752847
F	1.742779	7.859120	10.005843
F	-0.931299	12.667653	6.520530
H	0.392273	8.281880	3.930331

o-TS1-F-b

B3LYP-D3/Def2-SVP SCF energy (au): -4206.500021
B3LYP-D3/Def2-SVP enthalpy (au): -4205.785541
B3LYP-D3/Def2-SVP free energy (au): -4205.907411
ωB97XD/Def2-TZVP/SMD(THF) SCF energy (au): -4208.528771
ωB97XD/Def2-TZVP/SMD(THF) enthalpy (au): -4207.814291
ωB97XD/Def2-TZVP/SMD(THF) free energy (au): -4207.936161

Cartesian coordinates

ATOM	X	Y	Z
P	2.802269	11.405978	8.947989
C	1.113031	10.782877	6.100505
C	0.222318	11.672132	5.499727
Ni	2.594035	10.161724	7.143623
C	0.253969	11.868575	4.118638

H	-0.470325	12.542940	3.657126
C	1.214670	11.188814	3.349502
H	1.248279	11.370002	2.270635
C	1.394426	11.847378	10.074702
H	1.760685	12.592036	10.802767
O	5.805793	7.906340	8.547833
N	4.113005	8.975334	7.559000
C	2.143844	10.312441	3.939764
C	0.913755	10.599352	10.836983
H	0.622060	9.820055	10.118763
H	1.734691	10.181220	11.442631
C	-0.292954	10.913473	11.730351
H	0.023777	11.583546	12.552765
H	-0.649470	9.977444	12.187022
C	-1.428804	11.572052	10.938979
H	-2.261156	11.833488	11.613692
H	-1.820616	10.834702	10.219816
C	-0.941603	12.817446	10.190642
H	-1.756281	13.252573	9.588169
H	-0.638022	13.594488	10.917857
C	0.246838	12.492379	9.276898
H	0.588979	13.404543	8.760901
H	-0.078996	11.803204	8.484464
C	3.437087	13.024331	8.238592
H	2.641602	13.275118	7.513553
C	3.600438	14.198556	9.214958
H	4.368252	13.944999	9.966334
H	2.663509	14.388383	9.762030
C	4.035369	15.472247	8.473265
H	3.228019	15.780796	7.784211
H	4.165337	16.297807	9.192828
C	5.324364	15.246288	7.674589
H	6.153049	15.038853	8.376983
H	5.601459	16.161213	7.124973
C	5.176730	14.064823	6.708991
H	6.123576	13.880859	6.174304
H	4.424440	14.313390	5.938353
C	4.735201	12.790459	7.439668
H	5.533064	12.469708	8.134265
H	4.593032	11.967958	6.720161
C	4.148208	10.859094	10.085244
C	4.333293	11.536284	11.302547
H	3.658100	12.348638	11.571817
C	5.355501	11.198992	12.189597
H	5.468480	11.751270	13.125646
C	6.224652	10.154346	11.872037

H	7.031755	9.876352	12.553594
C	6.050286	9.451516	10.683814
H	6.715652	8.622941	10.447415
C	5.019215	9.780604	9.776122
C	4.914530	8.918252	8.574445
C	5.440229	7.031867	7.469773
H	4.938587	6.148871	7.899408
H	6.354960	6.716074	6.949798
C	4.491003	7.883051	6.601103
C	3.273026	7.076755	6.153983
H	2.613197	7.693168	5.529329
H	2.700296	6.715597	7.019787
H	3.600197	6.206237	5.563118
C	5.221362	8.526226	5.416810
H	5.487472	7.763208	4.668522
H	6.143665	9.025976	5.753228
H	4.571128	9.278345	4.951686
C	2.081678	10.181239	5.325424
S	-1.210731	7.269731	9.057769
O	-2.059144	6.147831	9.446121
O	-1.262428	8.512020	9.849913
O	-1.196796	7.522696	7.561823
C	-0.104949	9.159678	6.942099
H	-0.434449	9.736515	7.800080
H	0.747122	8.500568	7.020458
H	-0.690312	9.146187	6.029404
C	0.544102	6.645763	9.357366
F	0.708668	6.258102	10.618470
F	1.449284	7.625667	9.106776
F	0.844334	5.623208	8.549452
F	-0.745904	12.281912	6.212819
H	2.896757	9.816532	3.319403

References

(1) *Generation of Arynes via Ate Complexes of Arylboronic Esters with an ortho-Leaving Group | Organic Letters*. https://pubs-acs-org.ezp2.lib.umn.edu/doi/abs/10.1021/ol401140d (accessed 2020-10-08).

(2) Denman, B. N.; Plasek, E. E.; Roberts, C. C. Ligand-Induced Regioselectivity in Metal-Catalyzed Aryne Reactions Using Borylaryl Triflates as Aryne Precursors. *Organometallics* **2023**, *42* (10), 859–864. https://doi.org/10.1021/acs.organomet.3c00103.

(3) Wüstenberg, B.; Pfaltz, A. Homogeneous Hydrogenation of Tri- and Tetrasubstituted Olefins: Comparison of Iridium-Phospinooxazoline [Ir-PHOX] Complexes and Crabtree Catalysts with Hexafluorophosphate (PF_6) and Tetrakis[3,5-bis(Trifluoromethyl)Phenyl]Borate (BAr_F) as Counterions. *Adv. Synth. Catal.* **2008**, *350* (1), 174–178. https://doi.org/10.1002/adsc.200700438.

(4) Elgrishi, N.; Rountree, K. J.; McCarthy, B. D.; Rountree, E. S.; Eisenhart, T. T.; Dempsey, J. L. A Practical Beginner's Guide to Cyclic Voltammetry. *J. Chem. Educ.* **2018**, *95* (2), 197–206. https://doi.org/10.1021/acs.jchemed.7b00361.

(5) Sumida, Y.; Sumida, T.; Hashizume, D.; Hosoya, T. Preparation of Aryne–Nickel Complexes from Ortho-Borylaryl Triflates. *Org. Lett.* **2016**, *18* (21), 5600–5603. https://doi.org/10.1021/acs.orglett.6b02831.

(6) Retbøll, M.; Edwards, A. J.; Rae, A. D.; Willis, A. C.; Bennett, M. A.; Wenger, E. Preparation of Benzyne Complexes of Group 10 Metals by Intramolecular Suzuki Coupling of *o Rtho* -Metalated Phenylboronic Esters: Molecular Structure of the First Benzyne-Palladium(0) Complex. *J. Am. Chem. Soc.* **2002**, *124* (28), 8348–8360. https://doi.org/10.1021/ja0264091.

(7) Umanzor, A.; Garcia, N. A.; Roberts, C. C. Ligand-Controlled Regioinduction in a PHOX-Ni Aryne Complex. *ACS Org. Inorg. Au* **2024**, *4* (1), 97–101. https://doi.org/10.1021/acsorginorgau.3c00046.

(8) Fulmer, G. R.; Miller, A. J. M.; Sherden, N. H.; Gottlieb, H. E.; Nudelman, A.; Stoltz, B. M.; Bercaw, J. E.; Goldberg, K. I. NMR Chemical Shifts of Trace Impurities: Common Laboratory Solvents, Organics, and Gases in Deuterated Solvents Relevant to the Organometallic Chemist. *Organometallics* **2010**, *29* (9), 2176–2179. https://doi.org/10.1021/om100106e.

(9) Singh, K.; Singh, R.; Hazari, A. S.; Adhikari, D. Bimodal Photocatalytic Behaviour of a Zinc β-Diketiminate: Application to Trifluoromethylation Reactions. *Chem. Commun.* **2022**, *58* (27), 4384–4387. https://doi.org/10.1039/D2CC00397J.

(10) APEX4, APEX 5, Bruker Analytical X-ray Systems, Madison, WI (2016).

(11) Krause, L.; Herbst-Irmer, R.; Sheldrick, G. M.; Stalke, D. *J. Appl. Cryst.* **2015**, *48*, 3–10. doi:10.1107/S1600576714022985.

(12) SAINT, Bruker AXS Inc., Madison, Wisconsin, USA.

(13) Sheldrick, G. M. *Acta Cryst.* **2015**, *A71*, 3–8. doi:10.1107/S2053273314026370.

(14) Sheldrick, G. M. *Acta Cryst*. **2015**, *C71*, 3–8. doi:10.1107/S2053229614024218.
(15) Hubschle, C. B.; Sheldrick, G. M.; Dittrich, B. ShelXle: a Qt graphical user interface for SHELXL. *J. Appl. Cryst.* **2011**, *44*, 1281-1284. doi:10.1107/S0021889811043202
(16) Kratzert, D. FinalCif, V139, https://dkratzert.de/finalcif.html.
(17) Macrae, C.F.; Sovago, I.; Cottrell, S. J.; Galek, P. T. A.; McCabe, P.; Pidcock, E.; Platings, M.; Shields, G. P.; Stevens, J.S.; Towler, M.; Wood, P. A. Mercury 4.0: from visualization to analysis, design and prediction. *J. Appl. Cryst.*, **2020**, *53*, 226-235. doi:10.1107/S1600576719014092
(18) Spek, A.L. PLATON SQUEEZE: a tool for the calculation of disordered solvent contribution to the calculated structure factors. *Acta Cryst.*, **2015**, *C71*, 9-18. doi:10.1107/S2053229614024929
(19) Kratzert, D.; Krossing, I. Recent improvements in DSR. *J. Appl. Cryst.*, **2018**, *51*, 928-934. doi:10.1107/S1600576718004508

(20) Gaussian 16, Revision C.01, M. J. Frisch, G. W. Trucks, H. B. Schlegel, G. E. Scuseria, M. A. Robb, J. R. Cheeseman, G. Scalmani, V. Barone, G. A. Petersson, H. Nakatsuji, X. Li, M. Caricato, A. V. Marenich, J. Bloino, B. G. Janesko, R. Gomperts, B. Mennucci, H. P. Hratchian, J. V. Ortiz, A. F. Izmaylov, J. L. Sonnenberg, D. Williams-Young, F. Ding, F. Lipparini, F. Egidi, J. Goings, B. Peng, A. Petrone, T. Henderson, D. Ranasinghe, V. G. Zakrzewski, J. Gao, N. Rega, G. Zheng, W. Liang, M. Hada, M. Ehara, K. Toyota, R. Fukuda, J. Hasegawa, M. Ishida, T. Nakajima, Y. Honda, O. Kitao, H. Nakai, T. Vreven, K. Throssell, J. A. Montgomery, Jr., J. E. Peralta, F. Ogliaro, M. J. Bearpark, J. J. Heyd, E. N. Brothers, K. N. Kudin, V. N. Staroverov, T. A. Keith, R. Kobayashi, J. Normand, K. Raghavachari, A. P. Rendell, J. C. Burant, S. S. Iyengar, J. Tomasi, M. Cossi, J. M. Millam, M. Klene, C. Adamo, R. Cammi, J. W. Ochterski, R. L. Martin, K. Morokuma, O. Farkas, J. B. Foresman, and D. J. Fox, Gaussian, Inc., Wallingford CT, 2016.

(21) (a) Lee, C.; Yang, W.; Parr, R. G. Development of the Colle-Salvetti correlation-energy formula into a functional of the electron density. *Phys. Rev. B.*, **1988**, *37*, 785−789. (b) Becke, A. D. Density-functional thermochemistry. III. The role of exact exchange. *J. Chem. Phys.*, **1993**, *98*, 5648−5652. (c) Grimme, S.; Antony, J.; Ehrlich, S.; Krieg, H. A consistent and accurate ab initio parametrization of density functional dispersion correction (DFT-D) for the 94 elements H-Pu. *J. Chem. Phys.*, **2010**, *132*, 154104.

(22) Weigend, F.; Ahlrichs, R. Balanced basis sets of split valence, triple zeta valence and quadruple zeta valence quality for H to Rn: Design and assessment of accuracy. *Phys. Chem. Chem. Phys.*, **2005**, *7*, 3297-3305.

(23) Chai, J.-D.; Head-Gordon, M. Long-range corrected hybrid density functionals with damped atom-atom dispersion corrections. *Phys. Chem. Chem. Phys.*, **2008**, *10*, 6615.

24) Marenich, A. V.; Cramer, C. J.; Truhlar, D. G. Universal Solvation Model Based on Solute Electron Density and on a Continuum Model of the Solvent Defined by the Bulk Dielectric Constant and Atomic Surface Tensions. *J. Phys. Chem. B*, **2009**, *113*, 6378.

(25) (a) Luchini, G.; Alegre-Requena, J. V.; Funes-Ardoiz, I.; Paton, R. S. GoodVibes: Automated Thermochemistry for Heterogeneous Computational Chemistry Data. *F1000Research*, **2020**, 9, 291.
(b) Goodvibes github documentation:
https://goodvibespy.readthedocs.io/en/latest/source/README.html#examples

(26) Bickelhaupt, F. M.; Houk, K. N. Analyzing Reaction Rates with the Distortion/Interaction-Activation Strain Model. *Angew. Chem. Int. Ed.*, **2017**, *56*(34), 10070-10086.
